# Prenatal vitamin D supplementation reduces risk of asthma/recurrent wheeze in early childhood: A combined analysis of two randomized controlled trials

**DOI:** 10.1371/journal.pone.0186657

**Published:** 2017-10-27

**Authors:** Helene M. Wolsk, Bo L. Chawes, Augusto A. Litonjua, Bruce W. Hollis, Johannes Waage, Jakob Stokholm, Klaus Bønnelykke, Hans Bisgaard, Scott T. Weiss

**Affiliations:** 1 COPSAC, Copenhagen Prospective Studies on Asthma in Childhood, Herlev and Gentofte Hospital, University of Copenhagen, Copenhagen, Denmark; 2 Channing Division of Network Medicine, Department of Medicine, Brigham and Women’s Hospital, Boston, MA, United States of America; 3 Harvard Medical School, Boston, MA, United States of America; 4 Department of Pediatrics, Medical University of South Carolina, Charleston, SC, United States of America; University of Ottawa, CANADA

## Abstract

**Background:**

We recently published two independent randomized controlled trials of vitamin D supplementation during pregnancy, both indicating a >20% reduced risk of asthma/recurrent wheeze in the offspring by 3 years of age. However, neither reached statistical significance.

**Objective:**

To perform a combined analysis of the two trials and investigate whether maternal 25-hydroxy-vitamin D (25(OH)D) level at trial entry modified the intervention effect.

**Methods:**

VDAART (N = 806) and COPSAC_2010._ (N = 581) randomized pregnant women to daily high-dose vitamin D_3_ (4,000 IU/d and 2,400 IU/d, respectively) or placebo. All women also received a prenatal vitamin containing 400 IU/d vitamin D_3_. The primary outcome was asthma/recurrent wheeze from 0-3yrs. Secondary end-points were specific IgE, total IgE, eczema and lower respiratory tract infections (LRTI). We conducted random effects combined analyses of the treatment effect, individual patient data (IPD) meta-analyses, and analyses stratified by 25(OH)D level at study entry.

**Results:**

The analysis showed a 25% reduced risk of asthma/recurrent wheeze at 0-3yrs: adjusted odds ratio (aOR) = 0.74 (95% CI, 0.57–0.96), p = 0.02. The effect was strongest among women with 25(OH)D level ≥30ng/ml at study entry: aOR = 0.54 (0.33–0.88), p = 0.01, whereas no significant effect was observed among women with 25(OH)D level <30ng/ml at study entry: aOR = 0.84 (0.62–1.15), p = 0.25. The IPD meta-analyses showed similar results. There was no effect on the secondary end-points.

**Conclusions:**

This combined analysis shows that vitamin D supplementation during pregnancy results in a significant reduced risk of asthma/recurrent wheeze in the offspring, especially among women with 25(OH)D level ≥ 30 ng/ml at randomization, where the risk was almost halved. Future studies should examine the possibility of raising 25(OH)D levels to at least 30 ng/ml early in pregnancy or using higher doses than used in our studies.

**Trial registration:**

COPSAC_2010_: ClinicalTrials.gov NCT00856947; VDAART: ClinicalTrials.gov NCT00920621

## Introduction

There are several reasons to hypothesize that low *in utero* vitamin D exposure increases risk of childhood asthma/recurrent wheeze: (1) Genetic studies have shown that polymorphisms in the vitamin D receptor are associated with childhood asthma susceptibility[1]. (2) Vitamin D has an impact on fetal developmental processes influencing both lung maturation[2] and the immune system[3]. (3) Epidemiological studies have shown that high dietary vitamin D intake during pregnancy reduces the risk of asthma/recurrent wheeze in the offspring[4,5]. (4) A meta-analysis of available observational cord blood studies showed decreased risk of asthma/recurrent wheeze by increasing cord blood 25-hydroxy-vitamin D (25(OH)D) level[6].

Therefore, we recently conducted two independent randomized controlled trials (RCTs) of increased vitamin D supplementation during pregnancy to prevent childhood asthma/recurrent wheeze in the offspring by age 3 years: the Vitamin D Antenatal Asthma Reduction Trial (VDAART[7]) and the Copenhagen Prospective Studies on Asthma in Childhood 2010 (COPSAC_2010_[8]). Although both trials indicated a clinically important 20% or greater reduced risk of asthma/recurrent wheeze in the offspring, neither found this effect statistically significant in the primary intent-to-treat analyses[9,10].

A recent meta-analysis suggested that insufficient power due to an overestimation of the treatment effect may have been one of the issues for the non-significance of the findings[11], but we hypothesized that maternal 25(OH)D level at trial entry may also have influenced the effect of the vitamin D supplementation[12]. We therefore conducted a combined analysis of the two trials including the primary end-point asthma/recurrent wheeze by age 3 years as well as the secondary end-points specific IgE, total IgE, eczema and lower respiratory tract infections (LRTI) and did 25(OH)D level-based analyses to determine whether nutritional status at entry to the trials influenced the effect of vitamin D.

## Methods

VDAART: Monitored by NHLBI and COPSAC: approved by the Ethics Committee for Copenhagen.

### VDAART design (clinicaltrials.gov Identifier: NCT00920621)

Pregnant women were recruited from 3 clinical sites across the United States: (1) Boston Medical Center, Boston, MA; (2) Washington University at St. Louis, St. Louis, MO; and (3) Kaiser Permanente Southern California Region, San Diego, CA, as previously detailed[7]. Eligible participants were pregnant non-smoking women aged 18 to 39 years in estimated gestational age 10 to 18 weeks, who had a history of asthma, eczema, or allergic rhinitis or conceived the child with a man with a history of such diseases[7,9], excluding women with other chronic disorders.

The families were followed by telephone interviews every 3 months, capturing diagnoses, symptoms and the medication use of the child. In addition, the mother and child attended the clinics for yearly follow-up visits at age 1, 2 and 3 years. Content of VDAART prenatal and postnatal visits is previously reported[7].

#### VDAART vitamin D intervention

The women were randomized 1:1, between 10–18 weeks of gestation, to a daily intake of 4,000 IU vitamin D_3_ plus a multivitamin containing 400 IU vitamin D_3_ or a matching placebo tablet plus a multivitamin containing 400 IU vitamin D_3_, i.e. 4,400 vs. 400 IU vitamin D_3_ supplement per day. The intervention was continued until delivery. Adherence was measured by MEMS^®^ (Medication Events Monitoring Systems) caps, an electronic cap that recorded each time the tablet container was opened[7].

Blood was drawn from the mothers at entry to the trial and at the 3^rd^ trimester visit (32 to 38 weeks of gestation) for measurement of circulating levels of 25(OH)D, determined by using the DiaSorin Liaison® chemiluminescence immunoassay[13].

### COPSAC_2010_ design (COPSAC_2010_: clinicaltrials.gov identifier: NCT00856947)

Pregnant women were recruited from Zealand, Denmark between 2008–2010, by phone calls utilizing a monthly surveillance of reimbursement to general practitioners for the first pregnancy visit, as previously detailed[10]. Exclusion criteria were gestational age >26^th^ week, any endocrine, cardiovascular, or nephrological disorders or vitamin D intake >600 IU/day[8].

The children were followed at the COPSAC research unit by the study pediatricians with scheduled visits at 1 week, 1, 3, 6, 12, 18, 24, 30, and 36 months, and with additional unscheduled visits at any respiratory, allergy or skin-related symptoms. In addition, symptoms between visits were captured with daily diary cards monitoring: (1) troublesome lung symptoms, including components of cough, wheeze, and dyspnea; (2) skin symptoms; and (3) respiratory infections. The COPSAC pediatricians acted as general practitioners for the cohort and were the sole responsible physicians for diagnosis and treatment of asthma, allergy, and eczema adhering to predefined algorithms[8].

#### COPSAC_2010_ vitamin D intervention

The women were randomized 1:1 at gestational week 22–26 to a daily dose of 2,400 IU Vitamin D_3_ plus a multivitamin containing 400 IU vitamin D_3_ or matching placebo tablets plus a multivitamin containing 400 IU vitamin D_3_, i.e. 2,800 vs. 400 IU vitamin D_3_ supplement per day.

The 25(OH)D level of the mother was measured by isotope dilution liquid chromatography-tandem mass spectrometry (LC-MS/MS)[14,15] at time of randomization and at completion of the RCT at 1 week postpartum, allowing for assessment of adherence to the treatment plan, which was further scrutinized by counting returned tablets.

#### Additional RCTs

In COPSAC_2010_ the pregnant women concomitantly participated in a nested, factorial designed, double-blind, RCT of 2.4 g/day n-3 LCPUFA (ClinicalTrials.gov: NCT00798226)[16]. There was no interaction between the vitamin D and PUFA interventions on risk of asthma/recurrent wheeze[16]. Furthermore, a smaller subgroup participated in a randomized, participant-blinded comparison of influenza A vaccines[8,17] (ClinicalTrials.gov: NCT01012557); and a minor sub-group of children participated in a double-blinded RCT of azithromycin vs. placebo during asthmatic episodes (ClinicalTrials.gov: NCT01233297)[18].

#### Primary end-point

The primary end-point for both trials was asthma/recurrent wheeze in the child's first three years of life.

In VDAART, this was diagnosed from parental reporting of a physician diagnosis of asthma or recurrent wheeze, defined by the occurrence of at least one of the following five conditions: (1) report of wheeze after the child's second birthday, preceded by at least one report of wheeze prior to the second birthday; (2) report of the child using asthma controller medication after the second birthday, preceded by a report of wheeze before the second birthday; (3) two or more reports of wheeze after the second birthday; (4) at least one report of wheeze and use of asthma controller medications at distinct visits after the second birthday; or (5) two distinct reports of use of asthma controller medications after the second birthday[7].

In COPSAC_2010_, the children were diagnosed during the clinical visits based on the daily symptom recordings, according to a previously validated quantitative symptom algorithm requiring all of the following criteria[19,20]: (1) five episodes of troublesome lung symptoms within 6 months, each lasting at least 3 consecutive days; (2) symptoms typical of asthma including exercise induced symptoms, prolonged nocturnal cough, and persistent cough outside common cold; (3) need for intermittent rescue use of inhaled β2-agonist; and (4) response to a 3-month course of inhaled corticosteroids and relapse upon ended treatment[19].

#### Secondary end-points

*Total IgE* level was measured at age 3 years in VDAART and at age 6 and 18 months in COPSAC_2010_. Total IgE was analyzed as a dichotomized variable, using the median as the cut-off value.

*Specific IgE* levels were measured in VDAART at age 3 years, including *Alternaria Alternata*, *Dermatophagoides farinae*, *Dermatophagoides pteronyssinus*, German cockroach, cat and dog dander, egg, grass pollen mix, tree pollen mix, walnut tree, milk, peanut, soybean, and wheat. In COPSAC_2010_ specific IgE levels were measured at age 6 and 18 months, including milk, egg, dog and cat dander. In the meta-analysis we only included specific IgE levels of allergens analyzed in both cohorts (milk, egg, dog and cat) using specific IgE level ≥ 0.35 kU/l for any of the allergens as a dichotomized end-point.

*Eczema (0–3 years)*: In VDAART eczema was defined by parental reporting of physician diagnosed eczema, medication usage, and itchy rash with classic localization. In COPSAC_2010_ the diagnosis was performed at clinical visits by the research pediatricians, based on Hannifin and Rajka’s criteria[21].

*Lower respiratory tract infections (LRTI) (0–3 years)*: In VDAART LRTI was defined from parental reporting of physician diagnoses of bronchitis, bronchiolitis, pneumonia and croup. In COPSAC_2010_ LRTI included bronchiolitis and pneumonia, predominantly diagnosed by the COPSAC_2010_ pediatricians.

### Statistics

We created logistic regression models for all endpoints, except for LRTI, which was analyzed using a Poisson regression model. Subsequently, we performed a combined analysis of the two trials results utilizing a random-effects meta-analysis model. Odds ratios (ORs) were pooled separately, weighing estimates by the inverse of the estimate variance. The heterogeneity between the studies was tested with a Cochran Q test with a significance level of 0.10 and quantified by using I^2^ statistics. We also created regression models for all end-points utilizing individual patient data from the two trials; i.e. a IPD meta-analysis. All presented estimates are adjusted for the same covariates as in the original intent-to-treat analyses: maternal educational level, and center for VDAART, and gender, birth season and the fish-oil intervention for COPSAC_2010_.

For the primary end-point asthma/recurrent wheeze at age 0–3 years, we first analysed the effect of the intervention (vitamin D vs. placebo). Second, to investigate the influence of nutritional status at entry to the trial, we conducted stratified analyses of the intervention among women with 25(OH)D ≥30ng/ml and <30 ng/ml at randomization. Third, we cross-classified the women using 25(OH)D level at randomization (above or below 30 ng/ml) combined with the assigned treatment (vitamin D or placebo). For the secondary end-points, we performed analyses of the effect of the intervention (vitamin D vs. placebo).

The main purpose of the study was to conduct a combined analysis of the effect estimates and individual patient data from our own two large RCTs (COPSAC_2010_ and VDAART), which are very aligned with respect to vitamin D dosage, time of intervention during pregnancy, number of enrolled women, and offspring outcome definition[9,10]. However, a literature search revealed one additional trial of vitamin D supplementation in pregnancy and subsequent asthma/recurrent wheeze performed by Goldring et al., which differed markedly from our trials by enrolling 180 women at pregnancy week 27 and randomizing them to either no vitamin D, 800 IU ergocalciferol daily until delivery or a single oral bolus of 200,000 IU cholecalciferol[22]. We only included this trial in an exploratory combined analysis of asthma/recurrent wheeze at age 3 years.

We used the statistical software R v. 3.2.3[23] and CRAN package meta v. 4.3.0[24].

## Results

**Fig 1:**
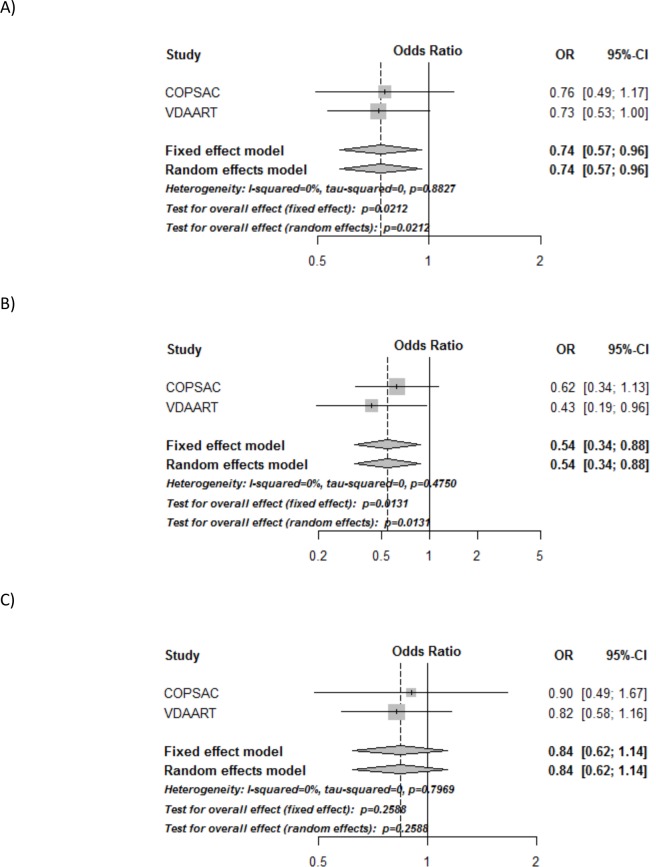
A) The VDAART participants flow, B) The COPSAC_2010_ participants flow.

For details on included and excluded participants, see the CONSORT flow diagram for each cohort (Fig 1A and 1B).

The vitamin D intervention resulted in a significant increase in the mothers post-interventional 25(OH)D levels in both trials (Table 1): in VDAART an increase from 23ng/ml to 39ng/ml and in COPSAC_2010_ an increase from 31ng/ml to 43ng/ml, respectively.

**Table 1:** Characteristics of the two trials.

Trial	No.	Intervention	Duration of intervention	Follow-up	Inclusion/ Exclusion criteria	25(OH)D levels: Control group (pre- to post-intervention)	25(OH)D levels: Treatment group (pre- to post-intervention)	Asthma/ wheeze estimates (95% CI)
**VDAART, double blinded, multicenter RCT, USA (2009)**	806	Active: 4,000 + 400 IU/d Placebo:400 IU/d	From 10–18 weeks of gestation until birth	3 years of follow-up by quarterly phone calls and annual visits to the clinic	Inclusion: Parental asthma, allergy or eczema Exclusion: Maternal chronic disease	23 ng/ml to 27 ng/ml	23 ng/ml to 39 ng/ml	HR: 0.8 (0.6–1.00) OR: 0.72 (0.52–1.00)
**COPSAC**_**2010**_**, double blinded, single center RCT, Denmark (2008)**	581	Active: 2,400 + 400 IU/d Placebo: 400 IU/d	From 22–26 weeks of gestation until 1 week postpartum	3 years of follow-up with visits to the clinic at age 1 week, 1, 3, 6, 12, 18, 24, 30, 36 months and at acute airway symptoms, plus a daily diary	Inclusion: No criteria Exclusion: Maternal chronic disease	31 ng/ml to 29 ng/ml	31 ng/ml to 43 ng/ml	HR: 0.76 (0.52–1.12) OR: 0.77 (0.5–1.18)

In VDAART, 625 women (78%) had an initial 25(OH)D level <30 ng/ml (307 received vitamin D and 318 placebo) and 176 women (22%) had a level ≥30 ng/ml at trial entry (95 received vitamin D and 81 placebo). Correspondingly, in COPSAC, 276 women (48%) had an initial level <30 ng/ml (139 received vitamin D and 137 placebo) and 301 women (52%) had an initial level of 25(OH)D ≥30 ng/ml (153 received vitamin D and 148 placebo).

### Primary end-point

#### Asthma/recurrent wheeze

A total of 218 (29%) children were diagnosed with asthma/recurrent wheeze in the VDAART trial and 104 (18%) in COPSAC_2010_ during the first 3 years of life (**Table 2**).

**Table 2:** End-points in the two trials at age 0–3 years.

End-point:	COPSAC_2010_ (581)	VDAART (806)
Asthma/recurrent wheeze	18% (104 of 581)	29% (218 of 748)
Eczema	25% (143 of 581)	23% (172 of 751)
LRTI	24% (139 of 581)	33% (266 of 806)
Specific IgE ≥ 0.35 kUa/L: milk, egg, cat, dog	8% (39 of 471)	39% (212 of 544)
Median (IQR) total IgE, kU/L	4.56 (2.50–8.79)	31.5 (11.5–103)

**Fig 2:**
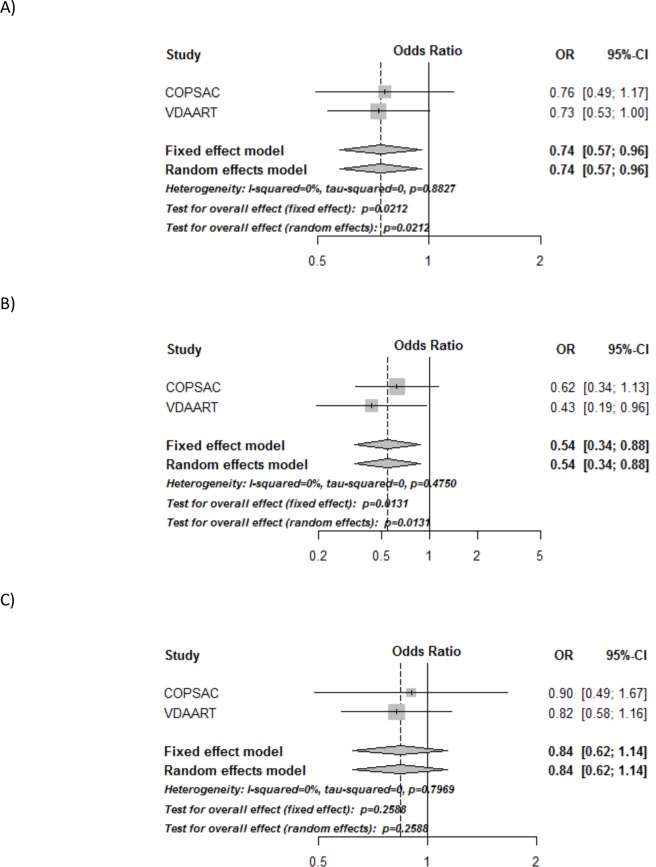
Forest plots of the combined analyses of asthma/recurrent wheeze in COPSAC_2010_ and VDAART. A) Combined analysis of the effect of randomization group, B) Combined analysis of women with initial 25(OH)D level ≥ 30 ng/ml, C) Combined analysis of women with initial 25(OH)D level < 30 ng/ml. All analyses are adjusted for gender, birth season and fish-oil intervention in COPSAC_2010_, and center and maternal education level in VDAART. All analyses of the average 25(OH)D level are also adjusted for intervention group. p<0.05 is shown in italic.

A combined analysis of the calculated odds-ratios from the separate trials showed a significant 26% reduced risk of asthma/recurrent wheeze in the vitamin D vs. control group: OR = 0.74 (95% CI, 0.57 to 0.96), p = 0.02 (Fig 2A and Table 3), with no heterogeneity among the trials: I^2^ = 0%, p = 0.89.

**Table 3:** Individual study results in VDAART and COPSAC_2010_ and the combined analyses of the primary end-point asthma/recurrent wheeze at age 0–3. All analyses are adjusted for gender, birth season and fish-oil intervention in COPSAC_2010_, and center and maternal education level in VDAART. p<0.05 is shown in italic. *Random effects meta-analysis.

**Asthma/recurrent wheeze**	**COPSAC**_**2010**_**OR (95% CI)**	**VDAARTOR (95% CI)**	Combined analysis*OR (95% CI)
Active treatment	0.76 (0.49–1.17)	0.73 (0.53–1.00)	*0*.*74 (0*.*57–0*.*96) p = 0*.*02*
Stratified ≥ 30 ng/ml	0.62 (0.34–1.13)	*0*.*43 (0*.*19–0*.*95)*	*0*.*54 (0*.*3–0*.*88) p = 0*.*01*
Stratified < 30 ng/ml	0.90 (0.49–1.67)	0.82 (0.58–1.17)	0.84 (0.62–1.14) p = 0.26
Interaction: ≥ 30 ng/ml * vitamin D	0.68 (0.29–1.62)	0.57 (0.24–1.33)	0.65 (0.36–1.20) p = 0.17
**Vitamin D categories:**	**COPSAC**_**2010**_**OR (95% CI)**	**VDAARTOR (95% CI)**	Combined analysis*OR (95% CI)
< 30 ng/ml + placebo (ref)	1	1	
< 30 ng/ml + vitamin D	0.93 (0.50–1.71)	0.82 (0.58–1.18)	0.85 (0.62–1.15) p = 0.29
≥ 30 ng/ml + placebo	1.13 (0.63–2.03)	1.01 (0.56–1.81)	1.07 (0.71–1.62)p = 0.75
≥ 30 ng/ml + vitamin D	0.72 (0.38–1.33)	0.47 (0.25–0.89)	0.58 (0.37–0.91) p = 0.02

In the analysis stratified for 25(OH)D level at trial entry, the offspring of women with 25(OH)D level ≥30ng/ml had a significantly reduced risk of asthma/recurrent wheeze: OR = 0.54 (0.33 to 0.88), p = 0.01, with little variation among the trials: I^2^ = 0% and p = 0.47, whereas no significant effect was observed among women with a level <30ng/ml: OR = 0.84 (0.62 to 1.15), p = 0.25, I^2^ = 0%, p = 0.79. However, no significant interaction between 25(OH)D level at trial entry (used as a dichotomized variable with ≥/< 30ng/ml as the cut-off) and randomization group was observed (p = 0.17), I^2^ = 0%, p = 0.66 (Fig 2B and 2C and Table 3).

An analysis of initial 25(OH)D level and randomization group, showed a lower risk of asthma/recurrent wheeze in women with 25(OH)D level ≥ 30ng/ml and randomized to vitamin D (women with initial 25(OH)D <30 ng/ml and randomized to placebo as reference), OR = 0.58 (0.37–0.91), p = 0.02, I^2^ = 0%, p = 0.35. There was no such association for women with 25(OH)D level ≥30ng/ml randomized to placebo or women with 25(OH)D level <30ng/ml randomized to vitamin D (**Table 3**).

**Fig 3:**
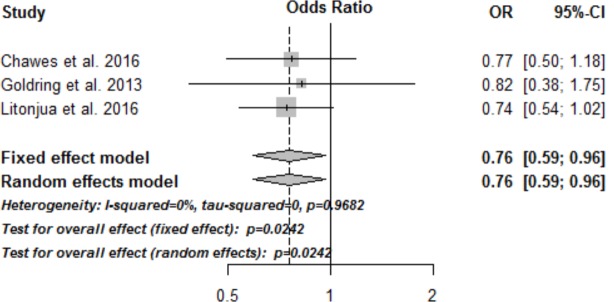
Forest plots of the meta-analysis of asthma/recurrent wheeze in all three published randomized trials of vitamin D intervention in pregnancy and subsequent asthma/recurrent wheeze at age 3 years.

An exploratory analysis of the primary end-point, asthma/recurrent wheeze, including the third vitamin D trial by Goldring et al. showed similar results with a significantly reduced risk of asthma/wheeze at 3 years of age in the vitamin D intervention group, compared to controls: OR = 0.76 (95% CI, 0.59–0.96), p = 0.02 (**Fig 3**), with little variation among the included studies: I^2^ = 0%, p = 0.97.

### Secondary end-points

#### Total IgE

The median (interquartile range (IQR)) level of total IgE was 31.5 (11.5 to 103) kU/l in VDAART and 4.56 (2.50 to 8.79) kU/l in COPSAC_2010_ (**Table 2**). The combined analysis of the vitamin D intervention showed no effect on development of elevated total IgE level (above the median): OR = 0.90 (0.71 to 1.16), p = 0.46 (**Table 4**), I^2^ = 0%, p = 0.46. Analyzing total IgE level as a continuous variable yielded similar results (data not shown).

**Table 4:** Individual study results in VDAART and COPSAC_2010_ and combined analyses of the effect of vitamin D vs. placebo on the secondary end-points at age 0–3. All analyses are adjusted for gender, birth season and fish-oil intervention in COPSAC_2010_, and center and maternal education level in VDAART. Lower respiratory tract infections (LRTI) are analyzed by Poisson regression model. *Random effects meta-analysis.

Vitamin D vs. Placebo	COPSAC_2010_OR (95% CI)	VDAARTOR (95% CI)	Combined analysis*OR (95% CI)
Total IgE > median	0.95 (0.68–1.34)	0.87 (0.62–1.23)	0.90 (0.71–1.16)p = 0.46
Specific IgE > 0.35kU/l	1.50 (0.76–2.97)	0.93 (0.65–1.31)	1.08 (0.69–1.67)p = 0.78
Eczema	0.92 (0.63–1.35)	0.89 (0.63–1.25)	0.90 (0.70–1.17)p = 0.44
LRTI	1.17 (0.81–1.71))	0.80 (0.62–1.03)	0.94 (0.65–1.37)p = 0.76

#### Specific IgE

A specific IgE level ≥0.35kU/l against milk, egg, cat and/or dog was present in 212(39%) children in the VDAART cohort at age 3 years; 61 children had elevated specific IgE against inhalant allergens and 193 against food allergens. In COPSAC_2010_, 39(8%) of the children had elevated specific IgE at ages 6 and/or 18 months; 10 had specific IgE against inhalant allergens and 31 against food allergens (**Table 2**). The combined analysis showed no effect of the vitamin D intervention on the risk of developing elevated specific IgE: OR = 1.08 (0.69 to 1.67), p = 0.78 (**Table 4**), I^2^ = 35%, p = 0.22.This also applied for analyses restricted to inhalant and food allergens (data not shown).

#### Eczema

Eczema was diagnosed in 172 (23%) children in VDAART and 143 (25%) children in COPSAC_2010_, between 0-3yrs of age (Table 2). The combined analysis showed no effect of the vitamin D intervention on the risk of developing eczema: OR = 0.90 (0.70 to 1.17), p = 0.44 (Table 4), I^2^ = 0%, p = 0.88.

#### LRTI

A total of 266 (39%) children in VDAART and 139 (24%) in COPSAC_2010_ developed one or more LRTI from 0–3 years of age (Table 2). Using a Poisson regression model, we found no effect on the risk of developing one or more LRTI in the combined analysis of the vitamin D intervention: OR = 0.94 (0.65 to 1.37), p = 0.76 (Table 4), I^2^ = 63%, p = 0.10.

#### Individual patient data meta-analysis

Additionally, we conducted individual patient data meta-analysis for all end-points. These analyses yielded results similar as the random effects meta-analyses (Table 5), with OR = 0.75 (0.57 to 0.97), p = 0.03 for development of asthma/recurrent wheeze.

**Table 5:** Meta-analyses of individual patient data (IPD), of vitamin D vs. placebo on the primary and secondary end-points at age 0–3. All analyses are adjusted for gender, birth season, fish-oil intervention, center and maternal education level. Lower respiratory tract infections (LRTI) are analyzed by Poisson regression model.

Vitamin D vs. Placebo	IPD OR (95% CI)
Asthma/ recurrent wheeze	0.75 (0.57–0.97) p = 0.03
Total IgE > median	0.93 (0.73–1.18) p = 0.43
Specific IgE > 0.35kU/l	1.05 (0.77–1.43) P = 0.78
Eczema	0.91 (0.71–1.18) P = 0.47
LRTI	0.92 (0.75–1.14) p = 0.44

## Discussion

In this combined analysis of the two largest RCTs to date of vitamin D supplementation during pregnancy, we showed that the supplementation resulted in a significant and clinically important 26% reduced risk of asthma/recurrent wheeze in the offspring until age 3, with a more pronounced 46% reduced risk if the women had 25(OH)D level ≥30ng/ml at trial entry. These results underline that vitamin D supplementation during pregnancy may be an important factor in the prevention of childhood asthma. The findings are suggestive that optimal effect from supplementation may be obtained by assuring baseline levels of at least 30 ng/ml in early pregnancy.

### Strengths and limitations

The VDAART and COPSAC_2010_ RCTs both had asthma/recurrent wheeze at age 0–3 years as primary end-point and had a range of mutual secondary end-points including total IgE, specific IgE, eczema and LRTI. Thus, the aligned design with respect to follow-up length and predefined end-points is a major strength of this combined analysis. This resulted in more power to detect true effects of the vitamin D supplementation than the individual intent-to-treat analyses, illustrated by a similar overall effect estimate in the analyses, which now reached statistical significance.

The VDAART and COPSAC_2010_ trials used different doses of vitamin D, initiated the supplementation at different pregnancy stages ranging from 10 to 26 weeks of gestation, and enrolled pregnant women with divergent 25(OH)D levels at entry into the trials. Despite these differences, the trials similarly showed that the vitamin D supplementation resulted in significantly increased post-interventional 25(OH)D levels in the mothers. Furthermore, taking advantage of these differences in the combined analysis by utilizing the measured 25(OH)D levels at study entry, unmasked the most pronounced protective effect of the vitamin D intervention in women with 25(OH)D level ≥ 30 ng/ml.

VDAART is a high-risk cohort, with parental asthma or allergy as inclusion criteria. This is in contrast to the unselected COPSAC_2010_ cohort, which may explain the overall higher frequencies of the primary and secondary end-points in VDAART, compared to COPSAC_2010_. Particularly, total IgE levels were higher and elevated specific IgE was much more prevalent in VDAART (34%) vs. COPSAC_2010_ (8%), which can also be ascribed the different ages of measurement (3 years in VDAART vs. 6–18 months in COPSAC_2010_).

Finally, VDAART included a large group of African-American women in the trial, who are well-known to have a higher risk of developing asthma and allergy[25] and had lower vitamin D levels at randomization compared to the other ethnic groups[12]. In contrast, COPSAC_2010_ primarily recruited Caucasians to the trial. However, this unequal distribution of races in the trials did not affect the individual intent-to-treat analyses, which yielded very similar effect estimates.

We conducted both random effects meta-analyses and individual patient data (IPD) analysis. These yielded similar results, increasing the confidence in our findings. Furthermore, including a third trial of vitamin D supplementation in the meta-analysis, which intervened at a later pregnancy stage (week 27) and with a much lower dose (800 IU/day) [22] also showed an overall protective effect of vitamin D on development of asthma/recurrent wheeze.

### Interpretation

This combined analysis of our two vitamin D RCTs showed a significant 26% reduced risk of the primary end-point asthma/recurrent wheeze during the first 3 years of life, which suggests that the non-significant/borderline significant trial responses observed in the individual intent-to-treat analyses were partly due to lack of power caused by an overestimation of the treatment effect, rather than lack of an intervention effect.

A recent meta-analysis supports our finding of a significant reduction in asthma/wheeze from prenatal vitamin D supplementation[11]. However, unlike drug trials, in nutrient trials, participants have varying levels of the nutrient in the body when entering the trial[26]. Thus, 25(OH)D level in the pregnant mother at enrollment into the trial could be an important effect modifier, which was not accounted for in the earlier reported individual intent-to-treat analyses[9,10]. To explore that issue, we performed the combined analyses stratified for the women’s 25(OH)D level at trial entry, which showed that the vitamin D supplement resulted in a significantly reduced risk of asthma/recurrent wheeze in the offspring of women with 25(OH)D ≥ 30 ng/ml, but a much smaller and statistically non-significant effect among women with 25(OH)D < 30 ng/ml. This finding is contrary to what many have suggested that only those who are deficient in vitamin D would benefit from supplementation. This finding suggests that the level of deficiency may be different for non-bone outcomes compared with bone outcomes, and that the level needed for optimal prevention of childhood asthma lies above 30 ng/ml, higher that what is needed for bone health[27].

When the two trials were initiated, it was thought that the effects of vitamin D were confined to the alveolar stage of lung development, occurring in the third trimester of pregnancy[28]. However, emerging evidence arising from lung gene expression data from our own lab have subsequently shown that vitamin D already has effects at the stage of branching morphogenesis of the fetal lung occurring in the first trimester of pregnancy prior to randomization in both these trials[2]. Therefore, initiating the supplementation at 10–18 weeks’ gestation in VDAART and in particular at 22–26 weeks’ gestation in COPSAC_2010_ was presumably too late to achieve a maximal effect of vitamin D on fetal lung development. In addition, as human fertility is also influenced by vitamin D[29,30], it may even be worthwhile to target supplementation before conception to optimize implantation of the fertilized egg and assure sufficient vitamin D for lung and immune development from the very beginning of life.

Our combined analysis also suggests that we may have used an insufficient dosage of vitamin D in both the trials. The Institute of Medicine has recently redefined the cut-off for sufficient 25(OH)D level from 30 ng/ml to 20 ng/ml or higher based on the effect on optimal bone health[27], but the sufficient 25(OH)D level in a mother during pregnancy for optimal immune and lung development in the fetus is unknown and might be as high as 40–60 ng/ml. We observed that the vitamin D supplement resulted in a significant increase in post-interventional maternal 25(OH)D level in both trials, but levels ≥ 30 ng/ml were only achieved in 75% of the supplemented mothers in VDAART and 82% in COPSAC_2010_ underscoring that too low a dose may have attenuated the trial results in both trials but to a greater extent in VDAART possibly because of the increased African American enrollment. The lack of effect of the vitamin D supplement among women with low 25(OH)D levels at trial entry also suggests that the dose might have been too low to reverse developmental changes caused by insufficient vitamin D exposure before the supplementation was initiated.

The secondary end-point combined analysis of total IgE confirmed the non-significant effect of the intervention reported in the individual trials[9,10]. The analysis showed also no overall effect on specific IgE levels towards the food and aeroallergens measured in both trials (egg, milk, cat and dog). This is consistent with a recent RCT showing no effect on cat specific IgE levels, whereas decreased house-dust mite specific IgE was shown in the offspring of mothers supplemented with vitamin D during pregnancy[31]. The combined analyses did not show a reduction in eczema risk or occurrence of LRTI, which is in line with previous observational studies[4,32,33].

It has already been shown that maternal vitamin D status is associated with other immune-mediated diseases such as pre-eclampsia[34] and Type I diabetes in the offspring[35]. Combining this knowledge with our findings from this combined analysis, showing that vitamin D supplementation has a protective effect on childhood asthma/recurrent wheeze, makes vitamin D a very promising nutrient for future prevention of immune-mediated disorders. Future studies should explore the optimal timing and level of vitamin D supplementation during pregnancy.

### Conclusion

This combined analysis shows a significant and clinically important 26% protective effect of vitamin D supplementation during pregnancy on the risk of asthma/recurrent wheeze in the offspring, with a more pronounced risk reduction of 46% in women with 25(OH)D level ≥ 30 ng/ml at trial entry. Prenatal care strategies targeted at raising vitamin D levels in pregnant women should therefore be considered.

## Supporting information

S1 Table VDAART consort checklist. (PDF)

S2 Table COPSAC_2010_ consort checklist. (DOCX)

S1 Text Chawes et al, JAMA paper, COPSAC_2010_. (PDF)

S2 Text Ethical committee approval, COPSAC_2010_. (PDF)

S3 Text VDAART IRB approval. (PDF)

S4 Text Litonjua et al., JAMA paper, VDAART. (PDF)

S5 Text Original protocol, VDAART. (PDF)

S6 Text Manuscript VDAART. (DOCX)

S7 Text Manuscripts COPSAC_2010_. (DOCX)

S8 Text Supplement to manuscript, COPSAC_2010_. (DOCX)

## Abbreviations

VDAART: Vitamin D Antenatal Asthma Reduction Trial
COPSAC_2010_: Copenhagen Prospective Studies on Asthma in Childhood 2010
25(OH)D: 25Hydroxyvitamin D
IU: International Units
ng/ml: nanogram per milliliter
