# Supplementary material for: Prenatal vitamin D supplementation reduces risk of asthma/recurrent wheeze in early childhood: A combined analysis of two randomized controlled trials
Source: PLoS One. 2017 Oct 27;12(10):e0186657. doi: 10.1371/journal.pone.0186657 (PMC5659607; doi:10.1371/journal.pone.0186657)
Supplement: S1 Text — (PDF) [file pone.0186657.s003.pdf]

Original Investigation

# Effect of Vitamin $D_3$ Supplementation During Pregnancy on Risk of Persistent Wheeze in the Offspring
## A Randomized Clinical Trial

Bo L. Chawes, MD, PhD; Klaus Bønnelykke, MD, PhD; Jakob Stokholm, MD, PhD; Nadja H. Vissing, MD, PhD; Elín Bjarnadóttir, MD; Ann-Marie M. Schoos, MD, PhD; Helene M. Wolsk, MD; Tine Marie Pedersen, MD; Rebecca K. Vinding, MD; Sunna Thorsteinsdóttir, MD; Lambang Arianto, MD; Henrik W. Hallas, MD; Lene Heickendorff, MD, DMSc; Susanne Brix, MSc, PhD; Morten A. Rasmussen, MSc, PhD; Hans Bisgaard, MD, DMSc

Editorial page 347

Related article page 362

Supplemental content at jama.com

**IMPORTANCE** Observational studies have suggested that increased dietary vitamin D intake during pregnancy may protect against wheezing in the offspring, but the preventive effect of vitamin D supplementation to pregnant women is unknown.

**OBJECTIVE** To determine whether supplementation of vitamin $D_3$ during the third trimester of pregnancy reduces the risk of persistent wheeze in the offspring.

**DESIGN, SETTING, AND PARTICIPANTS** A double-blind, single-center, randomized clinical trial conducted within the Copenhagen Prospective Studies on Asthma in Childhood 2010 cohort. Enrollment began March 2009 with a goal of 708 participants, but due to delayed ethical approval, only 623 women were recruited at 24 weeks of pregnancy. Follow-up of the children (N = 581) was completed when the youngest child reached age 3 years in March 2014.

**INTERVENTIONS** Vitamin $D_3$ (2400 IU/d; n = 315) or matching placebo tablets (n = 308) from pregnancy week 24 to 1 week postpartum. All women received 400 IU/d of vitamin $D_3$ as part of usual pregnancy care.

**MAIN OUTCOMES AND MEASURES** Age at onset of persistent wheeze in the first 3 years of life. Secondary outcomes included number of episodes of troublesome lung symptoms, asthma, respiratory tract infections, and neonatal airway immunology. Adverse events were assessed.

**RESULTS** Of the 581 children, persistent wheeze was diagnosed during the first 3 years of life in 47 children (16%) in the vitamin $D_3$ group and 57 children (20%) in the control group. Vitamin $D_3$ supplementation was not associated with the risk of persistent wheeze, but the number of episodes of troublesome lung symptoms was reduced, and the airway immune profile was up-regulated (principal component analysis, $P = .04$). There was no effect on additional end points. Intrauterine death was observed in 1 fetus (<1%) in the vitamin $D_3$ group vs 3 fetuses (1%) in the control group and congenital malformations in 17 neonates (5%) in the vitamin $D_3$ group vs 23 neonates (8%) in the control group.

| End Point | No. (%) Vitamin $D_3$ | Control | Estimate (95% CI) | *P* Value |
|---|---|---|---|---|
| Persistent wheeze | 47 (16) | 57 (20) | Hazard ratio (HR), 0.76 (0.52-1.12) | .16 |
| Episodes of troublesome lung symptoms, mean (95% CI) | 5.9 (5.2-6.6) | 7.2 (6.4-8.1) | Incidence risk ratio (IRR), 0.83 (0.71-0.97) | .02 |
| Asthma at 3 y | 32 (12) | 47 (14) | Odds ratio, 0.82 (0.50-1.36) | .45 |
| Respiratory tract infections | | | | |
| Upper, annual mean (95% CI) | 5.2 (4.8-5.5) | 5.3 (4.9-5.6) | IRR, 0.99 (0.90-1.09) | .84 |
| Lower | 94 (32) | 95 (33) | HR, 0.96 (0.72-1.27) | .76 |

**CONCLUSIONS AND RELEVANCE** The use of 2800 IU/d of vitamin $D_3$ during the third trimester of pregnancy compared with 400 IU/d did not result in a statistically significant reduced risk of persistent wheeze in the offspring through age 3 years. However, interpretation of the study is limited by a wide CI that includes a clinically important protective effect.

**TRIAL REGISTRATION** ClinicalTrials.gov identifier: NCT00856947.

*JAMA*. 2016;315(4):353-361. doi:10.1001/jama.2015.18318

**Author Affiliations:** Author affiliations are listed at the end of this article.

**Corresponding Author:** Hans Bisgaard, MD, DMSc, Copenhagen Prospective Studies on Asthma in Childhood, Herlev and Gentofte Hospital, University of Copenhagen, Ledreborg Alle 34, DK-2820 Gentofte, Denmark (bisgaard@copsac.com).



jamanetwork/2016/jama/26jan2016/joi150163 PAGE: right 1 SESS: 32 OUTPUT: Jan 11 17:52 2016

Asthma often begins in early childhood and is the most common chronic childhood disorder.[1] The incidence has increased during the last half-century in westernized societies, presumably related to a changing lifestyle or environment inducing immune deregulation in early life and subsequent chronic inflammation.[2] In parallel, vitamin D deficiency has also become a common health problem in westernized societies, possibly caused by a more sedentary indoor lifestyle and decreased intake of vitamin D containing foods.[3] Vitamin D possesses a range of immune regulatory properties, and it has been speculated that vitamin D deficiency during pregnancy may affect fetal immune programming and contribute to asthma pathogenesis.[4,5]

This hypothesis is supported by a recent observational study in the Copenhagen Prospective Studies on Asthma in Childhood 2000 ($COPSAC_{2000}$) high-risk birth cohort showing an association between low cord blood vitamin D levels and an increased risk of childhood wheezing.[6] Results of other studies have been ambiguous, as some showed an association between decreased maternal dietary vitamin D intake[7,8] or decreased cord blood vitamin D levels[6,9,10] and increased risk of wheezy disorders, whereas others reported no such association.[11,12]

Therefore, we conducted the COPSAC 2010 ($COPSAC_{2010}$) Vitamin D RCT, a double-blind RCT of vitamin $D_3$ supplementation during pregnancy in the 2010 population-based COPSAC mother-child cohort, to assess the potential effect on risk of persistent wheeze during the first 3 years of life.[13]

## Methods

The trial protocol and statistical analysis plan are available in Supplement 1.[14]

The $COPSAC_{2010}$ study was approved by the local ethics committee with a separate approval for the vitamin $D_3$ during pregnancy RCT, by the Danish Data Protection Agency, and by the Danish Health and Medicines Authority. Written and oral informed consent were obtained at enrollment of participants.

### Study Design

This double-blind, placebo-controlled study identified pregnant women in Denmark by reviewing monthly the lists of reimbursements to general physicians for first pregnancy visits. The identified women received a written invitation to contact the COPSAC clinic by telephone, and they were given detailed verbal information and screened for eligibility. Thereafter, detailed information was sent and the first visit to the clinic was planned within pregnancy weeks 22 through 26. Exclusion criteria were gestational age above week 26; any endocrine, cardiovascular, or nephrological disorders; or vitamin $D_3$ (cholecalciferol) intake more than 600 IU/d.[13]

The offspring were recruited to the $COPSAC_{2010}$ birth cohort and followed up by the study pediatricians with scheduled visits at 1 week, 1, 3, 6, 12, 18, 24, 30, and 36 months, and with acute visits for any respiratory or skin-related symptoms. The symptom burden between visits was captured with daily diary cards monitoring: (1) significant troublesome lung symptoms including components of cough, wheeze, and dyspnea; (2) skin symptoms; and (3) respiratory tract infections. The study pediatricians acted as general practitioners for the cohort and were solely responsible for diagnosis and treatment of persistent wheeze, asthma, allergy, and eczema adhering to predefined algorithms and blinded to the intervention.[13]

### Study Intervention

Women were randomized 1:1 to a daily dose of 2400 IU vitamin $D_3$ supplementation or matching placebo tablets (Camette A/S) from pregnancy week 24 to 1 week postpartum. In addition, all women were instructed to continue supplementation of 400 IU of vitamin $D_3$ during pregnancy as recommended by the Danish National Board of Health; thus, the study is a dose comparison of 2800 IU/d vs 400 IU/d of vitamin $D_3$ supplementation. Women were randomized using a computer-generated list of random numbers, supplied by an external investigator who had no further involvement in the RCT. The intervention code was unblinded when the youngest child reached age 3 years or in case of a medical emergency.

The mother's serum vitamin D level was measured[15,16] at time of randomization corresponding to pregnancy week 24 and at the first visit after birth (ie, 1 week postpartum) (Supplement 2), when the women stopped the supplement. This allowed assessment of adherence to the treatment plan, which was further complemented by counting returned tablets.

All the women also participated in a concomitant factorial designed, double-blind RCT of 2.4 g per day of long-chain n-3 polyunsaturated fatty acids (PUFAs) during pregnancy (ClinicalTrials.gov: NCT00798226).

### Primary End Point

Persistent wheeze was diagnosed according to a previously validated quantitative algorithm[17,18] requiring all of the following: (1) recurrent wheeze (verified diary recordings of ≥5 episodes of troublesome lung symptoms [cough, wheeze, and/or dyspnea] lasting ≥3 days within 6 months), (2) typical symptoms of asthma (eg, exercise-induced symptoms, prolonged nocturnal cough, or persistent cough outside common cold), (3) need for intermittent bronchodilator, and (4) response to a 3-month trial of inhaled corticosteroids and relapse upon cessation.[17] Risk of persistent wheeze analyzed by age-at-onset analysis (Cox proportional hazards regression model) from birth to age 3 years was the primary end point.

### Secondary End Points

Asthma was diagnosed in children fulfilling the persistent wheeze criteria at age 3 years.

Episodes of troublesome lung symptoms included the number of episodes of troublesome lung symptoms lasting 3 or more consecutive days in the first 3 years of life.

Upper respiratory tract infections included episodes of common cold, acute tonsillitis, croup, and acute otitis media until age 3 years.[13]



jamanetwork/2016/jama/26jan2016/joi150163 PAGE: left 2 SESS: 32 OUTPUT: Jan 11 17:52 2016

Figure 1. Flow of Participants Through the $COPSAC_{2010}$ Vitamin D RCT

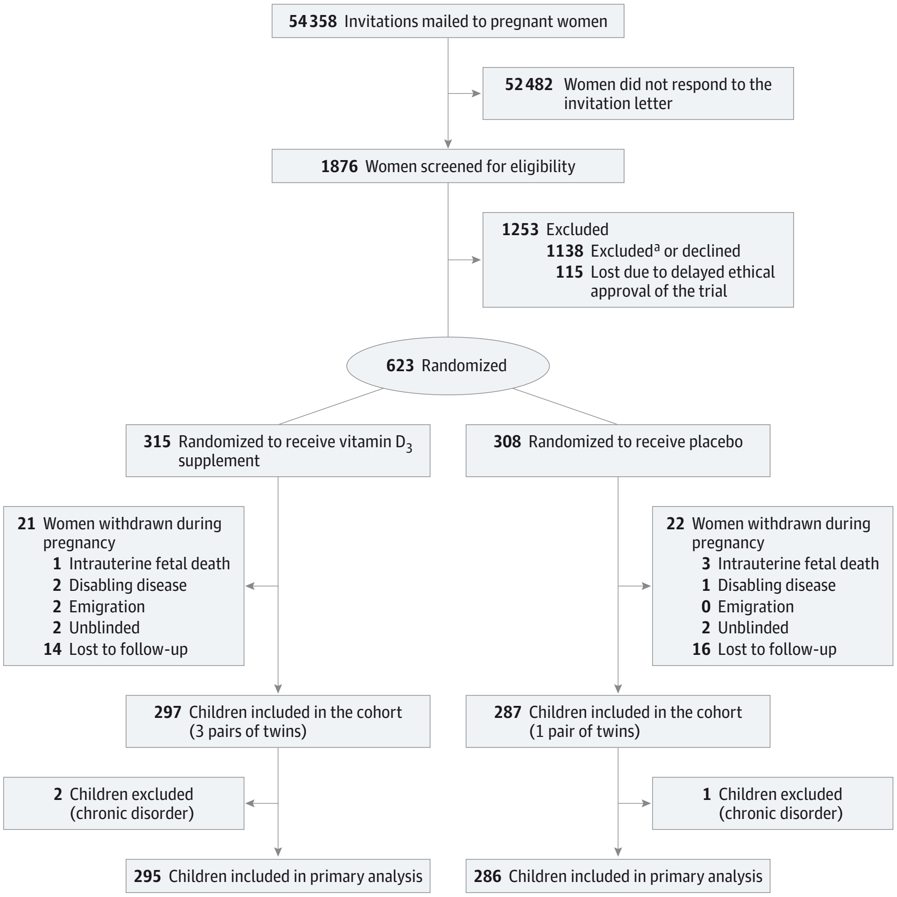


COPSAC indicates Copenhagen Prospective Studies on Asthma in Childhood; RCT, randomized clinical trial.

[a] Exclusion criterions were gestational age above week 26, any endocrine, cardiovascular, or nephrological disorders, or vitamin D intake more than 600 IU per day.

Lower respiratory tract infections included pneumonia and bronchiolitis. Pneumonia was diagnosed in children with significant cough, tachypnea, fever, and abnormal lung stethoscopy, whereas bronchiolitis was defined as cough, tachypnea, chest retractions, auscultative widespread crepitation, or rhonchi in an infant below 1 year.[19-21]

Airway immunology was assessed at age 1 month by measuring unstimulated levels of 20 cytokines and chemokines in airway mucosal lining fluid sampled by a nasosorption technique as previously detailed[22-24] (Supplement 2). The 20 cytokines and chemokines include interleukin (IL)-12p70, CXCL10 (interferon gamma-induced protein [IP]-10), interferon (IFN)-γ, tumor necrosis factor (TNF)-α, CCL4 (macrophage inflammatory protein [MIP]-1β), CCL2 (monocyte chemoattractant protein [MCP]-1), CCL13 (MCP-4), IL-4, IL-5, IL-13, CCL11 (eotaxin-1), CCL26 (eotaxin-3), CCL17 (thymus- and activation-regulated chemokine [TARC]), CCL22 (macrophage-derived chemokine [MDC]), IL-17, IL-1β, CXCL8 (IL-8), transforming growth factor (TGF)-β1, IL-10, and IL-2.

Systemic low-grade inflammation was determined by measuring serum levels of high-sensitivity C-reactive protein, IL-6, TNF-α, and CXCL8 (also known as IL-8) at age 6 months.[25]

Allergic sensitization was diagnosed at 6 and 18 months by any skin prick test (ALK-Abelló) result 2 mm or larger or specific immunoglobulin E (IgE) level of 0.35 $kU_A/L$ or higher against raw milk, pasteurized eggs, dogs, or cats (ImmunoCAP; Thermo Fischer Scientific).[13]

Eczema at age 0 through 3 years was diagnosed according to the criteria of Hanifin and Rajka[26] including typical morphology and localization of skin lesions.[27,28]

## Safety

Parents were routinely interviewed about the mother's medical history during pregnancy and the health of the child(ren) at all scheduled and unscheduled visits to the research unit. All diagnoses were registered online in the dedicated COPSAC database.

## Study Power

The prespecified sample size calculation found that 708 participants (354 in each group) would be required to obtain 80% power to detect a difference between the treatment groups (2-tailed $\alpha = .05$) based on a 12% expected frequency of persistent wheeze in the control group (estimated from the 16.5% observed in the $COPSAC_{2000}$ high-risk cohort) and an effect of 0.5 in the vitamin $D_3$ group.



jamanetwork/2016/jama/26jan2016/joi150163 PAGE: right 3 SESS: 32 OUTPUT: Jan 11 17:52 2016

Table 1. Baseline Characteristics of Mothers, Children, and Births in the $COPSAC_{2010}$ Vitamin D RCT

| | No. (%) | | |
|---|---|---|---|
| | | Randomization | |
| | All (n = 581) | Vitamin $D_3$ (n = 295 [51%]) | Control (n = 286 [49%]) |
| **Mothers** | | | |
| Socioeconomics | | | |
| Maternal | | | |
| Age at birth, mean (SD), y | 32.3 (4.3) | 32.5 (4.4) | 32.0 (4.3) |
| Asthma[a] | 152 (26) | 79 (27) | 73 (26) |
| Maternal educational level[b] | | | |
| Low | 45 (8) | 20 (7) | 25 (9) |
| Medium | 375 (65) | 186 (63) | 189 (66) |
| High | 160 (27) | 88 (30) | 72 (25) |
| Household annual income[c] | | | |
| Low | 54 (9) | 27 (9) | 27 (9) |
| Medium | 304 (53) | 151 (51) | 153 (54) |
| High | 222 (38) | 116 (40) | 106 (37) |
| Pregnancy | | | |
| Smoking | 46 (8) | 20 (7) | 26 (9) |
| Cat or dog in the home during pregnancy | 202 (35) | 92 (31) | 110 (38) |
| Antibiotic usage during pregnancy | 205 (35) | 103 (35) | 102 (37) |
| Participation in the long-chain n-3 PUFA RCT | 581 (100) | 295 (51) | 286 (49) |
| Serum vitamin D level, mean (SD), ng/mL | 31 (10) | 31 (10) | 31 (10) |
| **Children** | | | |
| Male | 298 (51) | 155 (53) | 143 (50) |
| Age at unblinding, mean (SD), y | 4.1 (0.5) | 4.1 (0.5) | 4.1 (0.5) |
| White | 555 (96) | 283 (96) | 272 (95) |
| Season of birth | | | |
| Winter | 209 (36) | 106 (36) | 103 (36) |
| Spring | 106 (18) | 53 (18) | 53 (18) |
| Summer | 120 (21) | 63 (21) | 57 (20) |
| Fall | 146 (25) | 73 (25) | 73 (26) |
| **Births** | | | |
| Term birth >37 wk | 561 (97) | 284 (96) | 277 (97) |
| Primiparity | 263 (45) | 122 (41) | 141 (49) |
| Intrapartum antibiotics | 183 (32) | 97 (33) | 86 (30) |
| Antibiotics to the neonate | 14 (2) | 5 (2) | 9 (3) |
| Apgar score at 5 min <10 | 26 (5) | 14 (5) | 12 (4) |
| Neonate hospitalized after birth | 56 (10) | 29 (10) | 27 (9) |
| Section | | | |
| Cesarean | 128 (22) | 69 (23) | 59 (20) |
| Emergency | 71 (12) | 39 (13) | 32 (11) |
| Elective | 57 (10) | 30 (10) | 27 (9) |

Abbreviations: COPSAC, Copenhagen Prospective Studies on Asthma in Childhood; PUFA, polyunsaturated fatty acids; RCT, randomized clinical trial.

SI Conversion: to convert vitamin D to nmol/L, multiply by 2.496.

[a] History of physician-diagnosed asthma.

[b] Low (primary school, secondary school, or college graduate), medium (tradesman or bachelor's degree), high (master's degree).

[c] Low (below €50 000), medium (€50 000-€110 000), high (above €110 000); €1 = $1.07.

## Statistical Analysis

The effect of vitamin $D_3$ supplementation on the primary end point, age at onset of persistent wheeze, as well as lower respiratory infections and eczema, was analyzed by Cox proportional hazards regression, for which *P* values correspond to Wald tests. The children were retained in the model from birth until age of diagnosis, drop out, or age at their last clinic visit before the RCT was unblinded.

The effect of vitamin $D_3$ supplementation on the cross-sectional end points of asthma and allergic sensitization was analyzed by logistic regression, whereas the effect on number of episodes of troublesome lung symptoms and upper respiratory tract infections was analyzed by a generalized estimating equation Poisson regression model taking account of repeated participant measurements.

The effect on airway immunology in the vitamin $D_3$ group vs control group was analyzed and visualized by a principal component analysis (PCA)[29] capturing the overall immunological trends in the data and their relation to the intervention analyzed by Wilcoxon rank sum test. Initially, the mediator levels were log-transformed. Prior to the PCA the variables were scaled to unit variance.



jamanetwork/2016/jama/26jan2016/joi150163 PAGE: left 4 SESS: 32 OUTPUT: Jan 11 17:52 2016

The primary analysis of age at onset of persistent wheeze was an intention-to-treat analysis, which is presented crude and adjusted for sex, birth season, maternal vitamin D level at randomization, and participation in the PUFA RCT. A 2-sided *P* value of .05 for significance was used in all types of analyses, which were conducted using SAS (SAS Institute), version 9.3, and MATLAB R2014a (Natick). No imputation was performed for missing data.

Additional methodological details are outlined in Supplement 2 and the $COPSAC_{2010}$ design paper.[13]

## Results

Of 1876 pregnant Danish women screened for eligibility to the $COPSAC_{2010}$ mother-child cohort, 1138 were not included or declined to participate. We randomized 623 of the 738 eligible women from March 4, 2009, to November 17, 2010, as the ethical approval of the vitamin D trial was delayed during enrollment of the first 115 eligible women into the $COPSAC_{2010}$ cohort. In addition, 43 women were withdrawn before childbirth. We unblinded 8 randomizations during pregnancy (including 4 intrauterine deaths) and 3 children were excluded due to chronic disorders, leaving 581 children for the primary analysis (**Figure 1**). The clinical follow-up rate of the children was 94% at age 3 years.

At randomization, 52% of the women had sufficient vitamin D (25-hydroxyvitamin D) levels (>30 ng/mL; to convert to nmol/L, multiply by 2.496), 34% had insufficient levels (20-30 ng/mL) and 14% had deficient levels (<20 ng/mL). Baseline characteristics of the participating mother-child pairs are outlined in **Table 1** showing no clinically important differences in maternal serum vitamin D levels at randomization (mean [SD] level: 31 ng/mL [10] for the vitamin $D_3$ group vs 31 ng/mL [10] for the control group) or season of birth (eg, winter birth: 36% for the vitamin $D_3$ group vs 36% for the control group).

Adherence to the intervention, defined as mothers taking more than 80% of the prescribed tablets, was 74%. The intervention resulted in a significant increase in maternal serum vitamin D level in the treatment group (mean [SD] at randomization vs postpartum: vitamin $D_3$ group, 31 ng/mL [10] at randomization vs 43 ng/mL [14] postpartum; control group, 31 ng/mL [10] at randomization vs 29 ng/mL [13] postpartum; mean between-group difference, 13 ng/mL [95% CI, 11-16], *P* < .001) (eFigure in Supplement 2). Correspondingly, the percentage of women with sufficient levels of vitamin D (>30 ng/mL) after the intervention was 81% in the vitamin $D_3$ group compared with 44% in the control group (mean difference, 37% [95% CI, 30%-45%], *P* < .001).

### Vitamin $D_3$ Supplementation and Risk of Persistent Wheeze

During the first 3 years of life, persistent wheeze was diagnosed in 104 (18%) of the 581 children, with 47 affected children (16%) in the vitamin $D_3$ group vs 57 affected children (20%) in the control group. The intention-to-treat analysis of age at onset of persistent wheeze did not show a significant effect on risk of persistent wheeze from vitamin $D_3$ supplementation during pregnancy (hazard ratio [HR], 0.76 [95% CI, 0.52-1.12], *P* = .16) (**Figure 2**).

Figure 2. Effect of Vitamin $D_3$ Supplementation on Risk of Persistent Wheeze in Children in the $COPSAC_{2010}$ Vitamin D RCT

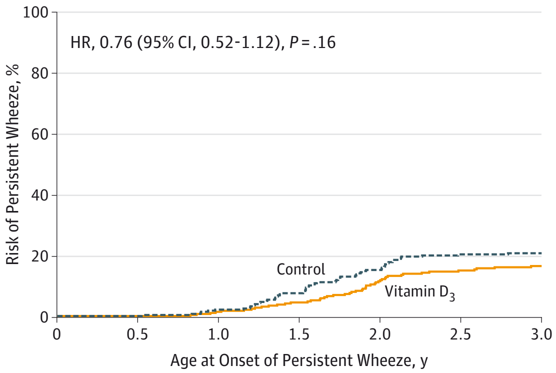


| No. at risk | | | | |
|---|---|---|---|---|
| Vitamin $D_3$ | 295 | 287 | 253 | 198 |
| Control | 286 | 275 | 231 | 184 |

COPSAC indicates Copenhagen Prospective Studies on Asthma in Childhood; HR, hazard ratio; RCT, randomized clinical trial. *P* values were evaluated using the Wald test.

Sex, season of birth, maternal vitamin $D_3$ level at randomization, and the long-chain n-3 PUFA RCT did not interact with the supplementation effect (*P* > .05 for all interaction analyses). Adjusting the primary analysis for these variables did not modify the results (HR, 0.75 [95% CI, 0.51-1.10], *P* = .14) (**Table 2**). An analysis of the full 3 to 5 years follow-up at the time of unblinding of the study showed similar results (HR, 0.78 [95% CI, 0.54-1.13], *P* = .20).

A post hoc analysis of the effect of vitamin $D_3$ levels after the intervention showed a reduced risk of persistent wheeze per 4 ng/mL increase in maternal serum vitamin D level after intervention (HR, 0.94 [95% CI, 0.89-0.99], *P* = .03). Accordingly, the risk of persistent wheeze was increased in children born to mothers with postinterventional vitamin $D_3$ levels in the lowest vs middle and upper tertiles (**Figure 3**). These findings are consistent with the effect estimate for persistent wheeze of 0.76 (approximately 20% reduced risk) associated with the intervention, which on average resulted in a 13 ng/mL higher level of vitamin D after the intervention. Adjusting the analysis for sex, season of birth, maternal smoking during pregnancy, and vitamin $D_3$ level at randomization did not modify the result (HR, 0.93 [95% CI, 0.88-0.99], *P* = .02).

### Vitamin $D_3$ Supplementation and Secondary End Points

#### Episodes of Troublesome Lung Symptoms

Vitamin $D_3$ supplementation during pregnancy resulted in significantly fewer episodes of troublesome lung symptoms during the first 3 years of life in the vitamin $D_3$ group vs the control group (mean episodes, 5.9 in the vitamin $D_3$ group vs 7.2 in the control group; incidence risk ratio [IRR], 0.83 [95% CI, 0.71-0.97], *P* = .02 (Table 2).



jamanetwork/2016/jama/26jan2016/joi150163 PAGE: right 5 SESS: 32 OUTPUT: Jan 11 17:52 2016

Table 2. Primary and Secondary End Point Analyses in the $COPSAC_{2010}$ Vitamin D RCT

| | Vitamin $D_3$ Group | | Control Group | | Difference, % (95% CI) | Estimate (95% CI) | P Value |
|---|---|---|---|---|---|---|---|
| | Total, No. | Cases, No. (%) | Total, No. | Cases, No. (%) | | | |
| **Primary End Point** | | | | | | | |
| Persistent wheeze, 0-3 y | 295 | 47 (16) | 286 | 57 (20) | 4 (−2 to 10) | HR, 0.76 (0.52 to 1.12) | .16 |
| Persistent wheeze, 0-3 y, adjusted[a] | 292 | 47 (16) | 285 | 57 (20) | 4 (−2 to 10) | aHR, 0.75 (0.51 to 1.10) | .14 |
| **Secondary End Points** | | | | | | | |
| Episodes of troublesome lung symptoms, 0-3 y[b] | 287 | 5.9 (5.2 to 6.6) | 278 | 7.2 (6.4 to 8.1) | 1.3 (0.2 to 2.4) | IRR, 0.83 (0.71 to 0.97) | .02 |
| Asthma, 3 y | 278 | 32 (12) | 271 | 47 (14) | 2 (−3 to 8) | OR, 0.82 (0.50 to 1.36) | .45 |
| Upper respiratory tract infections, 0-3 y[c] | 295 | 5.2 (4.8 to 5.5) | 284 | 5.3 (4.9 to 5.6) | 0.1 (−0.6 to 0.4) | IRR, 0.99 (0.90 to 1.09) | .84 |
| Lower respiratory tract infections, 0-3 y | 292 | 94 (32) | 284 | 95 (33) | 1 (−1 to 6) | HR, 0.96 (0.72 to 1.27) | .76 |
| Eczema, 0-3 y | 295 | 68 (23) | 286 | 72 (25) | 2 (−5 to 9) | HR, 0.90 (0.65 to 1.26) | .55 |
| **Allergic Sensitization, 0-3 y[d]** | | | | | | | |
| Skin prick test | 294 | 24 (8) | 283 | 19 (7) | −1 (−6 to 3) | OR, 1.24 (0.66 to 2.31) | .51 |
| Specific IgE | 289 | 34 (12) | 278 | 22 (8) | −4 (−9 to 2) | OR, 1.55 (0.89 to 2.73) | .13 |

Abbreviations: aHR, adjusted hazard ratio; COPSAC indicates Copenhagen Prospective Studies on Asthma in Childhood; HR, hazard ratio; IgE, immunoglobulin E; IRR, incidence risk ratio; OR, odds ratio; RCT, randomized clinical trial.

[a] Adjusted for sex, birth season, the long-chain n-3 polyunsaturated fatty acids randomized clinical trial, and maternal vitamin D at randomization.

[b] Reported as mean No. of episodes (95% CI) at age 0 to 3 y.

[c] Reported as mean No. of episodes per year (95% CI) at age 0 to 3 y.

[d] Positive result for allergic sensitization at age 6 months or 18 months against dogs, cats, milk, or eggs.

Figure 3. Effect of Maternal Serum Vitamin $D_3$ Level at 1 Week Postpartum (n = 573) on Risk of Persistent Wheeze in Children in the $COPSAC_{2010}$ Vitamin D RCT

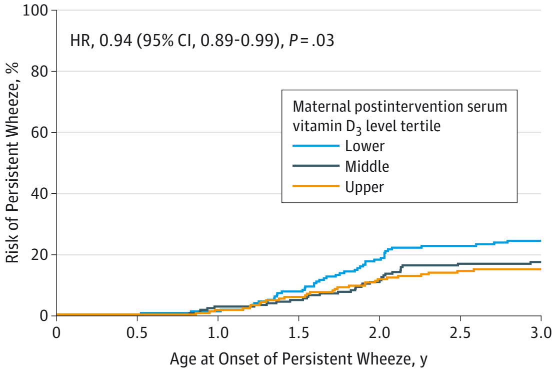


No. at risk by maternal serum vitamin $D_3$ level tertile

| | | | | |
|---|---|---|---|---|
| Lower | 190 | 182 | 147 | 108 |
| Middle | 191 | 184 | 165 | 129 |
| Upper | 192 | 189 | 165 | 141 |

COPSAC indicates Copenhagen Prospective Studies on Asthma in Childhood; HR, hazard ratio; RCT, randomized clinical trial. To convert vitamin D to nmol/L, multiply by 2.496. The HR describes risk of persistent wheeze per 4 ng/mL increase in maternal serum vitamin D level after intervention. Mean (range) of tertiles: lower tertile, 20 ng/mL (6-29); middle tertile, 35 ng/mL (29-41); upper tertile, 53 ng/mL (42-103). *P* values were evaluated using the Wald test.

### Asthma

Asthma at age 3 years was diagnosed in 69 children (13%) of 549, with 32 children (12%) in the vitamin $D_3$ group and 47 children (14%) in the control group (odds ratio [OR], 0.82 [95% CI, 0.50-1.36], *P* = .45).

### Airway Immunology

The purely data-driven PCA showed a uniform up-regulated mediator pattern in principal component 1, which explained 54% of the variation in the data and significantly separated children in the intervention groups (*P* = .04) (**Figure 4** and eTable 1 in Supplement 2).

### Systemic Low-Grade Inflammation

Children in the vitamin $D_3$ group vs control group did not show a significant difference in levels of high-sensitivity C-reactive protein at age 6 months (median [interquartile range], 1.10 mg/L [0.56-4.23] vs 1.45 mg/L [0.51-4.90], *P* = .09). There were also no significant differences in levels of IL-6, TNF-α, or CXCL8 between children in the vitamin $D_3$ group vs control group (eTable 2 in Supplement 2).

### Respiratory Tract Infections

Vitamin $D_3$ supplementation did not modify the number of upper respiratory tract infections (5.2/y in the vitamin $D_3$ group vs 5.3/y in the control group; IRR, 0.99 [95% CI, 0.90-1.09], *P* = .84) or lower respiratory tract infections at age 0 to 3 years (94 children [32%] in the vitamin $D_3$ group vs 95 children [33%] in the control group; HR, 0.96 [95% CI, 0.72-1.27], *P* = .76).

### Allergic Sensitization

The risk of allergic sensitization was not significantly affected by the vitamin $D_3$ supplement assessed by either skin prick test (OR, 1.24 [95% CI, 0.66-2.31], *P* = .51) or specific IgE level (OR, 1.55 [95% CI, 0.89-2.73], *P* = .13).



jamanetwork/2016/jama/26jan2016/joi150163 PAGE: left 6 SESS: 32 OUTPUT: Jan 11 17:52 2016

Figure 4. Effect of Vitamin $D_3$ Supplementation on the Neonatal Airway Immune Profile in the $COPSAC_{2010}$ Vitamin D RCT

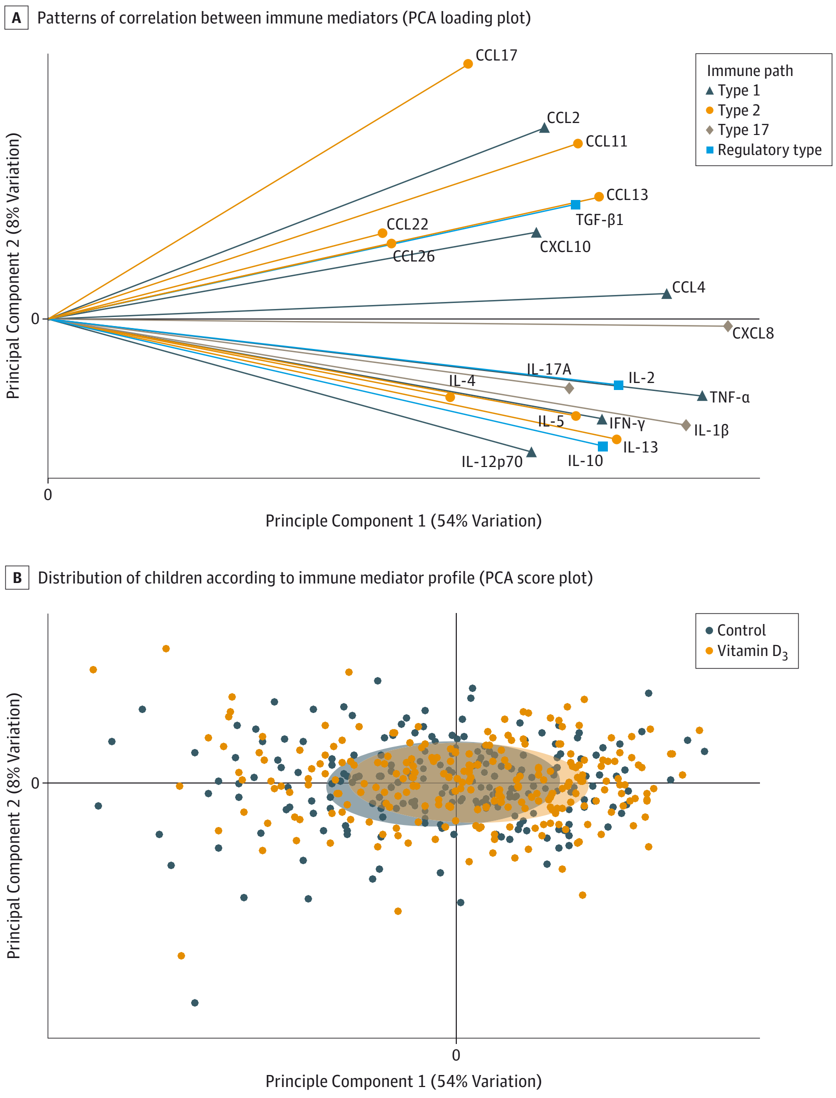


COPSAC indicates Copenhagen Prospective Studies on Asthma in Childhood; IFN, interferon; IL, interleukin; PCA, principal component analysis; RCT, randomized clinical trial; TGF, transforming growth factor; TNF, tumor necrosis factor. Panel A, 0/0 is the base for interpreting how well a particular mediator is explained. Increasing length of the arrows from 0/0 corresponds to an increased proportion of the mediator being explained by the model. Principal component 1 reflects the overall immune activity regardless of individual mediators, accounting for 54% of the variation in data. Principal component 2 reflects a pattern related to pertubation of CCL17, CCL2, CCL11, CCL13, and TGF-1β vs IL-12p70, IL-10, IL-13, IL-5, IFN-γ, IL-1β, TNF-α, IL-4, IL-2, and IL-17α, accounting for additionally 8% of the variation. Thus, children with loading values above 0 on principal component 2 have an immune profile skewed toward CCL17, CCL2, CCL11, CCL13, and TGF-1β, whereas children with loading values below 0 have an immune profile skewed toward IL-12p70, IL-10, IL-13, IL-5, IFN-γ, IL-1β, TNF-α, IL-4, IL-2, and IL-17α. Panel B, each point corresponds to a child, and 2 points closely positioned indicate a similar profile. The ellipses reflect the 2-dimensional distribution of children in the vitamin $D_3$ group (orange) vs control group (blue), for which 0/0 is the multivariate average of the distribution. Principal component 1 reflects a significant discrepancy between the groups (*P* = .04), pointing at generally higher airway immune activity in children from mothers supplemented with vitamin $D_3$ during pregnancy.

### Eczema

Eczema development was unaffected by the intervention (68 [23%] for the vitamin $D_3$ group vs 72 [25%] for the control group; HR, 0.90 [95% CI, 0.65-1.26], *P* = .55).

### Safety

Intrauterine death was observed in 1 fetus (<1%) in the vitamin $D_3$ group vs 3 fetuses (1%) in the control group, any congenital malformations in 17 neonates (5%) in the vitamin $D_3$ group vs 23 neonates (8%) in the control group, and child hospitalization after birth in 32 children (11%) in the vitamin $D_3$ group vs 28 children (10%) in the control group (eTable 3 in Supplement 2).

## Discussion

Maternal supplementation with 2800 IU/d vs 400 IU/d of vitamin $D_3$ during the third trimester of pregnancy did not result in a reduced risk of persistent wheeze through age 3 years in the offspring. However, a clinically important protective effect cannot be excluded as the lower limit of the HR CI was 0.52. The possibility of a protective effect is further supported by secondary end point analyses showing a significant reduction in number of episodes of troublesome lung symptoms, an up-regulated neonatal airway immune profile. However, the development of asthma, upper and lower respiratory tract infections, allergic sensitization, and eczema was unaffected by the vitamin $D_3$ supplementation.

The main limitation of the study is a reduced statistical power to detect an effect on the primary end point of persistent wheeze. In addition, the vitamin $D_3$ supplementation dose may have been too low[30,31] as suggested by the significant decreased risk of persistent wheeze per increase in maternal serum vitamin D level at cessation of the trial (Figure 3). The normal level of vitamin D in a mother during pregnancy for optimalimmune and lung development is unknown and might be as high as 40-60 ng/mL.



jamanetwork/2016/jama/26jan2016/joi150163 PAGE: right 7 SESS: 32 OUTPUT: Jan 11 17:52 2016

Also, we may have begun supplementation too late, while only 81% of the women had serum vitamin $D_3$ above 30 ng/mL after the intervention. Initiating vitamin $D_3$ supplementation at earlier pregnancy stages may be beneficial, as recent data in humans suggest that vitamin D affects fetal lung development as early as the start of the second trimester.[32] However, our data suggesting no effect of early (pre-intervention) levels of vitamin D argue against this hypothesis. Finally, the study did not include postnatal supplementation of the children, which could have induced an additive effect if the effects of maternal supplementation declined postnatally.

The primary strength of the study is the single-center design with standardized diagnoses performed solely by the experienced COPSAC research pediatricians. Other multicenter studies have used various clinicians with different training and experience, which may introduce diagnostic heterogeneity. The longitudinal clinical assessments at both scheduled and unscheduled visits to the research center accompanied by daily diary recordings of troublesome lung symptoms to capture disease burden between visits provided a highly specific primary end point with strong data on age at onset, which is a major advantage compared with studies using retrospective or cross-sectional unspecific community-based diagnoses.[7-9] This approach is important as diagnosis of wheezy disorders in young children is heterogeneous outside research settings due to lack of objective tests and nonstandardized diagnostic procedures.[2] Our close clinical surveillance resulted in a high follow-up rate of the children during the 3-year double-blinded period with more than 94% completing the year 3 visit.

Vitamin D levels are associated with lifestyle factors such as diet, sun exposure, physical activity, and tobacco smoke exposure, which confer a risk of residual confounding in observational studies.[33] Our placebo-controlled study design with unbiased randomization mitigated such confounding and allowed us to examine the isolated effect of vitamin $D_3$ supplementation. In addition, the mothers had good adherence to the intervention with 74% taking more than 80% of the intervention tablets with no differences between intervention groups.

Vitamin D deficiency may lead to wheezy disorders by interfering with fetal lung cell maturation during pregnancy and subsequent lung function development.[34] An alternative mechanism founded on alterations of the airway microbiome by induction of the antimicrobial cathelicidin in bronchial epithelial cells has also been proposed.[35] It is a common belief that vitamin D possesses a range of immune regulatory properties, which are important for immune constitution in early life.[5] Our finding of a significantly up-regulated airway immune profile at age 1 month in the vitamin $D_3$ supplemented group supports this hypothesis and the interrelationship between the relative up-regulations of immune mediators related to type 1, type 2, type 17, and regulatory immune paths may protect against development of wheeze.

Vitamin D immune-modulatory mechanisms have been suggested to increase the frequency of respiratory tract infections,[12] leading to virus-induced wheezing.[36] Such a pathway is not supported by our results showing no effect of vitamin $D_3$ supplementation on either upper or lower respiratory tract infections.

Effective preventive strategies to alleviate the large burden of childhood wheezing and related disorders represent a major unmet clinical need. This RCT of vitamin $D_3$ supplementation during pregnancy did not show a statistically significant effect on the primary end point of persistent wheeze, although a clinically important protective effect cannot be excluded, and a protective effect is suggested by the observed effect on airway immunology and symptomatic episodes. Therefore, further studies with a larger sample size, higher dose, and potentially earlier intervention during pregnancy and postnatal supplementation should be performed to establish the potential benefits of vitamin $D_3$ supplementation to pregnant women to reduce occurrence of wheezy disorders in the offspring.

## Conclusions

The use of 2800 IU/d of vitamin $D_3$ during the third trimester of pregnancy compared with 400 IU/d did not result in a statistically significant reduced risk of persistent wheeze in the offspring through age 3 years. However, interpretation of the study is limited by a wide CI that includes a clinically important protective effect.

### ARTICLE INFORMATION

**Author Affiliations:** Copenhagen Prospective Studies on Asthma in Childhood, Herlev and Gentofte Hospital, University of Copenhagen, Copenhagen, Denmark (Chawes, Bønnelykke, Stokholm, Vissing, Bjarnadóttir, Schoos, Wolsk, Pedersen, Vinding, Thorsteinsdóttir, Arianto, Hallas, Rasmussen, Bisgaard); Department of Pediatrics, Naestved Hospital, Naestved, Denmark (Stokholm, Bjarnadóttir, Pedersen, Vinding); Department of Clinical Biochemistry, Aarhus University Hospital, Aarhus, Denmark. (Heickendorff); Center for Biological Sequence Analysis, Department of Systems Biology, Technical University of Denmark, Lyngby, Denmark (Brix); Department of Food Science, Faculty of Science, University of Copenhagen, Copenhagen, Denmark (Rasmussen).

**Author Contributions:** Dr Bisgaard had full access to all of the data in the study and takes responsibility for the integrity of the data and the accuracy of the data analysis. Drs Chawes and Bønnelykke contributed equally to the manuscript.
*Study concept and design:* Chawes, Bønnelykke, Stokholm, Bisgaard.
*Acquisition, analysis, or interpretation of data:* All authors.
*Drafting of the manuscript:* Chawes, Bønnelykke, Stokholm, Bisgaard.
*Critical revision of the manuscript for important intellectual content:* All authors.
*Statistical analysis:* Chawes, Stokholm, Rasmussen.
*Obtained funding:* Chawes, Bønnelykke, Stokholm, Vissing, Bisgaard.
*Administrative, technical, or material support:* Chawes, Bønnelykke, Stokholm, Heickendorff, Brix, Bisgaard.
*Study supervision:* Chawes, Bønnelykke, Stokholm, Bisgaard.

**Conflict of Interest Disclosures:** All authors have completed and submitted the ICMJE Form for Disclosure of Potential Conflicts of Interest. Dr Bisgaard reports receiving consulting fees from Chiesi. No other disclosures were reported.

**Funding/Support:** The Copenhagen Prospective Study on Asthma in Childhood (COPSAC) is funded by private and public research funds that are all listed on http://www.copsac.com. The Lundbeck Foundation, Danish State Budget, Danish Council for Strategic Research, Danish Council for Independent Research, and the Capital Region Research Foundation have provided core support for COPSAC.

**Role of the Funder/Sponsor:** The funders had no role in the design and conduct of the study; collection, management, analysis, and interpretation of the data; preparation, review, or



jamanetwork/2016/jama/26jan2016/joi150163 PAGE: left 8 SESS: 32 OUTPUT: Jan 11 17:52 2016

approval of the manuscript; and decision to submit the manuscript for publication.

**Additional Information:** The COPSAC biobank is publicly available at the Danish National Biobank (http://www.biobankdenmark.dk) and data will become available in the Danish Data Archive (https://www.sa.dk/).

**Additional Contributions:** We thank the children and families of the $COPSAC_{2010}$ cohort study for all their support and commitment. We also thank the COPSAC research team for their efforts.

## REFERENCES

**1**. Bisgaard H, Szefler S. Prevalence of asthma-like symptoms in young children. *Pediatr Pulmonol*. 2007;42(8):723-728.

**2**. Eder W, Ege MJ, von Mutius E. The asthma epidemic. *N Engl J Med*. 2006;355(21):2226-2235.

**3**. Holick MF. Vitamin D deficiency. *N Engl J Med*. 2007;357(3):266-281.

**4**. Litonjua AA, Weiss ST. Is vitamin D deficiency to blame for the asthma epidemic? *J Allergy Clin Immunol*. 2007;120(5):1031-1035.

**5**. Litonjua AA. Childhood asthma may be a consequence of vitamin D deficiency. *Curr Opin Allergy Clin Immunol*. 2009;9(3):202-207.

**6**. Chawes BL, Bønnelykke K, Jensen PF, Schoos A-MM, Heickendorff L, Bisgaard H. Cord blood 25(OH)-vitamin D deficiency and childhood asthma, allergy and eczema: the $COPSAC_{2000}$ Birth Cohort Study. *PLoS One*. 2014;9(6):e99856.

**7**. Camargo CA Jr, Rifas-Shiman SL, Litonjua AA, et al. Maternal intake of vitamin D during pregnancy and risk of recurrent wheeze in children at 3 y of age. *Am J Clin Nutr*. 2007;85(3):788-795.

**8**. Devereux G, Litonjua AA, Turner SW, et al. Maternal vitamin D intake during pregnancy and early childhood wheezing. *Am J Clin Nutr*. 2007;85 (3):853-859.

**9**. Baïz N, Dargent-Molina P, Wark JD, Souberbielle J-C, Annesi-Maesano I; EDEN Mother-Child Cohort Study Group. Cord serum 25-hydroxyvitamin D and risk of early childhood transient wheezing and atopic dermatitis. *J Allergy Clin Immunol*. 2014;133 (1):147-153.

**10**. Camargo CA Jr, Ingham T, Wickens K, et al; New Zealand Asthma and Allergy Cohort Study Group. Cord-blood 25-hydroxyvitamin D levels and risk of respiratory infection, wheezing, and asthma. *Pediatrics*. 2011;127(1):e180-e187.

**11**. Jones AP, Palmer D, Zhang G, Prescott SL. Cord blood 25-hydroxyvitamin $D_3$ and allergic disease during infancy. *Pediatrics*. 2012;130(5): e1128-e1135.

**12**. Morales E, Romieu I, Guerra S, et al; INMA Project. Maternal vitamin D status in pregnancy and risk of lower respiratory tract infections, wheezing, and asthma in offspring. *Epidemiology*. 2012;23(1):64-71.

**13**. Bisgaard H, Vissing NH, Carson CG, et al. Deep phenotyping of the unselected $COPSAC_{2010}$ Birth Cohort Study. *Clin Exp Allergy*. 2013;43(12): 1384-1394.

**14**. Schulz KF, Altman DG, Moher D; CONSORT Group. CONSORT 2010 statement: updated guidelines for reporting parallel group randomised trials. *BMJ*. 2010;340:c332.

**15**. Højskov CS, Heickendorff L, Møller HJ. High-throughput liquid-liquid extraction and LCMSMS assay for determination of circulating 25(OH) vitamin $D_3$ and $D_2$ in the routine clinical laboratory. *Clin Chim Acta*. 2010;411(1-2):114-116.

**16**. Maunsell Z, Wright DJ, Rainbow SJ. Routine isotope-dilution liquid chromatography-tandem mass spectrometry assay for simultaneous measurement of the 25-hydroxy metabolites of vitamins $D_2$ and $D_3$. *Clin Chem*. 2005;51(9):1683-1690.

**17**. Bisgaard H, Hermansen MN, Loland L, Halkjaer LB, Buchvald F. Intermittent inhaled corticosteroids in infants with episodic wheezing. *N Engl J Med*. 2006;354(19):1998-2005.

**18**. Bisgaard H, Bønnelykke K, Sleiman PMA, et al. Chromosome 17q21 gene variants are associated with asthma and exacerbations but not atopy in early childhood. *Am J Respir Crit Care Med*. 2009; 179(3):179-185.

**19**. Bisgaard H, Hermansen MN, Bønnelykke K, et al. Association of bacteria and viruses with wheezy episodes in young children: prospective birth cohort study. *BMJ*. 2010;341:c4978.

**20**. Vissing NH, Chawes BLK, Bisgaard H. Increased risk of pneumonia and bronchiolitis after bacterial colonization of the airways as neonates. *Am J Respir Crit Care Med*. 2013;188(10):1246-1252.

**21**. Chawes BLK, Poorisrisak P, Johnston SL, Bisgaard H. Neonatal bronchial hyperresponsiveness precedes acute severe viral bronchiolitis in infants. *J Allergy Clin Immunol*. 2012;130(2):354-361.e3.

**22**. Chawes BLK, Edwards MJ, Shamji B, et al. A novel method for assessing unchallenged levels of mediators in nasal epithelial lining fluid. *J Allergy Clin Immunol*. 2010;125(6):1387-1389.e3.

**23**. Følsgaard NV, Chawes BL, Rasmussen MA, et al. Neonatal cytokine profile in the airway mucosal lining fluid is skewed by maternal atopy. *Am J Respir Crit Care Med*. 2012;185(3):275-280.

**24**. Følsgaard NV, Schjørring S, Chawes BL, et al. Pathogenic bacteria colonizing the airways in asymptomatic neonates stimulates topical inflammatory mediator release. *Am J Respir Crit Care Med*. 2013;187(6):589-595.

**25**. Chawes BLK, Stokholm J, Bønnelykke K, Brix S, Bisgaard H. Neonates with reduced neonatal lung function have systemic low-grade inflammation. *J Allergy Clin Immunol*. 2015;135(6):1450-1456.e1.

**26**. Hanifin J, Rajka G. Diagnostic features of atopic dermatitis. *Acta Derm Venereol*. 1980;92:44-47.

**27**. Bisgaard H, Halkjaer LB, Hinge R, et al. Risk analysis of early childhood eczema. *J Allergy Clin Immunol*. 2009;123(6):1355-1360.e5.

**28**. Halkjaer LB, Loland L, Buchvald FF, et al. Development of atopic dermatitis during the first 3 years of life: the Copenhagen Prospective Study on Asthma in Childhood cohort study in high-risk children. *Arch Dermatol*. 2006;142(5):561-566.

**29**. Rasmussen MA, Colding-Jørgensen M, Hansen LT, Bro R. Multivariate evaluation of pharmacological responses in early clinical trials—a study of rIL-21 in the treatment of patients with metastatic melanoma. *Br J Clin Pharmacol*. 2010;69 (4):379-390.

**30**. Hollis BW, Johnson D, Hulsey TC, Ebeling M, Wagner CL. Vitamin D supplementation during pregnancy: double-blind, randomized clinical trial of safety and effectiveness. *J Bone Miner Res*. 2011; 26(10):2341-2357.

**31**. Litonjua AA, Lange NE, Carey VJ, et al. The Vitamin D Antenatal Asthma Reduction Trial (VDAART): rationale, design, and methods of a randomized, controlled trial of vitamin D supplementation in pregnancy for the primary prevention of asthma and allergies in children. *Contemp Clin Trials*. 2014;38(1):37-50.

**32**. Kho AT, Sharma S, Qiu W, et al. Vitamin D related genes in lung development and asthma pathogenesis. *BMC Med Genomics*. 2013;6:47.

**33**. Theodoratou E, Tzoulaki I, Zgaga L, Ioannidis JPA. Vitamin D and multiple health outcomes: umbrella review of systematic reviews and meta-analyses of observational studies and randomised trials. *BMJ*. 2014;348:g2035.

**34**. Zosky GR, Berry LJ, Elliot JG, James AL, Gorman S, Hart PH. Vitamin D deficiency causes deficits in lung function and alters lung structure. *Am J Respir Crit Care Med*. 2011;183(10):1336-1343.

**35**. Yim S, Dhawan P, Ragunath C, Christakos S, Diamond G. Induction of cathelicidin in normal and CF bronchial epithelial cells by 1,25-dihydroxyvitamin ($D_3$). *J Cyst Fibros*. 2007;6 (6):403-410.

**36**. Hollams EM. Vitamin D and atopy and asthma phenotypes in children. *Curr Opin Allergy Clin Immunol*. 2012;12(3):228-234.



jamanetwork/2016/jama/26jan2016/joi150163 PAGE: right 9 SESS: 32 OUTPUT: Jan 11 17:52 2016
