# Supplementary material for: Prenatal vitamin D supplementation reduces risk of asthma/recurrent wheeze in early childhood: A combined analysis of two randomized controlled trials
Source: PLoS One. 2017 Oct 27;12(10):e0186657. doi: 10.1371/journal.pone.0186657 (PMC5659607; doi:10.1371/journal.pone.0186657)
Supplement: S2 Text — (PDF) [file pone.0186657.s004.pdf]

Koncern Organisation og Personale

De Videnskabsetiske Komiteer

Region Hovedstaden
Regionsgården
Kongens Vænge 2
3400 Hillerød

Opgang H
Telefon 3866 6395
Direkte 38 66 63 23
Mail vek@regionh.dk

Web www.regionh.dk/vek

EAN-nr: 5798001555203
Bank: 31003100142287
CVR/SE-nr: 30113713

Protokol nr.: H-B-2008-093

Ref.: Julie Dahl Larsen

Dato: 22. oktober 2013

Prof. Overlæge, dr. med Hans Bisgaard
Gentofte Hospital
Dansk børneAstma Center/afd. K
Ledreborg Allé 34
2820 Gentofte

Asthma Begins in Childhood - ABC-kohorten. En gen-miljøanalyse af den tidlige udvikling af astma, allergi og eksem.

Sekretariatet for De Videnskabsetiske Komiteer for Region Hovedstaden modtog den 9. september 2013 tillægsprotokol, anm.nr. 39915.

Afgørelse:

Tillægsprotokollen er godkendt i henhold til lov om et videnskabsetisk komitésystem, lov nr. 402 af 28. maj 2003 med senere ændringer.

Godkendelsen omfatter følgende dokumenter:

Protokol, version 3, modtaget pr. mail 8. oktober 2013
Deltagerinformation af 8. oktober 2013, version 1
Samtykkeerklæring af 8. oktober 2013, version 1

Forsøget er samtidig godkendt forlænget til 31. august 2017.

Med venlig hilsen

Julie Dahl Larsen
Juridisk konsulent

Kopi til:

- Jakob Stokholm

De Videnskabsetiske Komiteer
for Region Hovedstaden
Regionsgården
Kongens Vænge 2
3400 Hillerød

Gentofte, den 9.9.2013

Anmeldelse til de Videnskabsetiske Komitéer - Tillægsprotokol

Alle oplysninger på denne blanket kan blive offentliggjort.

Komité

Primærkomité:	De Videnskabsetiske Komiteer for Region Hovedstaden
Projekt-ID:	H-B-2008-093
Anmeldelsesnr.:	39915
Tillægsprotokolnr.:	15

A. Forsøgsansvarlig

1. Titel:	Professor, dr. med
2. Navn:	Hans Bisgaard
3. Hospital/institution:	Gentofte Hospital
4. Afdeling/institut	Dansk børneAstma Center
5. Vejnavn og nr.	Ledreborg Alle 34
6. Postnummer/7. by:	2820 Gentofte
8. Telefonnr.:	3977 7360
9. E-mail:	bisgaard@copsac.com

B. Evt. anden kontaktperson

1. Titel:	MD, PhD
2. Navn:	Jakob Stokholm
3. Hospital/institution:	Gentofte Hospital
4. Afdeling/institut	Dansk BørneAstma Center
5. Vejnavn og nr.	Ledreborg Alle 34
6. Postnummer/7. by:	2820 Gentofte
8. Telefonnr.:	3977 7360
9. E-mail:	jakob.stokholm@dbac.dk

C. Projektinformation

1. Projekttitel:	Asthma Begins in Childhood - ABC-kohorten. En gen-miljøanalyse af den tidlige udvikling af astma, allergi og eksem.
2. Ændring:	Vi ønsker at fortsætte det videnskabelige studie ABC-kohorten. Planen er at fortsætte studiet med 3 opfølgende besøg ved alderen: 4 år, 5 år og 6 år. Formålet med studiet er stadig, at få bedre viden om astmasygdommens udvikling og at finde nye metoder til at forudsige om et barn vil udvikle astma, allergi, høfeber og eksem. Derudover fokuseres tillige på andre faktorer med indflydelse på børnenes generelle sundhed og udvikling. Dette gøres primært med velkendte undersøgelser fra tidligere besøg, samt nye almindeligt anvendte lungefunktionsundersøgelser, som det ikke er muligt at lave før 6 års alderen: (Spirometri med metacholin provokation og FeNO-måling). Derudover ønsker vi at foretage et nasalskrab ved 6 års alderen, som viser hvilke gener der bliver udtrykt i næsens slimhinde.
3. Begrundelse for ændring:	Gennem de 11 planlagte besøg, der allerede har været afholdt fra graviditeten indtil 3 års alderen, har vi indsamlet en stor mængde informationer omkring faktorer, der kan påvirke børnene i det tidligste liv. Meget peger imod, at årsagerne til udviklingen af sygdomme så som astma, allergi og eksem, skal søges i det helt tidlige liv, allerede så tidligt som i graviditeten. Udviklingen skyldes sandsynligvis et komplekst samspil mellem genetik og miljø. Da kun få sygdomme debuterer inden for de 3 første leveår, samtidig med at de astmatiske sygdomme ændrer sig gennem livet, er det utroligt vigtigt, at følge børnenes videre med fortsatte undersøgelser. De udvidede lungefunktionsundersøgelser har alle været anvendt i mange år til at diagnosticere og følge børn og voksne med astma. Ved nasalskrabet måles på mRNA i næsens slimhinden. Dette hjælper os til at forstå, hvilke gener der er aktive under sygdom, og kan kobles med de genetiske undersøgelser der bliver udført på blodprøver.
4. Etiske overvejelser i forbindelse med ændring:	Ingen af de foreslåede undersøgelser er ikke forbundet med nogen former for risici, men der kan forekomme ubehag i form af smerte og en gang imellem lidt næseblod når nasalskrabet udføres. Undersøgelserne med spirometri, metacholin og FeNO, samt nasalskrab er alle ufarlige og foregår under overvågning af klinikkens læger, og vi finder derfor ikke, at der nogle etiske problemstillinger i forbindelse med de nye undersøgelser.
5. Medfører ændringerne at deltagerinformationen ændres ? (Hvis ja, skal den reviderede deltagerinformation medsendes):	Ja
6. Evt. øvrige bemærkninger:	

Underskrift

Professor, dr. med Hans Bisgaard
Gentofte Hospital
Dansk børneAstma Center
Ledreborg Alle 34
2820 Gentofte

Asthma Begins in Childhood - ABC-kohorten

En gen-miljøanalyse af den tidlige udvikling af astma, allergi og eksem

Indhold

Formål 2
Baggrund 2
Asthma Begins in Childhood - ABC-kohorten 4
Samfundsøkonomiske forhold 19
Metode 20
Forsøgsforløb 20
Planlagte besøg 21
Akutte besøg 30
Astma-besøg 30
Forsøgspersonerne 31
Begrundelse for undersøgelse af mindreårige 32
Bivirkninger, risici og ulemper 33
Ulemper og komplikationer for forsøgsdeltagerne 33
Fordele for forsøgspersonerne 36
Etiske aspekter 36
Genetisk materiale 38
ABC studiets organisation 40
Statistisk styrke 40
Publiceringsstrategi 41
Samarbejde i Danmark 41
Økonomisk støtte 42
Fremtidige undersøgelser 42
Bilag 1: Oversigt over undersøgelser 51
Bilag 2. Godkendte VEK-protokoller i COPSAC 53
Bilag 3: Almindelige blodprøve-analyser 55

Formål

Det overordnede formål med denne undersøgelse er at forøge den nuværende viden om udvikling af astma, allergi og atopisk eksem hos børn med henblik på bedre diagnostik, behandling og forebyggende tiltag. Dette gøres ved hjælp af en fødselskohorte med 800 børn, hvor deltagerne følges fra før fødslen og herefter prospektivt med faste besøg samt besøg ved hud- og luftvejssygdom.

Baggrund

Astma, atopisk eksem og allergi – de såkaldt atopiske lidelser - er de hyppigste kroniske sygdomme blandt børn og de almindeligste årsager til hospitalsindlæggelse af børn i den vestlige verden[1].
Omkring 20 % af alle børn udvikler astma-lignende tilstande og 15 % udvikler atopisk eksem.[2]
Sygdommene udvikler sig i et samspil mellem arv (genetik) og miljø. Gennem de sidste 40 år har vi i den vestlige verden været vidne til en markant stigning i forekomsten af astma, allergi og eksem.[3]
Det er endnu kun delvist belyst, hvad denne stigning skyldes, men meget tyder på, at tendensen til atopiske lidelser grundlægges helt tidligt i barndommen. F.eks. debuterer 70-80 % af børn med atopisk eksem i første leveår, hvorfor ansvarlige eksponeringer må findes i det tidlige liv.[4]
Man ved ikke hvilke miljø- og livsstilsfaktorer der er ansvarlige for udviklingen, men der er formentlig tale om et kompliceret samspil mellem forskellige faktorer i miljøet, der på en endnu ukendt måde hænger sammen med vestlig levevis. En række faktorer har været mistænkt: Pre- og perinatale forhold, [5-14]. amning og tidlig ernæring,[15, 16] bakterier og virus der kan udløse akutte lungesymptomer[17], og senere hen astma[18] samt tobakseksponering der både pre- og postnatalt er mistænkt som medvirkende årsag til lungesymptomer [12, 19-23], eksem og allergi.[24]. Men den eksisterende viden om risikofaktorer er overvejende baseret på retrospektive studier og tværsnits-undersøgelser, som ikke er velegnede metoder til at belyse årsagssammenhænge, da de kun kan præsentere et indirekte mål for ekspositionerne. Derfor er der i høj grad brug for at få belyst disse sammenhænge i prospektive undersøgelser, der i videst muligt omfang også tager højde for potentielle confoundere. Viden om risikofaktorer vil kunne forbedre den rådgivning, der gives om adfærd og derved forhåbentlig mindske antallet af nye tilfælde af atopisk sygdom.

COPSAC-kohorten

Nærværende studie tager udgangspunkt i COPSAC-studiet (*Copenhagen Prospective Studies on Asthma in Childhood*), en dansk kohorte på 411 børn fulgt på Dansk Børne Astma Center (DBAC) gennem 7 år med intense beskrivelser af symptomerne på astma, eksem og allergi, samt omfattende analyser af miljø og genetik. Dansk Børne Astma Center er en selvejende institution, som forsker i årsager, diagnose og behandling af astma, allergi og eksem hos børn. Formålet er at skabe evidens for en strategi for primær profylakse (forebyggelse af sygdommens opståen) og en strategi for sekundær profylakse (forebyggelse af udvikling i allerede etableret sygdom). Det er det hidtil mest omfattende studie af årsager til astma, allergi og eksem.

Studiet har påvist flere banebrydende fund indenfor risikofaktorer, genkandidater og nye mål for behandling, og det forventes at der fortsat vil genereres nye resultater de næste 10 år. COPSAC-studiet har desuden bekræftet hypotesen om, at udviklingen af atopiske lidelser grundlægges i den tidlige barndom.
For nylig har COPSAC påvist det hidtil vigtigste gen for udviklingen af eksem og astma (filaggrin) [25] og har endvidere fundet, at dette gens aktivering kan moduleres, hvis man bliver udsat for pelsdyr i de første levemåneder[26, 27]. Der er desuden påbegyndt en altomfattende analyse af genmassen i kohorten, en såkaldt ”Genome-Wide Scan”, og i samarbejde med en række andre udenlandske genetiske laboratorier afsøges COPSAC-kohorten for mistænkte kandidat-gener til de atopiske sygdomme. Det overordnede perspektiv er, at afdækning af mulige kandidat-gener vil bidrage til bedre diagnostik af sygdommene, ligesom en forståelse af hvad, der aktiverer generne kan give bedre videnskabelige holdepunkter for vejledning om bl.a. forebyggende indretning af barnets miljø.

COPSAC har desuden vist, at nogle nyfødte børn huser bakterier i luftvejene og netop disse børn har stærkt øget risiko for at udvikle astma og allergi de følgende år.[28] Det er vores hypotese at de bakterier, der findes i børns luftveje og tarmsystem er det fælles led mellem livsstilsfaktorer og udviklingen af livsstilsygdommene astma, allergi og eksem. Det er derfor oplagt at forske i betydningen af tidlig mikrobiologisk kolonisering og eksposition. Det overordnede perspektiv her er, at vi gennem øget forståelse for sygdommene bedrer mulighederne for forebyggelse og behandling, eksempelvis med probiotika eller antibiotika.

Asthma Begins in Childhood - ABC-kohorten

Som anført har vi i COPSAC genereret vigtige hypoteser omkring udviklingen af atopiske sygdomme, som fortjener at blive undersøgt yderligere. Desuden er der fortsat mange ubesvarede spørgsmål om astma, eksem og allergi, som vi ønsker at se på. På baggrund af den succes vi har haft, såvel videnskabeligt som behandlingsmæssigt for kohortedeltagerne og deres familier, ønsker vi at opstarte en ny fødselskorte, ABC-kohorten *(Asthma begins in Childhood).*
Overordnet vil studiet blive mere fokuseret med færre undersøgelser end i COPSAC-studiet. Til gengæld vil antallet af forsøgsdeltagere være større hvilket giver materialet øget styrke.

Kohortestudiet har følgende delmål:

I. At opstarte en ny prospektiv kohorte, der muliggør løbende indsamling af informationer på en lang række væsentlige områder, miljømæssige og socio-økonomiske, der kan mistænkes for at have indflydelse på udviklingen af atopiske lidelser.

II. At undersøge den mikrobiologiske eksposition hos børn og dennes betydning for udvikling af atopisk sygdom. Undersøgelsen vil inkludere analyser af bakteriel kolonisering af fødselskanalen og luftvejene.

III. At undersøge kæledyrs betydning for børns mikroflora og senere udvikling af atopiske sygdomme.

IV. At undersøge sammenhængen mellem genetik og udvikling af astma, allergi og atopisk eksem, ved hjælp af Genome-Wide Analysis.

V. At udføre en detaljeret opgørelse over forekomsten af almindelige infektioner og andre sygdomme i den tidlige barndom.

VI. At undersøge om indtagelse af fiskeolie kapsler med højt indhold af Omega 3-fedsyrer (n-3 fedtsyrer), i sidste tredjedel af graviditeten, har en gunstig effekt på udviklingen af atopi hos børnene. Sekundært at undersøge om indtagelse af fiskeolie nedsætter risikoen for præterm fødsel.

VII. At undersøge immunforsvarets betydning for den senere udvikling af sygdomme

VIII. Analyse af udåndingsluften hos børn med en ”elektronisk næse”.

IX. Undersøgelse af børns neurologiske udvikling

X. At undersøge lungefunktionen ved helkropsplethysmografi og multiple breath washout

XI. At undersøge børnenes vækst.

XII. Undersøgelse af de øvre luftvejes anatomi.

I. Opstart af ny fødselskohorte

ABC-kohorten udføres i tråd med COPSAC som et klinisk-epidemiologisk studie af astma, atopisk eksem og allergi gennem barndommen med kliniske, cellulære og molekylære analyse under hensyntagen til miljø- og livsstilsfaktorer. Børnene ses i ABC-klinikken hvert halve år, samt ved akutte symptomer fra luftveje og hud. Kliniske data indsamles prospektivt, og der indsamles biologisk materiale til en biobank, som muliggør parakliniske analyser af forløbet forud for og under sygdommenes udvikling. Suppleret med data indsamlet ved COPSAC er ABC-studiet på verdensplan en unik ressource til belysning af årsager til atopi hos børn.

ABC-studiet vil i høj grad drage nytte af erfaringer fra COPSAC-studiet. COPSAC er designet som et overordnet kohortestudie, der screener bredt for en lang række risikofaktorer, kendte og ukendte. Den store bredde kræver mange ressourcer og koster nødvendigvis noget i dybden af de enkelte undersøgelser. ABC-kohorten er en smallere, mere fokuseret undersøgelse, der tager afsæt i positive fund i COPSAC og studerer dem intensivt og dermed langt mere detaljeret end i COPSAC.
Den teknologiske udvikling siden 1998 har på en række områder åbnet muligheder for bedre dataindsamling, særlig indenfor genetisk forskning. Endvidere er den viden og erfaring om opstart og udførsel af en klinisk-epidemiologisk fødselskohorte, som medarbejderstaben på DBAC har tilegnet sig gennem COPSAC, en uvurderlig ressource, der vil lette arbejdet i ABC-studiet betydeligt.

Fra bred screening til fokuseret identifikation:

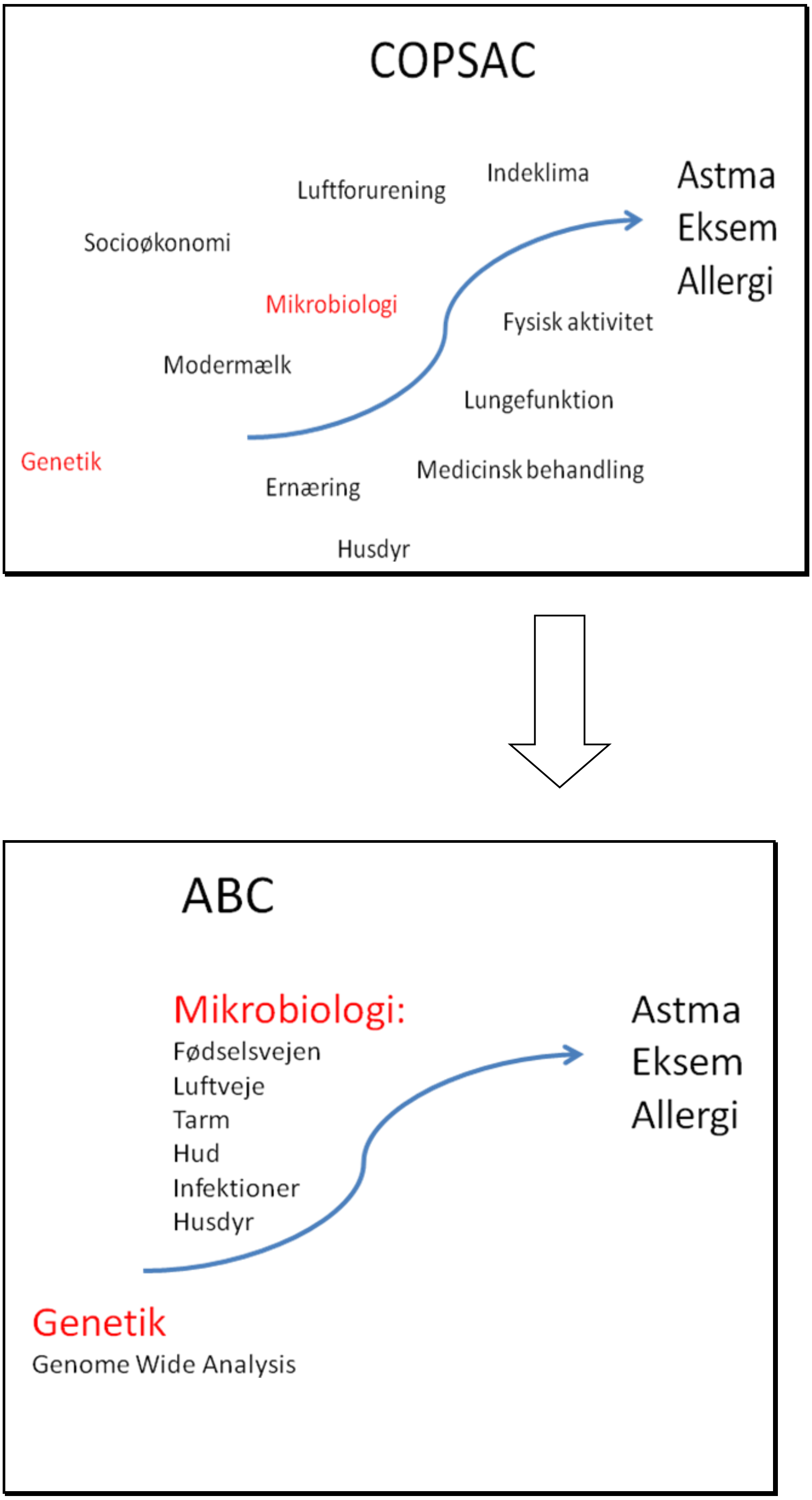

II: Mikrobiologi og immunmodulering

Som anført har vi i COPSAC påvist banebrydende fund i forhold til viden om kolonisering af luftvejene hos småbørn og den senere udvikling af astma. Vi undersøgte mikrofloraen i luftvejene hos 321 børn kort efter fødslen, og sammenholdt det med forekomsten af astma 5 år efter. Studiet viste en 3-4 gange øget risiko for at udvikle astma og tidlig allergi hos de børn, der var koloniseret med bakterierne S.pneumoniae, H. influenzae og/eller M. catarrhalis – bakterier, der betegnes som potentielt sygdomsfremkaldende[28, 29],

Som anført grundlægges tendensen til atopiske lidelser formentligt tidligt i barndommen i forbindelse med modning af immunforsvaret. En udbredt forklaringsmodel er "Hygiejnehypotesen" [30], ifølge hvilken enten utilstrækkelig eksponering i barneårene for diverse mikrober i form af infektioner, eller kontakt med en uhensigtsmæssig sammensætning af mikroorganismer forhindrer stimulation af immunforsvaret. Processen, hvor immunforsvaret ændres fra det medfødte, antistofproducerende Th2 respons til det mere modne og mindre allergigivende cellemedierede Th1 respons[31, 32], bliver således ikke optimal. Ifølge denne hypotese vil en uhensigtsmæssig mikroflora altså forskyde modningen af immunforsvaret mod astma, atopisk dermatit og allergi.

Menneskets slimhinder er koloniseret med mikroorganismer, hovedsageligt bakterier, der ikke medfører sygdom. Fosteret er frit for mikroorganismer, men udsættes for disse allerede under fødslen, hvor barnet under en vaginal fødsel påføres bakterier fra hovedsageligt fødselskanalen men også fra moderens endetarm og hud. Koloniseringen grundlægges således tidligt i livet. Flere forskere har peget på, at koloniseringen med 'sunde' bakterier påvirker immunforsvaret i en retning, der beskytter mod udviklingen af allergiske sygdomme og samtidig nedsætter risikoen for almindelige virus infektions-sygdomme[33].
Betydningen af virusinfektioner tidligt i livet og senere udvikling af astma har tidligere været belyst, og særligt luftvejsvira, f.eks. Respiratorisk Syncytial Virus og Rhinovirus er mistænkt for at kunne påvirke udviklingen af lungerne[34-36]. Det er dog uvist, om det er virus i sig selv, der fører til sygdom, eller om tendensen til at pådrage sig symptomgivende virusinfektioner er et udtryk for sårbare luftveje, muligvis på grund af en anden bakterieflora i luftvejene.

Alt i alt er der fortsat mange ubesvarede spørgsmål om betydningen af mikrobiologi og udviklingen af atopiske lidelser. Derfor ønsker vi at gennemføre en fyldestgørende undersøgelse af ABC-kohortedeltagernes mikrobiologiske kolonisering.

Barnet møder de første bakterier i fødselskanalen, hvorfor vi undersøger den mikrobiologiske kolonisering i skeden hos mødrene kort før fødslen ved hjælp af en podning. Kort efter fødslen indsamler vi slim med eventuelle bakterier og virus fra børnenes luftveje ved sugning, svarende til undersøgelser i COPSAC, og gentager dette 3 gange i barnets første leveår samt ved akut luftvejssygdom. Desuden undersøger vi deres tarmflora ved hjælp af afføringsprøver. Derudover foretages der 2 hudpodninger og en halspodning. Den mikrobiologiske status og eventuelle ændringer kan så senere relateres til udviklingen af atopisk sygdom.

Tarmens bakterielle sammensætning opretholder en velafbalanceret vært-mikrobiel symbiotisk tilstand, og samtidig repræsenterer denne interaktion en kompleks mekanisme, som giver mikrobiomet mulighed for at kommunikere med og modulere det lokale miljø og påvirke barnet imod sygdom. Ved at tage afføringsprøver fra denne store gruppe børn og kortlægge tarmens bakteriesammensætning tidligt i livet samt udviklingen gennem livet, kan vi sammenholde sammensætningen af bakterier med børnenes helbred. Vi vil herved få en unik mulighed for at se, om der er en sammenhæng mellem den tidlige bakteriesammensætning og udvikling af sygdom senere i livet.

III: Betydningen af genetik og husdyr i hjemmet for udviklingen af atopisk eksem hos børn

I COPSAC har vi påvist det hidtil vigtigste gen for udviklingen af eksem og senere hen astma. Vi har dokumenteret at mutationer (genændringer) i genet for filaggrin (FLG) er stærkt associeret til udvikling af atopisk eksem.[25] Filaggrin er et protein, der dannes i det yderste lag af huden (epidermis, stratum granulosum). Det er medvirkende til dannelsen af hudens barriere, der forhindrer væsketab samt indtrængen af infektiøse agens, toksiske kemikalier og ikke mindst allergener.[37] Siden COPSAC dokumenterede sammenhængen mellem disse to mutationer og udviklingen af eksem, har flere grupper bekræftet dette fund [38-40], og associationen mellem filaggrinmutation og udviklingen af eksem fremtræder som den stærkeste kendte genetiske faktor til dato.

Dog er det kun ca. 60 % af de børn, der har genmutationen, der udvikler atopisk eksem, og noget tyder på, at særligt udsættelse for pelsdyr er en afgørende faktor. I COPSAC fandt vi at børn med

FLG-mutationen, er yderligere i væsentlig risiko, hvis de også er eksponeret for kat i de første levemåneder (HR 11.11)[27]. Omvendt nedsættes risikoen, hvis der i stedet er hund i hjemmet[26] (OR 0.44).

Figur: Kaplan-Meier estimat af kumulativ risiko for eksem i løbet af det første leveår, stratificeret for filaggrinmutation og forekomst af kat ved fødsel.

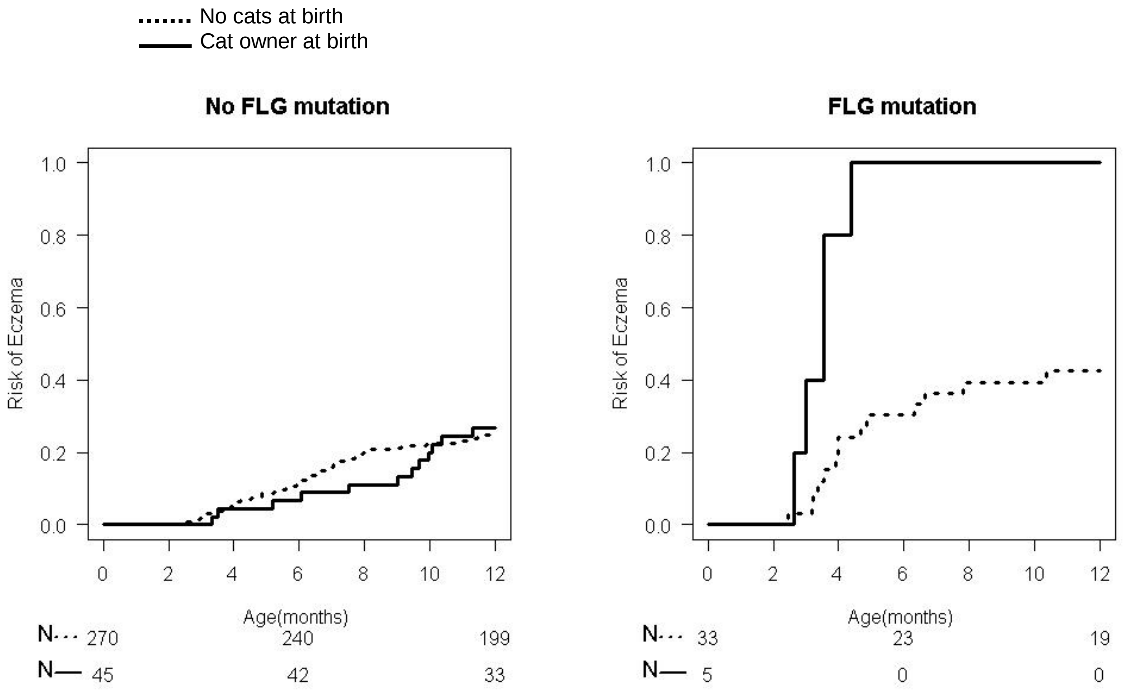

Andre studier kan ikke påvise nogen sammenhæng mellem husdyr og udvikling af atopisk eksem, og nogle finder det modsatte af COPSAC – altså at kat mindsker risikoen mens hund øger den. Ingen af disse studier har dog inddraget FLG, dvs. den genetiske interaktion med miljøet [41, 42].

Man kender endnu ikke årsagen til den eventuelle association mellem pelsdyr, genmutation og udvikling af atopisk eksem. I forlængelse af vores fund om bakteriel flora er vores hypotese, at årsagen til interaktionen mellem husdyr og aktivering af FLG ved udvikling af eksem er, at børnene via dyrene bliver eksponeret for en ændret bakteriel flora.

Sundhedsstyrelsen anbefaler i dag familier med tendens til atopiske sygdomme, at de ikke har pelsdyr hjemme. Denne anbefaling er som det fremgår ikke evidensbaseret. Måske er denne anbefaling blot besværligt for de involverede og til ærgrelse for de familier, der eventuelt føler sig

tvunget til at skaffe sig af med deres husdyr. I værste fald er den forkert og gør mere skade end gavn, idet der risikeres en øgning af genetisk disponerede børns risiko for at få atopisk dermatit. Det er derfor vigtigt, at dette emne bliver bedre undersøgt, så implicerede familier kan rådgives korrekt.

IV: Genetik

Astma, allergi og eksem kan betegnes som en kompleks genetisk sygdom, idet der er multiple genetiske faktorer, der ved interaktion med diverse miljøfaktorer fører til udviklingen af sygdommene.
Da astma, allergi og eksem skyldes et samspil mellem flere forskellige gener og samtidig interaktion med miljøfaktorer, har bestemmelse af de ansvarlige gener været særlig vanskelig. Men gennem det sidste årti har fremskridt inden for diagnostik, molekylærgenetik, statistik og computerteknologi dannet en ny platform til genetisk analyser af komplekse arvelige sygdomme.
Først og fremmest *Genome-Wide Analysis (GWA)*, hvor man identificerer genregioner (loci) med statistisk signifikante forskelle i forekomsten hos hhv. syge og raske. Dette vil tyde på at disse gener spiller en rolle for udviklingen af sygdom og man forsætter dernæst med at identificere relevante gener i disse regioner.
GWA analyserne giver på grund af stor statistisk styrke også mulighed for at analysere gener, der kun fører til en moderat øget risiko for sygdom.

Når genetiske kandidater er identificeret sammenholdes de desuden med indsamlede informationer om miljømæssige ekspositioner og mikrobiologisk kolonisering.
I ABC-kohorten indgår 800 børn med forældre, svarende til 2400 individer. Styrken i dette delstudie er, at GWA analyserne kan suppleres med fundene fra COPSAC, hvorved den samlede GWA-population øges til i alt 3600 (400 COPSAC-børn = 1200 trioer).

Den genetiske forskning udføres i tæt samarbejde med et af USA's førende genetiske laboratorier ved børnehospitalet I Philadelphia (Childrens Hospital of Philadelphia, CHOP). CHOP er en af USA's største medicinske forskningsinstitutioner og råder over det mest avancerede laboratorium til genetiske analyser. Når blodprøver er samlet ind fra alle kohortedeltagere og forældre vil de i anonymiseret form blive sendt til CHOP, der forestår en GWA-scanning. Der vil udelukkende blive set på gener med relation til astma, allergi, eksem og luftvejssymptomer.

Indtil videre kan vi kun analysere genomet overordnet. Vi ved, at det langt fra er alle vores gener, der er aktive, og at mange aktiveres og inaktiveres gennem livet på baggrund af endnu ukendte faktorer. Eksempelvis så vi, at filaggrin-mutationernes betydning for udvikling af eksem øges markant efter udsættelse for kat. Man er ved at udvikle metoder til analyse af, om enkelte gener er aktive eller ej. Det ville rykke forskning i sammenhængen mellem genetik og miljøfaktorer op på et nyt niveau, og kohortestudier som vores vil være den oplagte metode til disse analyser.
GWA er et forskningsfelt i fortsat rivende udvikling, der må forventes at blive endnu mere markant i de nærmeste år. Identifikation af sygdomsgener og kendskab til deres opbygning, regulering og det tilhørende proteins funktion kan muliggøre forbedringer inden for diagnostik, forebyggelse og behandling. Kohortestudiet er velegnet til sådanne studier, idet der er mulighed for løbende undersøgelse i takt med den hastigt udviklende viden på området.
Vi planlægger derudover at udføre nasalskrab, hvorved det er muligt at undersøge, hvilke gener der kommer til udtryk i næsens slimhinde ved at måle på mRNA i slimhinden. Astma, allergi og eksem udløses ved en kompleks interaktion mellem genetik og miljø. På trods af, at sygdommene oftest samles til én enhed, forekommer der stor heterogenitet mellem dem, også inden for hver enkelt sygdom. Måling af genekspression ved mRNA i næsens slimhinde kan forhåbentlig hjælpe os til en større sygdomsforståelse. Således kan det blive muligt at forstå, hvilke gener der er aktive under sygdom, og kan kobles sammen med de overordnede genetiske undersøgelser udført på blodprøverne.

V: Infektioner

Som et delstudie i ABC-kohorten ønsker vi at udføre en detaljeret, prospektiv opgørelse over almindelig sygdom hos småbørn.
Børn sygelighed varierer fra voksnes. Børn er oftere syge, særligt de første leveår. Infektionssygdomme er mest fremtrædende og det formodes at hovedparten skyldes virale infektioner. Selvom disse sygdomme ikke er alvorlige, udgør de hyppige sygdomsepisoder en væsentlig belastning for familierne. På trods af den høje hyppighed er vores faktuelle viden om almindelige børneinfektioner og behandlingen af dem overraskende sparsom.
Der er lavet få undersøgelser af børns almindelige sygelighed. P. Uldalls undersøgelse fra 1981 af 3000 børn er baseret på spørgeskemaer omkring sygelighed gennem den sidste måned[43]. SFI's

Sundheds- og sygelighedsundersøgelser fra 2000 og 2005 baseres ligeledes på spørgeskemaer og behandler børn sygelighed indenfor de sidste 14 dage[44]. Det bliver alene et øjebliksbillede, der ikke fanger årstidsvariationer m.m. Vi har ikke kendskab til undersøgelser, der detaljeret følger børns almindelige sygelighed over længere tid.
Derfor er det i sig selv interessant med en opgørelse over prævalens og incidens af sygdom hos børn, såvel indlæggelseskrævende sygdom som de mindre alvorlige men meget hyppige sygdomsperioder, der i høj grad belaster børnefamilierne.
Derudover er det interessant at se på forskelle i sygelighed i forhold til atopiske sygdomme. Børn med astma har formodentlig flere sygedage end ikke-astmatiske børn. Desuden er der en sammenhæng mellem antallet at infektioner i de første leveår og udvikling af astma og andre atopiske lidelser[45], om end denne sammenhæng ikke er tilstrækkelig belyst.
Opgørelsen vil være en af de bedste af sin art, da al sygdom registreres prospektivt i udleveret dagbog.

Studiets indsamling af informationer om socioøkonomiske variable vil kunne bruges til identifikation af indsatsområder i forhold til at kunne nedbringe almindelig børnesygelighed. F.eks. vil vi kunne se på sammenhængen mellem pasningsforhold, herunder areal, antal børn i institution, m.m. – faktorer, som vi ved, har en betydning[46], men som man kunne ønske at få yderligere belyst.

VI: Fiskeolie

n-3 fedtsyrer er essentielle fedtsyrer, dvs. fedtsyrer der er nødvendige for den menneskelige organisme, men som vi ikke selv kan danne og derfor er nødt til at indtage med kosten. Der findes 2 grupper af essentielle fedtsyrer – n-3 og n-6. Fedtsyrerne har 2 funktioner – dels indgår de som strukturelle komponenter i cellevæggene, dels omdannes de til vigtige aktive molekyler, kaldet prostaglandiner. Der findes 3 typer af prostaglandiner – PG_1, PG_2 og PG_3:
PG_1 har flere vigtige egenskaber og hæmmer bl.a. sammenklumpning af de røde blodlegemer samt mindsker graden af inflammation. *PG_2* har den modsatte effekt af PG_1. PG_2 øger inflammation, kontraherer blodkar samt fremmer sammenklumpning af de røde blodlegemer. Disse egenskaber er vigtige når fx et sår skal hele, men i overflod kan det blive skadeligt for kroppen. *PG_3* har blandede funktioner, hvoraf en af de vigtigste er, at mindske raten hvormed PG_2 dannes. PG_3 bliver derfor, ligesom PG_1, beskrevet som havende anti-inflammatoriske egenskaber.

Omdannelsen af de essentielle fedtsyrer til prostaglandiner sker ved hjælp af enzymer. Det er de samme enzymer der bliver brugt til både n-3 og n-6 fedtsyrerne, men enzymerne foretrækker n-3, dvs. at n-3 først og fremmest omdannes hvis både n-3 og n-6 er til stede. n-6 fedtsyrer kan enten omdannes til det anti-inflammatoriske PG_1, eller til arachidon syre, der videreomdannes til PG_2 (inflammatorisk). Omdannelsen til PG_1 foregår uden enzymer, hvorimod omdannelsen til arachidon syre er enzym-afhængig. Ved en diæt rig på n-3 fedtsyrer vil størstedelen af enzymerne blive brugt til at omdanne n-3, og kun en lille del kunne bruges på omdannelsen af n-6 til arachidon syre og videre til PG_2. n-6 ender derfor hovedsageligt med at blive omdannet til det PG_1, og en n-3 rig diæt vil altså mindske graden af inflammation. Omvendt vil en diæt fattig på n-3 medføre øget omdannelse af n-6 til arachidon syre og dermed PG_2, dvs. øge graden af inflammation. n-6 er ikke i sig selv skadeligt, men derimod er det forholdet mellem indtaget af n-3 og n-6 fedtsyrer der er det essentielle. Desværre er den typiske vestlige kost rig på n-6 og fattig på n-3 fedtsyrer, og favoriserer altså dermed dannelsen af de inflammatoriske prostaglandiner[47-49].

Prostaglandinerne påvirker også vores immunsystem. PG_2 forskyder forholdet mellem Th1 og Th2 i Th2's favør, og vil således påvirke immunsystemet i retning af det mere allergigivende respons. Forholdet mellem Th1 og Th2 er særlig vigtig i det tidlige liv, formodentlig allerede intrauterint [50, 51]. En diæt fattig på n-3 fedtsyrer, i forhold til n-6, vil altså via PG_2 dels øge den generelle grad af inflammation og dels forskyde immunsystemet i retning af Th2 og dermed mod astma og allergi.

Studier har vist at moderens indtag af fisk – som netop er rigt på n-3 fedtsyrer - i den sidste del af graviditeten har betydning for barnets senere risiko for udvikling af astma. Jo større indtag af fisk, jo lavere risiko for astma [52-57]. Disse studier har dog alle været spørgeskema-baserede, enkelte retrospektivt flere år senere, hvor man har spurgt til moderens diæt og sammenholdt det med barnets senere risiko for udvikling af atopi. I et enkelt studie (Olsen *et al* [58]) har man i randomiseret form givet kvinderne tilskud af fiskeolie hhv. placebo i form af olivenolie, og har senere fundet, at færre af børnene til de kvinder der fik fiskeolie siden hen udviklede astma og allergi. Dog er denne opgørelse gjort retrospektivt med de komplikationer det knytter sig hertil.
Så vidt vi ved, foreligger der endnu ingen randomiserede, prospektive interventionsstudier med henblik på muligheden for at mindske risikoen for udvikling af astma og allergi ved at give et tilskud af fiskeolie (rigt på n-3 fedtsyrer) til mødrene i 3. trimester.

Vi ønsker derfor at undersøge muligheden for at mindske barnets senere risiko for at udvikle atopi, ved at give moderen øget tilskud af n-3 fedtsyrer, i form af fiskeolie, og dermed allerede intrauterint påvirke samspillet mellem prostaglandiner, Th1 og Th2.

Olsen *et al* [59, 60] har desuden undersøgt om n-3 fedtsyrer via dets påvirkning af prostaglandiner har en gunstig indvirkning på præterm fødsel. Konklusionen hvad angår præterm fødsel er usikker, men det tyder på at n-3 fedtsyrer generelt kan forlænge graviditeten 3-4 dage, men det er endnu uvist om n-3 fedtsyrer også kan forhindre for tidlig fødsel. Dette vil vi derfor undersøge som sekundært mål.

VII: Immunforsvaret

Børnenes immunforsvar undersøges ved brug af 2 forskellige metoder – dels en undersøgelse af luftvejenes immunforsvar vha. filterpapirer i næsen, dels en undersøgelse af navlesnorsblodets celler og stamceller.

Immunforsvarets funktion i luftvejene (næsen) hos raske småbørn undersøges ved hjælp af filterpapirer i næsen for derved at belyse og forstå den senere udvikling af atopi (astma, eksem og allergi) hos børnene. Ifølge "Hygienjehypotesen" [61] grundlægges tendensen til atopiske lidelser allerede i den tidlige barndom i forbindelse med modningen af immunforsvaret, hvor det medfødte antistofproducerende Th2 respons ændres til det mindre allergigivende cellemedierede Th1 respons. Manglende mikrobiel stimulering af immunforsvaret eller eksponering med en uhensigtsmæssig sammensætning af mikroorganismer fører formodentlig til en skævvridning af immunforsvaret i allergigivende retning[62, 63].
Som anført har COPSAC påvist en 3-4 gange øget risiko for udvikling af astma hos børn, der som spæde (4 uger gamle) havde visse bakterier i luftvejene[64]. Efterfølgende har vi fundet ud af, at sammenhængen mellem bakterier i luftvejene og den senere udvikling af astma hænger sammen med mutationer i T-celle receptoren. Det vil altså sige, at asymptomatiske børn med en medfødt ændring i deres immunforsvar (via T-celle receptoren) har en øget risiko for senere hen at udvikle astma (*Bisgaard et al*, indsendt mhp. publikation). Det synes derfor essentielt også at undersøge hvorledes immunforsvaret fungerer lokalt i luftvejene hos raske småbørn før de udvikler atopisk sygdom.

Traditionelt monitoreres immunforsvarets funktion i næsen ved at måle koncentrationen og fordelingen af cytokiner og kemokiner, som er signal stoffer i immunforsvaret. Dette har man gjort ved at undersøge næseskyllevæske (nasal lavage), hvilket er svært at udføre på småbørn og derfor har ført til divergerende resultater[65]. Dette har ført til udviklingen af specielle filterpapirer, som lægges op i næsen og absorberer næse-sekret, hvorefter det har været muligt at måle høje koncentrationer af cytokiner og kemokiner. Studier med filterpapirer har dog primært været udført på voksne høfeber-patienter efter fremprovokering af symptomer ved indsnusning af pollenekstrakter[66, 67].

På Dansk BørneAstma Center har vi tidligere gennemført et pilotprojekt i samarbejde med en førende høfeber-enhed i London, UK, hvor 45 skolebørn blev undersøgt med specielle filterpapirer; SAM (Synthetic Absorptive Matrix); uden forudgående pollen provokation. Dette pilotstudie viste, at det var muligt at måle koncentrationen af en lang række centrale cytokiner/kemokiner i næsen hos børn (*Chawes et al*, upublicerede data).

Denne simple og let gennemførlig metode til at monitorere immunforsvarets funktion lokalt i luftvejene ønsker vi at applicere til ABC kohorten, for at opnå indsigt i den afgørende skævvridning af immunforsvaret, som formodentlig fører til astma og allergi senere i livet.

Navlesnorsblodet undersøges for indholdet af allergi-antistoffer (IgE), immunforsvarets celler og forstadier til disse (stamceller) for derved at belyse og forstå udvikling af atopiske sygdomme (astma, eksem og allergi) senere i livet.

Tendensen til udvikling af atopisk sygdom grundlægges tidligt i livet og opstår formodentlig i et samspil mellem gener og miljøfaktorer i forsterlivet og neonatal perioden.

Navlesnors IgE måles for at kunne undersøge tendensen til allergi hos det nyfødte barn. Forstadier til immunforsvarets celler (stamceller) undersøges ved stimulation til vækst og modning til centrale celler i den allergiske reaktion (mastceller) [68, 69]. Derudover undersøges indhold og funktion af de modne celler fra immunforsvaret i navlesnorsblodet. Håbet er at kunne isolere specifikke immunologiske sub-grupper (fænotyper), som giver ophav til udvikling af atopisk sygdom senere i livet.

VIII: Analyse af udåndingsluften

Udåndingsluften analyseres vha. en ”elektronisk næse” der en sensor, der kan analysere sammensætningen af gasser i udåndingsluften og derved bruges til prædiktion og diagnostik af

astma hos småbørn. De nuværende objektive metoder til diagnosticering og monitorering af astma hos småbørn er begrænsede. Astma er en heterogen samling af tilstande med uspecifikke symptomer samt forskelle i udløsende årsager, prognose og behandlingsrespons [70]. En bedre forståelse af sygdommen, herunder en objektiv undergruppering vha. den ”elektroniske næse” samt en mere effektiv metode til diagnosticering kan bidrage til forbedret forebyggelse og behandling. Forholdet mellem gasser i udåndingsluften kan variere fra individ til individ, afhængig af både ydre og indre faktorer. Processer i kroppen, herunder astma, kan påvirke indholdet af gasser i udåndingsluften. Nitrogen monooxid (NO) er en kendt markør for astma hos børn, men er formodentlig ikke den eneste gas af interesse. I forsøget med den ”elektroniske næse” forventes det at finde specifikke astma gas-mønstre hos børn med astma-lignende symptomer.
Den elektroniske næse er et bærbart måleudstyr, som er baseret på nanoteknologi. Den er tidligere afprøvet på voksne i en hollandsk undersøgelse [71], hvor det lykkedes at skelne mellem raske forsøgspersoner og astmatikere vha. gas-mønstre. Vi ønsker at afprøve den elektroniske næse på børn fra ABC-kohorten for at undersøge hvorvidt metoden er anvendelig til prædiktion og diagnostik af børneastma. Ydermere vil vi undersøge, hvorvidt den elektroniske næse kan adskille:

1) luftvejssymptomer på baggrund af virus, bakterier eller af ukendt genese
2) asymptomatiske børn med og uden bakterier i luftvejene
3) børn med forskellige defekter i immunforsvaret
4) børn udsat for forskellige astma- risikofaktorer tidligt i livet (fx tobaksrøg og pelsdyr)

IX: Den neurologiske udvikling

Formålet er at vurdere om der findes en sammenhæng mellem mors indtagelse af fiskeolie hhv. olivenolie under graviditeten påvirker barnets senere neurologisk udvikling.
Tilbuddet af fiskeolie i fostertilværelsen er vist at være vigtig for hjernens vækst og udvikling. Samtidig er det vist, at barnets senere psykomotoriske udvikling, herunder kognitive funktion, sprog og grov-motorik, afhænger af hjernens udvikling i fostertilværelsen. Det er derfor nærliggende at forestille sig, at et øget tilskud af fiskeolie under graviditeten vil fremme barnets psykomotoriske udvikling. På trods af at flere randomiserede studier har forsøgt at vise denne sammenhæng, er konklusionen stadig tvivlsom. Et større studie indikerede, at der kunne være en sammenhæng

mellem mors indtag af fiskeolie og barnets senere psykomotoriske udvikling, men de resterende studier har overvejende været små og inkonklusive.

Børnenes neurologiske udvikling vurderes vha. tre metoder:

- Registrering af milepæle (mhp. den motoriske udvikling)
- Sprogtest
- Bailey III test (mhp. den kognitive udvikling)
- Ages & Stages Questionnaires (ASQ-3) - kommunikation, grovmotorik, finmotorik, problemløsning og personlig-social udvikling.
- Child Behaviour Checklist (CBCL) fra the Achenbach System of Empirically Based Assessment (ASEBA), til børn fra 6-16 år.

X: Lungefunktionen

Diagnose og behandling af astma er i dag guidet af barnets symptomer og måling af lungefunktion ved traditionel spirometri, som primært afspejler forandringer i proksimale luftveje, og er vanskelig at gennemføre på børn under 6 år. Der er ringe overensstemmelse mellem barnets symptomer og luftvejsinflammation samt mellem luftvejsinflammation og resultatet af spirometri.[72-74] Dette kan resultere i mangelfuld astma behandling til især de subkliniske astmatyper.

Astma kan være udtrykt ved sygdom i de distale luftveje før det kan måles ved traditionel spirometri.[75, 76] Med helkropsplethysmografi og især MBW kan vi detektere subklinisk astma tidligt[77, 78] og beskrive udviklingen af astma over tid helt fra 3 års alderen, hvor man ikke med traditionelle diagnostiske redskaber kan gennemføre effektive målinger af lungefunktion. Metodernes resultater sammenlignes indbyrdes og sammenholdes med børnenes kliniske tilstand samt diagnoser.

Fra 6 års alderen suppleres lungefunktionsundersøgelserne yderligere. Spirometri er den mest almindelige lungefunktionsmåling, der kan udføres fra børnene er ca. 6 år gamle og anses for ”golden standard”. Den beskriver luftvejsmodstand i de øverste dele af luftvejene. For at vurdere følsomheden i luftvejene kan man provokere med stoffet metacholin, der irriterer slimhinderne. Derudover sammenlignes målingen med MBW, der forventes at kunne måle inflammation i de yderste dele af lungerne. Denne metode menes at kunne detektere astma i tidligere stadier end traditionel lungefunktionsundersøgelse med spirometri og helkropsplethysmografi, der primært måler inflammation i de øverste lungeafsnit.

Derudover udføres en FeNO-måling, der måler fraktioneret nitrogenoxid i udåndingsluften fra børnene. Øgede FeNO-værdier kan være tegn på inflammation i luftvejene og astma. Metoderne har alle været anvendt i flere år på både små og store børn med astma.

XI: Vækst

Mødrene i ABC kohorten har alle været randomiseret til indtagelse af højdosis D vitamin samt fiskeolie gennem sidste del af deres graviditet og disse tilskud har potentiel virkning på børnenes knogle- og fedtvævsudvikling,[79, 80] men ingen studier har endnu vist en sammenhæng på et så stort antal deltagere som det aktuelle.

Med Dual X-ray Absorptiometry (DXA) scanning kan vi undersøge præ- og postnatale faktorers betydning for fedtmasse og knoglemineraliseringen. Prævalensen af overvægt og osteoporose er nærmest epidemisk i den vestlige verden. Overvægt i voksenlivet er associeret med vækst i barndommen,[81, 82] og selvom osteoporose karakteriseres som en sygdom hos den ældre del af befolkningen, er der nu bred enighed om, at dispositionen starter i barndommen.[83-85] Vi ønsker derfor at undersøge børnenes vækst og knoglemineralisering, og hvilke faktorer, der er af betydning for disse.

XII: Øvre luftvejes anatomi

Astma er en kronisk inflammationssygdom i lungerne og hænger sammen med sarte og overreagerende lunger med luftvejs obstruktion. Lignende symptomer kan imidlertid ses ved obstruktion af øvre luftveje som ved rhinitis og hypertrofi af de adenoide vegetationer (polypper) i rhinopharynx, der giver sig til kende ved mundrespiration, snorken og søvnapnø. Sådanne natlige symptomer kan i alvorlige tilfælde føre til nedsat kognitiv funktion. Derudover er astma og rhinitis oftest sameksisterende og mange non-astmatikere med allergisk rhinitis har nedsat lungefunktion.[86]

Med akustisk rhinometri af øvre luftveje vil vi undersøge association mellem obstruktion af øvre luftveje og forældrenes rapportering af astmatiske symptomer samt barnets lungefunktion.

Samfundsøkonomiske forhold

Som nævnt er de atopiske sygdomme; astma, atopisk dermatit og allergi de største folkesygdomme hos børn og de hyppigste årsager til hospitalsindlæggelse hos småbørn. Disse sygdomme har derfor stor samfundsøkonomisk betydning pga. øget lægekontakt og medicinforbrug samt forældrenes hyppigere sygefravær i forbindelse med barnets 1. sygedag[1]. Sygdommene er således af stor betydning for børns sundhedstilstand og livskvalitet og dermed også for familiernes. Endvidere er det af særlig betydning at behandle astma hos børn tidligt for at forhindre lungeskader og senfølger i voksenalderen[87].

Specielt delstudiet om opgørelse af almindelig sygelighed hos småbørn har betydning for store dele af den danske befolkning, idet det vedrører stort set alle børnefamilier, enten direkte eller indirekte på arbejdspladsen, da omkring halvdelen af den danske arbejdsstyrke har børn under 12 år. Der har fra politisk side været fokus på børnefamiliernes balance mellem arbejdsliv og familieliv.
I trivselsanalyser beskriver forældre sygdom hos børnene som en faktor der fører til højere stressniveau[88]. En tredjedel af forældre har sendt et barn, der ikke var rask i daginstitution, da der ikke var andre pasningsmuligheder. Herved øges smittekæderne i børneinstitutionerne med øget sygelighed som resultat[46]. Kun 13 % af forældrene har børn, der når at blive raske i de dage, deres arbejdsplads giver mulighed for at være hjemme. Resten arrangeres ved at forældrene selv melder sig syg, tager ferie- eller fridage eller mobiliserer netværk, hovedsagelig bedsteforældrene. [89, 90]
Øget viden om forholdende vedrørende børns sygdom og eventuelle forebyggende tiltag vil derfor have betydelige samfundsøkonomiske konsekvenser.

Metode
Studiet udføres som en prospektiv kohorteundersøgelse af 800 børn, hvoraf mindst den ene forælder har eller har haft astma. Den prospektive kohorte metode er valgt, fordi atopiske sygdomme ikke er konstante fænomener hos børn, men typisk ændrer sig gennem tiden. Da det er den tidlige udvikling af atopiske lidelser, det er interessant at undersøge, er det nødvendigt at undersøge småbørn frem for voksne. Ud af 800 inkluderede børn forventes 200-400[91-93] børn at ville udvikle astmatiske symptomer og/eller rhinit, og omtrent det samme antal at udvikle atopisk eksem[93, 94]. Styrken ved dette studie, sammenlignet med andre lignende undersøgelser, er desuden at alle tidlige data indsamles ved kliniske besøg med max. 6 måneders mellemrum.
Studiet udføres på 2 centre, hhv. i Gentofte og i Næstved og kan derfor geografisk dække hele Sjælland og øerne. Herved opnås forholdsvis bred rekruttering af deltagere, hvilket øger kohortens repræsentativitet i forhold til baggrundsbefolkningen.

Forsøgsforløb

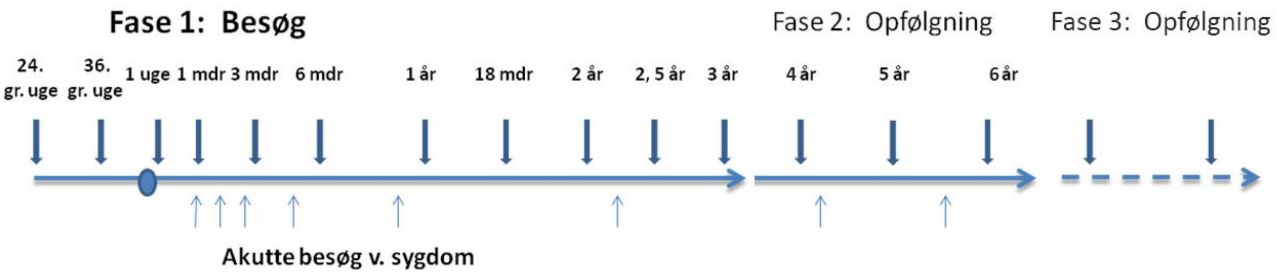

Interesserede gravide mødre tilbydes deltagelse. Nyfødte der opfylder inklusionskriterierne inkluderes indenfor første levemåned. De følges i ABC-klinikken med halvårlige besøg samt ved akutte hud- eller luftvejsrelaterede symptomer. I første omgang er der planlagt besøg for de første 3 leveår. Herefter vil børnene blive set til klinisk opfølgning.
Planen er at fortsætte studiet med 3 opfølgende besøg ved alderen: 4 år, 5 år og 6 år. Formålet med studiet er stadig, at få bedre viden om astmasygdommens udvikling og at finde nye metoder til at forudsige om et barn vil udvikle astma, allergi og eksem. Derudover fokuseres der på andre faktorer med indflydelse på børnenes generelle sundhed og udvikling. Dette gøres primært med velkendte undersøgelser fra tidligere besøg. Børnene tilbydes tillige undersøgelse ved akutte episoder med luftvejs- og hudproblematik, som vi har gjort i fase 1.

Fase 3 er endnu ikke planlagt i detaljer, men forventes at være med 2-5 års mellemrum. (Der vil senere blive søgt tilladelse til denne opfølgning.)

Dagbog

Mellem besøgene registrerer familien atopi-relaterede symptomer samt almindelige infektioner i en udleveret dagbog i afkrydsningsformat. Dagbogen gennemgås med familierne ved besøgene og indtastes herefter i en til formålet oprettet database.

Planlagte besøg

Udlevering af fiskeolie/olivenoliepaksler

Ved første besøg i klinikken (graviditetsuge 24), randomiseres kvinderne i forholdet 1:1 til at indtage enten fiskeolie eller olivenolie. Interventionen står på indtil 1. besøg på DBAC efter fødslen (uge 1-2). Olivenolie anvendes som placebo. Metoden er valgt som kopi af føromtalte retrospektive interventionsstudie af Olsen *et al* [95], hvilket dels øger sandsynligheden for et positivt out-come, dels øger muligheden for bedst muligt efterfølgende at kunne sammenligne de to studier.

Som fiskeolie-tilskud planlægges at give fire kapsler Incromega[1] (Omega 3: 60 % min; EPA: 33 % min; DHA: 22 % min) Som placebo gives fire olivenolie kapsler à 1 g, indeholdende 72 % oleinsyre (n-9) og 12 % linolinsyre (n-6).

Mængden af n-6 fedtsyre i olivenolie svarer til under 3 % af en gennemsnitlig kvindes daglige indtag, og betyder derfor ikke noget for forholdet mellem n-3 og n-6. Derimod svarer indholdet af n-3 i fiskeolie-kaplserne til ca. 10 gange det gennemsnitlige daglige indtag af n-3 fedtsyrer.

De gravide vil få udleveret alle kapsler til eget forbrug ved besøget i uge 24 og skal levere emballage og ubrugte kapsler retur. Kapslerne vil være påskrevet randomiseringsnummer og det vil ikke være synligt om kapslerne indeholder fiskeolie eller olivenolie. Kvinderne informeres om dette. Kvinderne vil blive tildelt enten fiskeolie- eller placebo-kapsler via en tilfældig randomiseringskode-generator.

---

[1] **Croda Nordica AB,** Krossverksgatan 5 C, SE-216 16 Limhamn, Sweden
Pharmatech AS, Po 85, 1662 Rolvsøy, Norway

Interview

Ved besøgene indsamles information om risikofaktorer for udvikling af atopiske lidelser ved standardiserede interviews af forældrene.

Ved interview spørges til

- Pre-, peri- og neonatal eksponering (kun første besøg): Graviditetskomplikationer, paritet samt maters indtagelse af medicin, alkohol og tobak, infektioner, gestationsalder, fødselsmåde, APGAR og antropometrik.
- socioøkonomiske forhold: forældres uddannelse og erhverv og husstandsindkomst
- miljømæssige forhold: bolig (herunder type og areal), pasningsforhold (herunder start i institution og antallet af børn), søskende, kæledyr

-ernæring: Ammeperioden og introduktion af andre fødeemner

Kliniske undersøgelser

Ved hvert besøg udføres almindelig ikke-invasiv lægelig helbredsundersøgelse af børnene med fokus på astma, allergi og eksem. Højde/længde og vægt registreres.

Derudover udføres undersøgelser planlagt til de forskellige besøg. Tidspunkterne fremgår af oversigt, bilag 1.

Priktest

En standardiseret priktest er den foretrukne undersøgelse ved udredning af allergisk sygdom hos børn. Det vandige allergenekstrakt bringes i kontakt med huden, og der prikkes forsigtigt på huden med en lancet gennem dråben. Derefter aflæses en eventuel hævelse på få millimeter på huden (allergisk reaktion). Undersøgelsen betragtes af mange som pædagogisk, da man relativ hurtigt får svar. Der er planlagt yderigere 1 undersøgelse ved 6 års alderen.

Tympanometri

Måling af trykket i mellemøret med tympanometer, der placeres ved øreåbningen i ca. 10 sekunder. Anvendes som standard i almen praksis.

Aktivitetsmåling

I forbindelse med 1 års besøget udleveres en aktivitetsmåler, der skal bæres i 2 uger med henblik på undersøgelse af børnenes bevægemønster. Måleren er ca. 2 X 2 cm, vejer ca. 10 gram og bæres på benet. Formålet er kortlægning af barnets bevægemønster og eventuelle forskelle i aktivitetsniveauet hos børn, der rammes af atopiske lidelser, såvel dagligt som under søvn. Aktivitetsmåleren er afprøvet ved flere kliniske besøg, herunder i COPSAC.

Elektronisk næse

Børnene vil blive undersøgt ved alle akutte besøg samt 1 og 18 måneders besøgene i klinikken. På børnene placeres en maske, som dækker næse og mund, og som er sammenkoblet med en beholder, hvori udåndingsluften opsamles ved 1-2 minutters rolig vejrtrækning. Den elektroniske næse bliver efterfølgende tilkoblet beholderen og luftprøvens indehold analyseres. Opsamlingen udføres 2 gange af hensyn til reproducerbarheden. Den efterfølgende analyse af den opsamlede luftprøve tager kun 1 minut, og alt i alt forventes undersøgelsen at tage omkring 15 minutter. Resultaterne fra det enkelte barns 2 separate målinger sammenlignes mhp. at beskrive reproducerbarhed og optimering af metoden. De individuelle profiler vil herefter sammenlignes og analyseres for specifikke mønstre. Der er planlagt yderigere 1 undersøgelse ved 6 års alderen.

Multiple Breath Washout

Dette er en ny metode, til at måle udvaskningen af inaktive gasser fra lungerne for at påvise sygdom i de distale luftveje.[77] Det er en meget sensitiv markør for tidlig udvikling af sygdom i luftvejene hos børn med astma.[96-99] Der bestemmes et såkaldt lung clearence index (LCI), som er et mål for inhomogen ventilation i lungerne. LCI har et smalt normalt-spektrum og er uafhængig af alder.[100, 101] Desuden måles S-cond (bidrag til MBW fase III fra de ledende (proksimale) luftveje) og S-acin (bidrag til MBW fase III fra de respiratoriske (distale) luftveje). Målingerne foregår med barnet i siddende stilling, hvor det trækker vejret roligt gennem et mundstykke. Der bliver gennem mundstykket indåndet ren ilt og på denne måde, er det muligt, at måle hvor længe barnet er om at udskifte alt eksisterende luft i lungerne ved at måle på nitrogen niveauet i udåndingsluften. Hver enkelt måling tager nogle minutter og er uden nogen form for ubehag, målingen bliver gentaget i løbet af besøget. Vi forventer, at resultaterne fra undersøgelsen kan afsløre tegn til subklinisk bronkial obstruktion.

Helkropsplethysmografi

Her måles lungefunktionen ved registrering af luftvejsmodstanden.[102, 103] Helkropsplethysmografi kan bruges til at detektere subklinisk astma hos børn helt ned til 2 år, hvor traditionelle målemetoder, som spirometri, er vanskelige at gennemføre. Barnet sættes i en forseglet boks med kendt volumen, som medfører, at trykændringer i boksen, forårsaget af barnets thoraxbevægelse under vejrtrækningen, kan måles. Respirationsflowet registreres via et flowmeter, hvorigennem barnet trækker vejret og den specifikke luftvejsmodstand udtrykkes som forholdet mellem trykforandring og flow.[104, 105] Metoden er ikke-invasiv, tager omkring 5 minutter, er uden ubehag, og er effektiv til at diagnosticere tidlig astma hos børn selv i perioder, hvor de er symptomfrie.[106] Metoden anvendes i dag rutinemæssigt på landets børneafdelinger.

Spirometri med metacholin provokation

Undersøgelsen foregår i siddende stilling, hvor barnet inhalerer dybt og derefter tager mundstykket i munden så læberne slutter tæt. Barnet foretager maksimal udånding så kraftigt og hurtigt som muligt. Barnet fortsætter udåndingen indtil undersøgelsen er tilfredsstillende. Ved metacholinprovokationen forstøves stoffet metacholin og inhaleres omtrent som fra en astma-spray. Der indåndes først meget små mængder, senere mere og mere. Lungefunktion følges ved spirometri mellem hver inhalation. Metacholin fremkalder en forbigående forsnævring af luftvejene, som kan påvise luftvejenes følsomhed. Der er ikke alvorlige bivirkninger ved spirometri eller metacholin undersøgelsen. Der kan opleves lettere vejrtrækningssymptomer. Hvis målingen viser, at et astmaanfald er på vej, standses undersøgelsen, og der gives straks astmamedicin.

FeNO-måling

FeNO-målingen foretages ved at puste ud gennem et mundstykke, der måler fraktioneret NO indhold i udåndingsluften. Øgede FeNO-værdier kan være tegn på inflammation i luftvejene og astma. FeNO målingen er helt ufarlig.

Dual X-ray Absorptiometry

Her måles kropssammensætningen, hvor man ved røntgen-gennemstråling måler kropskomponenter ud fra den passerede strålemængde, registreret af en detektor på modsatte side af barnet[107, 108] Blødt væv, som hovedsagelig består af vand og organiske forbindelser begrænser stråleflux i mindre grad

end knogle og man kan derigennem måle knoglemasse og -mineralisering og fedtmassen for hele kroppen samt i udvalgte regioner.

Barnet ligger fladt på ryggen og scanningen foretages fra hoved til tå i en bevægelse af ca. 3 minutters varighed.

Der er planlagt yderigere 1 undersøgelse ved 6 års alderen.

Akustisk rhinometri

Dette er en undersøgelse af pladsforholdene i næsen og næsesvælget og anvendes til diagnosticering af rumopfyldende processer som adenoide vegetationer samt forsnævringer, der er et fremtrædende ved allergisk som non- allergisk rhinitis.[109] Metoden er baseret på refleksionen af et akustisk signal, som via en probe sendes gennem næsekaviteten. Undersøgelsen tager under 5 minutter og er ikke forbundet med nogen former for ubehag eller risici for barnet.

Blodtryk

Der tages et blodtryk på børnene i forbindelse med planlagte undersøgelser. Undersøgelsen er ikke forbundet med nogen former for ubehag eller risiko for barnet.

Milepæle

Et skema (bilag 4) udleveres ved først kommende besøg i klinikken. Forældrene instrueres i at registrere datoen for, hvornår barnet opnår milepæle som at sidde, gå og stå. Denne registrering gennemgås med klinikkens personale ved hvert besøg.

Sprogtest

I forbindelse med barnets 1 og 2 års besøg får forældrene udleveret et informationsbrev med bruger-id samt password (bilag 5) til en database kaldet ”tidlig sprogvurdering” (www.cdi.sprogvurdering.dk), som er baseret på en dansk adaption af den amerikanske forældrerapport MacArthur Bates Communicative Inventori (CDI). Forældrene instrueres i at svare på spørgsmålene indenfor +/- 14 dage fra barnets fødselsdag. Det tager ca. 20min at lave testen. Databasen kører som et pilotprojekt på Syd Dansk Universitet, Center for Børnesprog. Der er lavet en kontrakt mellem SDU og DBAC, der indebærer at vi (DBAC) har retten til alle data.

Bayley III test

I forbindelse med barnets 2,5 års besøg undersøges barnets kognitive funktion i forbindelse med den lægeundersøgelse, der alligevel finder sted ved hvert besøg. Undersøgelsen foregår som en struktureret leg og varer ca. 20-30 min hvor barnet får opgaver som relaterer sig til forskellige stadier i dets udvikling. Børnene får points alt efter hvor mange opgaver de kan løse. For at gøre denne pointscoring nemmere, samt til at gennemgå testen igen ved tvivlssituationer videooptages testen. Til optagelsen bruges et simpelt webcam og filerne bliver opbevaret under barnets ABC nr på et lukket drev tilhørende ”ABC studiet”. Forældrene bliver bedt om at give giver separat, skriftlig tilladelse til optagelsen.

Ages & Stages Questionnaires (ASQ-3)

I forbindelse med 3 års besøget beder vi forældrene at udfylde et skema, Ages & Stages Questionnaires (ASQ-3) som dækker; kommunikation, grovmotorik, finmotorik, problemløsning og personlig-social udvikling. Skemaet er valideret og har tidligere været brugt til forskning i Danmark.[110]

Child Behaviour Checklist (CBCL)

I forbindelse med 6 års besøget beder vi forældrene at udfylde et skema, Child Behaviour Checklist (CBCL) til at vurdere børnenes mentale sundhed og adfærdsmæssige udvikling. Skemaet er valideret og er i 2010 blevet re-standardiseret i Danmark.

Ultralydsscanning af fingerknoglerne

Scanningen udføres for at bestemme knogledensiteten. Undersøgelsen foregår ved at påsætte en ’klemme’ på barnets fingre (en af gangen) på den ene hånd.

Biologisk materiale

Ved besøgene opsamles forskelligt biologisk materiale. Alle prøver opbevares i en forskningsbiobank til senere analyse. Biobanken udgøres af -80 graders frysere placeret på hhv. Gentofte Hospital og Næstved Sygehus i et aflåst lokale på biokemisk afdeling. Efter vanlig standard er der alarm på fryserne. Prøverne gemmes til barnets 18. leveår. Hvis en deltager trækker sit samtykke til deltagelse tilbage, destrueres alt indsamlet biologisk materiale. Hvis forældrene blot ønsker udmeldelse af kohorteundersøgelsen, men er indforståede med at det indsamlede materiale

fortsat kan opbevares og anvendes i analyser, gemmes det fortsat til barnets 18. år efter skriftligt og mundtligt informeret samtykke. Det er vores erfaring fra COPSAC, at forældre, der udmelder sig, ofte ønsker sidstnævnte, så tid og energi investeret i projektet ikke har været uden formål.

Navlesnorsblodprøve

Jordmoder udtager ved fødslen en blodprøve fra navlesnoren i særligt udleverede prøveglas. Prøven benyttes til undersøgelse af blodoverførte antistoffer fra mor til barn mod allergener samt indholdet af D-vitamin. Ved fødsler, hvor kvinden føder ved planlagt kejsersnit, undersøges navlesnorsblodet umiddelbart efter fødslen. Immunforsvarets celler oprenses og undersøges for overflademarkører samt udsættes for stimulationsforsøg for at belyse deres funktion. Ved alle øvrige fødsler tilsættes reagens til blodet, som beskytter stamcellerne og prøverne opbevares ved -80°C mhp. senere oprensning og undersøgelse af stamceller.

Blodprøver

Ved 6 måneder og 2 år tages en blodprøve fra forsøgsdeltagerne (hhv. 10 og 14 ml). Begge forældre afleverer én blodprøve ved inklusionen (20 ml). Der tages desuden blodprøve på mater ved første besøg efter fødslen (20 ml).

Prøverne bruges til undersøgelse af sammenhængen mellem genetik og udvikling af atopisk sygdom, herunder GWA.

Maters blodprøver bruges desuden til at bestemme indholdet af omega-3 i erythrocyt-membranen. Blodprøverne tages dels for efterfølgende statistisk at kunne tage højde for de kvinder der i forvejen har et højt indtag af fisk/fiskeolie og som derfor har et naturligt højt indhold i blodet af omega-3 og dels for at kontrollere kvindernes compliance i forhold til projektet.

Desuden undersøges børnenes prøver for almindeligt anvendte biokemiske parametre i monitorering af helbredsstatus (se bilag 3).

Ved 6 års alderen er der planlagt endnu en blodprøve (20 ml) fra forsøgsdeltagerne. Prøverne bruges til undersøgelse af sammenhængen mellem genetik og udvikling af sygdom samt analyse af biomarkører og allergiantistoffer, som kan sammenholdes med helbred og sygdom.

Luftvejssug

Ved 1 uge, 1 måned og 3 måneder tages et larynxsug samt næsesug, dvs. 2 sug af sekret fra luftvejene (2 gange 5 ml). Desuden suges der sekret ved akutte luftvejssymptomer.

Modermælk

Mødrene afleverer en prøve på 20 ml indsamlet i uge 1 eller 2. Mælken anvendes til analyse af D-vitaminindholdet samt fedtsyresammensætning som en del af kortlægningen af barnets ernæringsprofil.

Hårprøve

En årlig hårprøve på 1 cm (2 gram).

Hårprøverne anvendes til nikotin/cotinin-analyse, et estimat for graden af eksponering for passiv rygning.

Der er planlagt yderigere 1 hårprøve ved 6 års alderen.

Nasalskrab

Der planlægges udført et nasalskrab ved 6 års alderen. Nasalskrabet udføres med barnet i siddende stilling. Lægen kigger ind i næsen og holder det ene næsebor åbent. Der indføres en lille skarpske og skrabes på næsens skillevæg så man får en lille prøve af næsens slimhinde. Undersøgelsen er ikke forbundet med nogen former for risici, men der kan forekomme ubehag i form af smerte og en gang imellem lidt næseblod når skrabet udføres. Materialet fryses ved -80 grader og analyseres senere.

Afføring

3 afføringsprøver (à 15 g) indsamles. De 2 første når barnet er 5 og 10 dage gammel, den tredje når barnet er 1 år. Prøverne sendes til Statens Serum Institut (SSI) der analyserer børnenes tarmflora. Vi planlægger at indsamle yderligere 3 afføringsprøver fra børnene for at følge udviklingen af bakteriesammensætning gennem barndommen. Prøverne tages ved 4, 5 og 6 års alderen. Prøverne fryses ved -80 grader og analyseres senere.

Støv

Ved 6 måneders alderen indsamles støvprøve på omkring 5 gram fra barnets sengetøj. Prøverne sendes til analyse for dyreallergen samt endotoxin og glycan der er delprodukter fra bakterie- og svampecellevægge.

Urinprøve

Ved 3 besøg indsamles 4 ml urinprøve opsamlet ved pose i ble med henblik på analyse af leukotriener og interleukiner, der er immunologiske signalstoffer, som udskilles i urinen. Der er planlagt yderigere 1 urinprøve ved 6 års alderen. Prøverne fryses ved -80 grader og analyseres senere.

Vaginalpodning

Én podning med vatpind fra skeden hos moderen i 24. + 36. graviditetsuge. Udføres under sterile forhold. Sendes til SSI til analyse af mikrofloraen.

Næsefilterpapir

Børnene undersøges ved det planlagte 1 måneds og 2 års besøg i ABC klinikken. Først undersøges næsen af klinikkens læger mhp. at afsløre skæv næseskillevæg eller polypper, som vanskeliggør opsættelse af filterpapir. Hvis næsens anatomi er normal opsættes med pincet et filterpapir á 3x15mm, som skal sidde og absorbere næse-sekret i 2 minutter. En næseklemme sikrer, at papiret forbliver i næsehulen. Efterfølgende tages filterpapiret ud og klargøres til videre analyse. Der er planlagt yderigere 1 filterpapirprøve ved 6 års alderen. Prøverne fryses ved -80 grader og analyseres senere.

Halspodning

Denne udføres ved 3 års besøget for at beskrive mundhulens samt de øverste luftvejes mikroflora. 2 podepinde (Én til virus- og én til bakterieanalyse) føres ind gennem munden simultant og der podes fra ganebuerne. Podningerne fryses ved -80 grader og analyseres senere. Undersøgelsen tager få sekunder og medfører kun et minimum af ubehag og ingen risiko for barnet.

Hudpodninger

Hudpodninger foretages én gang inden for barnets 2 første leveår samt ved 3- års besøget. Med vatpinde podes dels fra volarsiden af underarmen, dels fra armhulen. Prøverne opbevares i fryseren til senere mikrobiologisk undersøgelse.

Akutte besøg

Luftvejssymptomer

Hvis børnene har akutte luftvejssymptomer, der varer 3 dage eller mere, ses barnet i ABC-klinikken. Her undersøges barnet på almindelig vis svarende til besøg hos praktiserende læge, og behandles ved behov. ABC-klinikkens læger har mulighed for kliniske og parakliniske undersøgelser og om nødvendigt indlæggelse af barnet på børneafdeling. Den faste gruppe forskningslæger sikrer kontinuitet og tryghed for familien og barnet på linie med vanlige besøg hos egen læge. Der skrives epikrise til egen læge.
Udover almindelig lægeundersøgelse laves som standard følgende undersøgelser: luftvejssug, næsesug, næsefilterpapir og elektronisk næse. Besøget arrangeres ved at familierne kontakter ABC-klinikken telefonisk. Allerede fra første dag med symptomer kan forældrene få telefonisk vejledning af ABC-klinikkens læger. I weekender og ferier kan man få fat i lægerne på akut-telefon i tidsrummet 12-14.

Hudsymptomer:

Hvis barnet udvikler eksem kan de blive set og behandlet på ABC-klinikken indenfor en uge af læge under uddannelse til dermatolog, superviceret af speciallæger i dermatologi. Ved behov arrangeres opfølgende besøg. Som standard laves undersøgelsen ”elektronisk næse”.

Astma-besøg

Hvis barnet udvikler astma er det ABC-klinikkens læger, der følger og behandler sygdommen efter nationale retningslinjer superviseret af professor Hans Bisgaard. Astmabørnene følges med kontrolbesøg i ABC-klinikken hver 3 måned.

Oversigt over indsamling og besøg kan ses på oversigt, bilag 1.

Forsøgspersonerne

Ved at koble CPR registeret med det ydelsesnummer praktiserende læger indberetter til Sygesikringen ved 1. graviditetskontrol samt teknisk assistance fra CSC/Danmark vil vi få lister med cpr-numre, navne og adresser på de gravide kvinder, der har været til 1. graviditetskontrol. Da stort set alle gravide kvinder i Danmark kommer til 1. graviditetskontrol, opnås hermed muligheden for et repræsentativt materiale for rekruttering. Vi vil skriftligt henvende os til disse kvinder med information om ABC projektet og tilbyde dem uforpligtende at kontakte os, hvis de er interesseret i at høre nærmere.
Kvinder, der udviser interesse vil dernæst blive kontaktet per telefon af en lille gruppe betroede medarbejdere fra ABC-studiet, der vil give en kort information om formål, indhold, risici, form, samt at der er tale om et biomedicinsk forskningsprojekt. Der bliver herefter fremsendt skriftligt informationsmateriale udarbejdet specielt til formålet. Familierne opfordres til at tage stilling til samtykket, og der aftales tid til opfølgende mundtlig information, hvor der også vil være mulighed for, at forældrene kan stille uddybende spørgsmål.
(jf. vedlagte informationsmateriale og samtykkeerklæring).

Inklusionskriterier

- Bopæl: Storkøbenhavn, Sjælland, Møn, Lolland eller Falster.
- Positivt interesseret i deltagelse i ABC-kohorten
- Flydende danskkunskaber
- Forældre/værge indvilger i at lade barnet indgå og er villige til at følge forsøgets procedurer.

Eksklusionskriterier

Ved første lægeundersøgelse ekskluderes barnet, hvis det:

- Forældre/værge ud fra lægens skøn er mentalt eller juridisk inhabil, hvilket forhindrer at der kan indhentes informeret samtykke.
- Deltager i et andet klinisk forsøg med lægemidler.
- Har en kronisk lungesygdom/hjertesygdom

Begrundelse for undersøgelse af mindreårige
Begrundes ud fra kriterium C.

Vi ønsker at undersøge de tidlige årsager til udvikling af atopiske lidelser, og vores formål er i sidste ende at bedre behandlingen og reducere forekomsten af atopiske sygdomme i barndommen. Det er derfor kun muligt alene at inkludere børn.

Da langt hovedparten af patienter med atopisk sygdom er debuteret i de første leveår, kan studiet ikke foretages på ældre personer.

Som anført er astma, allergi og eksem de hyppigste kroniske lidelser hos børn, og forebyggelse og lindring af disse lidelser vil få betydelige og fordelagtige konsekvenser for børn og børnefamilier generelt.

Ca. 20 % af alle børn udvikler astma, allergi eller eksem og størstedelen af disse børn er børn af ikke atopiske forældre. Idet så mange børn udvikler atopiske lidelser og en så stor andel af dem *ikke* er højrisikobørn (dvs. deres forældre har ikke astma), vil alle børn, både høj- og *ikke*-høj- risikobørn vil kunne drage stor fordel af studiet.

Undersøgelserne indebærer efter vores vurdering minimale risici. Generne er hovedsagelige tidsmæssige aspekter, hvilket i højere grad påvirker forældrene end det enkelte barn.

Alle planlagte undersøgelser er evidensbaserede, godkendt til børn og har tidligere indgået i forskningsprojekter med børn. Således har samtlige undersøgelse indgået i COPSAC studiet. Størstedelen anvendes i daglig udredning og behandling. Der er altså intet eksperimentelt element forbundet med deltagelse.

ABC-klinikkens personale har bred erfaring i arbejdet med børn i forskningsprojekter, primært fra COPSAC-projektet. Ved hjælp af pædagogiske principper udføres undersøgelserne gennem leg.

Alle undersøgelser vil blive foretaget i samarbejde med forældrene og med respekt for børnenesgrænser.

Alle undersøgelser udføres ihht Good Clinical Practice Guidelines samt EMEA (European Medicines Agency)'s vejledning 'Ethical Considerations for Clinical Trials on Medicinal Products with the Paediatric Population' baseret på EU-direktiv EC 1901/2006.

Bivirkninger, risici og ulemper

Intervention med fiskeolie/olivenolie er ufarlig og tidligere afprøvet i flere studier. Der er ingen kendte risici ved indtagelse af fiskeolie eller olivenolie for hverken moderen eller barnet.
Herudover er undersøgelsen udelukkende et observationelt studie uden lægemiddelafprøvning så der er ikke tale om bivirkninger. Som nævnt anvendes størstedelen af undersøgelserne i almindelig daglig udredning og behandling af børn.
Ved blodprøver er der er en teoretisk risiko for beskadigelse af årer, men den risiko må anses for yderst minimal
Til alle besøg afsættes rigelig tid. Ved at minimere tidspres reduceres risikoen for komplikationer yderligt i forhold til tilsvarende undersøgelse i hospitalsregi.

Ulemper og komplikationer for forsøgsdeltagerne

Prøver:
Sugninger af sekret fra luftvejene
Der udtages ca. 5 ml. og udføres som larynxsug under sterile forhold med brug af standardudstyr godkendt til hospitaler i Danmark iht. etableret standard.
Den hyppigste komplikation er selvlimiterende blødning fra næsehulen. Proceduren kan i yderst sjældne tilfælde medføre vaso-vagalt anfald og broncospasme, men der findes ikke angivelser for hyppigheden af disse. I sådanne tilfælde er ABC-klinikkens personale kvalificeret og udstyret til at behandle anfaldet, som yderst sjældent er alvorligt[111]

Blodprøver
Der tages blodpøver 2 gange, hhv. ved 6 måneder og 2 års-alderen, samt ved 6 års alderen, forudgået af lokalbedøvende salve (EMLA-creme).
Der tages blodprøve én gang for hver forælder.
Dette er rutineprøver. Der er en teoretisk risiko for beskadigelse af blodåren, for infektion ved indstiksstedet og for blødning hos mennesker med. Disse risici anses for at være minimale. Alle prøver udtages under sterile forhold med brug af standardudstyr godkendt til hospitaler i Danmark. EMEA anbefaler at blodvolumen ikke overstiger 1 % af samlet blodvolumen af gangen (8 ml pr. kg kropsvægt) og 3 % over 4 uger, hvilket ikke overskrides.

Vaginalpodning

Én podning med vatpind fra skeden hos moderen i 24. + 36. graviditetsuge. Udføres under sterile forhold. Dette er en klinisk rutineprøve uden kendte komplikationer.

Næsefilterpapir

Der er ikke rapporteret alvorlige bivirkning ved denne undersøgelse, som er afprøvet i flere studier. I pilotstudiet oplevede nogle børn, at det kildede i næsen, nogle fik nyseture, mens kun ét barn, som havde svær høfeber, fik let næseblod.

Undersøgelser:

Priktest

Her er der en teoretisk mulighed for systemisk allergisk reaktion på det allergifremkaldende materiale. Der er endnu ikke dokumenteret sådanne anfald på trods af testens udbredte brug. Alligevel er der altid medicin til rådighed til behandling af et sådant anfald (adrenalin).

Aktivitetsmåling

Barnet skal bære en aktivitetsmåler på ca. 2 x 2 cm om benet over en 2 ugers periode. Der er ingen kendte komplikationer.

Elektronisk næse

Metoden er tidligere afprøvet på voksne individer uden rapportering af bivirkninger. Desuden er den hurtig og ikke invasiv. Det vurderes derfor, at metoden er uden risici for forsøgspersonerne.

Multiple breath washout (MBW)

Der er ikke rapporteret alvorlige bivirkning ved MBW undersøgelsen. I en forundersøgelse oplevede nogle børn, at de blev udtørret i munden og hostede i afslutningen af undersøgelsen.

Spirometri med metacholin provokation

Der er ikke alvorlige bivirkninger ved spirometri eller metacholin undersøgelsen. Der kan opleves lettere vejrtrækningssymptomer. Hvis målingen viser, at et astmaanfald er på vej, standses undersøgelsen, og der gives straks astmamedicin.

FeNO-måling

FeNO målingen er helt ufarlig.

Nasalskrab

Undersøgelsen er ikke forbundet med nogen former for risici, men der kan forekomme ubehag i form af smerte og en gang imellem lidt næseblod når skrabet udføres.

Dual X-ray Absorptiometry

Undersøgelsen påfører barnet meget lille og ubetydelig stråledosis omkring 3-3,5 µSv svarende til baggrundsstråling på mindre end en dag[112] og er ikke forbundet med nogen former for ubehag eller risici.

Akustisk rhinometri

Undersøgelsen medfører kun et minimum af ubehag og ingen risiko for barnet.

Ultralydsscanning af fingerknoglerne

Undersøgelsen tager ca. 15 min og generer ikke barnet.

Halspodning

Undersøgelsen tager få sekunder og medfører kun et minimum af ubehag og ingen risiko for barnet.

<u>Tid</u>

Den største ulempe ved at deltage i dette projekt er, at der fra familiens side skal bruges tid på besøg på ABC-klinikken, dagbogsføring og prøveindsamlinger.

- Barnet med mindst én forælder skal besøge klinikken i alt 9 gange over 3 år, cirka 1 time hver gang.
- Dagbogen er i afkrydsningsform og skulle derfor tage ca. 1-2 minutter dagligt.
- Prøveindsamlinger af modermælk, afføring og støv vurderes til samlet at tage 1 time årligt.
- Planlagte besøg i fase 2 forventes at vare under 1 time for 4 og 5 års besøget og 2-3 timer for 6 års besøget.

Fordele for forsøgspersonerne

Familierne har mulighed for uvisiteret at blive set af en lille gruppe af læger med særlig interesse for pædiatri og dermatologi ved alle akutte luftvejsrelaterede sygdomme samt hudproblemer på alle hverdage. For almindelige familier er der ofte ventetid, og i yderområderne kan der være langt til speciallæge.
Hvis barnet får konstateret astma, atopisk dermatit, høfeber eller allergi – sygdomme som forsøgsdeltagerne er særligt disponerede til at udvikle – får deltagerne mulighed for behandling, vejledning og kontrol på specialistniveau. Også her er der tale om en lille gruppe af læger og sygeplejersker, hvilket sikrer kontinuiteten og øger trygheden hos børnene.

Etiske aspekter

Forskning med børn

Vi er fuldt ud bevidste om kravet til højeste etiske standard, når det drejer sig om studier med børn. Vores erfaringer fra COPSAC-studiet har været med til at forsikre om, at kliniske kohortestudier med børn kan udføres uden at deltagerne på nogen måde lider overlast, hverken i form af smerte, stress, frygt eller anden ubehag. Som nævnt er ABC-personalet særligt trænet til undersøgelser af børn. I afvejningen af ulempe-graden i fht til risikoen for sygdom, må man huske at deltagerne pga. arvelig disposition er i højere risiko end baggrundsbefolkningen for udvikling af atopiske lidelser.

Som nævnt har alle undersøgelser været anvendt under COPSAC-studiet og var forinden godkendt af VEK. I alt 16 protokoller for COPSAC er godkendt hos VEK (jf. bilag 2). Det er vigtigt at bemærke at ABC-kohorten er et mindre intensivt studie end COPSAC og undersøgelserne mindre invasive for projektdeltagerne.

De meget præmature børn (anslået 10 børn) samt gruppen af ekstremt præmature børn (anslået 4 børn) må forventes at kunne få problemer med lungemodningen og deraf muligvis være i højere risiko for at få infektioner og være mere sensible senere i livet. Gruppen af ekstremt præmature børn vil have den højeste risiko for at få senfølger af den tidlige fødsel. Dette vil naturligvis betyde flere lungesymptomer hos børnene, og derfor et større behov for at blive set af en læge. Da det samlede antal børn ikke anslås at være særlig stort, vurderer vi, at vi på ABC klinikken sagtens vil kunne overskue – og forsvare – også at følge disse børn tæt.

Forældre til for tidligt fødte børn kan have særlige behov for opfølgning fra sundhedsvæsenet de første år af livet, og mange forældre vil naturligt være bekymrede for, hvordan barnet trives og udvikler sig, og om det er alderssvarende. De forældre, der vælger at deltage i ABC projektet, og hvis børn er for tidligt fødte, vil i højere grad have mulighed for at blive fulgt tæt af interesserede læger, sammenlignet med forældre til børn der ikke deltager i ABC projektet. Ved den løbende kontakt til familierne vil det således være muligt at fokusere på, vejlede og behandle, såfremt der forekommer lungesymptomer fremkaldt af den tidlige fødsel, ligesom barnets udvikling, vækst og trivsel ligeledes vil blive løbende vurderet.
Forældre til meget tidligt fødte børn kan føle, at det er uoverskueligt at komme til undersøgelserne i ABC-klinikken, da den hjemlige situation måske i forvejen er belastet af den for tidlige fødsel. Hvis forældrene ønsker det, kan de naturligvis, til enhver tid vælge at trække sig ud af projektet uden at behøve at skulle forklare nærmere.
For nogle af de meget tidligt fødte børn kan det blive svært at gennemføre de første undersøgelser i ABC-projektet, da familierne ofte er indlagt på neonatal afdelinger indtil børnene har opnået en tilfredsstillende trivsel – i nogle tilfælde flere måneder. I disse tilfælde vil vi – i tæt dialog med forældrene – vurdere hvordan vi bedst muligt håndterer situationen. Hvis forældrene på dette tidspunkt vælger at gå ud af projektet kan de naturligvis det.

I COPSAC-studiet har de etiske aspekter i forskningsprojekter med børn været i fokus og vi har gennemført 2 studier, med analyse af netop de etiske aspekter. Studierne er udført uafhængigt af COPSAC's øvrige arbejde. Interviewene blev udført at Anne Gammelgaard, cand.scient., ph.d., Afdeling for Medicinsk Videnskabsteori, Institut for Folkesundhedsvidenskab, Københavns Universitet.
I den første undersøgelse deltog 23 mødre i semistrukturerede kvalitative interviews. De viste at den primære motivationsfaktor for deltagelse i COPSAC var altruisme, dvs. ønsket om at hjælpe andre med atopiske lidelser. En anden vigtig motivationsfaktor var muligheden for hurtigt? at få sit barn undersøgt af specialister. Endelig var det en udbredt holdning, at forældrene oplevede at deres børn var glade for besøgene, og at de desuden ville have trukket sig, hvis deres børn gav udtryk for ubehag ved projektet. Forældrenes mening var ikke afhængig af om deres børn var syge eller raske.[113, 114].
I en anden undersøgelse blev 21 projektdeltagere på 7 år interviewet om oplevelsen af at have deltaget i COPSAC, og konklusionen var at børnene følte at det havde været en positiv oplevelse[115].

Genetisk materiale

Vi kender endnu ikke de specifikke gener eller biokemiske markører, der spiller en rolle for udvikling af de atopiske sygdomme, og det er da netop også formålet med vores analyser at finde disse. Vi har derfor kun mulighed for at ansøge om samlet tilladelse til en GWA-scan, hvor vi udelukkende ser på genvariationer, hvis forekomst relateres til astma, allergi, eksem og luftvejssymptomer. I de tilfælde hvor vi finder kandidatgener vil det føre til analyse af biokemiske markører i andet indsamlet biologisk materiale, f.eks. plasma, urin, luftvejssekret eller navlesnorsblod.

Denne åbenhed omkring fremtidige analyser er en naturlig konsekvens af studiets formål: at identificere mulige prædiktorer for udviklingen af atopiske lidelser. Vi forventer at deltagerne accepterer denne usikkerhed i forhold til analyser, så længe vi udelukkende undersøger for relationer til astma, allergi, eksem og luftvejssymptomer.

Genetiske analyser udføres i anonymiseret form. CHOP har lang erfaring med disse analyser. Al genetisk materiale opbevares forsvarligt. Kun medarbejdere godkendt af den forskningsansvarlige på stedet har adgang til materialet. Efter GWA-analyserne er udført, destrueres eventuelt overskydende materiale som er afsendt til CHOP, således at der ikke ligger genmateriale i USA længere end højest nødvendigt.

Da genanalyserne er anonymiserede kender forskerne ikke det enkelt barns genetik, svarende til Good Clinical Practice. I tilfælde af, at vores forskning fører til fund af risikogener med betydning for forebyggelse og behandling af sygdom, vil det efter vores vurdering være uetisk ikke at lade familierne få adgang til de informationer, som de selv har medvirket til at tilvejebringe. I så fald vil interesserede familier kunne få information om deres barns genetiske status i den relevante sammenhæng. Dette gives ved samtale på ABC-klinikken, hvor der gives mundtlig og skriftlig information om betydningen af fundene for deres barn. Hvis det skulle blive aktuelt vil informationsmateriale blive sendt til godkendelse i VEK. Dette forhold vil blive fremlagt for forældrene ved informationssamtalen ved inklusionen. Interesserede familier kan allerede her give udtryk for om de ønsker at blive kontaktet i tilfælde af, at nye oplysninger dukker op.

Fiskeolie/olivenolie i sidste del af graviditeten

Da fiskeolie allerede tidligere er givet til gravide kvinder og det er vist at det ingen risici har medført for hverken barnet eller moderen, finder vi ikke at der er nogle etiske problemstillinger i forbindelse med dette projekt. De kvinder der randomiseres til fiskeolie opnår fordelen af en eventuel forebyggende effekt hos børnene. Dette er dog endnu ikke videnskabeligt verificeret (hvorfor vi netop ønsker at lave dette studie) og vi finder det ikke etisk problematisk at halvdelen af kvinderne får placebobehandling i form af olivenolie, da de blot gennemfører en normal graviditet.

Opbevaring af stamceller

Forældrene vil blive informeret skriftligt om opbevaring og undersøgelse af stamceller fra navlesnorsblodet. Stamcellerne vil udelukkende blive brugt til at fremstille celler fra immunforsvaret og vil ikke tjene andre formål end at undersøge tendensen til at udvikle atopisk sygdom.

Hvis barnet udvikler en alvorlig blodsygdom, som fx leukæmi, er det muligt, at man i fremtiden kan bruge de resterende stamceller til at behandle barnet.

Vurdering af den neurologiske udvikling

Ca. 0,5 % af befolkningen ligger ekstremt lavt, når man måler deres kognitive funktion. Overført til vores børnekohorte, kan man forvente, at ca. 3-4 af vores børn vil afvige i testen. Disse børn vil blive konfereret med Peter Uldall, professor i neuropædiatri på Rigshospitalet, og henvist til yderligere udredning. Vi er naturligvis klar over de etiske problemstillinger, der kan ligge i at teste så mange børn, men vi vurderer at vi med de aftaler, vi har gjort med Peter Uldall, kan tilbyde børnene den bedst mulige behandling. Desuden vil eventuelle udviklingsmæssige afvigelser blive opdaget tidligere, end hvis børnene ikke var med i projektet, hvilket giver børnene og deres familier mulighed for tidligere, og derved måske bedre, behandling.

Vi vurderer derfor, at der ikke vil være nogen etiske problemstillinger i forbindelse med dette del-projekt.

ABC studiets organisation

I Dansk BørneAstma Center (DBAC) har man gennem COPSAC studiet fået opbygget en ekspertise på internationalt plan inden for udførelse af kohortestudier.
Det er en veletableret forskergruppe placeret på Gentofte hospital. Gruppen ledes af en professor i pædiatri. Som udgangspunkt vil der blive ansat 4 læger som Ph.d. studerende ved Københavns Universitet med delstudier i ABC-kohorten, 2 læger under uddannelse til børnelæger, 1 under uddannelse til hudlæge og 1 under uddannelse til gynækolog. Herudover er 3 læger ansat ved COPSAC som Ph.d.-studerende, 3 forskningsassistenter holder kontakt med familierne og udfører de kliniske undersøgelser. Yderligere bemandes studiet med en projektkoordinator der forestår databehandling og kvalitetssikring samt en statistiker og en projektsekretær.

DBAC har gennem flere år haft tæt samarbejde med børneafdelingen på Næstved sygehus. Her vil en speciallæge i pædiatrisk pulmonologi og allergologi være ansvarlig for den daglige drift af ABC-klinikken. Undersøgelserne varetages af 2 Ph.d.-studerende læger og 1 forskningsassistent. Der vil være tæt kontakt til DBAC Gentofte. Hudlægen fra DBAC besøger Næstved én gang ugentligt. Alle data indtastes online i én fælles database.

Alle undersøgelser, diagnoser og behandling styres af DBAC's forskningslæger (dvs. ikke praktiserende læger eller lokale børneafdelinger). Derved minimeres variation i diagnostisk og behandlings tradition. Studiet er et to-center studie med standardiserede procedurer og metoder. Data management og kvalitetskontrol følger Good Clinical Practise standard med on-line data opsamling, låsning af data efter data validering, samt registrering af "audit trail".

Statistisk styrke

Prospektive klinisk-epidemiologiske kohortestudier som ABC-kohorten er meget ressourcekrævende sammenlignet med f.eks. spørgeskema-undersøgelser eller retrospektive interviews. Dette begrænser antallet af forsøgsdeltagere væsentligt. Til gengæld er kvaliteten af data langt højere.[116, 117]Fødselskohorte studier er anses for at være 'guld standard' indenfor forskning i

tidlig astma, allergi og eksem. Kun randomiserede interventionsforsøg har højere validitet[56], men dette er sjældent gennemførligt eller etisk forsvarligt hos børn.
Hovedparten af lignende kohortestudier har været på cirka 200 – 1400 børn[118-123].
I COPSAC inkluderede vi 400, hvilket var et antal med statistisk styrke til at vise signifikante resultater. Dog blev vi i flere tilfælde begrænset af manglende statistisk styrke, når vi ønskede at lave subgruppeanalyser. Som nævnt er netop samspillet diverse genetiske og miljømæssige faktorer i fokus i ABC-studiet, hvilket gør det yderligere interessant at kunne udføre sådanne undersøgelser. Ved at inkludere det dobbelte antal kohortedeltagere øges den statistiske power og dermed muligheden for detaljerede studier med flere facetter. Ideelt set kunne vi ønske flere deltagere, men det ville indebære at gå på kompromis med datakvalitet i forhold til de ressourcer, vi har til rådighed.

Fiskeolie
Vi antager at den sande odds ratio, på baggrund af eksponering, er 0,5 og at sygdomsprævalensen er 15 % i en uselekteret kohorte.
I et 1:1 uparret case-control studie vil 337 cases (fiskeolie) og 337 kontroller (placebo) være nødvendige for at kunne detektere en effekt af eksponering med 80 % chance ved et 5 % signifikansniveau (normal approximation test). Efter hensyntagen til eventuelle drop-outs og lign. er en kohorte med 800 personer således stadig passende.

Publiceringsstrategi

Resultater for ABC-studiet forventes publiceret i internationale tidsskrifter med højeste impact. Der gennemføres løbende Ph.d.-studier som led i Dansk BørneAstma Centers forskningsprojekter. Let forståelige resumeer af resultaterne vil være tilgængelige på Dansk BørneAstma Centers website: www.dbac.dk

Samarbejde i Danmark

Alle mikrobiologiske analyser udføres på Statens Serum Institut (SSI) under vejledning af professor Karen Kroghfeldt, der er medansvarlig for den mikrobiologiske del af studiet.

Analyse af næsefilterpapir samt stamceller i navlesnorsblod foregår på Systems Biology of Immune Regulation, DTU under vejledning af lektor Susanne Brix Pedersen.

Økonomisk støtte

ABC-studiet udføres under Dansk BørneAstma Center, som støttes af offentlige midler samt private fonde. Hovedfinansieringen til dette projekt baseres på en bevilling til DBAC fra Lundbeckfonden på 20 mio. kr. til perioden 2008 - 2012.

Som følge af tildelingen af de 20 millioner kroner etableres ”Center for Translationel forskning i gen-miljø interaktion ved astma, eksem og allergi”. Tildelingen er sket som følge af, at Lundbeckfonden i 2007 og 2008 støtter etablering af forskningscentre, hvis sigte er at bringe resultaterne af den grundvidenskabelige forskning hurtigere og mere effektivt i anvendelse til gavn for patienterne.
Lundbeckfonden er en erhvervsdrivende fond. Afkastet af fondens formue anvendes bl.a. til støtte af videnskabelig forskning indenfor sundheds- og naturvidenskaberne.

Herudover modtager Dansk Børne Astma Center årligt støtte på finansloven. Der er netop indgået aftale om 5 millioner kr. årligt i perioden 2008 - 2011.

Endvidere har det tidligere Københavns amts forskningsfond givet tilsagn om støtte, det endelig beløb og andel til ABC-kohorten er endnu ikke fastlagt.

Der laves desuden løbende fundraising i forbindelse med de enkelte delstudier.

Fremtidige undersøgelser

Forskningen indenfor astma, allergi og eksem er i disse årtier i hastig udvikling. Det er derfor vores forventning, at der i løbet af kohortens levetid vil dukke nye interessante emner op. Det er styrken ved en fødselskohorte med bred dataindsamling, at vi har mulighed for hurtigt og uden belastning af patienter at supplere vores hypoteser med nye delmål. I tilfælde, hvor der skal udføres andre undersøgelser eller biologiske analyser end de, som er anfør i denne protokol, vil vi naturligvis ansøge VEK om ny tilladelse.

Reference Liste

1. Kocevar VS, Bisgaard H, Jonsson L et al. Variations in pediatric asthma hospitalization rates and costs between and within Nordic countries. Chest 2004; 125(5):1680-1684.

2. Worldwide variation in prevalence of symptoms of asthma, allergic rhinoconjunctivitis, and atopic eczema: ISAAC. The International Study of Asthma and Allergies in Childhood (ISAAC) Steering Committee. Lancet 1998; 351(9111):1225-1232.

3. Holgate ST. The epidemic of allergy and asthma. Nature 1999; 402(6760 Suppl):B2-B4.

4. Halkjaer LB, Loland L, Buchvald FF et al. Development of atopic dermatitis during the first 3 years of life: the Copenhagen prospective study on asthma in childhood cohort study in high-risk children. Arch Dermatol 2006; 142(5):561-566.

5. Xu B, Pekkanen J, Jarvelin MR, Olsen P, Hartikainen AL. Maternal infections in pregnancy and the development of asthma among offspring. Int J Epidemiol 1999; 28(4):723-727.

6. Nafstad P, Magnus P, Jaakkola JJ. Risk of childhood asthma and allergic rhinitis in relation to pregnancy complications. J Allergy Clin Immunol 2000; 106(5):867-873.

7. McKeever TM, Lewis SA, Smith C, Hubbard R. Mode of delivery and risk of developing allergic disease. J Allergy Clin Immunol 2002; 109(5):800-802.

8. Bager P, Melbye M, Rostgaard K, Benn CS, Westergaard T. Mode of delivery and risk of allergic rhinitis and asthma. J Allergy Clin Immunol 2003; 111(1):51-56.

9. Barker DJ, Godfrey KM, Fall C, Osmond C, Winter PD, Shaheen SO. Relation of birth weight and childhood respiratory infection to adult lung function and death from chronic obstructive airways disease. BMJ 1991; 303(6804):671-675.

10. McKeever TM, Lewis SA, Smith C et al. Early exposure to infections and antibiotics and the incidence of allergic disease: a birth cohort study with the West Midlands General Practice Research Database. J Allergy Clin Immunol 2002; 109(1):43-50.

11. Mielck A, Reitmeir P, Wjst M. Severity of childhood asthma by socioeconomic status. Int J Epidemiol 1996; 25(2):388-393.

12. Bisgaard H, Dalgaard P, Nyboe J. Risk factors for wheezing during infancy. A study of 5,953 infants. Acta Paediatr Scand 1987; 76(5):719-726.

13. Strachan DP. Family size, infection and atopy: the first decade of the "hygiene hypothesis". Thorax 2000; 55 Suppl 1:S2-10.

14. Remes ST, Castro-Rodriguez JA, Holberg CJ, Martinez FD, Wright AL. Dog exposure in infancy decreases the subsequent risk of frequent wheeze but not of atopy. J Allergy Clin Immunol 2001; 108(4):509-515.

15. Wright AL, Holberg CJ, Taussig LM, Martinez FD. Factors influencing the relation of infant feeding to asthma and recurrent wheeze in childhood. Thorax 2001; 56(3):192-197.

16. Sears MR, Greene JM, Willan AR et al. Long-term relation between breastfeeding and development of atopy and asthma in children and young adults: a longitudinal study. Lancet 2002; 360(9337):901-907.

17. Johnston SL, Pattemore PK, Sanderson G et al. Community study of role of viral infections in exacerbations of asthma in 9-11 year old children. BMJ 1995; 310(6989):1225-1229.

18. Sigurs N. Epidemiologic and clinical evidence of a respiratory syncytial virus-reactive airway disease link. Am J Respir Crit Care Med 2001; 163(3 Pt 2):S2-S6.

19. Li JS, Peat JK, Xuan W, Berry G. Meta-analysis on the association between environmental tobacco smoke (ETS) exposure and the prevalence of lower respiratory tract infection in early childhood. Pediatr Pulmonol 1999; 27(1):5-13.

20. Nafstad P, Kongerud J, Botten G, Hagen JA, Jaakkola JJ. The role of passive smoking in the development of bronchial obstruction during the first 2 years of life. Epidemiology 1997; 8(3):293-297.

21. Peat JK, Keena V, Harakeh Z, Marks G. Parental smoking and respiratory tract infections in children. Paediatr Respir Rev 2001; 2(3):207-213.

22. Stein RT, Holberg CJ, Sherrill D et al. Influence of parental smoking on respiratory symptoms during the first decade of life: the Tucson Children's Respiratory Study. Am J Epidemiol 1999; 149(11):1030-1037.

23. Cook DG, Strachan DP. Health effects of passive smoking-10: Summary of effects of parental smoking on the respiratory health of children and implications for research. Thorax 1999; 54(4):357-366.

24. Kulig M, Luck W, Lau S et al. Effect of pre- and postnatal tobacco smoke exposure on specific sensitization to food and inhalant allergens during the first 3 years of life. Multicenter Allergy Study Group, Germany. Allergy 1999; 54(3):220-228.

25. Palmer CN, Irvine AD, Terron-Kwiatkowski A et al. Common loss-of-function variants of the epidermal barrier protein filaggrin are a major predisposing factor for atopic dermatitis. Nat Genet 2006; 38(4):441-446.

26. Bisgaard H, Halkjaer LB, Loland L, Palmer C, Silveira L. Dog Exposure Protects against Atopic Dermatitis in Early Childhood. 2008.
Ref Type: Unpublished Work

27. Bisgaard H, et al. Gene-Environment Interaction in the Penetrance of Eczema in Infancy Replicated in Two Birth-Cohort Studies:
Filaggrin Loss-of-Function Mutations Triggered by Cat Exposure. 2008.
Ref Type: Unpublished Work

28. Bisgaard H, Hermansen MN, Buchvald F et al. Childhood asthma after bacterial colonization of the airway in neonates. N Engl J Med 2007; 357(15):1487-1495.

29. Bisgaard H. The Copenhagen Prospective Study on Asthma in Childhood (COPSAC): design, rationale, and baseline data from a longitudinal birth cohort study. Ann Allergy Asthma Immunol 2004; 93(4):381-389.

30. Strachan D. Hay fever, Hygeine, and household size. BMJ 299, 1259-1266. 1989.
Ref Type: Generic

31. Martinez FD, Holt PG. Role of microbial burden in aetiology of allergy and asthma. Lancet 1999; 354 Suppl 2:SII12-SII15.

32. Liu AH, Murphy JR. Hygiene hypothesis: fact or fiction? J Allergy Clin Immunol 2003; 111(3):471-478.

33. Bjorksten B. Allergy priming early in life. Lancet 1999; 353(9148):167-168.

34. Price JF. Acute and long-term effects of viral bronchiolitis in infancy. Lung 1990; 168 Suppl:414-421.

35. Papadopoulos NG, Kalobatsou A. Respiratory viruses in childhood asthma. Curr Opin Allergy Clin Immunol 2007; 7(1):91-95.

36. Yoo J, Tcheurekdjian H, Lynch SV, Cabana M, Boushey HA. Microbial manipulation of immune function for asthma prevention: inferences from clinical trials. Proc Am Thorac Soc 2007; 4(3):277-282.

37. Candi E, Schmidt R, Melino G. The cornified envelope: a model of cell death in the skin. Nat Rev Mol Cell Biol 2005; 6(4):328-340.

38. Lerbaek A, Bisgaard H, Agner T, Ohm KK, Palmer CN, Menne T. Filaggrin null alleles are not associated with hand eczema or contact allergy. Br J Dermatol 2007; 157(6):1199-1204.

39. Weidinger S, Illig T, Baurecht H et al. Loss-of-function variations within the filaggrin gene predispose for atopic dermatitis with allergic sensitizations. J Allergy Clin Immunol 2006; 118(1):214-219.

40. Marenholz I, Nickel R, Ruschendorf F et al. Filaggrin loss-of-function mutations predispose to phenotypes involved in the atopic march. J Allergy Clin Immunol 2006; 118(4):866-871.

41. Lau S, Illi S, Sommerfeld C et al. Early exposure to house-dust mite and cat allergens and development of childhood asthma: a cohort study. Multicentre Allergy Study Group. Lancet 2000; 356(9239):1392-1397.

42. Ownby DR, Johnson CC, Peterson EL. Exposure to dogs and cats in the first year of life and risk of allergic sensitization at 6 to 7 years of age. JAMA 2002; 288(8):963-972.

43. Uldall P. Spæd- og småbørns almindelige sygelighed. 1-12-1986. 1986.

44. Nyboe A. Børns sundhed og sygelighed. Sundheds- og sygelighedsundersøgelsen, SFI. 2005.

45. Illi S, von ME, Lau S et al. Early childhood infectious diseases and the development of asthma up to school age: a birth cohort study. BMJ 2001; 322(7283):390-395.

46. Koefoed B, Nielsen A, Keiding L. Udvalgte miljøfaktorers betydning for børns sygelighed i daginstitutioner. Ugeskr.Laeger 164, 5759. 2002.
Ref Type: Generic

47. Calder PC. omega 3 polyunsaturated fatty acids, inflammation and immunity. World Rev Nutr Diet 2001; 88:109-116.

48. Calder PC. N-3 polyunsaturated fatty acids and inflammation: from molecular biology to the clinic. Lipids 2003; 38(4):343-352.

49. Blumer N, Renz H. Consumption of omega3-fatty acids during perinatal life: role in immuno-modulation and allergy prevention. J Perinat Med 2007; 35 Suppl 1:S12-S18.

50. Magnan AO, Mely LG, Camilla CA et al. Assessment of the Th1/Th2 paradigm in whole blood in atopy and asthma. Increased IFN-gamma-producing CD8(+) T cells in asthma. Am J Respir Crit Care Med 2000; 161(6):1790-1796.

51. van Oosterhout AJ, Bloksma N. Regulatory T-lymphocytes in asthma. Eur Respir J 2005; 26(5):918-932.

52. Denburg JA, Hatfield HM, Cyr MM et al. Fish oil supplementation in pregnancy modifies neonatal progenitors at birth in infants at risk of atopy. Pediatr Res 2005; 57(2):276-281.

53. Dunstan JA, Mori TA, Barden A et al. Fish oil supplementation in pregnancy modifies neonatal allergen-specific immune responses and clinical outcomes in infants at high risk of atopy: a randomized, controlled trial. J Allergy Clin Immunol 2003; 112(6):1178-1184.

54. Mihrshahi S, Peat JK, Webb K, Oddy W, Marks GB, Mellis CM. Effect of omega-3 fatty acid concentrations in plasma on symptoms of asthma at 18 months of age. Pediatr Allergy Immunol 2004; 15(6):517-522.

55. Mihrshahi S, Peat JK, Marks GB et al. Eighteen-month outcomes of house dust mite avoidance and dietary fatty acid modification in the Childhood Asthma Prevention Study (CAPS). J Allergy Clin Immunol 2003; 111(1):162-168.

56. Olsen SF, Osterdal ML, Salvig JD et al. Fish oil intake compared with olive oil intake in late pregnancy and asthma in the offspring: 16 y of registry-based follow-up from a randomized controlled trial. Am J Clin Nutr 2008; 88(1):167-175.

57. Prescott SL, Calder PC. N-3 polyunsaturated fatty acids and allergic disease. Curr Opin Clin Nutr Metab Care 2004; 7(2):123-129.

58. Olsen SF, Osterdal ML, Salvig JD et al. Fish oil intake compared with olive oil intake in late pregnancy and asthma in the offspring: 16 y of registry-based follow-up from a randomized controlled trial. Am J Clin Nutr 2008; 88(1):167-175.

59. Olsen SF, Sorensen JD, Secher NJ et al. Randomised controlled trial of effect of fish-oil supplementation on pregnancy duration. Lancet 1992; 339(8800):1003-1007.

60. Olsen SF. Is supplementation with marine omega-3 fatty acids during pregnancy a useful tool in the prevention of preterm birth? Clin Obstet Gynecol 2004; 47(4):768-774.

61. Strachan DP. Hay fever, hygiene, and household size. BMJ 1989; 299(6710):1259-1260.

62. Liu AH, Murphy JR. Hygiene hypothesis: fact or fiction? J Allergy Clin Immunol 2003; 111(3):471-478.

63. Martinez FD, Holt PG. Role of microbial burden in aetiology of allergy and asthma. Lancet 1999; 354 Suppl 2:SII12-SII15.

64. Bisgaard H, Hermansen MN, Buchvald F et al. Childhood asthma after bacterial colonization of the airway in neonates. N Engl J Med 2007; 357(15):1487-1495.

65. Howarth PH, Persson CG, Meltzer EO, Jacobson MR, Durham SR, Silkoff PE. Objective monitoring of nasal airway inflammation in rhinitis. J Allergy Clin Immunol 2005; 115(3 Suppl 1):S414-S441.

66. Alam R, Sim TC, Hilsmeier K, Grant JA. Development of a new technique for recovery of cytokines from inflammatory sites in situ. J Immunol Methods 1992; 155(1):25-29.

67. Erin EM, Zacharasiewicz AS, Nicholson GC et al. Topical corticosteroid inhibits interleukin-4, -5 and -13 in nasal secretions following allergen challenge. Clin Exp Allergy 2005; 35(12):1608-1614.

68. Andersen HB, Holm M, Hetland TE et al. Comparison of short term in vitro cultured human mast cells from different progenitors - Peripheral blood-derived progenitors generate highly mature and functional mast cells. J Immunol Methods 2008; 336(2):166-174.

69. Theoharides TC, Kempuraj D, Tagen M, Vasiadi M, Cetrulo CL. Human umbilical cord blood-derived mast cells: a unique model for the study of neuro-immuno-endocrine interactions. Stem Cell Rev 2006; 2(2):143-154.

70. Anderson GP. Endotyping asthma: new insights into key pathogenic mechanisms in a complex, heterogeneous disease. Lancet 2008; 372(9643):1107-1119.

71. Dragonieri S, Schot R, Mertens BJ et al. An electronic nose in the discrimination of patients with asthma and controls. J Allergy Clin Immunol 2007; 120(4):856-862.

72. Leuppi JD, Salome CM, Jenkins CR et al. Markers of airway inflammation and airway hyperresponsiveness in patients with well-controlled asthma. Eur Respir J 2001; 18(3):444-450.

73. Strunk RC, Szefler SJ, Phillips BR et al. Relationship of exhaled nitric oxide to clinical and inflammatory markers of persistent asthma in children. J Allergy Clin Immunol 2003; 112(5):883-892.

74. Sigurs N, Aljassim F, Kjellman B et al. Asthma and allergy patterns over 18 years after severe RSV bronchiolitis in the first year of life. Thorax 2010; 65(12):1045-1052.

75. Macklem PT. The physiology of small airways. Am J Respir Crit Care Med 1998; 157(5 Pt 2):S181-S183.

76. Gustafsson PM. Inert gas washout in preschool children. Paediatr Respir Rev 2005; 6(4):239-245.

77. Macleod KA, Horsley AR, Bell NJ, Greening AP, Innes JA, Cunningham S. Ventilation heterogeneity in children with well controlled asthma with normal spirometry indicates residual airways disease. Thorax 2009; 64(1):33-37.

78. Gustafsson PM, Aurora P, Lindblad A. Evaluation of ventilation maldistribution as an early indicator of lung disease in children with cystic fibrosis. Eur Respir J 2003; 22(6):972-979.

79. Lauritzen L, Hoppe C, Straarup EM, Michaelsen KF. Maternal fish oil supplementation in lactation and growth during the first 2.5 years of life. Pediatr Res 2005; 58(2):235-242.

80. Javaid MK, Crozier SR, Harvey NC et al. Maternal vitamin D status during pregnancy and childhood bone mass at age 9 years: a longitudinal study. Lancet 2006; 367(9504):36-43.

81. Chomtho S, Wells JC, Williams JE, Davies PS, Lucas A, Fewtrell MS. Infant growth and later body composition: evidence from the 4-component model. Am J Clin Nutr 2008; 87(6):1776-1784.

82. Stettler N, Zemel BS, Kumanyika S, Stallings VA. Infant weight gain and childhood overweight status in a multicenter, cohort study. Pediatrics 2002; 109(2):194-199.

83. Javaid MK, Cooper C. Prenatal and childhood influences on osteoporosis. Best Pract Res Clin Endocrinol Metab 2002; 16(2):349-367.

84. Carrie Fassler AL, Bonjour JP. Osteoporosis as a pediatric problem. Pediatr Clin North Am 1995; 42(4):811-824.

85. Eisman JA, Kelly PJ, Morrison NA et al. Peak bone mass and osteoporosis prevention. Osteoporos Int 1993; 3 Suppl 1:56-60.

86. Ciprandi G, Cirillo I, Pistorio A. Impact of allergic rhinitis on asthma: effects on spirometric parameters. Allergy 2008; 63(3):255-260.

87. Sears MR, Greene JM, Willan AR et al. A longitudinal, population-based, cohort study of childhood asthma followed to adulthood. N Engl J Med 2003; 349(15):1414-1422.

88. Gallupundersøgelse lavet for FTF. Børns sygefravær og forældrenes muligheder, intervew af 1028 forældre til børn under 16 år. 2006.
Ref Type: Generic

89. Deding M, Lausten M, Andersen A. Børnefamiliernes balance mellem famile og arbejdsliv. Socialforskningsinstituttet (SFI) . 7-12-2006.
Ref Type: Generic

90. Gallupundersøgelse lavet for FTF. Børns sygefravær og forældrenes muligheder, intervew af 1028 forældre til børn under 16 år. 2006.
Ref Type: Generic

91. Tariq SM, Matthews SM, Hakim EA, Stevens M, Arshad SH, Hide DW. The prevalence of and risk factors for atopy in early childhood: a whole population birth cohort study. J Allergy Clin Immunol 1998; 101(5):587-593.

92. Zeiger RS, Heller S. The development and prediction of atopy in high-risk children: follow-up at age seven years in a prospective randomized study of combined maternal and infant food allergen avoidance. J Allergy Clin Immunol 1995; 95(6):1179-1190.

93. Dold S, Wjst M, von ME, Reitmeir P, Stiepel E. Genetic risk for asthma, allergic rhinitis, and atopic dermatitis. Arch Dis Child 1992; 67(8):1018-1022.

94. Bohme M, Wickman M, Lennart NS, Svartengren M, Wahlgren CF. Family history and risk of atopic dermatitis in children up to 4 years. Clin Exp Allergy 2003; 33(9):1226-1231.

95. Olsen SF, Osterdal ML, Salvig JD et al. Fish oil intake compared with olive oil intake in late pregnancy and asthma in the offspring: 16 y of registry-based follow-up from a randomized controlled trial. Am J Clin Nutr 2008; 88(1):167-175.

96. Schibler A, Schneider M, Frey U, Kraemer R. Moment ratio analysis of multiple breath nitrogen washout in infants with lung disease. Eur Respir J 2000; 15(6):1094-1101.

97. Latzin P, Thamrin C, Kraemer R. Ventilation inhomogeneities assessed by the multibreath washout (MBW) technique. Thorax 2008; 63(2):98-99.

98. Verbanck S, Schuermans D, Van MA, Paiva M, Noppen M, Vincken W. Ventilation distribution during histamine provocation. J Appl Physiol 1997; 83(6):1907-1916.

99. Robinson PD, Goldman MD, Gustafsson PM. Inert gas washout: theoretical background and clinical utility in respiratory disease. Respiration 2009; 78(3):339-355.

100. Horsley AR, Gustafsson PM, Macleod KA et al. Lung clearance index is a sensitive, repeatable and practical measure of airways disease in adults with cystic fibrosis. Thorax 2008; 63(2):135-140.

101. Aurora P, Kozlowska W, Stocks J. Gas mixing efficiency from birth to adulthood measured by multiple-breath washout. Respir Physiol Neurobiol 2005; 148(1-2):125-139.

102. Klug B, Bisgaard H. Measurement of the specific airway resistance by plethysmography in young children accompanied by an adult. Eur Respir J 1997; 10(7):1599-1605.

103. Lowe L, Murray CS, Custovic A, Simpson BM, Kissen PM, Woodcock A. Specific airway resistance in 3-year-old children: a prospective cohort study. Lancet 2002; 359(9321):1904-1908.

104. Bisgaard H, Nielsen KG. Plethysmographic measurements of specific airway resistance in young children. Chest 2005; 128(1):355-362.

105. Poorisrisak P, Vrang C, Henriksen JM, Klug B, Hanel B, Bisgaard H. Accuracy of whole-body plethysmography requires biological calibration. Chest 2009; 135(6):1476-1480.

106. Klug B, Bisgaard H. Lung function and short-term outcome in young asthmatic children. Eur Respir J 1999; 14(5):1185-1189.

107. Pietrobelli A, Formica C, Wang Z, Heymsfield SB. Dual-energy X-ray absorptiometry body composition model: review of physical concepts. Am J Physiol 1996; 271(6 Pt 1):E941-E951.

108. Horlick M, Wang J, Pierson RN, Jr., Thornton JC. Prediction models for evaluation of total-body bone mass with dual-energy X-ray absorptiometry among children and adolescents. Pediatrics 2004; 114(3):e337-e345.

109. Chawes BL, Kreiner-Moller E, Bisgaard H. Objective assessments of allergic and nonallergic rhinitis in young children. Allergy 2009; 64(10):1547-1553.

110. Engel S, Tronhjem KM, Hellgren LI, Michaelsen KF, Lauritzen L. Docosahexaenoic acid status at 9 months is inversely associated with communicative skills in 3-year-old girls. Matern Child Nutr 2012.

111. Pedersen SS. Trakealsugning (Tracheal intubation). Ugeskr Laeger 2001; 163(50):7078-7079.

112. Thomas SR, Kalkwarf HJ, Buckley DD, Heubi JE. Effective dose of dual-energy X-ray absorptiometry scans in children as a function of age. J Clin Densitom 2005; 8(4):415-422.

113. Gammelgaard A, Knudsen LE, Bisgaard H. Perceptions of parents on the participation of their infants in clinical research. Arch Dis Child 2006; 91(12):977-980.

114. Gammelgaard A, Knudsen LE, Bisgaard H. Perceptions of parents on the participation of their infants in clinical research. Arch Dis Child 2006; 91(12):977-980.

115. Gammelgaard A, Bisgaard H. Evidence-based medicines for children: ethical aspects. Eur Respir J 2007; 29(4):821-822.

116. Hennekens CH, Buring JE. Design Strategies. Epidemiology in Medicine. 1987.

117. Gøtzsche P, Wulff H. Kritisk læsning af tidskriftartikler. Rationel Klinik. 1997.

118. Taussig LM, Wright AL, Holberg CJ, Halonen M, Morgan WJ, Martinez FD. Tucson Children's Respiratory Study: 1980 to present. J Allergy Clin Immunol 2003; 111(4):661-675.

119. Le SP. Prediction of asthma in children at 6 and 12 years of age: Perth infant asthma follow-up study. Pediatr Allergy Immunol 2002; 13 Suppl 15:44-46.

120. Arshad SH, Tariq SM, Matthews S, Hakim E. Sensitization to common allergens and its association with allergic disorders at age 4 years: a whole population birth cohort study. Pediatrics 2001; 108(2):E33.

121. Bergmann RL, Bergmann KE, Lau-Schadensdorf S et al. Atopic diseases in infancy. The German multicenter atopy study (MAS-90). Pediatr Allergy Immunol 1994; 5(6 Suppl):19-25.

122. Gold DR, Burge HA, Carey V, Milton DK, Platts-Mills T, Weiss ST. Predictors of repeated wheeze in the first year of life: the relative roles of cockroach, birth weight, acute lower respiratory illness, and maternal smoking. Am J Respir Crit Care Med 1999; 160(1):227-236.

123. Custovic A, Simpson BM, Murray CS, Lowe L, Woodcock A. The National Asthma Campaign Manchester Asthma and Allergy Study. Pediatr Allergy Immunol 2002; 13 Suppl 15:32-37.

Bilag 1: Oversigt over undersøgelser; fase 1

**Børn**	**24. uge**	**36. uge**	**1 uge**	**1 mdr**	**3 mdr**	**6 mdr**	**1 år**	**18 mdr**	**2 år**	**2,5 år**	**3 år**	**Undersøgelser af biologisk materiale**
Navlesnorsblod			x									IgE, IgM, D-vit. Immunforsvarets celler
Dagbog			x	x	x	x	x	x	x	x	x	
Milepæle			x	x	x	x	x	x	x			
Objektiv undersøgelse			x	x	x	x	x	x	x	x	x	
Vækst			x	x	x	x	x	x	x	x	x	
Luftvejssug			x	x	x							Mikrobiologisk kolonisering
Afføringsprøve			x	x	x							Mikrobiologisk kolonisering
Næsefilterpapir			x						x			Immunforsvaret
Elektronisk næse				x				x				Udåndingsluften
Urinprøve				x			x					Interleukiner, leukotriener
Blodprøve						x		x				GWA-scan, gener med relation til atopi
Priktest						x		x				Allergi
Støvprøve						x						
Tympanometri							x		x		x	
Hårprøve							x		x		x	Cotinin (nikotin-produkt)
Hudpodning							x					Mikrobiologisk kolonisering
Sprogtest							x		x			
Aktivitetsmåling									x			
Kognitiv test (Bailey)										x		
UL af fingerknogler											x	Knogledensiteten
**Forældre**												
Modermælk			x									Fedtsyresammensætning, D-vitamin
Vaginal podning	x	x										Mikrobiologisk kolonisering
Blodprøve	x		x									GWA-scan, gener med relation til atopi

Højde	x											
Interview om ekspositioner			x				x		x		x	

For børn med astmatiske symptomer uden behandlingsrespons

- Røntgen af lunger
- Blodprøve til undersøgelse for cystisk fibrose

Oversigt over undersøgelser; fase 2:

Børn	4 år	5 år	6 år	Undersøgelser af biologisk materiale
Dagbog for luftvej + eksem	x	x	x	
Undersøgelse af læge			x	
Tympanometri (trykmåling i øret)			x	
Blodtryk			x	
Vægt og højdemåling	x	x	x	
Næsefilterpapir			x	Immunforsvaret
Elektronisk næse			x	Udåndingsluften
Urinprøve			x	Interleukiner, leukotriener og metaboliske produkter
Blodprøve			x	Immunforsvar, epigenetik og metaboliske produkter
Priktest			x	Allergi
Hårprøve			x	Cotinin (nikotin-produkt)
Afføringsprøve	x	x	x	Mikrobiologisk kolonisering
Hudpodning			x	Mikrobiologisk kolonisering
Halspodning			x	Mikrobiologisk kolonisering
Nasalskrab*			x	mRNA - genekspression
Aktivitetsmåling			x	
DXA-scanning			x	Knogledensitet
Spirometri*			x	
Bodybox	x		x	
Multiple Breath Washout	x		x	
Metacholin provokation*			x	
FeNO måling*			x	
CBCL-skema*			x	Child Behaviour Checklist, mentalitet og adfærd
**Forældre**				
Interview om barnets miljø.	x	x	x	
Interview om astma og allergi hos barnet.	x	x	x	

Nye undersøgelser

Bilag 2. Godkendte VEK-protokoller i COPSAC

Journal nr		Godkendt
(HKF)	01-289/96	01-04-2008
(KF)	11-308923	13-06-2006
(KF)	11-270698	11-07-2005
(KF)	11-260071	18-03-2005
(KF)	12-158/03	13-01-2004
(KF)	11-152/03	30-12-2003
(KF)	11-092/03	18-08-2003
(KF)	07-00-058/02	29-01-2003
(KF)	11-107/02	29-11-2002
(KF)	12-061/02	02-05-2002
(KF)	12-095/01	09-09-2001
(KF)	11-115/99	25-10-1999
(KF)	11-037/99	17-05-1999
(KF)	02-118/98	22-09-1998
(KF)	01-178/98	26-08-1998
(KF)	01-277/97	09-09-1997
(KF)	01-289/96	24-10-1996

Bilag 3: Almindelige blodprøve-analyser

Udover Genome Wide Analysis undersøges:

Hæmatologisk status: Hæmoglobin, erytrocytter, MCV, MCHC, transferin, haptoglobin, trombocytter

Infektionstal: leucocytter, differentialtælling, CRP

Immunologisk status: Immunoglobulin A, G, M, E, orosomucoid, alfa-1-antitrypsin

Endokrinologisk status: Triglycerid, Cholesterol, Hemoglobin A1C, D-vitamin.

Bilag 4: Skema til registrering af milepæle

Milepæle registering

Milepæle	Dato
Løfter hoved til 45 grader (liggende på maven)	
Smiler spontant	
Griner	
Sidder med støtte (stabilt hoved)	
Løfter overkroppen fra underlaget og hviler på albuer	
Ruller fra ryg til mave og/eller mave til ryg	
Sidder uden støtte*	
Står med støtte*	
Tommelfinger(pincet) greb	
Kravler (på alle 4)*	
Står uden støtte (i 10 sekunder)*	
Går med hjælp*	
Går uden hjælp*	

* forklaring nedenfor

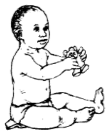

Sidder uden støtte

Barnet sidder med hovedet opret i mindst 10 sekunder uden at bruge arme eller hænder til at balancere kroppen eller sig selv.

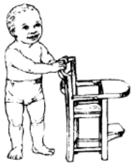

Står med støtte

Barnet står i oprejst stilling på begge fødder og holder på et stabilt objekt (f. eks møbler) med begge hænder uden at læne sig up ad det. Kroppen rører ikke det stabile objekt, og benene støtter det meste af kroppens vægt. Barnet står med støtte i mindst 10 sekunder.

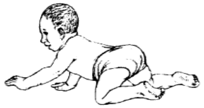

Kravler på alle 4

Barnet bevæger sig skiftevis fremad eller baglæns oppe på hænder og knæ. Maven rører ikke underlaget. Der er løbende og sammenhængende bevægelser mindst 3 i træk.

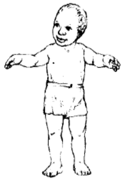

Står uden støtte

Barnet står i oprejst stilling på begge fødder (ikke tæer) med ryggen lige. Benene støtter 100% af barnets vægt. Der er ingen kontakt med en person eller objekt. Barnet står alene i mindst 10 sekunder.

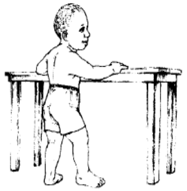

Går med hjælp

Barnet er i oprejst stilling med lige ryg. Barnet går sidelæns eller fremad ved at holde på et stabilt objekt (f. eks møbler) med en eller begge hænder. Et ben bevæger sig fremad, mens den anden støtter en del af kroppens vægt. Barnet tager mindst fem skridt på denne måde.

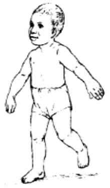

Går uden hjælp

Barnet tager mindst fem skridt selsvændigt i opret stilling med lige ryg. Et ben bevæger sig fremad, mens det andet støtter det meste af vægten. Der er ingen kontakt med person eller objekt.

Bilag 5: Informationsbrev om MacArthur sprogtest

Asthma Begins in Childhood

Gentofte 18-11-2010

Kære ABC-forældre

En del undersøgelser viser at fiskeolieindtag under graviditeten har indflydelse på barnets udvikling. Derfor har vi valgt at indføre nogle nye undersøgelser som vurderer jeres barns udvikling. I den forbindelse har mange af jer allerede fået udleveret et skema til dagbogen som hedder "milepæle".

Udover denne registrering vil vi gerne undersøge børnenes sprogudvikling. Vi bruger en database "Tidlig sprogvurdering" som er baseret på en dansk tilpasning af den amerikanske MacArthur Bates Communicative Development Inventory og er tilgængelig via et webinterface (cdi.sprogvurdering.dk).

Vi vil bede jer udfylde testen hurtigst muligt. I skal bare gå ind på hjemmesiden cdi.sprogvurdering.dk og logge på med jeres bruger id samt password, som er vedlagt dette brev.
Testen er udformet som afkrydsningsliste hvor I skal markere hvilke ord jeres barn forstår eller de ord, jeres barn både forstår og siger. Skemaet indeholder også en afkrydsningsliste om den første ordforståelse og tegngivning. Dette forklares nærmere, når i har logget ind.

Det er vigtigt at hele skemaet udfyldes på én gang, da det skal afspejle hvad jeres barn siger på et bestemt tidspunkt, og ikke over en længere periode.

Det er desuden vigtigt, at I udfylder listen hurtigst muligt, fordi vurderingen af børnenes sprogudvikling skal være så tæt på et/to års alderen som muligt

Hvis I har brug for mere information er i velkommen til at kontakte os på tlf. 39 77 73 60, hverdage mellem 9-14.

På forhånd tak

Med venlig hilsen
Hans Bisgaard og ABC-teamet

INFORMATION TIL DELTAGERE

Tillægsdeltagerinformation i forbindelse med projektet

Asthma Begins in Childhood, ABC

Et kohortestudie under Dansk BørneAstma Center, Gentofte Hospital, Region Hovedstaden.

Kære ABC-forældre.
Vi henvender os til jer for at høre, om I vil give tilladelse til, at jeres barn fortsætter deltagelsen i det videnskabelige studie ABC-kohorten. Planen er at fortsætte studiet med 3 opfølgende besøg ved alderen: 4 år, 5 år og 6 år. Formålet med studiet er stadig at få bedre viden om astmasygdommens udvikling og at finde nye metoder til at forudsige om et barn vil udvikle astma, allergi, høfeber og eksem. Derudover fokuseres der på andre faktorer med indflydelse på børnenes generelle sundhed og udvikling. Dette gøres primært med velkendte undersøgelser fra tidligere besøg.

På de næste sider vil vi beskrive, hvad undersøgelsen går ud på samt praktiske forhold, hvis I beslutter jer for at deltage i undersøgelsen. Det er frivilligt, om I vil deltage. Fortryder I jeres deltagelse, kan I altid trække jer uden I skal komme med forklaring herpå. Tag jer god tid til at læse alle papirerne, før I beslutter jer.

Undersøgelsen kan afbrydes, hvis der kommer resultater, der overflødiggør projektet; f.eks. hvis andre laver en lignende undersøgelse og offentliggør deres resultater før os.
Vi forbeholder os retten til at ekskludere jeres barn fra studiet, hvis de overordnede retningslinjer for projektet ikke overholdes. Alle familier, der har deltaget i ABC-kohortens fase 1 (0-3 år), kan deltage i de planlagte opfølgningsbesøg.

Vi opfordrer jer desuden til at læse det fortrykte tillæg ”Forsøgspersoners rettigheder i et biomedicinsk forskningsprojekt”.

Baggrund og formålet med undersøgelsen

Astma, allergi og eksem er de hyppigste kroniske sygdomme hos børn. Der har gennem de sidste årtier været en kraftig vækst i forekomsten af disse sygdomme, uden at man med sikkerhed har kunnet forklare hvorfor. Vi vil gerne undersøge årsagerne til, at børn udvikler astma, allergi, høfeber og eksem, for bedre at kunne forebygge sygdommene samt rådgive om dem.

Gennem de 11 planlagte besøg, der allerede har været afholdt fra graviditeten indtil 3 års alderen, har vi allerede indsamlet en stor mængde informationer omkring faktorer, der muligvis kan påvirke børnene i det tidligste liv. Flere og flere resultater peger imod, at årsagerne til udviklingen af sygdomme så som astma, allergi og eksem, skal søges i det helt tidlige liv, allerede så tidligt som i graviditeten. Det er ikke udelukkende de astmatiske sygdomme, der ser ud til at være påvirket af det tidligste miljø, men også sygdomme der debuterer senere i livet så som overvægt, diabetes og andre livstilssygdomme. Udviklingen skyldes sandsynligvis et komplekst samspil mellem genetik og det omgivende miljø. Da kun få sygdomme debuterer inden for de 3 første leveår, samtidig med at de astmatiske sygdomme ændrer sig gennem livet, er det utroligt vigtigt, at følge børnenes videre med fortsatte undersøgelser.

Beskrivelse af studiet

ABC-kohorten består af 700 børn, som er fulgt tæt fra fødslen til 3 års alderen. I denne 3 års periode er der været 9 planlagte besøg i klinikken. Ud over disse besøg, har der været to graviditetsbesøg i klinikken.

Nu planlægges yderligere 3 besøg, som kommer til at foregå ved 4, 5 og 6 års alderen. Besøgene forventes at komme til at vare mellem 1 og 3 timer.

Besøgsoversigt:

**Børn**	**4 år**	**5 år**	**6 år**	**Undersøgelser af biologisk materiale**
Dagbog for luftvej + eksem	x	x	x	
Undersøgelse af læge			x	
Tympanometri (trykmåling i øret)			x	
Blodtryk			x	
Vægt og højdemåling	x	x	x	
Næsefilterpapir			x	Immunforsvaret
Elektronisk næse			x	Udåndingsluften
Urinprøve			x	Interleukiner, leukotriener og metaboliske produkter
Blodprøve			x	Immunforsvar, epigenetik og metaboliske produkter
Priktest			x	Allergi
Hårprøve			x	Cotinin (nikotin-produkt)
Afføringsprøve	x	x	x	Mikrobiologisk kolonisering
Hudpodning			x	Mikrobiologisk kolonisering
Halspodning			x	Mikrobiologisk kolonisering
Nasalskrab*			x	mRNA - genekspression
Aktivitetsmåling			x	
DXA-scanning			x	Knogletæthed og fedtfordeling
Spirometri*			x	
Bodybox	x		x	
Multiple Breath Washout	x		x	
Metacholin provokation*			x	
FeNO måling*			x	
CBCL-skema*			x	Child Behaviour Checklist, mentalitet og adfærd
**Forældre**				
Interview om barnets miljø.	x	x	x	
Interview om astma og allergi hos barnet.	x	x	x	

**Nye undersøgelser.*

Nye tillægsundersøgelser, som ikke allerede er udført består af følgende delpunkter:

1. Lungefunktionsundersøgelse (Spirometri med metacholin provokation og FeNO-måling)
2. Nasalskrab (Undersøgelse af udtrykte gener næsens slimhinde)
3. Neurologisk udvikling

På de følgende sider er disse punkter yderligere beskrevet, og bagerst findes underskriftssiderne. Alle tillægsundersøgelserne er godkendt af Videnskabs Etisk komite, Region Hovedstaden.

1. Lungefunktionsundersøgelse

Der gennemføres lungefunktionsundersøgelserne: Helkropsplethysmografi (bodybox) og multiple breath washout (MBW), som allerede er udført tidligere, derudover suppleres med traditionel spirometri med metacholin provokation og FeNO måling.
Formålet med alle disse undersøgelser er, at få bedre viden om astmasygdommens udvikling, at beskrive aktuel lungestatus, samt at finde metoder til at forudsige om et barn vil udvikle astma.
Astma er en inflammatorisk luftvejssygdom, der både involverer de øverste og de nederste dele af lungerne.
Helkropsplethysmografi måler lungefunktionen ved registrering af luftvejsmodstanden hos børn helt ned til 2 år, hvor traditionelle målemetoder, som spirometri, er vanskelige at gennemføre. Spirometri er den mest almindelige lungefunktionsmåling, der kan udføres fra børnene er ca. 5 år gamle, den beskriver luftvejsmodstand i de øverste dele af luftvejene. For at vurdere følsomheden i luftvejene kan man provokere med stoffet metacholin, der irriterer slimhinderne. Derudover anvendes en ny metode til at måle inflammation i de yderste dele af lungerne: MBW. Denne metode menes at kunne detektere astma i tidligere stadier end traditionel lungefunktionsundersøgelse med spirometri og helkropsplethysmografi, der primært måler inflammation i de øverste lungeafsnit. Metoderne har alle været anvendt i flere år på både små og store børn med astma. FeNO-målingen måler fraktioneret nitrogenoxid i udåndingsluften fra børnene. Øgede FeNO-værdier kan være tegn på inflammation i luftvejene og astma.

Metode

Spirometri undersøgelsen foregår i siddende stilling, hvor man inhalerer dybt og derefter tager mundstykket i munden så læberne slutter tæt. Barnet foretager maksimal udånding så kraftigt og hurtigt som muligt. Barnet fortsætter udåndingen indtil undersøgelsen er tilfredsstillende. Ved metacholinprovokationen forstøves stoffet metacholin og inhaleres omtrent som fra en astma-spray. Der indåndes først meget små mængder, senere mere og mere. Lungefunktion følges ved spirometri mellem hver inhalation. Metacholin fremkalder en forbigående forsnævring af luftvejene, som kan påvise luftvejenes følsomhed. MBW og bodyboksmålingen er tidligere beskrevet. FeNO-målingen foretages ved at puste ud gennem et mundstykke, der måler fraktioneret NO indholdet i udåndingsluften.

Risici og ulemper
Der er ikke alvorlige bivirkninger ved spirometri eller metacholin undersøgelsen. Der kan opleves lettere vejrtrækningssymptomer. Hvis målingen viser, at et astmaanfald er på vej, standses undersøgelsen, og der gives straks astmamedicin. FeNO målingen er helt ufarlig.

2. Nasalskrab
Der er stort fokus på genetik i ABC studiet, og der udføres genetiske undersøgelser på eksisterende blodprøver. Disse udføres for at lede efter genetiske typer, der hænger sammen med sygdom, men de eksisterende undersøgelser fortæller ikke om et gen rent faktisk kommer til udtryk. Ved at udføre et nasalskrab er det muligt at undersøge, hvilke gener der kommer til udtryk i næsens slimhinde ved at måle på mRNA i slimhinden. Dette hjælper os til at forstå, hvilke gener der er aktive under sygdom.

Metode
Nasalskrabet udføres med barnet i siddende stilling. Lægen kigger ind i næsen og holder det ene næsebor åbent. Der indføres en lille skarpske og skrabes på næsens skillevæg så man får en lille prøve af næsens slimhinde.

Risici og ulemper
Undersøgelsen er ikke forbundet med nogen former for risici, men der kan forekomme ubehag i form af smerte og en gang imellem lidt næseblod når skrabet udføres.

3. Neurologisk udvikling
I forbindelse med 6 års besøget beder vi forældrene at udfylde et skema, Child Behaviour Checklist (CBCL) til at vurdere børnenes mentale sundhed og adfærdsmæssige udvikling.

Bivirkninger, risici, komplikationer og ulemper
Der er ingen kendte bivirkninger, komplikationer eller risici knyttet til undersøgelserne.
Ved selve undersøgelserne vil der muligvis være ulemper i form af ubehag forbundet med:

- Filterpapir i næsen ved 6 års-alderen.
- Blodprøve ved 6 års-alderen, forudgået af lokalbedøvende salve (EMLA-creme) (20 ml).
- Priktest ved 6 års-alderen.
- Nasalskrab ved 6 års-alderen.
- Halspodning ved 6 års-alderen.
- Opsamling af afføringsprøver 3 gange (á 15 g).
- Opsamling af urinprøve (10 ml).
- DXA-scanning ved 6-års alderen, udsætter barnet for en meget lille stråledosis, som det ellers ikke ville være udsat for. Denne er dog meget beskeden (< en dags baggrundsstråling).

Desuden skal der afsættes tid til registrering i dagbog af luftvejssymptomer og eksem samt besøg på ABC-klinikken, i alt 3 gange over 3 år, cirka 1-3 timer hver gang. Generne ved selve undersøgelserne anses for at være minimale, den største gene er nok den tid der skal afsættes.

Behandling af prøvematerialet
Vi beder om jeres tilladelse til at udtage og opbevare prøver i en biobank, indtil de kan indgå i de planlagte analyser. Vi hverken vil, eller må, analysere prøverne til andre formål, før vi har fået lov af dig. Ligeledes vil det biologiske materiale kun blive anvendt i eventuelt nye forskningsprojekter efter tilladelse fra De Videnskabsetiske Komiteer.

Nytte ved forsøget
Projektets resultater vil forhåbentligt munde ud i en bedre forståelse for astmatiske og allergiske sygdomme, og deres udvikling over tid. Vi håber, at vi med studiet i fremtiden bliver bedre til at undersøge, monitorere og behandle disse sygdomme i de tidlige stadier.

Økonomiske forhold
ABC-studiet udføres under Dansk BørneAstma Center (DBAC), som støttes af offentlige midler og private fonde. Alle donationer fremgår af vores hjemmeside: www.copsac.com. Blandt disse fonde er; Lundbeckfonden, Dansk Strategisk Forskningsråd, Apotekerfonden, og Augustinusfonden. Ingen af projektets ansatte er tilknyttet disse fonde. Dansk BørneAstma Center modtager desuden årligt støtte fra Staten på finansloven på 5 millioner kr. om året. De ansatte på projektet, herunder den forsøgsansvarlige, er helt eller delvist aflønnet via ovennævnte bevillinger. Der gives overenskomstmæssig løn. Forskerne ved Dansk BørneAstma Center har via ny viden, og uden indblanding fra udefrakommende institutioner, taget initiativ til dette studie. Den forsøgsansvarlige har ikke øvrig økonomisk tilknytning til støttegivere.

Adgang til forsøgsresultaterne:
Resultaterne vil blive gemt på en computer på Gentofte Hospital. Alle helbredsoplysninger, som fremkommer i forbindelse med projektet, er fortrolige og underlagt tavshedspligten. Undersøgelsen vil blive beskrevet i videnskabelige tidsskrifter og på vores hjemmeside: www.copsac.com. Ingen uden for forskergruppen får adgang til data. Forsøgsdata opbevares til barnets 18. leveår. Hvis vores undersøgelser fører til betydelige resultater, vil du få mulighed for at få oplysninger om dit barns undersøgelser.

Forsøgsansvarlig og initiativtager
Hans Bisgaard
Overlæge i børnesygdomme, professor, dr. med.
Dansk BørneAstma Center
Gentofte Hospital
Ledreborg Allé 34
2820 Gentofte
Tlf. nr.: 3977 7360

Tak fordi du tog dig tid til at læse materialet. Du er altid velkommen til at kontakte:

Dansk BørneAstma Center

e-mail: abc@boernogastma.dk
Den lægelige **kontaktperson:**
Jakob Stokholm tlf. 3977 7360

Forsøgspersonens rettigheder i et biomedicinsk forskningsprojekt.

Som deltager i et biomedicinsk forskningsprojekt skal du vide at:

- din deltagelse i forskningsprojektet er helt frivillig og kan kun ske efter, at du har fået både skriftlig og mundtlig information om forskningsprojektet og underskrevet samtykkeerklæringen
- du til enhver tid mundtligt, skriftligt eller ved anden klar tilkendegivelse kan trække dit samtykke til deltagelse tilbage og udtræde af forskningsprojektet. Såfremt du trækker dit samtykke tilbage påvirker dette ikke din ret til nuværende eller fremtidig behandling eller andre rettigheder, som du måtte have
- du har ret til at tage et familiemedlem, en ven eller en bekendt med til informationssamtalen
- du har ret til betænkningstid, før du underskriver samtykkeerklæringen
- oplysninger om dine helbredsforhold, øvrige rent private forhold og andre fortrolige oplysninger om dig, som fremkommer i forbindelse med forskningsprojektet, er omfattet af tavshedspligt
- opbevaring af oplysninger om dig, herunder oplysninger i dine blodprøver og væv, sker efter reglerne i lov om behandling af personoplysninger og sundhedsloven
- der er mulighed for at få aktindsigt i forsøgsprotokoller efter offentlighedslovens bestemmelser. Det vil sige, at du kan få adgang til at se alle papirer vedrørende din deltagelse i forsøget, bortset fra de dele, som indeholder forretningshemmeligheder eller fortrolige oplysninger om andre
- der er mulighed for at klage og få erstatning efter reglerne i lov om klage- og erstatningsadgang inden for sundhedsvæsenet

Samtykke fra forældremyndighedens indehaver til deres søns /datters deltagelse i et biomedicinsk forskningsprojekt.

Forskningsprojektets titel: Tillægsprotokol til Asthma Begins in Childhood, ABC-kohorten, fase 2 – opfølgende besøg ved alderen: 4 år, 5 år og 6 år.

Erklæring fra indehaveren af forældremyndigheden:

Jeg har fået skriftlig og mundtlig information og jeg ved nok om formål, metode, fordele og ulemper til at give mit samtykke.

Jeg ved, at det er frivilligt at deltage, og at jeg altid kan trække mit samtykke tilbage uden, at min datter/søn mister sine nuværende eller fremtidige rettigheder til behandling.

Jeg giver samtykke til, at

____(barnets navn + CPR-nummer)

ABC-nummer:______________

deltager i forskningsprojektet og til, at hans/hendes biologiske materiale udtages med henblik på opbevaring i en forsknings-biobank. Jeg har fået en kopi af dette samtykkeark samt en kopi af den skriftlige information om projektet til eget brug.

Navnet eller navnene på forældremyndighedens indehaver(e):

____

Dato ________ Underskrift ________________________________

____

Dato ________ Underskrift ________________________________

Erklæring fra den forsøgsansvarlige:

Jeg erklærer, at der er givet mundtlig information om projektet, udleveret skriftlig information og der foreligger et samtykke til, at forsøgspersonen kan deltage.

Informationsgiverens navn________________________________

Dato ________ Underskrift ________________________________
