# Supplementary material for: Prenatal vitamin D supplementation reduces risk of asthma/recurrent wheeze in early childhood: A combined analysis of two randomized controlled trials
Source: PLoS One. 2017 Oct 27;12(10):e0186657. doi: 10.1371/journal.pone.0186657 (PMC5659607; doi:10.1371/journal.pone.0186657)
Supplement: S3 Text — (PDF) [file pone.0186657.s005.pdf]

PARTNERS
HEALTHCARE
FOUNDED BY BRIGHAM AND WOMEN'S HOSPITAL
AND MASSACHUSETTS GENERAL HOSPITAL

Partners Human Research Committee
116 Huntington Avenue, Suite 1002
Boston, MA 02116
Tel: (617) 424-4100
Fax: (617) 424-4199

Continuing Review: Notification of IRB Approval/Activation
Protocol #: 2009P000557/BWH

Date: September 13, 2016

To: Scott Tillman Weiss, MD,MS
BWH
Medicine / Channing Weiss Group

From: Partners Human Research Committee
116 Huntington Avenue, Suite 1002
Boston, MA 02116

Title of Protocol:	"Randomized Trial of Maternal Vitamin D Supplementation to Prevent Childhood Asthma (VDAART: Vitamin D Antenatal Asthma Reduction Trial)"
Version/Number:	Protocol Version 0.70
Version Date:	3/4/2011
IRB Continuing Review #:	9
IRB Review Type:	Expedited
Expedited Category/ies:	(4)
IRB Approval Date:	9/13/2016
Approval Activation Date:	9/13/2016
IRB Expiration Date:	10/8/2017

This project has been reviewed by BWH IRB . During the review of this project, the IRB specifically considered (i) the risks and anticipated benefits, if any, to subjects; (ii) the selection of subjects; (iii) the procedures for obtaining and documenting informed consent; (iv) the safety of subjects; and (v) the privacy of subjects and confidentiality of the data.

Please note that if an IRB member had a conflict of interest with regard to the review of this project, consistent with IRB policies and procedures, the member was required to leave the room during the discussion and vote on this project except to provide information requested by the IRB.

none

As Principal Investigator, you are responsible for ensuring that this project is conducted in compliance with all applicable federal, state and local laws and regulations, institutional policies, and requirements of the IRB, which include, but are not limited to, the following:

1. Submission of any and all proposed changes to this project (e.g., protocol, recruitment materials, consent form, status of the study, etc.) to the IRB for review and approval prior to initiation of the change(s), except where necessary to eliminate apparent immediate hazards to the subject(s). Changes made to eliminate apparent immediate hazards to subjects must be reported to the IRB as an unanticipated problem.

PARTNERS® HEALTHCARE
FOUNDED BY BRIGHAM AND WOMEN'S HOSPITAL AND MASSACHUSETTS GENERAL HOSPITAL

Partners Human Research Committee
116 Huntington Avenue, Suite 1002
Boston, MA 02116
Tel: (617) 424-4100
Fax: (617) 424-4199

2. Submission of continuing review submissions for re-approval of the project prior to expiration of IRB approval and a final continuing review submission when the project has been completed.
3. Submission of any and all unanticipated problems, including adverse event(s) in accordance with the IRB's policy on reporting unanticipated problems including adverse events.
4. Obtaining informed consent from subjects or their legally authorized representative prior to initiation of research procedures when and as required by the IRB and, when applicable, documenting informed consent using the current IRB approved consent form(s)with the IRB-approval stamp in the document footer.
5. Informing all investigators and study staff listed on the project of changes and unanticipated problems, including adverse events, involving risks to subjects or others.
6. When investigator financial disclosure forms are required, updating your financial interests in Insight and for informing all site responsible investigators, co-investigators and any other members of the study staff identified by you as being responsible for the design, conduct, or reporting of this research study of their obligation to update their financial interest disclosures in Insight if (a) they have acquired new financial interests related to the study and/or (b) any of their previously reported financial interests related to the study have changed.

The IRB has the authority to terminate projects that are not in compliance with these requirements.
Questions related to this project may be directed to Deena G Segal, DSEGAL@PARTNERS.ORG, 857-282-1910.

CC: Augusto Ampil Litonjua, MD,MPH, BWH - Medicine - Channing Weiss Group, Co-Investigator
Sharon C. O'Toole, BWH - Medicine - Women's Health, Research Coordinator/Manager
