# Supplementary material for: Prenatal vitamin D supplementation reduces risk of asthma/recurrent wheeze in early childhood: A combined analysis of two randomized controlled trials
Source: PLoS One. 2017 Oct 27;12(10):e0186657. doi: 10.1371/journal.pone.0186657 (PMC5659607; doi:10.1371/journal.pone.0186657)
Supplement: S4 Text — (PDF) [file pone.0186657.s006.pdf]

Original Investigation

Effect of Prenatal Supplementation With Vitamin D on Asthma or Recurrent Wheezing in Offspring by Age 3 Years
The VDAART Randomized Clinical Trial

Augusto A. Litonjua, MD, MPH; Vincent J. Carey, PhD; Nancy Laranjo, BA; Benjamin J. Harshfield, BA; Thomas F. McElrath, MD, PhD; George T. O'Connor, MD, MS; Megan Sandel, MD, MPH; Ronald E. Iverson Jr, MD, MPH; Aviva Lee-Paritz, MD; Robert C. Strunk, MD, PhD; Leonard B. Bacharier, MD; George A. Macones, MD, MSCE; Robert S. Zeiger, MD, PhD; Michael Schatz, MD, MS; Bruce W. Hollis, PhD; Eve Hornsby, PhD; Catherine Hawrylowicz, PhD; Ann Chen Wu, MD, MPH; Scott T. Weiss, MD, MS

IMPORTANCE Asthma and wheezing begin early in life, and prenatal vitamin D deficiency has been variably associated with these disorders in offspring.

OBJECTIVE To determine whether prenatal vitamin D (cholecalciferol) supplementation can prevent asthma or recurrent wheeze in early childhood.

DESIGN, SETTING, AND PARTICIPANTS The Vitamin D Antenatal Asthma Reduction Trial was a randomized, double-blind, placebo-controlled trial conducted in 3 centers across the United States. Enrollment began in October 2009 and completed follow-up in January 2015. Eight hundred eighty-one pregnant women between the ages of 18 and 39 years at high risk of having children with asthma were randomized at 10 to 18 weeks' gestation. Five participants were deemed ineligible shortly after randomization and were discontinued.

INTERVENTIONS Four hundred forty women were randomized to receive daily 4000 IU vitamin D plus a prenatal vitamin containing 400 IU vitamin D, and 436 women were randomized to receive a placebo plus a prenatal vitamin containing 400 IU vitamin D.

MAIN OUTCOMES AND MEASURES Coprimary outcomes of (1) parental report of physician-diagnosed asthma or recurrent wheezing through 3 years of age and (2) third trimester maternal 25-hydroxyvitamin D levels.

RESULTS Eight hundred ten infants were born in the study, and 806 were included in the analyses for the 3-year outcomes. Two hundred eighteen children developed asthma or recurrent wheeze: 98 of 405 (24.3%; 95% CI, 18.7%-28.5%) in the 4400-IU group vs 120 of 401 (30.4%, 95% CI, 25.7%-73.1%) in the 400-IU group (hazard ratio, 0.8; 95% CI, 0.6-1.0; $P = .051$). Of the women in the 4400-IU group whose blood levels were checked, 289 (74.9%) had 25-hydroxyvitamin D levels of 30 ng/mL or higher by the third trimester of pregnancy compared with 133 of 391 (34.0%) in the 400-IU group (difference, 40.9%; 95% CI, 34.2%-47.5%, $P < .001$).

CONCLUSIONS AND RELEVANCE In pregnant women at risk of having a child with asthma, supplementation with 4400 IU/d of vitamin D compared with 400 IU/d significantly increased vitamin D levels in the women. The incidence of asthma and recurrent wheezing in their children at age 3 years was lower by 6.1%, but this did not meet statistical significance; however, the study may have been underpowered. Longer follow-up of the children is ongoing to determine whether the difference is clinically important.

TRIAL REGISTRATION clinicaltrials.gov Identifier: NCT00920621

JAMA. 2016;315(4):362-370. doi:10.1001/jama.2015.18589

Editorial page 347

Related article page 353

Supplemental content at jama.com

Author Affiliations: Author affiliations are listed at the end of this article.

Corresponding Author: Augusto A. Litonjua, MD, MPH, Channing Division of Network Medicine, Department of Medicine, Brigham and Women's Hospital, 181 Longwood Ave, Boston, MA 02115 (augusto.litonjua@channing.harvard.edu).

Copyright 2016 American Medical Association. All rights reserved.

Downloaded From: http://jamanetwork.com/pdfaccess.ashx?url=/data/journals/jama/934885/ by a Harvard University User on 03/30/2017

Wheezing illnesses begin as early as the first few weeks of life, suggesting that there may be prenatal determinants involved with the genesis of these illnesses. Although wheezing and other asthmalike symptoms are common in the preschool ages, it remains difficult to predict which of these preschoolers will have asthma that persists through midchildhood and onward. Nevertheless, preschool children with wheezing have lung function deficits that persist through later life.[1,2] Thus, prenatal interventions that prevent early recurrent wheezing may be important to reduce the short-term morbidity and to prevent the potential long-term sequelae of reduced lung growth.

Vitamin D deficiency occurs worldwide. Vitamin D status is defined by the circulating level of 25-hydroxyvitamin D, from either plasma or serum. Pregnant and lactating mothers and their neonates are at especially high risk of vitamin D deficiency. In a nationally representative sample of pregnant US women, 69% had 25-hydroxyvitamin D levels in the insufficient range (ie, ≤30 ng/mL).[3]

Vitamin D deficiency in pregnancy may be important in early asthma and wheezing because vitamin D has effects on the developing lung and immune system during the fetal and early postnatal periods.[4-8] Observational studies that have investigated the association of vitamin D in pregnancy on the development of wheeze, asthma, and allergies in early life have produced mixed results. We therefore designed a clinical trial of vitamin D (cholecalciferol) supplementation in pregnancy to address the question of whether supplementation with vitamin D during pregnancy can prevent the development of asthma-related phenotypes in very young children—the Vitamin D Antenatal Asthma Reduction Trial (VDAART).

Methods

Participants

Pregnant women were recruited from 3 clinical sites across the United States—Boston Medical Center, Boston, Massachusetts; Washington University at St Louis, St Louis, Missouri; and Kaiser Permanente Southern California Region, San Diego. The data coordinating center was based in the Channing Division of Network Medicine, Brigham and Women's Hospital, Boston, Massachusetts. Eligible participants were women between the ages of 18 and 39 years, who presented between the estimated gestational ages of 10 and 18 weeks; who had a history of asthma, eczema, or allergic rhinitis, or whose partner (biologic father of the child) had a history of asthma, eczema, or allergic rhinitis; who was a nonsmoker; and who was English or Spanish speaking, with intent to participate for 4 years (up to the third birthday of the child). The study protocol was approved by the institutional review boards at each participating institution and at the Brigham and Women's Hospital. All women provided written informed consent.

Study Design

Details of the study design are available in the protocol (Litonjua et al[9] and Supplement 1). We conducted a randomized, double-blind, placebo-controlled study of vitamin D (daily 4000 IU of vitamin D plus a multivitamin with 400 IU of vitamin D) vs placebo (daily placebo pill plus a multivitamin with 400 IU of vitamin D). Participants were screened for eligibility between October 2009 and July 2011. Follow-up of the last child was completed in January 2015. Prenatal interval visits, labor and delivery room data collection, and postnatal visits and data collected during these visits are detailed in the eMethods in Supplement 2. Study staff met with pregnant women monthly to administer a brief health questionnaire, assess medication use, and monitor for complications (via the questionnaire and medical record review). Adherence was assessed with electronic medication container caps. After delivery, children were monitored by telephone every 3 months and in-person annually for 3 years, during which time infants' health, respiratory symptoms, and medications were assessed. Data collection is summarized in eTable 1, and the number of scheduled and completed telephone calls and visits are summarized in eTable 2, both in Supplement 2.

Randomization was performed using a system that automates the random assignment of treatment groups to study identification numbers. The randomization scheme used stratified permuted blocks with randomly varied block sizes of 4 and 6, and 1 block allocation list per stratum (study site and racial/ethnic group) (**Figure 1**).[9]

The study had 2 primary outcomes. The first was parental report of physician diagnosis of asthma or occurrence of recurrent wheeze in the child's first 3 years of life ascertained from questionnaires administered every 3 months. Parental report of physician's diagnosis of asthma was taken directly from the offspring questionnaires. Recurrent wheeze was defined by the occurrence of at least 1 of the following 5 conditions: (1) parental report of wheeze after child's second birthday preceded by at least 1 report of wheeze prior to second birthday; (2) report of child's use of asthma controller medication (defined as report of use of steroid inhalers or nebulizers, leukotriene modifiers, or steroid pills or liquids) after the second birthday, preceded by a report of wheeze before the second birthday; (3) 2 or more distinct parental reports of wheeze after the second birthday; (4) at least 1 parental report of wheeze and use of asthma controller medications at distinct visits, both subsequent to the second birthday; or (5) 2 distinct reports of use of asthma controller medications after the second birthday. The second, coprimary outcome was achieved maternal 25-hydroxy vitamin D level of 30 ng/mL or higher at the third trimester sampling. This cutoff was the usual accepted minimum level of sufficiency based on evidence of associations with outcomes[10] at the start of the trial. However, the Institute of Medicine released its statement in 2011,[11] setting a level of 20 ng/mL or higher as sufficient for bone health. Thus, both cutoffs were analyzed.

Prespecified secondary outcomes were assessed at 3 years of age, including child 25-hydroxyvitamin D levels, parental report of physician's diagnosis of eczema with rash in typical distribution, total IgE and allergen sensitization (specific IgE to a panel of aeroallergens and food allergens), and lower respiratory tract infections (LRIs) (defined as parental report of physician-diagnosed bronchitis, bronchiolitis, croup, or pneumonia). Questions about the presence of LRIs were asked every 3 months, allowing for recurrent events. Counts of re-

Copyright 2016 American Medical Association. All rights reserved.

Downloaded From: http://jamanetwork.com/pdfaccess.ashx?url=/data/journals/jama/934885/ by a Harvard University User on 03/30/2017

Figure 1. The VDAART Participant Flow

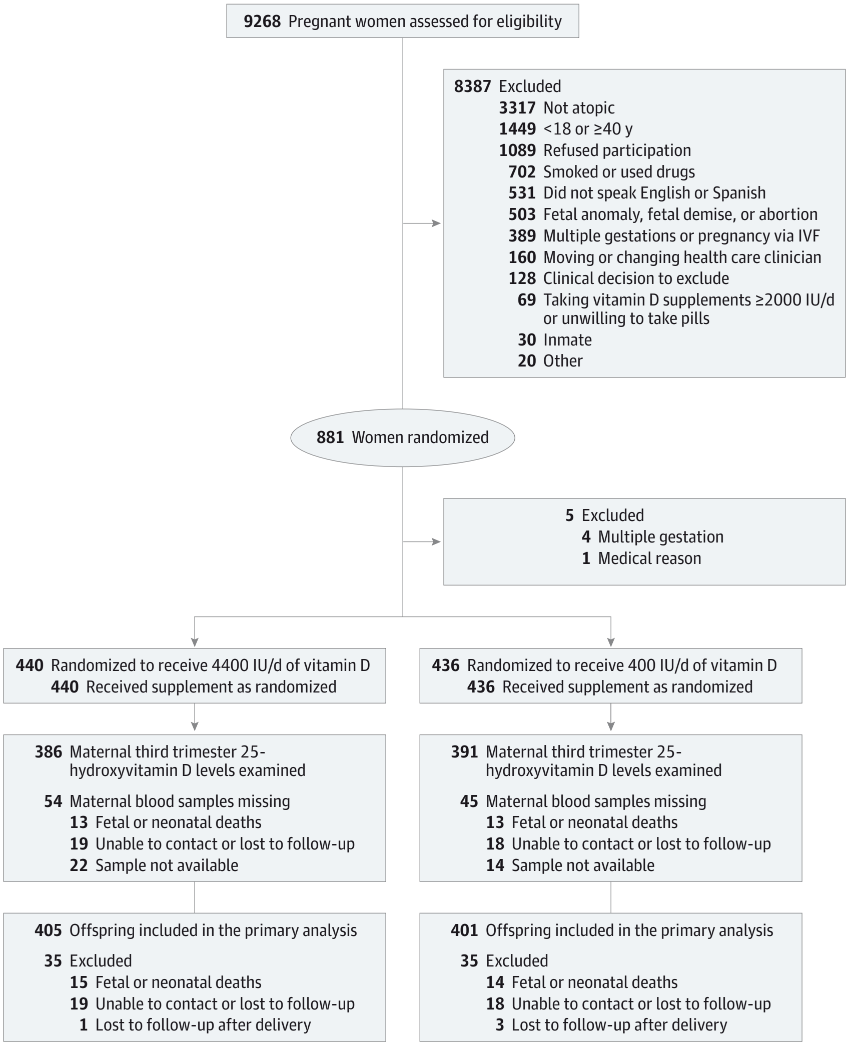

IVF indicates in vitro fertilization.

ported LRIs were assembled from all available follow-up data on each child. For details on the questions from which the outcomes were derived and the measurement of total and specific IgE, please see the eMethods (Supplement 2).

Blood was drawn from mothers at enrollment, 32 to 38 weeks' gestation, and 1 and 3 years after delivery. Cord blood was obtained and children had blood drawn at ages 1 and 3 years. Circulating 25-hydroxyvitamin D from maternal and child plasma samples was determined using the DiaSorin Liaison (DiaSorin) chemiluminescence immunoassay,[12] whereas for cord blood samples liquid chromatography tandem mass spectrometry[13] was used (eMethods, Supplement 2). (To convert 25-hydroxyvitamin from ng/mL to nmol/L, multiply by 2.496).

Race and ethnicity information was collected because they are determinants of circulating 25-hydroxyvitamin D levels. Participants were asked to first categorize themselves as either Hispanic or non-Hispanic, then to categorize their race into prespecified categories. The child's ethnicity and race was determined by responses to the questions regarding maternal or paternal ethnicity and race. In the analyses, ethnic and racial groups were collapsed into 4 groups: black, white Hispanic, nonwhite Hispanic, and other. Further details are in the eMethods (Supplement 2).

Safety Monitoring

Monitoring for adverse events occurred on a monthly basis during the active intervention phase. The occurrence of prespecified severe adverse events and adverse events (symptomatic hypercalcemia; eclampsia; preeclampsia; hemolysis, elevated liver enzymes, and low platelet count [HELLP] syndrome; or death) were identified through maternal questionnaires and review of the obstetrical medical record. In addition, potential hypercalcemia was monitored through monthly checks of urinary calcium-to-creatinine ratios (eMethods, Supplement 2). Neonatal adverse events (preterm delivery, congenital anomalies, and fetal or neonatal death) were identified through the labor and delivery medical record review. Attribution of adverse events to vitamin D treatment was determined by independent review by the data and safety monitoring board.

Copyright 2016 American Medical Association. All rights reserved.

Downloaded From: http://jamanetwork.com/pdfaccess.ashx?url=/data/journals/jama/934885/ by a Harvard University User on 03/30/2017

Statistical Analysis

The trial was designed to detect a 25% reduction[14] in the incidence of asthma and recurrent wheeze in the first 3 years of life in the supplemented group. Using data from 2 birth cohorts,[15,16] the incidence of asthma and recurrent wheeze in the control group was estimated at 40% to 50%, when either the mother or the father had asthma or allergies. The recruitment target was 870 pregnant women. Assuming an 8% miscarriage rate and 17% to 18% loss to follow-up, the targeted sample size at age 3 years was 660 children. With a 3-year incidence of 45% in the control group, the power to detect a 25% reduction in the supplemented group was 83%.

To make full use of all available observation time and to obtain a fully nonparametric intent-to-treat analysis, the original protocol-based plan to base treatment effect inference on logistic regression analysis was replaced by a plan to use interval-censored event-time analysis. This change in plan was reviewed and approved, prior to unbinding and analysis, by the study's steering committee, and the overall final analysis was reviewed and approved by the data and safety monitoring board. Tests of treatment effect for the first occurrence of the primary outcome used either the earlier of time to asthma diagnosis or the earliest wheeze report or medication event indicative of recurrent wheeze. Distributions of outcome-free time were compared between groups using nonparametric maximum likelihood estimation to create Kaplan-Meier curves, and optimal nonparametric testing with interval censored response times.[17,18] Estimates of proportions of children experiencing the primary outcome at selected times are derived from these curves (Kaplan-Meier estimates). The nonparametric procedure with interval-censored response times does not allow incorporation of covariate effects. The Weibull family of parametric models for interval-censored event times[19] was used to conduct adjusted testing and estimation of treatment and covariate effects. Additional models incorporating effects of clinical center and maternal educational level were conducted post hoc using logistic regression based on the subset of participants providing complete follow-up times, and interval censored Weibull regression using all available observation time.

Outcome events were analyzed using the interval formed from the last negative visit and first positive visit or departure from or completion of the study. Individuals who had no reports of asthma or recurrent wheeze up to the time of departure from the study were censored at that time.

Reports of LRIs were counted, represented as an integer-valued outcome variable and analyzed using a negative binomial generalized linear model to accommodate extra-Poisson variation.[20] The effect of treatment on the LRI rate per month was estimated using the log of person-months in the study as offset. Mean total IgE at age 3 years was compared between groups using a common-variance *t* test with log-transformed measures. Analyses of maternal vitamin D and secondary outcomes assume missingness occurred completely at random. Statistical analyses were conducted using SAS version 9.3 (SAS Institute Inc.) and R version 3.1 (R Foundation for Statistical Computing; packages interval, survival, ggplot2). All tests were 2-sided and the significance level was prespecified at $P < .05$, even for the coprimary outcomes because these have distinct interpretations.

Table 1. Baseline Characteristics of Mothers Participating in the Vitamin D Antenatal Asthma Reduction Trial (N = 876)

	No. (%) of Participants	
	4400 IU/d of Vitamin D (n = 440)	400 IU/d of Vitamin D (n = 436)
Age, mean (SD), y	27.5 (5.5)	27.3 (5.6)
Gestation age, mean (SD), wk	14.1 (2.8)	14.2 (2.7)
25-Hydroxyvitamin D, mean (SD), ng/mL[a]	23.3 (10.1)	22.5 (10.1)
Mother		
Asthma	191 (43)	167 (38)
Allergic rhinitis	275 (63)	283 (65)
Eczema	139 (32)	139 (32)
Maternal partner		
Asthma	108 (25)	94 (22)
Allergic rhinitis	173 (39)	192 (44)
Eczema	81 (18)	63 (14)
Mother's race/ethnicity		
Black	190 (43)	190 (44)
White Hispanic	59 (13)	61 (14)
White non-Hispanic	114 (26)	116 (27)
Other	77 (18)	69 (16)
Educational status		
<High school	66 (15)	42 (10)
High school or technical school	123 (28)	142 (33)
Some college	108 (25)	105 (24)
College graduate or graduate school	143 (33)	147 (34)
Marital status		
Married	191 (43)	203 (47)
Divorced or separated	13 (3)	10 (2)
Not married	236 (54)	223 (51)
Household income, US $		
<30 000	132 (30)	128 (29)
30 000-49 999	62 (14)	58 (13)
50 000-74 999	50 (11)	51 (12)
75 000-99 999	42 (10)	39 (9)
100 000-149 000	35 (8)	36 (8)
>150 000	18 (4)	14 (3)
Refused to say or unknown	101 (23)	110 (25)

SI conversion factor: To convert 25-hydroxyvitamin D from ng/mL to nmol/L, multiply by 2.496.

[a] The 4400 vitamin D IU group included 437 women; 400 vitamin D IU group, 433 women.

Results

Characteristics of the Trial Population

Eight hundred eighty-one women were randomized (Figure 1), but 5 women were found to be ineligible, resulting in 876 women—440 to the 4400 IU/d vitamin D group and 436 to the 400 IU/d vitamin D group. The baseline characteristics of the pregnant women were comparable between the 2 treatment groups (**Table 1**). For the analyses on the childhood outcomes,

Copyright 2016 American Medical Association. All rights reserved.

Downloaded From: http://jamanetwork.com/pdfaccess.ashx?url=/data/journals/jama/934885/ by a Harvard University User on 03/30/2017

Table 2. Pregnancy and Infant Characteristics of Mothers Whose Children Contributed Delivery and Follow-up Data (n = 806)[a]

	No. (%) of Participants		
	4400 IU/d of Vitamin D (n = 405)	400 IU/d of Vitamin D (n = 401)	Mean Difference Between Groups (95% CI)
**Maternal Characteristics**			
25-Hydroxyvitamin D levels, ng/mL			
Baseline			
Women, No.	402	399	
Mean (95% CI)	23.3 (22.3 to 24.4)	22.6 (21.6 to 23.6)	0.7 (−0.7 to 2.2)
Third trimester			
Women, No.	383	387	
Mean (95% CI)	39.3 (37.8 to 40.9)	26.8 (25.7-27.9)	12.4 (10.7-14.4)
Cord blood[b]			
Newborn, No.	303	317	
Mean (95% CI)	28.0 (26.6 to 29.4)	19.2 (18.1 to 20.3)	8.8 (7.1 to 10.6)
Time in study, mean (95% CI), d[c]	173.5 (171.2 to 175.8)	174.4 (172.0 to 176.8)	−0.9 (−4.3 to 2.4)
Medication adherence (MEMS), %			
Women, No.	402	400	
Mean (95% CI)	70 (67.3 to 72.7)	71.3 (68.6 to 74.0)	−1.3 (−5.2 to 2.5)
			**Rate Difference (95% CI)[d]**
Mode of delivery			
Cesarean	123 (31)	116 (29)	2 (−8 to 5)
Vaginal	279 (69)	285 (71)	−2 (−10 to 6)
Delivery <37 wk	40 (10)	31 (8)	2 (−2 to 6)
**Infant Characteristics in First Year**			
Birth weight, g			
Infants, No.	402	401	
Mean (95% CI)	3267.9 (3214.9 to 3321.1)	3282.5 (3224.9 to 3340.1)	−14.5 (−92.8 to 63.7)
Birth length, cm			
Infants, No.	401	399	
Mean (95% CI)	50.6 (50.3 to 50.9)	50.7 (50.3 to 51.0)	−0.1 (−0.5 to 0.4)
Head circumference, cm			
Infants, No.	398	398	
Mean (95% CI)	34 (33.8 to 34.2)	34 (33.8 to 34.2)	0 (−0.3 to 0.3)
			**Rate Difference (95% CI)[d]**
Sex			
Boy	200 (49)	220 (55)	−5 (−13 to 2)
Girl	205 (51)	181 (45)	5 (−2 to 13)
Race/ethnicity			
Black	199 (49)	191 (48)	2 (−6 to 9)
White Hispanic	53 (13)	51 (13)	0 (−5 to 5)
White, non-Hispanic	82 (20)	79 (20)	1 (−5 to 6)
Other	71 (18)	80 (20)	−2 (−8 to 3)
First-year reports			
Breastfeeding	213 (55)	206 (53)	2 (−6 to 9)
Formula use	338 (87)	330 (85)	2 (−3 to 7)
400 IU Vitamin D drops/d	179 (46)	169 (43)	2 (−5 to 10)
Use of multivitamin drops	74 (19)	87 (22)	−3 (−9 to 3)

Abbreviation: MEMS, medication event monitoring system.

SI conversion factor: To convert 25-hydroxyvitamin from ng/mL to nmol/L, multiply by 2.496.

[a] Numbers and percentages reflect some missing data and may not sum due to rounding.

[b] Measured by liquid chromatography coupled to tandem mass spectrometry.

[c] Enrollment to delivery.

[d] Rate differences are expressed as difference in percentages.

Copyright 2016 American Medical Association. All rights reserved.

Downloaded From: http://jamanetwork.com/pdfaccess.ashx?url=/data/journals/jama/934885/ by a Harvard University User on 03/30/2017

Table 3. Treatment Comparisons for Primary and Secondary Outcomes in the Vitamin D Antenatal Asthma Reduction Trial

	Vitamin D, IU/d			
	4400	400	Difference (95% CI)	*P* Value[a]
No. of offspring	405	401		
**Coprimary End Points**[b]				
Asthma or recurrent wheeze in first 3 y of life[c]				
Positive, No. (%)	98 (24.3)	120 (30.4)	−6.1 (−30 to 18)	.051
HR (95% CI)	0.8 (0.6 to 1.0)	1 [Reference]		
25 Hydroxyvitamin D ≥30 ng/mL[d]				
Women positive, No. (%)	289 (74.9)	133 (34.0)	40.9 (34.2 to 47.5)	<.001
**Secondary End Points Among Offspring**				
In first 3 y of life				
Eczema with rash[c]				
Positive, No. (%)	83 (21)	89 (23)	−2 (−17 to 13)	.56
HR (95% CI)	0.9 (0.7 to 1.2)	1 [Reference]		
Lower respiratory tract infections[e]				
Any, No. (%)	129 (31.9)	137 (34.2)	-2.3 (−9.1 to 4.4)	.07
Unique events reported, No.	222	276		
Negative binomial rate ratio (95% CI)	0.8 (0.6 to 1.0)	1 [Reference]		
Age 3 y measures				
Log total IgE concentration[f]				
Geometric mean (95% CI)	29.3 (24.2 to 35.4)	37.3 (30.7 to 45.4)	−8 (−17.2 to 1.2)	.08
No. of children	276	262		
Any allergic sensitization				
Positive test results for allergens, No./total	146/279	147/264	−3.4 (−12.0 to 5.4)	.49
Mean (95% CI), %	52.3 (46.3 to 58.3)	55.7 (49.5 to 61.8)		
Positive-specific IgE tests				
Positive, %	10.7	12.4	−1.7 (−3.4 to 0.0)	.02
No. of events/No. tests	414/3881	458/3700		
Specific tests per person, mean (SD)	13.9 (0.48)	13.9 (0.86)		

Abbreviation: HR, hazard ratio.

SI conversion factor: To convert 25-hydroxyvitamin from ng/mL to nmol/L, multiply by 2.496.

[a] Two-sided and intention-to-treat *P* values were derived from the nonparametric procedure of Sun.

[b] Hazard ratios for these outcomes were estimated using a Weibull model; HRs and CIs are 2 sided.

[c] Asthma or recurrent wheeze and eczema with rash were analyzed as events with interval-censored times of occurrence; estimated proportions are based on the method of Kaplan and Meier.

[d] Maternal achievement of 25-hydroxyvitamin D levels of 30 ng/mL or higher (386 in the 4400 IU group; 391 in the 400 UI group) and report of any allergic sensitizations by offspring at age 3 years were analyzed as Bernoulli outcomes.

[e] Lower respiratory infection was analyzed as a count of recurring events governed by the negative binomial distribution, to accommodate extra-Poisson variation in these counts (binomial generalized linear model).

[f] The total number of positive specific IgE per child was recorded as a binary outcome per test, summed to a binomial outcome per visit. Log total IgE was analyzed as a Gaussian variable, transformed to geometric mean (*t* test).

data on 806 mothers and their children were available—405 in the 4400-IU group vs 401 in the 400-IU group (35 participants in the 4400-IU group and 35 participants in the 400-IU group did not contribute data on the infants and children due to fetal deaths or early loss to follow-up). Fifty-eight of 806 children did not provide a full 3 years of observation time, and thus do not provide information on the protocol-defined outcome. No significant differences were observed in comparisons of characteristics of newborns in the 2 treatment groups (**Table 2**).

Effect of Treatment on Maternal, Cord Blood, and Child 25-Hydroxyvitamin D Levels

Maternal levels of 25-hydroxyvitamin D in the third trimester were higher in the 4400-IU group (mean, 39.2 ng/mL) vs the 400-IU group (mean, 26.8 ng/mL; mean difference, 12.4; 95% CI, 10.5-14.3 ng/mL, *P* < .001; eFigure 1A in Supplement 2). The proportion of women who achieved 25-hydroxyvitamin D levels higher than 30 ng/mL in the 4400-IU group (74.9%) was greater than that in the 400-IU group (34.0%; difference, 40.9%, 95% CI, 34.2%-47.5%; *P* < .001) (**Table 3**). Similarly, the proportion of women who achieved 25-hydroxyvitamin D levels of 20 ng/mL was greater in the 4400-IU group (88.6%) than those in the 400-IU group (71.6%; difference, 17%; 95% CI, 11.3%-22.7%, *P* value <.001). For cord blood 25-hydroxyvitamin D levels, samples from the 4400-IU group had statistically significantly greater levels than those from the 400-IU group (Table 2). No treatment group-related differences in 25-hydroxyvitamin D levels were observed in the mothers 1-year postpartum (eFigure 1A) or in the children at 1 year and 3 years (eFigure 1B in Supplement 2).

Copyright 2016 American Medical Association. All rights reserved.

Downloaded From: http://jamanetwork.com/pdfaccess.ashx?url=/data/journals/jama/934885/ by a Harvard University User on 03/30/2017

Figure 2. Asthma or Recurrent Wheeze-Free Proportion by Treatment

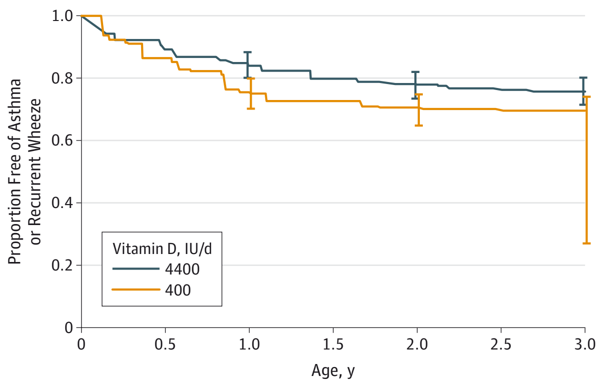

No. at risk Vitamin D, IU/d				
4400	405	349	319	309
400	401	320	287	281

Kaplan-Meier survival estimates. Error bars indicate 95% CI estimates at intervals of 1, 2, and 3 years. Estimates were obtained from nonparametric maximum likelihood estimation, with optimal nonparametric testing with interval-censored response times. These interval-censored response times lead to gaps in the curves, which we have denoted with diagonal lines. The hazard ratio for the time to first event of asthma or recurrent wheeze was 0.8 (95% CI, 0.6-1.0; *P* value = .051).

Effect of Treatment on Asthma/Recurrent Wheeze

Two hundred eighteen (27%) of the eligible 806 children developed asthma or recurrent wheezing according to the composite definition by their third-year visit with many children fulfilling multiple criteria and with broad overlap between these criteria (eTable 3 in Supplement 2). There were 58 children for whom the outcome could not be resolved (29 in the 4400-IU group and 29 in the 400-IU group) because they were missing interim- or final-visit data that would have categorized the outcome; however, all available observation time on all 806 children was used in the interval-censored analysis. In the 4400-IU group, 98 children (estimate, 24.3%, 95% CI, 18.7%-28.5%) developed asthma or recurrent wheeze by age 3 years, whereas in the 400-IU group, 120 children (estimate, 30.4%; 95% CI, 25.7%-73.1%) developed asthma or recurrent wheeze by age 3 years (hazard ratio [HR], 0.8; 95% CI, 0.6-1.0; nonparametric *P* = .051) (**Figure 2** and Table 3). The estimates of the incidence of asthma or recurrent wheeze at 1 year were 16.0% (95% CI, 11.6%-19.5%) in the 4400-IU group vs 24.9% (95% CI, 20.2%-31.7%) in the 400-IU group. At 2 years of age, the estimates were 22.0% (95% CI, 16.7%-26.5%) in the 4400-IU group vs 29.4% (95% CI, 25.2-36.1) in the 400-IU group.

We performed sensitivity analyses to complement the main intention-to-treat (ITT) analysis (eResults and eTable 4 in Supplement 2), using a Weibull model for interval-censored event times and a logistic regression analysis using only individuals who provided the full 3-year outcomes (n = 748). When adjustments for site and maternal education were made, inferences and estimates of treatment effect were consistent with the primary ITT analysis for both modeling approaches. For the site-adjusted logistic regression, the odds ratio (OR) was 0.74 (95% CI, 0.54-1.02; *P* = .063) and for the site and education adjusted model, the OR was 0.72 (95% CI, 0.52-1.00; *P* = .049).

Secondary Outcomes

The results of the analyses on the secondary outcomes are presented in Table 3. There were no significant differences in the development of eczema, LRI, and total IgE levels. However, children in the 4400-IU group had fewer positive specific IgE tests (10.7%, 95% CI, 9.4%-12.1%) than children in the 400 IU/d group (12.4%, 95% CI, 11.45-13.5%, *P* = .02).

Safety

There were no significant differences in the rates of severe adverse events between the 2 treatment groups (eResults and eTable 5 in Supplement 2), and no adverse event was attributed to vitamin D treatment. No events of hypercalcemia in the mothers occurred with vitamin D treatment.

Discussion

This randomized, double-blinded multicenter clinical trial studied effects of prenatal treatment with vitamin D on 806 infants followed up from birth to 3 years. The study demonstrated that supplementation with 4400 IU/d of vitamin D significantly raised 25-hydroxyvitamin D levels in pregnant women. We estimated that the 3-year incidence of asthma or recurrent wheeze in the infants was 24.3% with 4400-IU/d and 30.4% with a 400-IU/d supplement. This absolute reduction of 6.1% was not statistically significant (*P* = .051). Both the 3-year incidence of asthma or recurrent wheeze observed for recipients of 400 IU/d and the reduction associated with the 4400 IU/d treatment were less than we hypothesized when we designed the study; thus, the study power may have been lower than anticipated. In addition, most of the secondary outcomes were not statistically significantly different between groups, and these analyses should be considered exploratory given the null primary outcome and the absence of adjustment for multiple comparisons. Therefore, whether supplementation of pregnant women with vitamin D will reduce asthma and recurrent wheeze in their offspring at age 3 years remains unclear. Larger studies and longer follow-up of the children in this study will be needed to answer the question. If additional studies identify a significant effect, given the high prevalence of low vitamin D levels in pregnant women, the effect of this inexpensive intervention on child health could be substantial.

At present, there is no consensus on the definition of a minimal clinically important difference for prenatal interventions against offspring asthma and wheezing. A multicenter trial involving 6154 primary care clinic participants that used a multiintervention approach (increased intake of n-3 polyunsaturated fatty acids and oily fish, reduced parental smoking, and reduced indoor dampness) showed a lower asthma incidence by 2 years of age in the intervention group (71 of 1374, 5.2%) vs the control group (337 of 4780, 7.1%) with a difference of 1.9% (OR, 0.72; 95% CI, 0.55-0.93).[21] Another multi-

Copyright 2016 American Medical Association. All rights reserved.

Downloaded From: http://jamanetwork.com/pdfaccess.ashx?url=/data/journals/jama/934885/ by a Harvard University User on 03/30/2017

intervention trial (avoidance of house dust mite, pet allergen, and environmental tobacco smoke; encouragement of breastfeeding; and delayed introduction of solid foods) in 545 high-risk infants on the basis of an immediate family history found a decreased asthma incidence in 2-year old children in the intervention (40 of 246, 16.3%) vs the control (53 of 230, 23.0%) groups, for a 6.7% difference (adjusted OR, 0.60, 95% CI, 0.37-0.95). The effect sizes for these diverse interventions are of comparable magnitude to our estimate of prenatal vitamin D supplementation.

Although the diagnosis of asthma in early life is difficult, the primary symptom associated with asthma—wheezing—is common in this period in life, with attendant high resource utilization.[22] It is recognized that several wheezing phenotypes exist in early childhood (≤3 years old), and a subset of these children (about 40%) will have symptoms that persist through age 6 years[23] and are ultimately diagnosed as asthma. Although several asthma predictive indices have been tested, these are specific but not very sensitive predictors of asthma that persists through age 6 years.[24,25] Wheezing in early life, with or without a subsequent asthma diagnosis, may have long-term consequences, such as deficits in lung function in adolescence,[1] impairments in aspects of health-related quality of life,[26] and more rapid lung function declines in transient wheezers despite attaining normal lung function in early adulthood.[27] Therefore, we chose the outcome of a parental report of asthma or recurrent wheeze by 3 years of age because we hypothesized that prenatal interventions are likely to affect this early phenotype.

A number of limitations in VDAART are worthy of mention. The population was selected because the offspring of these mothers were at high risk of asthma and allergies; thus, caution should be exercised before generalizing these results to nonhigh-risk populations. Timing of vitamin D supplementation during pregnancy may not have been ideal. On average, supplementation was initiated at about the 14th week of gestation, but lung development begins in the 4th week of gestation.[28] Finally, fewer women than expected achieved the target threshold level of 30 ng/mL of 25-hydroxyvitamin D by the third trimester, suggesting that the dose of 4400 IU/d may not be adequate for all pregnant women.

Because lung development continues throughout childhood,[28] it is possible that vitamin D continues to exert its influence on the respiratory and immune systems in the postnatal period. In this trial, we saw a significant difference in 25-hydroxyvitamin D levels in cord blood between the 2 groups, but this difference disappeared by 1 year. Consistent with these findings, the differences in asthma or recurrent wheeze between the 2 groups in the first year were greater than in the second and third years, suggesting an early effect that was not sustained. Thus, adequate vitamin D supplementation after birth to maintain 25-hydroxyvitamin D levels in the sufficient range, in addition to prenatal supplementation, may be needed to more fully prevent wheezing illnesses, and perhaps asthma, in early life.

All of these factors—the timing of the intervention, the dose of vitamin D administered, adherence with treatment, and inadequate postnatal vitamin D supplementation—may have limited the effect of the intervention on asthma or recurrent wheeze by 3 years. Future studies will need to address these issues.

Conclusions

In pregnant women at risk of having a child with asthma, supplementation with 4400 IU/d of vitamin D compared with 400 IU/d significantly increased vitamin D levels in the women. The incidence of asthma and recurrent wheezing in their children at age 3 years was lower by 6.1%, but this did not meet statistical significance; however, the study may have been underpowered. Longer follow-up of the children is ongoing to determine whether the difference is clinically important.

ARTICLE INFORMATION

Author Affiliations: Channing Division of Network Medicine, Department of Medicine, Brigham and Women's Hospital, Boston, Massachusetts (Litonjua, Carey, Laranjo, Harshfield, Weiss); Harvard Medical School, Boston, Massachusetts (Litonjua, Carey, McElrath, Wu, Weiss); Department of Obstetrics and Gynecology, Brigham & Women's Hospital, Boston, Massachusetts (McElrath); Pulmonary Center, Department of Medicine, Boston University School of Medicine, Boston, Massachusetts (O'Connor); Department of Pediatrics, Boston Medical Center, Boston, Massachusetts (Sandel); Department of Obstetrics and Gynecology, Boston Medical Center, Boston, Massachusetts (Iverson, Lee-Paritz); Division of Pediatric Allergy, Immunology and Pulmonary Medicine, Department of Pediatrics, Washington University School of Medicine, St Louis, Missouri (Strunk, Bacharier); St Louis Children's Hospital, St Louis, Missouri (Strunk, Bacharier); Department of Obstetrics and Gynecology, Washington University School of Medicine, St Louis, Missouri (Macones); Kaiser Permanente Southern California, San Diego, California (Zeiger, Schatz); Department of Pediatrics, Medical University of South Carolina, Charleston (Hollis); King's College London School of Medicine, Asthma, Allergy and Respiratory Science, Guy's Hospital Campus, London, United Kingdom (Hornsby, Hawrylowicz); Department of Population Medicine, Harvard Pilgrim Health Care Institute and Children's Hospital Boston, Boston, Massachusetts (Wu).

Author Contributions: Dr Carey and Mr Harshfield had full access to all of the data in the study and take responsibility for the integrity of the data and the accuracy of the data analysis.
Study concept and design: Litonjua, Carey, O'Connor, Strunk, Macones, Zeiger, Schatz, Hollis, Hawrylowicz, Weiss.
Acquisition, analysis, or interpretation of data: Litonjua, Carey, Laranjo, Harshfield, McElrath, O'Connor, Sandel, Iverson, Lee-Paritz, Strunk, Bacharier, Macones, Zeiger, Hollis, Hornsby, Wu, Weiss.
Drafting of the manuscript: Litonjua, Carey, O'Connor, Macones, Zeiger, Hollis, Wu, Weiss.
Critical revision of the manuscript for important intellectual content: All authors.
Statistical analysis: Carey, Laranjo, Harshfield, Wu.
Obtained funding: Litonjua, Carey, O'Connor, Strunk, Hollis, Weiss.
Administrative, technical, or material support: Litonjua, Laranjo, McElrath, O'Connor, Sandel, Iverson, Strunk, Macones, Zeiger, Hollis, Hornsby, Hawrylowicz.
Study supervision: Litonjua, Carey, O'Connor, Sandel, Strunk, Macones, Weiss.

Conflict of Interest Disclosures: All authors have completed and submitted the ICMJE Form for Disclosure of Potential Conflicts of Interest. Dr Litonjua reported receiving personal fees from UpToDate Inc and Springer Humana Press. Dr McElrath reported receiving grants from the National Institutes of Health (NIH). Dr O'Connor reported receiving grants from the NIH. Dr Bacharier reported receiving grants from the NIH and National Heart, Lung, and Blood Institute (NHBLI), and personal fees from Aerocrine, GlaxoSmithKline, Genentech/Novartis, Merck, Schering, Cephalon, DBV Technologies, Teva, Boehringer Ingelheim, AstraZeneca, WebMD/Medscape, Sanofi, and Vectura. Dr Zeiger reported receiving grants from the NHBLI, AstraZeneca, Aerocrine, MedImmune, Genentech, Merck, and

Copyright 2016 American Medical Association. All rights reserved.

Downloaded From: http://jamanetwork.com/pdfaccess.ashx?url=/data/journals/jama/934885/ by a Harvard University User on 03/30/2017

GlaxoSmithKline and personal fees from Genentech, Novartis, GlaxoSmithKline, and TEVA. Dr Hornsby reported receiving an NIH ancillary grant. Dr Hawrylowicz reported receiving an NIH ancillary grant, a fellowhip grant from Wellcome Trust Clinical Training Research Fellowship, grant G100758 from the Medical Research Council Centre, and grants from Asthma UK, the Lord Leonard and Lady Estelle Wolfson Foundation, and the Alpha 1 Foundation. No other disclosures were reported.

Funding/Support: VDAART was supported by grant U01HL091528 from the NHLBI. Additional support was provided by grant U54TR001012 from the National Centers for Advancing Translational Sciences (NCATS) for participant visits at the Boston Medical Center.

Role of the Funder/Sponsor: The NHLBI monitored the conduct of the trial and selected the membership of the data and safety monitoring board (DSMB). All communication between the investigators and the DSMB coursed through the staff of the NHLBI. The manuscript was presented to the DSMB for approval prior to submission for peer review. The NHLBI had no role in the design and the conduct of the study, in the collection, analysis, and interpretation of the data; or in the preparation, review, or approval of the manuscript, other than what pertained to the DSMB.

Disclaimer: Dr O'Connor, *JAMA* associate editor, was not involved in the editorial evaluation or decision to publish the article.

Data Coordinating Center: Augusto A. Litonjua, Scott T. Weiss, Vincent J. Carey, Nancy Laranjo, Benjamin J. Harshfield, Sharon O'Toole, Stacey Brown, Amali Chung, Sujata Datta, Caroline Holcomb, Sarah Kraft, and Bruce Hollis.

Clinical Centers: *Washington University at St Louis:* Robert C. Strunk (lead clinical center principal investigator [PI]), Leonard B. Bacharier (coinvestigator), Michael Nelson, Monica Anderson, Danae Larson, Yvonne Burrage, Jennifer Byers, Megan Isaac-Schmid; *Boston University Medical Center:* George T. O'Connor (clinical center PI), Megan Sandel (coinvestigator), Katherine Muse, Jessica Long, Erin Collins, Amanda Barbeau, Benvy Caldwell, Tawil Contreras, Corey Costanzo, Ingrid Gonzalez, Mahsan Mohammadi, Ashley Oliver, Penny Price, Chloe Sakow, Danuzia Silva, AneesaThannicka, Lena Wang; *Kaiser Permanente Southern California:* Robert S. Zeiger (clinical center PI), Michael X. Schatz (coinvestigator), Shawn Menafee, Diana Donofero, Kathleen Harden, Terrie Long, Travis Macaraeg, Maria Marcial, Elsa Rodriguez, Elizabeth Sanchez.

Data and Safety Monitoring Board: Lynn M. Taussig, MD (chair), Mitchell P. Dombrowski, MD, Carol L. Freund, PhD, Frank R. Greer, MD, Martin Hewison, PhD, Dennis R. Ownby, MD, Anthony Scialli, MD, Gerald Teague, MD, John N. Van Den Anker, MD, PhD, O. Dale Williams, PhD, Susan R. Hintz, MD, MS, and Jean Lowe, PhD.

Additional Contribution: We thank the participants of VDAART for their participation and contribution to the trial.

REFERENCES

1. Morgan WJ, Stern DA, Sherrill DL, et al. Outcome of asthma and wheezing in the first 6 years of life: follow-up through adolescence. *Am J Respir Crit Care Med*. 2005;172(10):1253-1258.

2. Strachan D, Gerritsen J. Long-term outcome of early childhood wheezing: population data. *Eur Respir J Suppl*. 1996;21:42s-47s.

3. Ginde AA, Sullivan AF, Mansbach JM, Camargo CA Jr. Vitamin D insufficiency in pregnant and nonpregnant women of childbearing age in the United States. *Am J Obstet gynecol*. 2010;202(5):436e1-436e8.

4. Zosky GR, Berry LJ, Elliot JG, James AL, Gorman S, Hart PH. Vitamin D deficiency causes deficits in lung function and alters lung structure. *Am J Respir Crit Care Med*. 2011;183(10):1336-1343.

5. Ferreira GB, Gysemans CA, Demengeot J, et al. 1,25-Dihydroxyvitamin D_3 promotes tolerogenic dendritic cells with functional migratory properties in NOD mice. *J Immunol*. 2014;192(9):4210-4220.

6. Xystrakis E, Kusumakar S, Boswell S, et al. Reversing the defective induction of IL-10-secreting regulatory T cells in glucocorticoid-resistant asthma patients. *J Clin Invest*. 2006;116(1):146-155.

7. Penna G, Amuchastegui S, Giarratana N, et al. 1,25-Dihydroxyvitamin D_3 selectively modulates tolerogenic properties in myeloid but not plasmacytoid dendritic cells. *J Immunol*. 2007;178(1):145-153.

8. Yurt M, Liu J, Sakurai R, et al. Vitamin D supplementation blocks pulmonary structural and functional changes in a rat model of perinatal vitamin D deficiency. *Am J Physiol Lung Cell Mol Physiol*. 2014;307(11):L859-L867.

9. Litonjua AA, Lange NE, Carey VJ, et al. The Vitamin D Antenatal Asthma Reduction Trial (VDAART): rationale, design, and methods of a randomized, controlled trial of vitamin D supplementation in pregnancy for the primary prevention of asthma and allergies in children. *Contemp Clin Trials*. 2014;38(1):37-50.

10. Dawson-Hughes B, Heaney RP, Holick MF, Lips P, Meunier PJ, Vieth R. Estimates of optimal vitamin D status. *Osteoporos Int*. 2005;16(7):713-716.

11. Ross AC, Manson JE, Abrams SA, et al. The 2011 report on dietary reference intakes for calcium and vitamin D from the Institute of Medicine: what clinicians need to know. *J Clin Endocrinol Metab*. 2011;96(1):53-58.

12. Ersfeld DL, Rao DS, Body JJ, et al. Analytical and clinical validation of the 25 OH vitamin D assay for the LIAISON automated analyzer. *Clin Biochem*. 2004;37(10):867-874.

13. Grebe SK, Singh RJ. LC-MS/MS in the clinical laboratory—where to from here? *Clin Biochem Rev*. 2011;32(1):5-31.

14. Litonjua AA, Weiss ST. Is vitamin D deficiency to blame for the asthma epidemic? *J Allergy Clin Immunol*. 2007;120(5):1031-1035.

15. Gern JE, Visness CM, Gergen PJ, et al. The Urban Environment and Childhood Asthma (URECA) birth cohort study: design, methods, and study population. *BMC Pulm Med*. 2009;9:17.

16. Oken E, Baccarelli AA, Gold DR, et al. Cohort profile: project viva. *Int J Epidemiol*. 2015;44(1):37-48.

17. Sun J. A non-parametric test for interval-censored failure time data with application to AIDS studies. *Stat Med*. 1996;15(13):1387-1395.

18. Fay MP, Shaw PA. Exact and asymptotic weighted logrank tests for interval censored data: the interval R package. *J Stat Softw*. 2010;36(2):1-33.

19. Miller RG Jr. *Survival Analysis*. 2nd ed. Hoboken, NJ: John Wiley & Sons; 2011.

20. Venables WN, Ripley BD. *Modern Applied Statistics with S-PLUS*. 3rd ed. New York, NY: Springer; 1999.

21. Dotterud CK, Storrø O, Simpson MR, Johnsen R, Øien T. The impact of pre- and postnatal exposures on allergy related diseases in childhood: a controlled multicentre intervention study in primary health care. *BMC Public Health*. 2013;13:123.

22. Stevens CA, Turner D, Kuehni CE, Couriel JM, Silverman M. The economic impact of preschool asthma and wheeze. *Eur Respir J*. 2003;21(6):1000-1006.

23. Martinez FD, Wright AL, Taussig LM, Holberg CJ, Halonen M, Morgan WJ; The Group Health Medical Associates. Asthma and wheezing in the first six years of life. *N Engl J Med*. 1995;332(3):133-138.

24. Bacharier LB, Guilbert TW. Diagnosis and management of early asthma in preschool-aged children. *J Allergy Clin Immunol*. 2012;130(2):287-296.

25. Ducharme FM, Tse SM, Chauhan B. Diagnosis, management, and prognosis of preschool wheeze. *Lancet*. 2014;383(9928):1593-1604.

26. Braig S, Brandt S, Wabitsch M, et al. Age-specific influence of wheezing phenotypes on pre-adolescent and adolescent health-related quality of life. *Pediatr Allergy Immunol*. 2014;25(8):781-787.

27. Edwards CA, Osman LM, Godden DJ, Douglas JG. Wheezy bronchitis in childhood: a distinct clinical entity with lifelong significance? *Chest*. 2003;124(1):18-24.

28. Schittny J, Burri P. Development and growth of the lung. In: Fishman A, Elias J, Fishman J, Grippi M, Senior R, Pack A, eds. *Fishman's Pulmonary Diseases and Disorders*. Vol 1. New York, NY: McGraw-Hill Professional; 2008:91-114.

Copyright 2016 American Medical Association. All rights reserved.

Downloaded From: http://jamanetwork.com/pdfaccess.ashx?url=/data/journals/jama/934885/ by a Harvard University User on 03/30/2017
