# Supplementary material for: Prenatal vitamin D supplementation reduces risk of asthma/recurrent wheeze in early childhood: A combined analysis of two randomized controlled trials
Source: PLoS One. 2017 Oct 27;12(10):e0186657. doi: 10.1371/journal.pone.0186657 (PMC5659607; doi:10.1371/journal.pone.0186657)
Supplement: S5 Text — (PDF) [file pone.0186657.s007.pdf]

Original Protocol

Downloaded From: http://jamanetwork.com/pdfaccess.ashx?url=/data/journals/jama/934885/ by a Harvard University User on 03/30/2017

DETAILED PROTOCOL – DATA COORDINATING CENTER
"Randomized Trial of Maternal Vitamin D Supplementation to Prevent Childhood Asthma (VDAART: Vitamin D Antenatal Asthma Reduction Trial)"

I. Background and Significance

Asthma is one of the leading causes of morbidity in children with 90% of all cases diagnosed by age 6[1, 2]. It remains the most common chronic disease of childhood in the world[3, 4], and incurs significant healthcare costs[5, 6]. The burden of the disease in both the developed and the developing world is significant and increasing rapidly with over 300 million people affected worldwide. Thus, preventing the development of this disorder would be of great public health importance.

Vitamin D deficiency has been documented in many populations worldwide[7, 8], and has been reported in healthy children, young adults (especially African Americans), and middle-aged and elderly adults[7, 8]. Vitamin D deficiency has occurred despite fortification of foods in some countries and despite intake of multivitamins containing vitamin D. This suggests that as countries adopt a western lifestyle, there is shift from outdoor activities to more time spent indoors. For example, it is estimated that in the US alone, Americans spend an average of 93% of their time indoors[9]. **Relevant to this proposal, pregnant and lactating mothers and their neonates are at especially high risk for vitamin D deficiency**[10-13]. Preliminary data from ongoing clinical trials show that many infants whose mothers are not being supplemented with *sufficient doses of* vitamin D have circulating 25(OH)D levels in the "insufficient" range (defined as levels between 10 - 25 ng/ml)[14]. Furthermore, it has recently been documented that 50% of mothers and 65% of their newborn infants from an inner city hospital were vitamin D deficient, with levels <12 ng/ml (or <30 nmol/L)[15], despite the fact that most mothers were taking their prenatal vitamins. This analysis also showed a strong positive correlation between maternal and newborn plasma levels, providing further evidence that infant vitamin D status is dependent on maternal vitamin D status. These results are similar to other recent reports that show a high prevalence of vitamin D deficiency in pregnant women and their newborns, even in some sun replete areas of the world. The prevalence of deficiency (25(OH)D levels < 10ng/ml among pregnant women in these studies were: 80% in Iran[16], 42% in Northern India[17], 61% in New Zealand[18], and 60-84% among non-Western women in the Netherlands[19].

The importance of vitamin D deficiency in pregnancy in relation to asthma development is that it has many recognized effects in on the developing lung and immune system. Nguyen and coworkers, through a series of studies in fetal rat lung, have identified the type II alveolar cells as a target for $1,25(OH)_2D_3$ action. Firstly, they identified specific binding sites (vitamin D receptors) for $1,25(OH)_2D_3$ that are distinguishable at a time corresponding to the start of type II pneumocyte differentiation and onset of surfactant secretion[20]. Next, with the use of monoclonal antibodies, they localized these vitamin D receptors to type II pneumocytes[21].Thirdly, in explanted fetal rat lung tissue, they showed that $1,25(OH)_2D_3$ accelerated the decrease in type II cells glycogen content and increased surfactant synthesis and surfactant secretion[22, 23]. Vitamin D was about half as potent as an equivalent dose of dexamethasone in stimulating surfactant synthesis and secretion. They also showed that a paracrine system for vitamin D is present in rat fetal lung and function during the last 3 days of pregnancy[21], and that some of the effects of vitamin D may be mediated by fructose 1,6 biphosphatase, whose gene contains a vitamin D response element in the promoter region[24]. **These recent observations strongly suggest that vitamin D plays a role in lung maturation late in pregnancy, and support earlier observations such as the association of respiratory distress in vitamin D deficient preterm infants ("rachitic respiratory distress")[25]. In humans, the effect of vitamin D on surfactant production has been confirmed[26], although the mechanisms appear to be more complex than in the rat[27].**

Downloaded From: http://jamanetwork.com/pdfaccess.ashx?url=/data/journals/jama/934885/ by a Harvard University User on 03/30/2017

Aside from effects on type II pneumocytes and surfactant production, vitamin D also appears to have effects on lung growth and development. Gaultier et al[28] studied lung mechanics in 50-day old rats born to mothers deprived of dietary vitamin D and reported significantly decreased lung compliance compared with rats born to mothers whose diet was supplemented with vitamin D, suggesting that disturbances in lung growth occurred in the vitamin D deficient rats. In humans, vitamin D also has been shown to play a role in the developing lung. Early studies used the presence of calbindin, a vitamin D dependent calcium binding protein, as a molecular marker of $1,25(OH)_2D_3$ action in tissues. Among many other tissues, Brun et al[29] reported high levels of calbindin in human fetal lung tissue at 14-32 weeks of gestation, suggesting that vitamin D plays a role in fetal lung development, as early as 14 weeks. Lunghi and colleagues[30] obtained normal human fetal (16 weeks gestation) lung fibroblasts and reported that in the presence of vitamin D, pyruvate kinase activity and lactate production of the cells increased. Other findings included a decrease in cell number and DNA synthesis in the vitamin D exposed cells compared with control cells. Subsequently, they showed that the vitamin D receptor was present in these human fetal fibroblasts[31] and confirmed their original findings in fetal lung fibroblasts in a subsequent study on senescent human lung fibroblasts[32]. **Thus, these animal and human tissue studies show that later in pregnancy, vitamin D stimulates lung maturation and surfactant production and secretion. Additionally, the data shows that at various points in fetal development, as early as 14 weeks of gestation, vitamin D has a regulatory role in fetal lung growth and development.**

We have recently shown in two independent birth cohorts that higher maternal intakes during pregnancy of vitamin D from both foods and supplements were associated with an almost 60% reduction of asthma and recurrent wheezing in 3-5 yr old children[33, 34].

We propose to conduct a double-blind, multicenter, placebo-controlled clinical trial of vitamin D supplementation in pregnant mothers to prevent the development of asthma and allergies in their children. The trial will be composed of a Data Coordinating Center and three Clinical Centers. The DCC will be composed of Brigham and Women's Hospital investigators led by Drs. Scott T. Weiss and Augusto A. Litonjua, and Dr. Bruce Hollis from the Medical University of South Carolina. **Members of the DCC will only see de-identified data and samples, and will not have any direct contact with recruited subjects. Members of the DCC will not participate in recruitment of subjects.**

II. Hypothesis and Specific Aims

Our hypothesis is that supplementing pregnant women with sufficient vitamin D will decrease the incidence of allergies, asthma and wheezing illnesses in children. The initial basis for this hypothesis is our finding in two separate birth cohorts of the protective effect of vitamin D intake on wheezing illnesses in 3-5 yr old children. Our birth cohort studies are consistent with data from studies that have investigated animal fetal models and human fetal lung tissue that show that (1) vitamin D receptors are present in the fetal lung, (2) vitamin D enhances lung maturation in the last stages of pregnancy, (3) vitamin D controls growth and development of the fetal lung, and (4) profile of human cord blood cell response to antigens is affected by vitamin D. Finally, there is a large amount of literature that shows that there is a high prevalence of vitamin D deficiency among women of child-bearing age and in pregnant women, such that infants are being born deficient in vitamin D. Furthermore, in both our birth cohorts, vitamin D intake in the first year of life and in childhood did not modify the association of maternal vitamin D intakes with any of the wheeze outcomes. Taken together, these data strongly suggest that the right time to intervene is during pregnancy. Therefore, we propose a two-arm, randomized, double-blind, placebo-controlled, multi-center clinical trial to determine whether vitamin D supplementation in the pregnant mother will prevent asthma and allergy at age 3. We will utilize asthma/recurrent wheezing as our primary end point and allergy and lower respiratory infection

Downloaded From: http://jamanetwork.com/pdfaccess.ashx?url=/data/journals/jama/934885/ by a Harvard University User on 03/30/2017

as secondary end points. We will recruit *870* pregnant women during the first trimester of pregnancy and randomize them to one of two treatment arms of a 5-year clinical trial: the addition of 4000 IU daily of vitamin D to usual pregnancy vitamin D supplementation (daily prenatal vitamins with 400 IU vitamin D) in the treatment group compared with placebo plus usual vitamin D supplementation (daily prenatal vitamins with 400 IU vitamin D) in the control group. Because we expect an 8% rate of miscarriages and stillbirths, and about *18*% withdrawals or loss to follow-up, we expect that we will have *660* children (*330* in each arm) for analysis of the primary outcome at 3 years of age.

Our primary aims are:

1. To determine whether a vitamin D dose of 4,000 IU per day vs the standard 400 IU/day in pregnancy is sufficient to maintain mothers' vitamin D levels in the immune-sufficient range of ≥ 30 ng/ml 25(OH)D.

2. To determine whether *sufficient* vitamin D supplementation in the pregnant mother is associated with reduced incidence of asthma in the child during the first 3 years of life. Our primary outcomes will be doctor's diagnosis of asthma and*/or* recurrent wheeze in the child at age 3 years.

In addition to our primary aims, we have the following secondary aims of the trial:

3. To determine whether *sufficient* vitamin D supplementation in the pregnant mother is associated with reduced secondary outcomes in the child of (a) allergic sensitization (total and specific IgE), (b) doctor's diagnosis of eczema and (c) lower respiratory tract infections during the first 3 years of life.

4. To determine whether *sufficient* vitamin D supplementation in the pregnant mother is associated with improved vitamin D status in their offspring through measurement of 25(OH)D levels in cord blood (at delivery), and children's blood (at 1 and 3 yrs of age).

III. Vitamin D supplementation: safety considerations.

The current adequate intake (AI) for pregnant and lactating women is 200 IU/day (although most prenatal vitamins contain 400 IU vitamin D). However, multiple studies have shown that these current recommendations result in a high degree of vitamin D deficiency in different adult populations[35-37]. One recent study supplemented 160 minority women in the UK with 800-1600 IU/day vitamin D throughout their pregnancies[37]. The investigators found a mean increase in circulating 25(OH)D concentrations from 5.8 ±0.9 ng/ml at the beginning of pregnancy to 11.2 ± 6.4 ng/ml at term after vitamin D supplementation. Therefore, mothers who were deficient at the beginning of their pregnancy were still deficient at the end of their pregnancy after supplementation, despite supplementation with a higher that recommended dose of vitamin D. A more recent study has supported these findings. Talwar et al [38] studied 208 postmenopausal African American women over 3 years. Their results showed that a dose of 2,000 IU/day raised 25(OH)D levels to 30 ng/ml in only 60% of the participants. This finding is relevant because pregnant women have greater vitamin D requirements (due to the growing fetus) than postmenopausal women. The results of these studies point out that the AI for vitamin D during pregnancy is grossly inadequate. **Given that a single minimal erythemic dose (MED) exposure (for a Caucasian individual, this is approximated by ~10-12 minutes of peak summer sun exposure) will release ~10,000 – 20,000 IU vitamin D into the circulation within 24 hours[39], the dose of vitamin D supplementation required to maintain adequate vitamin D status in adults is likely to be orders of magnitude greater than the current AI.**

Downloaded From: http://jamanetwork.com/pdfaccess.ashx?url=/data/journals/jama/934885/ by a Harvard University User on 03/30/2017

TABLE 1. Summary of high-dose vitamin D supplementation studies in healthy adults and pregnant and lactating women[*]. Adapted from Hollis and Wagner[10]

Reference†	Subject type	No. of subjects	Vitamin D dose	Therapy duration	Initial 25(OH)D	Endpoint 25(OH)D	Actual change in 25(OH)D
			IU/d	mo	ng/mL	ng/mL	ng/mL
**Supplementation in pregnancy**							
Brooke et al, 1980[40]‡	Pregnant Asians	67 Control<br>59 Supplemented	0<br>1,000	3	8.0<br>8.0	6.5<br>67.2	-1.5<br>+59.2
Cockburn et al, 1980[41]	Pregnant women	82 Control<br>82 Supplemented	0<br>400	4	13.0<br>15.6	13.0<br>17.1	0<br>+1.5
Delvin et al, 1986[42]	Pregnant women	13 Supplemented	1,000	3	13±4[5]	26±7	+13
Mallet et al, 1986[43]	Pregnant women	29 Supplemented	1,000	3	3.8±2.0	10.1±6.3	+6.3
Datta et al, 2002[37]	Pregnant minorities	80 Supplemented	800-1,600	>6	5.8±0.9	11.2±6.3	+5.4
**Supplementation in Lactating women**							
Ala-Houhala, 1985[44]	Lactating women	16 Supplemented<br>17 Supplemented	1,000<br>2,000	4.5	10<br>13	26<br>36	+16<br>+23
Hollis and Wagner, 2004[45]	Lactating women	9 Supplemented<br>9 Supplemented	2,000<br>4,000	3	27.6±9.8<br>32.6±6.9	36.1±7.0<br>44.5±11.4	+8.5<br>+11.9
Wagner et al, 2006[46]	Lactating women and infants	10 Supplemented<br>9 Supplemented<br>10 Supplemented	400<br>6,400<br>300	6	32.2<br>34.0	38.4<br>58.8	+4.2<br>+24.8
**Supplementation in non-Pregnant Healthy subjects**							
Vieth et al, 2001[47]	Healthy Males and females	10 M, 23 F<br>10 M, 23 F	1,000<br>4,000	5	16.3±6.2<br>18.7±7.1	27.5±6.8<br>38.6±5.8	+11.2<br>+19.9
Heaney et al, 2003[48]	Healthy males	67 Supplemented	200<br>1,000<br>5,000<br>10,000	5	28.0±9.4<br>28.8±6.4<br>27.7±6.7<br>26.2±9.7	23.0±7.1<br>33.6±6.5<br>64.5±15<br>90.0±25	-5.0<br>+4.8<br>+36.8<br>+63.8

[*] 25(OH)D, 25-hydroxyvitamin D.

† Sunlight exposure was not discussed in the studies by Brooke et al, Cockburn et al, Delvin et al, Mallet et al, and Datta et al. Minimal sunlight exposure was controlled for in the studies by Ala-Houhala, Vieth et al, Heaney et al, and Hollis and Wagner.

‡ It is likely that the wrong dose was reported. The response observed is one that would be expected after supplementation with 10,000 IU/d for 3 mos.

The safety of giving "large" (relative to current AI, but not to normal responses to sun exposure) doses of vitamin D and the potential risk of vitamin D toxicity are valid concerns. The exact amount of vitamin D required to induce toxicity in humans is unknown. The safe Tolerable Upper Intake Level (UL) for vitamin D was set at 2,000 IU/day by the Food and Nutrition Board (FNB)[49] in 1997. However, this recommendation was not based on current evidence, and is viewed by many as being too restrictive. Vieth[47] estimates that the physiologic limit for daily vitamin D intake is 10,000 – 20,000 IU/day, and toxicity has never occurred when physiologic amounts are ingested. The data on safety of vitamin D supplementation in physiologic doses and animal models of toxicity have been extensively reviewed by Dr. Hollis, who is a co-investigator on this proposal[10]. In this review, several studies of high-dose vitamin D

Downloaded From: http://jamanetwork.com/pdfaccess.ashx?url=/data/journals/jama/934885/ by a Harvard University User on 03/30/2017

supplementation (ranging from 400 IU/day to 10,000 IU/day) conducted in healthy adults and pregnant and lactating women were noted, and these studies are summarized in Table 1. It is noteworthy that none of these studies have reported adverse outcomes due to supplementation. Supporting the notion that high-dose supplementation is safe, Hathcock and co-workers[50] reviewed new evidence in addition to the evidence reviewed by Dr. Hollis in 2004, and applying the risk assessment methodology used by the FNB, concluded that the absence of toxicity in trials conducted on healthy adults supported the selection of 10,000 IU/day as the new daily UL for vitamin D. We should note here that we are proposing a dose that is less than half of this. Furthermore, as noted above, Dr. Hollis' group is conducting a Phase 3 trial of vitamin D supplementation of up to 4,000 IU/day during pregnancy (http://clinicaltrials.gov; RO1 HD 043921). Dr Wagner (Dr. Hollis' co-investigator) has generously supplied us the October, 2008 Data Safety Monitoring Committee (DSMC) report for their trial. **To date 347 women have completed the study and have delivered their babies and no episodes of vitamin D toxicity have occurred in the women, nor have adverse reactions been found in the fetuses or infants. The rate of spontaneous abortions, or other pregnancy related complications such as prematurity, have not increased in the vitamin D supplemented arms. Indeed in these subjects followed for 3123 person months of follow up, a total of 92 "adverse events" have been reported to the trial's DSMC. These events included diabetes (n=18), preeclampsia (n=5), gestational diabetes (n=17), hypertension (n=14), preterm labor (n=29), premature rupture of membranes (n=1), deep vein thrombosis (n=2), maternal infection (n=6), and placental abnormality (n=1). In detailed reviews, none of these pregnancy complications were attributed to vitamin D supplementation. Additionally, no neonatal physical abnormalities were detected on examination (C. Wagner, personal communication).** Therefore, the administration of 4,000 IU/day of vitamin D to pregnant women is safe. Dr. Hollis holds an IND with the FDA for safety of vitamin D supplementation in pregnancy (IND 66,346). He has shown that this dose is appropriate to bring levels in all of the women, regardless of race/ethnicity, to sufficient levels. Furthermore, the 4,000 IU/day dose eliminated all seasonal variation in 25(OH)D levels, which lower doses did not. His trials and data from others show that this dose is safe and not associated with any adverse effects in the mother or in the fetus/infant. This protocol will be covered under Dr. Hollis' IND.

Figure 1. Mean 25(OH)D levels in African American, Caucasian, and Hispanic pregnant women in the three supplementation groups.

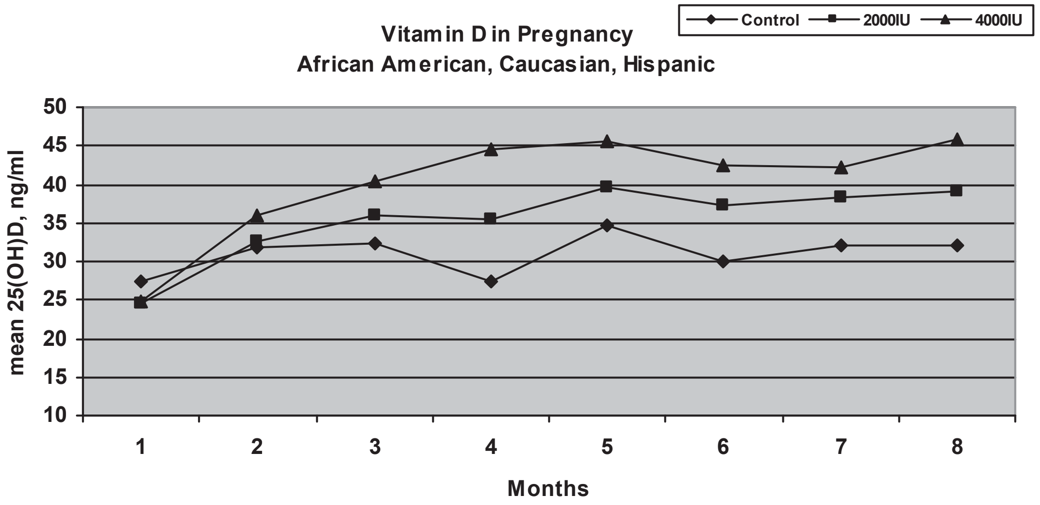

Dr. Hollis' trial has also generated more preliminary data regarding the response to the various doses of vitamin D. Figure 1 shows the response to varying doses of vitamin D in pregnant

Downloaded From: http://jamanetwork.com/pdfaccess.ashx?url=/data/journals/jama/934885/ by a Harvard University User on 03/30/2017

women from 3 ethnic groups: Caucasian (n=38 in Control group; n=37 in 2,000 IU, and n=39 in 4,000 IU), African American (n=24 in Control; n=35 in 2,000 IU; and n=26 in 4,000 IU), and Hispanic (n=45 in Control; n=48 in 2,000 IU; and n=44 in 4,000 IU). Baseline 25(OH)D levels varied by ethnic group, with African Americans having the lowest levels and Caucasians having the highest baseline levels. The 2000 IU/day dose raised the circulating 25(OH)D levels to a mean of about 38 ng/ml, and only approximately 60% achieved levels of >30 ng/ml. The 4000 IU/day dose most rapidly and consistently increased 25(OH)D levels in the participants from all ethnic groups, with a mean of about 45 ng/ml, and practically all participants achieved levels of at least 30 ng/ml. Additionally, the 4000 IU/day dose eliminated all seasonal variation in circulating 25(OH)D levels. There were no instances of adverse events from the 4000 IU/day dose, either in the mother or the infant, even in those mothers whose beginning 25(OH)D levels were > 30 ng/ml. **Thus, 4000 IU/day of vitamin D was the dose that achieved sufficient supplementation in the pregnant women. The preliminary results and experience from Dr. Hollis' clinical trials have provided valuable information for our current proposal, regarding the dosing and safety of vitamin D supplementation in pregnancy.**

IV. Study Overview and Subject Selection

This is a multicenter, randomized, double-blind, controlled clinical trial that compares high-dose vitamin D_3 supplementation (4,400 IU/day) vs. usual dose vitamin D_3 supplementation (400 IU/day) in pregnancy to prevent asthma in the women's children. The study base will be all pregnant women from each Clinical Center who have a personal history of asthma and/or allergies or a history of asthma and/or allergies in the biologic father of the child (i.e. a "high risk" cohort for asthma and allergies in the child). Seven hundred twenty (360 in each arm) will be followed through their pregnancy and delivery, and their infants will be followed up to the age of 3 yrs. *A*ll infants who are consuming < 1000 ml of formula per day will be offered vitamin D supplementation (400 IU/day) in accordance with the most recent recommendations from the American Academy of Pediatrics[51]*.* This includes all infants who are breastfed, either partially or exclusively, and all formula-fed infants until they consume at least 1000 ml of formula. The primary outcomes are doctor-diagnosis of asthma and recurrent wheezing at age 3 yrs. Due to dropouts from miscarriages, stillbirths, other pregnancy complications, and loss-to-follow-up, so we expect that we will be left with 660 3-yr old children (300 in each arm) for the analyses.

Women who come into their Obstetrician's clinics for their first pre-natal visit will be screened on a daily basis by Research Coordinator and a physician co-investigator will verify eligibility criteria.

Inclusion Criteria (applied to the pregnant women)

- Personal history of asthma, eczema, allergic rhinitis or a history of asthma, eczema, allergic rhinitis in the biological father of the child
- Gestational age between 10 and 18 weeks at the time of randomization
- Maternal age between 18 and 35 years
- Not a current smoker
- English- or Spanish-speaking
- Intent to participate for the full 4 years (through pregnancy and then until the 3rd birthday of the child)

Exclusion Criteria

- Not meeting Inclusion Criteria
- Gestational age >18 weeks
- Presence of chronic medical conditions: (i) Hypertension, (ii) Type 1 or type 2 diabetes mellitus, (iii) Parathyroid disease, and (iv) Uncontrolled thyroid disease.
- Taking vitamin D supplements containing > 2,000 IU/day of vitamin D3

Downloaded From: http://jamanetwork.com/pdfaccess.ashx?url=/data/journals/jama/934885/ by a Harvard University User on 03/30/2017

- Multiple gestation pregnancy (i.e. twins, triplets, etc.)
- Pregnancy achieved by assisted reproduction techniques (e.g. IUI, IVF)

Both arms of the study will get prenatal multivitamins which contain 400 IU vitamin D. Additional supplementation will occur in a double blind placebo controlled fashion with one arm receiving an additional 4000 IU of vitamin D (total Vitamin D 4400 IU daily), and the other arm receiving a matching placebo. We will not exclude subjects who have "normal" levels at the beginning of the trial, since many studies have now documented that a large proportion of pregnant women have insufficient levels. Even women who may have normal levels may have insufficient levels at other times during their pregnancy (i.e. during winter). The 4400 IU dose has been shown to remove seasonal variation in vitamin D levels.

V. Recruitment. The recruitment goal for each site will be *290* subjects. As we anticipate miscarriages and stillbirths (about 8%[52]), and a *17-18%* dropout by the end of the 3-year follow-up of the children, this number should give us the target of *220* 3-yr old children per Clinical Center or a total of *660* 3-yr old children at the end of the trial. The 3 clinical centers have considerable experience and a proven track record in recruiting participants for longitudinal studies of asthma. We expect that each center will be able to fulfill the recruitment goals in 9-12 months.

The study coordinator at each Clinical Center will obtain the schedules for all prenatal visits for the week, and scheduled visits will be reviewed for potential subjects. At their first prenatal visit, participants will be contacted by the Study Coordinator and interest in participating in the study will be assessed; if the participant is interested, then a screening questionnaire inquiring about a history of asthma or allergies in the patient or the biologic father of the child, a copy of the description of the study, and a consent form will be given to her. An enrollment/randomization visit will be scheduled within 1 week of the prenatal visit, where a review of the study procedures and consent form will be performed, and consent will be obtained. At that visit, a detailed symptom questionnaire, short food frequency questionnaire, and habitual sun exposure questionnaire will be administered, and determination of skin pigmentation will be performed. After randomization, the Study Coordinator will note the subject's scheduled visits with her obstetrician and will make sure that urine samples are collected at each of these scheduled clinical prenatal visits. At each of these clinical prenatal visits, a short maternal health questionnaire will be administered, MEMS cap information will be downloaded, and refill of the Study medication and prenatal vitamins will be perfomed. The Study Coordinator and RAs will also conduct a monthly review of electronic medical records to review any complications of pregnancy. A follow-up telephone call at 6 weeks post randomization will occur to promote adherence and to administer a maternal questionnaire that asks about over-the-counter medications, smoking, alcohol consumption, and symptoms. An in-person research prenatal visit will be scheduled at 6-8 weeks post-randomization for a blood draw, questionnaire administration, and refill of Study Medication and prenatal vitamins. Another follow-up telephone call will occur at 26 weeks to promote adherence. At delivery, an aliquot of blood drawn from the mother for clinical purposes will be retrieved, and cord blood will also be collected. After delivery, monthly calls to inquire about the health and symptoms of the infant will be done for the first 6 months. After the first 6 months, calls will become quarterly. Yearly visits for the child will also be scheduled. The questionnaires and measurements to be performed are summarized in Table 2.

Downloaded From: http://jamanetwork.com/pdfaccess.ashx?url=/data/journals/jama/934885/ by a Harvard University User on 03/30/2017

Table 2. Summary of questionnaires and measurements for the trial.

Variable	Enrollment 10-18 weeks gestation	Monthly	6-8 weeks after enrollment	28-32 weeks gestation	Delivery	Monthly	Quarterly	Year 1	Year 2	Year 3
Maternal Symptom Questionnaire (includes demographics)	x									
Food Frequency Questionnaire	x			x						
Sun Exposure Questionnaire	x			x						
Maternal Health in Pregnancy Questionnaire		x		x						
Electronic Medical Record Review		x		x						
Determination of skin pigmentation	X			x				x (mother and child)		x (mother and child)
Blood draw	x (mother)		x (mother)	x (mother)	x (Mother and cord blood)			X *(*mother and child*)*		x (child)
Urine for Ca/Cr ratio		x		x						
Delivery form					x					
Brief infant follow-up questionnaire						x (for the first 6 months)				
Follow-up questionnaire							x	x	x	x
In-person visit	x		x	x				x	x	x
MEMS information download		x	x	x	x (after delivery)					

<u>Screening visit:</u> This screening visit will occur at the subjects' first pre-natal visit. Each day, the study nurse/RA will review scheduled prenatal visits in the participating Clinical Center obstetric practices for the following day (Monday schedules will be reviewed the previous Friday), and will identify potential subjects who are presenting for their first prenatal visit. During the visit, the study nurse/RA will describe the study and will present the potential subject with a written description of the study and will review the eligibility criteria via a screening questionnaire. If the subject is eligible, does not meet any exclusion criteria, and expresses interest in the study, a

Downloaded From: http://jamanetwork.com/pdfaccess.ashx?url=/data/journals/jama/934885/ by a Harvard University User on 03/30/2017

follow-up visit will be scheduled within a week. At this point, the subject will be issued a unique screening ID, which will be attached to her pertinent screening information.

Enrollment visit: Within 1 week of the screening visit, but not later than the 22nd week of gestation, the study nurse/RA will meet with the subject, and the study description will be reviewed. The rest of the visit will entail completion of informed consent forms, completion of the health questionnaire, completion of the food frequency questionnaire, sun exposure questionnaire, and a blood draw for 25(OH)D measurement.

Randomization and Treatment Assignment: Participants who are deemed eligible to participate will be randomized by the Data Coordinating Center (DCC) to either of the 2 arms of the trial. Only those who firmly agree to follow-up until the child is 3 years old will be randomized.

Randomization will be performed prior to enrollment of any study participants by the DCC using a system that automates the random assignment of treatment groups to Study ID numbers. The randomization scheme will employ permuted blocks, and one blocked allocation list per stratum (study site and ethnic group) will be generated. That is, separate permuted block randomization lists will be maintained for each ethnic group (e.g., Caucasian/Non-Hispanics, Caucasian/Hispanic, African-American, and Other) within each study site. This will assure that within each site, there is approximate balance across arms over the course of enrollment, and that within ethnic groups within site, there is also approximately equal representation on both arms. A woman who is deemed eligible will receive the next available Study ID number for her given study center site and ethnic group. The Study ID will be linked to the women's Screening ID. It should be noted that women who are deemed ineligible will not receive Study IDs (i.e. randomization to a treatment), thus, this will help insure balance in treatment and control groups by not 'wasting' randomization assignments on ineligible subjects.

When a potential participant reports for the Enrollment Visit, informed consent will be obtained and a formal eligibility determination will be made. Women that consent and meet all eligibility criteria will be sequentially assigned a five-digit Study Identification Number (Study ID). The first digit of the Study ID will identify the site (St. Louis, San Diego, or Boston) and the next three digits will identify the participant; and the last digit will be a check digit that is calculated from a formula to insure the accuracy of data entry. Each Study ID number is pre-assigned a randomization assignment during the randomization process performed by the DCC before the study starts. The Study ID number assigned to a participant will determine treatment arm, and the study medication provided for a specific Study ID number will be dispensed to that participant. The schedule of visits, calls, and data collection points are summarized in Table 2.

VI. Data Collection

A. Screening Questionnaire. This short questionnaire will be administered at the screening visit at the time of the first pre-natal visit. This questionnaire will be used to obtain information on each participating woman and her husband/partner regarding demographics; physician-diagnosed illnesses; respiratory symptoms; smoking by members of the household; history of asthma, hay fever, eczema, or allergies; indicators of asthma severity; use of medications; and cigarette smoking. This questionnaire will also inquire about the diagnosis of asthma and allergies in the mother and the biologic father of the child, and whether the mother is willing to participate in the trial.

B. Maternal Behavior, Symptoms, and Medication Inventory

This questionnaire will be administered to the mother by the Study Coordinator at the scheduled telephone calls. The mother will be asked to complete this health assessment questionnaire to ascertain her:

a. Use of prescription medications
b. Use of over-the-counter preparations that may influence vitamin D and calcium homeostasis.
c. Use of cigarettes and alcohol

Downloaded From: http://jamanetwork.com/pdfaccess.ashx?url=/data/journals/jama/934885/ by a Harvard University User on 03/30/2017

d. Report of headaches—frequency and severity
e. Report of swelling of feet and hands
f. Employment during pregnancy: hours and type of work

C. Monthly Review of Medical Records (paper-based) and Electronic Medical Records (EMR)

EMR systems are in place at each of the clinical centers. Each month, the Study Coordinator or the RA will review the Obstetric EMR of each of the participants for any potential complication of pregnancy. Examples include but are not limited to the following: (a) Preeclampsia, (b) HELLP (Hemolytic anemia, Elevated Liver enzymes, Low Platelet count) syndrome, (c) Gestational diabetes, (d) Hypertension, (e) Preterm labor, (f) Premature rupture of membranes (PROM), (g) Deep venous thrombosis or a coagulopathy, (h) Maternal infection such as varicella or herpes (1° or 2°), (i) Placental abnormalities such as previa or marginal abruption. For centers where EMRs are not complete, paper medical records will be reviewed and the relevant information extracted.

D. Measurement of 25(OH)D

Blood will be drawn from the mothers at randomization, the in-person research prenatal visit, and at delivery. Cord blood will also be collected at delivery. Blood from the child will be collected at 1 yr and at 3 yrs. All specimens will be shipped to the DCC, where they will be logged in and processed. Aliquots will be sent to Heartland Assays, Inc from the DCC for measurement of 25(OH)D. Measurement of 25(OH)D will be performed as previously described[53]. Twenty five (25) microliters of serum or plasma (EDTA or Heparin) is required for the 25-OH-D extraction, however, 100 microliter samples will be sent to allow for duplicate measurements on each sample. The 25-OH-D assay consists of a two-step procedure. The first procedure involves a rapid extraction of 25-OH-D and other hydroxylated metabolites from serum or plasma with acetonitrile. Following extraction, the treated sample is then assayed using an equilibrium RIA procedure. The RIA method is based on an antibody that is co-specific for 25-OH-D2 and 25-OH-D3. The sample, antibody and tracer are incubated for 120 minutes at 20-25°C. Phase separation is accomplished after a 20-minute incubation at 20-25°C with a second antibody-precipitating complex. An NSB/Addition buffer is added after this incubation prior to centrifugation to aid in reducing non-specific binding. 25-(OH)D-equivalent values will be calculated directly by the γ-radiation counting system with use of a smooth-spline method. The results will be expressed in terms of 25-(OH)D equivalents.

In order to monitor assay performance each assay will include one in-house control sample. The control will be treated as unknown specimen and multiple (5) determinations will be made. Assay variation will be determined and acceptable performance limits will be defined as ≤15 C.V. In addition 20% of the samples will also be measured in Dr. Hollis' laboratory in the Medical University of South Carolina.

Heartland Assays, Inc, is located at the Iowa State University Research Park, Ames, IA, and is a joint venture between Dr. Bruce Hollis, Medical University of South Carolina and Dr. Ronald Horst, Adjunct Professor and Collaborator, Iowa State University, Ames IA[54]. Dr. Hollis and Dr. Horst have over 60 years of combined experience in the development and application of vitamin D assays in both human and veterinary medicine[54-58]. Each has made significant contributions to the assessment of vitamin D status as affected by a number of physiological states in humans and animals. Most notably and more recently has been Dr Hollis' determination that measurement of 25(OH)D is the best circulating biomarker to reflect vitamin D status[39, 59, 60]. Similar findings where reported by the Horst Lab regarding vitamin D status in poultry[61]. Dr. Hollis' laboratory has measured 25(OH)D for large epidemiologic studies dealing with pregnant women[10, 45].

Downloaded From: http://jamanetwork.com/pdfaccess.ashx?url=/data/journals/jama/934885/ by a Harvard University User on 03/30/2017

E. Urinary Ca^{++}/Cr ratios

At each monthly prenatal visit, mothers will provide a urine sample ("clean catch"). Because dairy products and higher calcium foods can affect urine calcium levels, mothers will be instructed to avoid dairy products in the hour prior to their scheduled visit. Urine samples will be sent to each Clinical Center's Clinical Chemistry Laboratory for urinary calcium and creatinine measurement, and the urinary calcium to creatinine ratio will be derived. Dr. Hollis has used this measure to monitor women on vitamin D supplements in his other trials and has shown that this is the earliest sign of impending toxicity.

F. Food Frequency Questionnaire. Maternal dietary intake of vitamin D will be assessed using a standardized, validated food frequency questionnaire[62]. This questionnaire has been slightly modified to apply to pregnant women and has been validated in pregnancy[63]. Mothers will be asked to complete the FFQ on enrollment in the 1st trimester. A second questionnaire will be completed at the 28-32-week visit. Assessment of the responses to these questionnaires will help account for dietary sources of vitamin D in addition to the study intervention. In addition, the FFQ will measure other nutrients that may have confounding effects, and which we will be able to adjust in the analyses. Filled out questionnaires will be sent to the Harvard Nutrition Questionnaire Services Center at the Harvard School of Public Health for scanning and processing. Individual nutrients will be calculated using the Harvard nutrient composition database, which contains food composition values from the US Department of Agriculture, supplemented by other sources[64].

G. Sun Exposure Questionnaire. We will collect UV exposure history using a modification of the UV exposure questionnaire that is used in the Nurses' Health Study[65]. Briefly, this questionnaire will inquire about UV exposure information such as average hours per week spent outdoors in summer and winter, sunscreen habits, and average times for sunlamp use or tanning salon attendance.

H. Measurement of Skin Pigmentation. Pregnant mothers will have the degree of pigmentation/melanin measured at study entry, at 28-32 weeks using the Smart Probe 400 (IMS, Inc., Milford, CT) and a skin tone chart (IMS, Inc.). This is a spectrophotometer device that measures degrees of pigmentation on a continuous scale from 0 to 100, 0 being absolute black and 100 being absolute white. Each mother will have pigmentation measurements recorded from her exposed forearm, underarm, and stomach, with two readings averaged and recorded.

I. Labor and Delivery Information and Questionnaires: At delivery, the research assistant will collect information on type of delivery (caesarian vs vaginal), birthweight and other anthropometric measurers, need of oxygen, etc. (see Appendix for Labor and Delivery questionnaire).

J. Infant Follow-up Questionnaires. Every month for the first 6 months after delivery, we will administer a questionnaire to the child's caretaker (in most instances, this will be the mother) to obtain information on the neonate general and respiratory health, with particular attention given to symptoms suggestive of asthma, wheeze, allergies, and atopic eczema. This questionnaire will also inquire about respiratory infections (upper, including ear infections, and lower tract infections, including bronchiolitis), medication use, particularly antibiotics, and smoking by adults in the home. Questions inquiring about breastfeeding, formula feeding, and introduction of solid foods will also be included. At the third month after delivery and every third month (quarterly) thereafter, the longer version of the questionnaire will be administered. In addition to the questions in the monthly questionnaire, this questionnaire will inquire about home characteristics, caregiver stress, pets in the home, and day care. At the first year questionnaire and each year thereafter, we will also inquire about the child's diet, using a short food-frequency questionnaire.

Downloaded From: http://jamanetwork.com/pdfaccess.ashx?url=/data/journals/jama/934885/ by a Harvard University User on 03/30/2017

K. Total and specific IgE: Total serum IgE levels will be determined in samples from the child (at age 3 year) by the UniCAP 250 system (Pharmacia & Upjohn, Kalamazoo, MI), with samples measured in duplicate. Serum from each subject will also be assayed for IgE to 11 allergens (dust mite [*D. farinae* and *D. pteronyssinus*], cockroach [*Bla g*], cat dander, dog dander, mixed grass pollen, mixed tree pollen, *Alternaria tenuis*, egg, milk, and peanut) using the UniCAP 250 system (Pharmacia & Upjohn, Kalamazoo, MI). For specific IgE, the enzyme-immunoassay is based on the sandwich technique, utilizing a solid phase (allergen-impregnated discs) for separation.[66, 67]

L. DNA Extraction and Samples for Gene Expression Studies: We will extract high molecular weight genomic DNA from the white blood cells from maternal peripheral blood and cord blood with commercially available PureGene Kits (Gentra Systems, Minneapolis, MN). Optical density (OD) will be measured to confirm DNA quality and quantity for each sample. This amount is sufficient to perform approximately 16,000 genotype assays using PCR technology (assuming a conservative use of 25 ng/reaction, although we routinely amplify single copy gene sequences from 10 ng of genomic DNA). Every sample undergoes additional quality control before use/release by subjecting it to PCR amplification. After DNA quantification, samples will be adjusted to TE buffer and partitioned into aliquots stored at -80°C. Information about quantity and storage of extracted DNA will be added as variables to each patient's database. DNA samples will then be stored at the Channing Laboratory for future genetic studies.

From the scheduled blood draws from the mothers and cord blood, we will include samples for gene expression studies. For mothers, two 2.5 PAXgene tubes and one 8.5 cc CPT tube will be drawn on entry into the study, 2 months post-enrollment, at the 30 week visit, at delivery, and at the child 1-yr visit. Two 2.5 cc PAXgene tubes and one 8.5 cc CPT tube will also be saved from the cord blood draw. These samples will be used to understand the genetics and gene expression of fetal development and asthma in future studies with a special focus on the relationship to vitamin D. While genetics and gene expression are not currently part of the specific aims of this protocol, it is imperative that samples be collected because the time window for appropriate collection is short (i.e. during pregnancy and delivery of the baby).

M. Manufacture and Distribution of Study Medications; The MEMS System

The study medications, including all prenatal vitamins, the vitamin D tablets, and the placebo tablets will be manufactured by Tishcon Corp. Founded in 1976, Tishcon Corp. (www.tishcon.com) is a leading manufacturer and marketer of vitamins, related dietary and herbal supplements, and private label non-prescription (OTC) pharmaceuticals in tablet, hard gelatin capsule, soft gelatin capsule and powder dosage forms. Tishcon is one of 12 soft gelatin capsule manufacturers in the United States. The pills and capsules will be sent in bulk from Tishcon to the DCC. The DCC will contract with the Brigham and Women's Hospital Pharmacy to package and label the pills in bottles with the MEMS caps. Once packaged and labeled with Study ID numbers, the pill bottles will be shipped to the respective Clinical Centers. Random pills from each lot will be sent to Dr. Hollis' lab where the vitamin D amount will be quantified for quality purposes. Each participant will receive 2 bottles – one bottle will contain the standard prenatal vitamins with 400 IU vitamin D, and will be labeled accordingly, and the other bottle will contain the intervention pill containing 4,000 IU vitamin D or a placebo.

The MEMS System. The MEMS® - Medication Events Monitoring Systems is a pill bottle equipped with special cap that includes an electronic microchip that stores the date and time of each opening of bottle. The MEMS monitors are drug packages with integral electronic micro circuitry designed to compile the dosing histories of ambulatory patients' prescribed medications. Each monitor consists of a conventional medicine bottle fitted with a special closure that records the time and date of each opening and closing of the container through integrated microcircuitry. The MEMS® stores up to 3800 medication events in non-volatile

Downloaded From: http://jamanetwork.com/pdfaccess.ashx?url=/data/journals/jama/934885/ by a Harvard University User on 03/30/2017

EEPROM memory, allows wireless data transfer, fits standard pharmacy bottles 38mm-400, 42mm-400, and 45mm-400 thread closure, provides 36 months battery life, and is water-resistant and CE marked. Monitors are designed to be used by one patient with one drug. A Reader allows transferring the dose timing data from the MEMS to a MS-Windows based computer. The MEMS monitors have demonstrated reliability and validity and have been widely used with great success in clinical trial research[68], including NHLBI sponsored studies in the Childhood Asthma Research and Education Network[69, 70] and asthma clinical research sponsored by industry[71]. The MEMS® - Medication Events Monitoring Systems is manufactured by Aardex LTD (http://www.aardexgroup.com) with a subsidiary in California for ease of ordering.

Data from the MEMS system will be downloaded monthly. The data from the pre-natal follow-up visits will be used to provide feedback to the participant, reinforcing those subjects with good adherence and providing extra teaching and encouragement for those with suboptimal adherence. These data can be used to derive a summary adherence measure that can be used as a covariate in our analyses and also for secondary analyses that exclude any women who have taken less than 50% of the prescribed study medication.

N. Statistical considerations

N.1. Overview

This is a randomized, double blinded, multi-center, placebo controlled, clinical trial of prenatal vitamin exposure as a preventive measure to reduce the risk of asthma and recurrent wheeze in childhood. There will be approximately *870* pregnant women enrolled, targeting an evaluable study population of *660* women randomized to Vitamin D supplementation of 4400 IU or 400 IU (standard prenatal supplementation). The treatment arms will be balanced (approximately n=300 in each arm, with some fluctuation possible owing to differential miscarriage or loss to follow-up, both expected to be completely at random). The primary outcome is doctor-diagnosed asthma and recurrent wheeze at 3 years of age. Secondary outcomes include allergy, doctor's diagnosis of eczema, and lower respiratory infection (LRI), at three years of age. The primary interpretive paradigm for analysis of this study is intent to treat.

N.2 Analytic plan

All analyses to be described will occur in a framework of continuous quality assurance and verification. Preliminary statistical analysis will be used to describe the univariate distributions of key measures of interest to detect outliers or data anomalies that need to be addressed by data editing or sequestration of doubtful entries. Additionally, these analyses will examine whether any important risk factors for childhood asthma are imbalanced across treatment arm, as well as assess whether differential loss to follow-up is present across treatment groups. Detailed blinded codebooks will be provided to investigators as data accumulate to facilitate efficient substantive verification of basic distributional summaries and joint distributions of measured factors.

N.2.a. Plan for *Specific Aim 1*

For this analysis, our N will be 800, since most of the dropouts will be expected to occur after delivery. Thus, the expected drop in N due to the 8% miscarriage rate was applied. We will test the hypothesis that the dose of 4,000 IU per day is sufficient to get 90% of the women to a sufficient level of 25(OH)D of at least 30 ng/ml, primarily using a logistic regression model, with randomization group (i.e. intervention group vs. usual care) as the independent variable. The dependent variable will be 25(OH)D level at delivery. We will adjust our analyses for other covariates that may affect 25(OH)D level, such as sun exposure (from the SmartProbe and the sun exposure questionnaire), age, and diet (calculated vitamin D intakes from dietary sources).

N.2.b. Plan for Specific Aim 2

The hypothesis that the incidence of asthma and recurrent wheeze at age three years is reduced in the high-dose Vitamin D supplementation group will be tested using logistic

Downloaded From: http://jamanetwork.com/pdfaccess.ashx?url=/data/journals/jama/934885/ by a Harvard University User on 03/30/2017

regression, allowing for fixed effects of center. Additional analyses will consider the modification of vitamin D exposure effects by maternal and child demographic/behavioral factors such as maternal age, BMI and weight gain during pregnancy, maternal history of asthma/allergy, and other factors potentially associated with asthma and/or Vitamin D exposure. The variables to be considered are summarized in Table 3. For variables that are measured post-natally, recall bias is a potential confounder among parents of infants diagnosed with asthma. However, the prospective nature of the data collection should limit the extent of this and other potential sources of bias. Due to the design of this clinical trial, we expect that the randomization process will distribute these variables equally between the intervention and control groups. However, we will check the distribution of these variables prior to conducting the analyses, and will adjust for variables that may be unevenly distributed across treatment and control groups.

Table 3. Information on selected potential confounders of the relationship between 25(OH)D levels and asthma/recurrent wheeze in 3-yr old children

Prenatal variables	Socioeconomic status
	Maternal diet
	Maternal sun exposure behavior
	Maternal age and parity
	Maternal history of asthma and allergies
	Maternal pre-pregnancy BMI and weight gain in pregnancy
	Maternal medication use in pregnancy (antibiotics, acetaminophen, NSAIDs, etc.)
	Pregnancy complications (e.g. gestational diabetes, pre-eclampsia)
Delivery variables	Gestational age
	Mode of delivery (caesarian section, normal vaginal delivery)
	Birthweight, length, head circumference
	Oxygen use
	Need for NICU stay
Postpartum and infancy variables	Breastfeeding or bottlefeeding
	Type of formula
	Timing and type of solid food introduction
	Daycare, including timing of and type of daycare (home vs institution)
	Pets in the home
	Other siblings in the home (gender and number)
	Smoking in the home
	Childhood diet
	Sun exposure behaviors in the child

Letting M_i be an indicator function for the primary endpoint, asthma, for the ith subject, the model is:

$$\text{logit } \Pr(M_i = 1 \mid x_i, a_i) = \alpha + \beta x_i + \gamma_1 a_{1,i} + \gamma_2 a_{2,i}$$

where x_i denotes an indicator function for level of vitamin D supplementation (0=400 IU, 1=4400 IU) in the ith subject, while $a_{1,i}$ and $a_{2,i}$ are indicator functions for study center. The specific aim for this outcome tests the hypothesis that β=0, asthma and recurrent wheeze incidence do not significantly differ across treatment groups, after adjusting for the potential effects of study center heterogeneity.

To assess modification of vitamin D exposure levels by other demographic and/or behavioral variables, the model is:

$$\text{logit} \Pr(M_i = 1 \mid x_i, a_i) = \alpha + \beta x_i + \gamma_1 a_{1,i} + \gamma_2 a_{2,i} + \sum_k \delta_k z_{ki} + \sum_k \eta_k x_i z_{ki}$$

Downloaded From: http://jamanetwork.com/pdfaccess.ashx?url=/data/journals/jama/934885/ by a Harvard University User on 03/30/2017

where z_{ki} represents the k measured covariates for each subject, and the other variables are as described above. This model addresses two hypotheses. First, main effects testing for behavioral/demographic covariates (Table 9), or the hypothesis that δ_k=0, will be used to examine whether maternal age, BMI, or other factors predict asthma and recurrent wheeze risk at age 3. More importantly, inclusion of any significant main effects will adjust for any distributional imbalances of these factors across the Vitamin D supplemented and placebo groups. Second, this model tests the hypothesis that η_k=0, or whether any maternal, child, or demographic/behavioral covariates modify the relationship between vitamin D intake and our primary outcome. To obtain a parsimonious model and limit multiple testing, only covariates with significant main effects and strong biologic plausibility for an exposure-dependent impact on asthma/recurrent wheeze risk will be considered for interaction testing with Vitamin D supplementation status. For example, only the prenatal and some delivery variables (e.g., gestational age, birthweight, length) listed in Table 9 will be considered, as the factors modifying the impact of Vitamin D supplementation on asthma and/or recurrent wheeze should occur temporally (i.e. in utero).

N.2.b.1. Interim Analyses for Specific Aim 2

For the primary aim of asthma and recurrent wheeze, we have developed an interim analysis plan to monitor this composite endpoint over the three years of subject follow-up. This sequential monitoring plan consists of two interim analyses (conducted at year 3 month 12 or when all first year study visit data are available and year 4 month 12/when all first and second year visit data are available), and a final analysis at the end of year 5, when the three-year follow-up has been completed on all subjects. For maximum flexibility in the analysis, we will use a Lan-Demets alpha spending function[72] with O'Brien-Fleming bounds. While the power simulations described in section O.2 use stopping boundaries that are designed for 3 'looks' over the study period, the Lan-DeMets procedure provides a spending function approach which does not require pre-specification of the frequency or timing of interim looks. This can be advantageous if the timing of data collection varies of if the need for more frequent monitoring arises. Conservative O'Brien-Fleming bounds are used to preserve most of the type-I error for the final analysis. The specific boundaries for the interim analyses are provided in section O.2. Only the primary endpoint of asthma and/or recurrent wheeze will be monitored sequentially. If the boundaries are crossed at either the first or second year analyses, results will be immediately reported and the study will be stopped.

N.2.c Plan for Specific Aim 3

Incidence of allergy, doctor's diagnosis of eczema, and lower respiratory tract infection (LRI) at age 3 will be individually assessed. Like asthma and recurrent wheeze, two of the three secondary outcomes are dichotomous endpoints, and will be analyzed using logistic regression. For allergy, logistic regression will be used for specific IgE and linear regression will be used for log transformed total serum IgE level. Both a primary analysis of the relationship between Vitamin D and the secondary outcome of interest will be conducted, as well as additional analyses including demographic and environmental risk factors for allergy, eczema, and LRI. The primary analysis, as well as additional analyses testing main demographic/behavioral effects and effect modification, will be conducted along the lines of analyses described for Specific Aim 2. The covariates to be considered for main effects and interaction analyses will be identical to those outlined for Specific Aim 2.

N.2.d. Plan for Specific Aim 4

This specific aim investigates the immediate and long-term impact of vitamin D supplementation on circulating 25(OH)D blood levels. The immediate impact of Vitamin D on 25(OH)D will be assessed through maternal plasma and cord blood measurements taken at

Downloaded From: http://jamanetwork.com/pdfaccess.ashx?url=/data/journals/jama/934885/ by a Harvard University User on 03/30/2017

delivery. The long-term influence will be examined via longitudinal analyses using children's blood measurements obtained at ages 1 and 3.

The hypotheses that 25(OH)D levels in maternal plasma and cord blood will be higher in the 4400 IU group will be tested using standard linear regression. If necessary, 25(OH)D levels will be transformed to meet the distributional requirements for the analysis. The 25(OH)D levels in maternal plasma and cord blood will be treated as separate outcomes, although both testing procedures for each analysis will be similar and are described jointly below. As with specific aims 1 and 2, the primary analysis will examine the relationship between 25(OH)D and Vitamin D levels, while the secondary analysis will include other important demographic and environmental factors related to 25(OH)D levels, such as climate and dietary vitamin D intake, testing for main effects and effect modification for these factors.

Letting y_i denote the 25(OH)D levels for each subject (as measured in mother's plasma for the first hypothesis, and cord blood for the second hypothesis), the model for the primary analysis is:

$$y_i = \alpha + \beta x_i + \gamma_1 a_{1,i} + \gamma_2 a_{2,i} + \varepsilon_i$$

where the covariates and parameters are as defined in Specific Aim 1. However, Specific Aim 3 tests the hypothesis that β=0, 25(OH)D levels do not significantly differ across the Vitamin D supplemented and control groups. All relevant covariates listed in Table 3 will be considered for the final model. For example, covariates measured post-natally would not be considered for inclusion in the analysis of maternal plasma or cord blood at delivery. Additionally, factors known to be directly associated with OH(25)D levels (i.e. sun exposure behavior, diet, ethnicity), will be included in the final model even if they are not statistically significant predictors of circulating vitamin D levels.

To assess modification of 25(OH)D levels by other demographic and behavioral variables, the model is:

$$y_i = \alpha + \beta x_i + \gamma_1 a_{1,i} + \gamma_2 a_{2,i} + \sum_k \delta_k z_{ki} + \sum_k \eta_k x_i z_{ki} + \varepsilon_i$$

where the parameters are as defined in Specific Aim 1. The k measured covariates for these analyses will be environmental, maternal, and child factors specific to 25(OH)D levels, such as sun exposure behavior, dietary intake of Vitamin D, and ethnicity. Additionally, any relevant covariates from Table 9 demonstrating main effects that have biologically plausible interactions with Vitamin D supplementation will be tested for effect modification. For both models, it is assumed that $\varepsilon_i \sim N(0,\sigma^2)$. The hypothesis that δ_k=0 will be used to examine whether any of these factors predict 25(OH)D levels, and the results will be used to adjust for any distributional imbalances of these factors across Vitamin D supplementation groups. As with the previous specific aims, we will also tests for effect modification via the hypothesis that η_k=0, or whether any maternal, child, or demographic/environmental covariates modify the relationship between vitamin D intake and circulating 25(OH)D levels in mother's plasma or cord blood. Additionally, we will follow the guidelines outlined in Specific Aim 2 to obtain the most parsimonious model.

The hypothesis that 25(OH)D levels in the child's blood will be higher in the 4400 IU group at birth, age 1 and age 3 will be tested using a repeated measures analysis framework such as generalized estimating equations.

N.3 Multiple comparisons.

All pre-specified hypotheses will be tested individually at the 0.05 level of significance, using a conservative two-sided alternative hypothesis (even when the stated hypothesis is one-sided). More specifically, the primary and secondary hypotheses of Vitamin D supplementation and Asthma/Recurrent Wheeze, Allergy/Exczema/LRI, will not be adjusted for multiple comparisons, since they have been defined a priori and are limited in scope. Additionally, an interim analysis plan has been developed for the primary outcome to adjust for the number of times (3) asthma and/or recurrent wheeze will be analyzed over the study period. Since

Downloaded From: http://jamanetwork.com/pdfaccess.ashx?url=/data/journals/jama/934885/ by a Harvard University User on 03/30/2017

25(OH)D status is being examined in 5 separate analyses (maternal plasma at 26-28 weeks, maternal plasma at delivery, cord blood at delivery, infant plasma at years 1 and 3), this analysis will be adjusted for 5 multiple comparisons (i.e., using Bonferroni correction). Finally, any findings with respect to interaction terms (i.e., exploring whether environmental and demographic covariates affect the relationship between Vitamin D supplementation and the outcome of interest) for any of the hypotheses will be adjusted (i.e., using FDR methodology) for the number of models tested, and interaction testing will be limited to those defined in sections D.14.2a-c, in order to limit the potential for spurious findings. Any serendipitous findings coming from exploratory analyses will be clearly reported as such, and test statistic p-values will be adjusted by the number of exploratory hypotheses considered (using FDR or another appropriate procedure for multiple comparisons adjustment).

O. Power and sample size

O.1. Power for Specific Aim 1. We will have excellent power to detect an increase of 20-60% in the proportion of mothers with 25(OH)D levels > 30 ng/ml between the intervention and the control group, as shown in Table . We expect that there will be 400 women in each arm for this analysis due to the 8% miscarriage rate. We do not expect there to be significant LTF by delivery.

Table 4. Power to detect a *20-60%* absolute increase in the proportion of mother's with *25(OH)D > 30 ng/ml* for *800* patients in a multi-site study*

	***Proportion of mothers with 25(OH)D > 30 ng/ml* in 4400 IU group**		
Proportion of mothers with 25(OH)D > 30 ng/ml in 400 IU group	***90%***	***80%***	***70%***
0.50	*>0.999*	*>0.999*	*>0.999*
0.40	*>0.999*	*>0.999*	*>0.999*
0.30	*>0.999*	*>0.999*	*>0.999*

* 8% miscarriage rate

O.2. Power for Specific Aim 2. The trial is designed to detect a *25*% reduction in asthma and recurrent wheeze incidence at 3 years in the vitamin D supplemented (4400 IU) group. Based on the observational results in the Viva Study, we estimate that the incidence of asthma and/or recurrent wheeze in the standard dosing group (400 IU) will be 40-50%. This incidence estimate is based on observations from Project Viva and the URECA Project**. In project Viva, the prevalence of persistent wheeze is 44% and of asthma is 56.2% in children born to families with parental history of asthma (about twice the prevalence in children without family history)**. These estimates are similar to those observed in the URECA Project in children who have turned 2 yrs old. The children in that cohort had a family history (mom or dad) of asthma or allergies. **In the URECA cohort, 99 of 194 (51.0%) children who have reached their 2nd birthday, have experienced "wheezing or asthma by age 2."** These observed rates in 2 cohorts give us confidence that we will see similar rates when we recruit the high-risk (for asthma and allergies in the child) pregnancies for this clinical trial. We anticipate an 8% miscarriage rate[52] and 17-18% LTF among remaining subjects over the study period, resulting in an estimated sample size of 660 subjects (330 per arm) at the end of the study period. Each power estimate is based on 10,000 simulations. The simulations incorporate the interim analysis plan described in N.2.b.1. For our group sequential design, we plan to use a Lan-Demets spending function with three equally spaced looks (with respect to the amount of

Downloaded From: http://jamanetwork.com/pdfaccess.ashx?url=/data/journals/jama/934885/ by a Harvard University User on 03/30/2017

information collected) and O'Brien-Fleming type bounds. The software program LANDEM (http://www.biostat.wisc.edu/landemets/)[73] was used to calculate the stopping boundar, which were |z|=3.7103, 2.5114, and 1.993 at the first, second, and third looks, respectively. The hypothesis tests are conducted with two-sided alternatives (i.e. a two-sided p-value), The power reflects the proportion of replicates where the Wald test statistic from the hypothesis test of β_1=0 is less than the stopping boundary specified for the first, second, or third looks, where β_1 is the coefficient for Vitamin D supplementation status from the logistic regression model outlined in section,D.14.2.a.i. The replication is considered a 'success' if the stopping boundary is exceeded at any of the analysis times. In the simulations, the incidence of asthma and/or recurrent wheeze at the first and second interim looks is proportionate to the overall incidence expected at the end of the study period (i.e. at the first look, the incidence is one-third of the specified rate; at the second look, it is two-thirds). We do not expect incidence rates of 40-50% to be achieved until the third year of follow-up; therefore, the simulations reflect the approximate amount of information anticipated at each interim analysis.

Finally, in the simulation studies, we also incorporate study heterogeneity into the model by varying the incidence of asthma and/or recurrent wheeze in the control group (400 IU Vitamin D) across study sites. The incidence varies by an absolute difference of 10% (i.e., for a mean incidence of 50%, incidence rates varied from 45-55% across study sites). Thus, the absolute reduction in incidence observed in the treated group (4400 IU Vitamin D) will also vary according to baseline incidence in the control group [i.e., assuming a 30% reduction in incidence, a site with a 50% incidence rate in the control group will have a 35% incidence rate (a 15% decrease) in the treated group, while a site with a 45% incidence in controls will have a 31.5% incidence rate (a 13.5% decrease) among supplemented subjects].

Table *5*. Power to detect a *20-30*% reduction in asthma and recurrent wheeze for *660* patients in a multi-site study*

	Percent reduction in Asthma and/or Recurrent Wheeze in 4400 IU group		
Mean Incidence of Asthma and/or Recurrent Wheeze in 400 IU group	***30%***	***25%***	***20%***
0.50	*0.974*	*0.906*	*0.726*
0.45	*0.950*	*0.837*	*0.653*
0.40	*0.901*	*0.773*	*0.574*

*18% LTF and 8% miscarriage rate

We anticipate that there will be excellent power *(91%)* to detect a *25*% reduction in asthma, assuming a *50*% incidence of asthma/recurrent wheeze in the 400 IU dosing group. Even if the children in this study have a lower incidence of asthma and recurrent wheeze than previous cohorts (i.e., 40-45%), we will still have good power (77-84%) to detect a 25% reduction in asthma and/or recurrent wheeze. To assess the potential impact of study center heterogeneity and interim analyses on power, powercalculations were conducted without adjustment for study-center and with a single analysis time point at the end of the study period (with a two-sided alpha=0.05). The calculations were performed in STPLAN (http://biostatistics.mdanderson.org), using an arcsine approximation for the binomially distributed outcome. Overall, the power estimates were consistently similar to the empirical estimates (shown in Table 5), with at most a 2% difference in absolute power. Therefore, we do not anticipate that multiple study sites or incorporating interim analyses will considerably reduce study power.

O.3. Power for Specific Aim 3. The estimated incidence of doctor's diagnosis of allergy (IgE), eczema, and and lower respiratory infection (LRI) at year 3 in the Viva cohort are 30%, 36%,

Downloaded From: http://jamanetwork.com/pdfaccess.ashx?url=/data/journals/jama/934885/ by a Harvard University User on 03/30/2017

and 45%, respectively, among the children with a parental history of asthma or allergies. Estimates of eczema and LRI differ from those submitted in the previous grant application, which were based on incidence of these secondary outcomes among all children (including those without a parental history of asthma and allergies). Additionally, information on allergy incidence in Project Viva is now available. We anticipate a 30%-*45*% reduction in each of these outcomes in the high-dose Vitamin D supplementation (4400 IU) group, though a larger range is provided in table 6 below. Each power calculation is based on 10,000 simulations, and are identical to the simulations described above, except that interim analyses are not planned for the secondary outcomes, and the studies are powered to detect an effect at the end of the three year follow-up period, with a two-sided alpha level of 0.05. As with the methods described above, these simulations incorporate an effect for study center heterogeneity in baseline incidence of secondary outcomes (i.e., incidence across sites varies by 10%) and 8/18% losses to miscarriage and follow-up/miscarriage, respectively.

With a 30% reduction in incidence, we anticipate that there will be good power (83.4%) to detect a clinically important difference in risk of eczema. Due to the lower incidence of allergy,a 35% reduction is required to have very good (89.0%) power. Additionally, since LRI is more prevalent at age 3, only a 25% reduction is required to have 85.8% power. Even if the incidence of these outcomes is 5% lower than the estimated rates in Viva, there will still be sufficient power to detect an effect of treatment. Overall, there is strong evidence that we will be adequately powered to detect a risk difference intreatment group (4400 IU Vitamin D) for the secondary outcomes, in addition to our primary outcome of interest, asthma and recurrent wheeze.

Table *6*. Power to detect a *25-35%* reduction in *secondary outcomes for 870 subjects in a multi-site study, (effectively 660 patients, assuming an 8% miscarriage rate and 18% loss to follow-up)*

Mean Incidence of secondary outcome in 400 IU group	***Percent reduction in secondary outcome in 4400 IU group***		
	35%	***30%***	***25%***
0.50	*0.998*	*0.982*	*0.913*
0.45	*0.992*	*0.958*	*0.858*
0.40	*0.973*	*0.906*	*0.779*
0.35	*0.936*	*0.845*	*0.691*
0.30	*0.890*	*0.736*	*0.608*
0.25	*0.795*	*0.654*	*0.531*

O.4. Power for Specific Aim 4. The research to date on the impact on Vitamin D supplementation of 25(OH)D is presented in Table 1. As noted in the table, only 4 studies have been conducted on pregnant women, all had fewer than 100 subjects, and the maximum daily dose for these studies was 1,600 IU. The only data available on 25(OH)D status in the supplementation ranges we are proposing has been provided by Dr. Bruce Hollis (unpublished data, see section III). In a study of 400 pregnant women (African Americans. Hispanic, and Caucasians) on either 4000 IU (supplemented) or 400 IU (control) of Vitamin D daily, the maternal plasma 25(OH) D levels were 46 ng/mL in supplemented subjects, and 32 ng/mL in controls after 8 months of supplementation, an overall difference of 14 ng/mL. The estimated standard deviation for 25(OH)D levels in these ranges is 16.

Based on the observed differences/variation in 25(OH)D levels, we used simulation studies to estimate the power to detect a mean difference of 3-15 ng/mL across the supplemented and control groups in maternal plasma at 26-28 weeks, as well as maternal plasma and cord blood at delivery, using an estimated standard deviation of 16. We anticipate that the difference in 25(OH)D levels in maternal plasma across treatment groups will less pronounced at the 26-28 week measurement period (e.g., 5-10 ng/ML), and we do not expect to observe a significant difference by supplementation status in circulating vitamin D in infants at 1 and 3 years of age, since no post-natal treatment intervention is planned. The simulations were generated in a similar manner to method described for Specific Aim 2, except that linear regression (rather than logistic) was used to model the relationship between circulating Vitamin

Downloaded From: http://jamanetwork.com/pdfaccess.ashx?url=/data/journals/jama/934885/ by a Harvard University User on 03/30/2017

D and Vitamin D supplementation status. Study heterogeneity was incorporated into the model by allowing OH(25)D levels to vary by 10 ng/mL across study sites. Since this phenotype will be measured five times throughout the studyperiod, the power was defined as the proportion of replicates with a Wald test statistic (i.e., testing the null hypothesis that the coefficient for Vitamin D supplementation status is 0) 2-sided p-value was less than 0.01 (0.05/5). The results are shown in Table 7.

Table 7. Power to detect a 3-15 ng/mL difference in 25(OH)D for 870 subjects in a multi-site study, (effectively 660 patients, assuming an 8% miscarriage rate and 18% loss to follow-up).

Mean change in 25(OH)D levels in 4400 IU group (ng/mL)	Power
3	0.444
5	0.925
10	0.999
15	0.999

For a mean difference greater than 3 ng/mL, we anticipate having excellent power to detect a difference in 25(OH)D status across treatment groups. This difference of at least this magnitude should be observed in both maternal plasma measurements and cord blood, given the preliminary data (a difference of 14 ng/mL in maternal plasma) provided by Dr. Hollis. To ensure that the results were not affected by incorporating an effect for study center, the simulations were conducted assuming homogenous effects across study sites. The power estimates were within 1.5% of those provided above, and are likely due to variation in estimates via simulation. Therefore, we are confident that we have excellent power to achieve this specific aim.

O.5 Missing data considerations

It is expected that missing data will be infrequent, that the treatment is sufficiently benign that any missingness would be unrelated to treatment group, and that the outcome ascertainment process is compatible with standard medical care for children. Thus it is reasonable to predict that missing data would be missing “completely at random” (MCAR) and that inference based on the complete cases would be valid, though only slightly inefficient, if missingness is rare (less than 5%) . Methods are available to assess the compatibility of response patterns with the MCAR condition (Chen and Little, 1999), and these will be used to diagnose the missing data situation. If there missingness is not rare, and it is desired to improve the efficiency of the analysis, two possible approaches to handling the imputation are described below.

Approach A: Impute the overall (ignoring treatment assignment) incidence of asthma and recurrent wheeze (or the incidence of the secondary outcome of interest) for children that are lost to follow-up, and analyze the resulting imputed dataset as if complete. This is a crude but very simple approach that is often proposed. Approach B: Use subject-specific modeling to determine an imputation distribution for those who drop out. Specifically, model the risk of asthma and recurrent wheeze (or secondary outcome) among completers as a function of baseline covariates, ignoring treatment assignment. Use the covariates z_i observed on dropouts to predict logit[Pr($M_i|z_i$,)]. The predicted estimate is then perturbed by repeatedly drawing randomly from a distribution whose variance is the sample variance in the group of completers. Both analyses will be conducted to examine the similarity of results to the complete case analysis. If the results are consonant, for simplicity, the complete case analysis will be reported along with any variations in findings from the described imputation strategies.

Downloaded From: http://jamanetwork.com/pdfaccess.ashx?url=/data/journals/jama/934885/ by a Harvard University User on 03/30/2017

VII. Study Implementation and logistics

A. Clinical Center Organizational Structure and Infrastructure

Each Clinical Center has a set organization structure with clearly defined personnel roles, a well-maintained computer infrastructure, and plans to establish numerous mechanisms to promote the flow of information between study center sites and the DCC.

B. CCC Organizational Structure

Dr. Strunk and colleagues at the Washington University in St. Louis, MO has been designated as the lead Clinical Center for this grant. Dr. O'Connor at Boston University Medical Center and Dr. Zeiger at Kaiser Permanente and University of California at San Diego are the PIs of their respective Clinical Centers. Dr. Strunk will schedule and lead regular conference calls to discuss the processes of recruiting, adherence, and retention throughout the trial, and will report directly to Dr. Weiss at the the DCC.

Because recruitment of participants will take place in Obstetrics Clinics, it will be crucial for each Clinical Center to establish ties and obtain cooperation from their respective Obstetric practices. All three centers have working relationships with the Obstetric practices from prior recruitment efforts. In the St. Louis Center, Dr. George Macones will support the trial by setting into place the system to recruit the pregnant women and obtain the necessary samples of peripheral blood during the prenatal visits and peripheral and cord blood at the time of delivery as well as obtaining urine to be measured for creatinine and calcium as safety for vitamin administration during pregnancy. Dr. Macones will assure that the OB practitioners are engaged in the trial and will support enrollment of their patients into the trial. Dr. Linda Heffner, MD, PhD (Chair, Dept. of OB and Gyn) and Ms. Margaret Shepherd, CNM (OB Department Manager), will fulfill the same roles for the BUMC and Kaiser Permanente San Diego Clinical Centers, respectively.

In each Center, a Research Patient Coordinator will be responsible for recruiting pregnant women into the trial at the time of their first visit to the OB offices and retaining them during the 4 years of their involvement in the trial. Specific activities of this Patient Coordinator will include but not be limited to:

- Working with the clerical staff of the OB department to assure that a brochure describing the vitamin D trial is included in the materials sent to the pregnant women giving information about the first OB visit.
- Working with the staff at the OB offices to determine when eligible women will have their first OB visit and being in the clinic to meet the women to give them information about the study, determine their level of interest, perform appropriate screening, give the informed consent and arrange a time for discussion of the trial and the consent and answer any questions.
- Make an appointment in the GCRC or the Allergy Research Department for enrollment and initiation of the study medication. The trial will also provide standardized prenatal vitamins, which will be initiated at this visit.
- Remain in contact with the scheduled phone calls and as needed for problem solving about taking study medication and the prenatal vitamin.
- Work with the staff in labor and delivery to establish as system for collection of peripheral blood from the mother to be used for assay of vitamin D and the cord blood for assay of vitamin D and for use in extraction of DNA to be used in the genetic studies.
- Visit the now new mother and her baby after delivery to deliver a small gift and further develop the relationship that will assure long-term follow-up of the child with the quarterly phone calls and annual visits.

Each Center will also have a second Research Patient Coordinator or Study Nurse who will be a partner of the other research patient coordinator in performing all the duties listed to assure recruitment and retention of the women and their offspring.

Downloaded From: http://jamanetwork.com/pdfaccess.ashx?url=/data/journals/jama/934885/ by a Harvard University User on 03/30/2017

C. DCC Organizational Structure and Infrastructure

The DCC has a set organization structure with clearly defined personnel roles, a well-maintained computer infrastructure, and plans to establish numerous mechanisms to promote the flow of information between study center sites and the DCC.

D. DCC Organizational Structure

The DCC will be chaired by Dr. Scott Weiss and Dr. Augusto Litonjua, and the statistical and informatics component will be managed by Dr. Vincent Carey. The DCC informatics operations will be executed by Dr. Carey's staff, who have an outstanding track record running multicenter trials funded by AHRQ and NHLBI since the mid 1990s. Nancy Laranjo will be the Senior Data Manager for the trial, and she will be supported by Melissa McEnery, Deputy Data Manager, John Ziniti, Senior Programmer, and Shelly Magno, Administrator. In consultation with Dr. Carey, Ms. Laranjo will specify the general communications and data archive workflows for the support and programming staff members. As the design of forms and general data flow processes evolves, prototypes and "dry run" exercises will be carried out with the clinical sites to verify the soundness and feasibility of the operations plan. The detailed task sets will be noted in the DCC Manual of Operations, which will be available through the internal study web site. The DCC will have full staff meetings attended by one of the PIs every month, and basic staff meetings every week. In addition, the chairs of the DCC will have a conference call with the PIs of the Clinical Centers once a month to obtain updates on recruitment and progress of the trial, and discuss any issues that pertain to the conduct of the trial.

E. Computing facility and software description

The clinical centers will be informatically supported by the DCC at the Channing Laboratory.

F. Trial Website

Each Center's participants will have access to the trial website. The DCC will establish a website with both public and private (SSL regulated, LDAP database of all study investigators or support staff needing website access will be established with private passwords) web portals at the beginning of the recruitment period. The public pages will provide information on the scope and background of study to assist in recruitment. Additionally, centers will be given links to be able independently generate and update region-specific (i.e., center directions and contact information) pages. The private pages will allow for general posting of any protocols changes or announcements, current versions of forms, minutes of phone calls, manuscripts in development, allow investigators to monitor recruitment process, and provide other general information. Patient-level treatment information, medical history, or endpoint information will not be linked to the website. The website will be maintained throughout the trial and closeout period, and aggregate study findings will be posted at the end of the trial.

G. Data management and tracking

Clinical data from screening (first-prenatal visit), each subsequent pre-natal visit, delivery day, quarterly follow-up questionnaires, and clinic assessment visits at 1, 2, and 3 years are entered on forms at each study center at the time of the patient visit/contact. The forms will be reviewed by the center coordinator, and copies sent weekly to the data monitor at the DCC. We have chosen this method for data entry, as this is easily auditable and cheap. We have the capability to set up a web-based data-entry for each Clinical Center, as investigators at the DCC have done in their previous projects and we will consider the feasibility and cost of this alternate approach.

Downloaded From: http://jamanetwork.com/pdfaccess.ashx?url=/data/journals/jama/934885/ by a Harvard University User on 03/30/2017

Calcium/creatinine ratios will be conducted by the study center laboratory on the day of the patient visit. The calcium/creatinine ratios will be reviewed by the laboratory head at each center. Alert values will be established and if reached will lead to immediate contact to the Study Coordinator and contact with the participant will be attempted within 24 hours of identification. Blood draws for 25(OH)D levels will be sent from the study center laboratory to the DCC on a weekly basis, where they will be logged in and aliquots sent to Heartland Assays, Inc for 25(OH)D assays.

Upon receipt on collected forms, all data forms (lab work and patient questionnaires, clinical assessments, etc.) will be double entered at the DCC with range, logic, and missing data checks built into the data entry software application. The data entry software will be configured to be schema-driven, so that an abstract schematic design is at the base, and the concrete (XML) schema that specifies form content and logic is automatically transformed to a functional data entry application. We currently use Altova XMLSpy and Microsoft InfoPath technologies to implement the schema-driven design. Forms with missing (illegible entries will be considered missing for the purposes of data entry) and/or inaccurate (i.e. out-of-range) entries will not be accepted into the system database, but will be cycled back to the site for correction.

For clinical data, the data monitor will send weekly reports of missing or inappropriate entries to the study project coordinators for clarification and resolution. Additionally, cross-form edit checks will be performed on a monthly basis, to detect unresolved problems. Data inconsistencies occurring across forms will be resolved with the assistance of study center staff. Standardized edit reports that summarize problems in the database provide an additional method of assuring data quality. To ensure accuracy of patient identification numbers (in particular, minimizing transposition error), digit checks will be utilized. Digit checks will identify whether there are inconsistencies in patient identification numbers across collection time points. Once data have been verified, data will be archived on a monthly basis as a SAS dataset. Data will be stored in coded form (i.e. numeric coding for ethnicity or gender) using standard codebooks currently implemented

For each patient, a data collection tracking query will be developed, which will contain expected time points for each data form/lab work, based upon patient's initial enrollment date. Forms more than one week late (from enrollment to delivery) or one month late (after delivery to year 3) will be flagged as missing, and a query will be generated for each site that contains a list of patient IDs and their outstanding forms/lab data. The data manager at the DCC will follow-up with site coordinators to track down any missing forms. For patients at-risk of dropping out (i.e. missed appointment or non-response to questionnaire request), the study coordinator or research assistant will make repeated attempts to contact the patient. A subject will be dropped only with explicit withdrawal request by the pregnant woman (initially) and later, the parent of the infant/child. Even with missed appointments or calls, every effort will be made to complete the final in-person visit when the child is 3 yrs old.

H. Data Security and Confidentiality

All computer files are stored in password-controlled accounts on DCC computers. There will be an SSL regulated private web portal established for the study at the DCC, LDAP database of all study investigators or support staff will be established with private passwords allocated to all who have a need to examine the study web site

Each participant will be assigned a unique study identification number. Once these data are linked (after a subject successfully enters randomization phase from screening), the study identification number will be the primary identification number. The data coordinating center and trial statisticians will not have access to only the unique identification number and therefore cannot link names to data. Files of study data will include the *screening and* study identification number but not participant names. Any hardcopy of data forms obtained by the coordinating

Downloaded From: http://jamanetwork.com/pdfaccess.ashx?url=/data/journals/jama/934885/ by a Harvard University User on 03/30/2017

unit (for example, as part of spot-checking for quality control) are will be maintained in locked file cabinets.

At the clinical centers, data are collected on forms with fields for study identification number but without a field for the participant's name. Still, separate lists of participant names, identification numbers, and contact information are used during the study to maintain contact with participants and eventually to provide feedback on study and personal results. These lists will only be available to Center Co-PIs and staff with direct patient contact. All hardcopies of forms are stored in locked file cabinets. All computer files are password protected. Participant results will only be released to the participant unless he/she provides written approval to release data (e.g. laboratory results to personal provider). Data will be only be presented and published in aggregate, i.e. no identifying characteristics of participants will be published or presented.

I. QC protocol

To ensure uniform data collection and reporting procedures, training and certification of study center research assistants (RAs) will occur at the DCC. Additionally, there will be a weekly conference call between the DCC deputy data manager and the site coordinators at each center to globally address any screening or data collection issues and maintain study-wide consistency in procedures. Monthly conference calls will be conducted between the DCC PI and all site investigators to check progress and data integrity. Patient recruitment and follow-up, as well as data collection and archiving procedures, will be monitored by the DCC with bi-annual site visits. 20% of the patient questionnaires and lab forms will be randomly sampled and queried against stored values in the DCC database and archives.

To verify the accuracy of 25(OH)D measurements obtained by Heartland Assay, Inc., 20% of the maternal blood samples (obtained at baseline and week 30), as well as maternal and cord blood samples at delivery, will also be sent to Dr. Hollis' lab. Any discrepant lab values will be checked to determine the source of the variation. If appropriate, a replicate assay will be conducted. Samples will also be obtained from each lot of the Vitamin D supplement (4000 IU Vitamin D) and prenatal vitamins (400 IU Vitamin D) and sent to the lab of Dr. Hollis for analysis to ensure the level of Vitamin D compound in each is with the range specified in trial protocol.

The DCC will provide monthly reports on the timeliness, quality and completeness of the data to the Steering Committee, organized by type of visit, e.g. screening visit 1, randomization day, 6-month visit, etc, and by specific data form. The Steering Committee will remain informed of any potential screening or data collection issues (with patient forms or lab work) and be able to recommend protocol changes or study center intervention as necessary to resolve any outstanding concerns.

J. Blinding

Until the end of the trial, all investigators, clinical staff and participants are masked to trial outcome data, with the exception of the trial statisticians, the data manager, and the Data, Safety and Monitoring Committee. Due to the nature of the intervention, laboratory technicians and staff obtaining calcium/creatinine ratios and 25(OH)D levels may be able to infer treatment group assignment. However, 25(OH)D levels will be measured by Heartland Assays, Inc. in Ames, IA, and results of these assays will be sent only to the DCC. Therefore, we do not anticipate any issues with unintentional unblinding of study investigators or clinical staff through routine contact between Heartland Assays and study sites. For quality control purposes, 20% of the blood samples will also be sent to Dr. Hollis' lab for 25(OH)D measurement. However, we will adhere to established procedures to maintain separation between laboratory staff and clinical staff that have patient contact. For quality assurance, Dr. Hollis will receive a report of

Downloaded From: http://jamanetwork.com/pdfaccess.ashx?url=/data/journals/jama/934885/ by a Harvard University User on 03/30/2017

measured 25(OH)D levels that are stripped of all identifying patient information (identifier and clinical information).

To ensure rapid intervention in the unexpected event of out-of-range ($\geq$1.0 mmol/mmol) calcium/creatinine ratios, urinary calcium and creatinine measurement will be conducted at each Clinical Center's Clinical Chemistry Laboratory. Again, we will adhere to established procedures to maintain separation between laboratory staff and clinical staff that have patient contact or are responsible for measuring primary or secondary endpoint outcomes. In the event of an out-of-range calcium/creatinine ratio, the Center Primary Investigator will be unblinded, the patient will be contacted for follow-up, and the DCC and NIH project officer will be notified.

Given the benign treatment intervention, the manufacturing of prenatal vitamins, placebo and Vitamin D supplement by the same drug company (Tishcon Corp), the distribution of study vitamins by the DCC, off-site assays of 25(OH)D levels, protocols to maintain separation between information available to laboratory staff, trial statisticians, the DCC, and site clinical staff and investigators, we anticipate that we will successfully maintain blinding of all investigators, clinical staff and participants to both treatment intervention and trial outcome until the end of the trial.

K. Data Safety and Monitoring of Each Subject

An external Data Safety and Monitoring Committee (DSMC), composed of a vitamin D metabolism expert, an early life asthma epidemiologist, and a statistician will be appointed. The committee will be convened prior to study initiation to review the protocol and to examine and comment upon "shell" reports that will be used to illustrate data accumulation as the study proceeds. The DSMC will determine the frequency and venue of study review meetings; we propose that reviews will occur as the enrollment process hits 50% and 100% of goal, and when the median follow-up times for enrolled patients hit 24, and 36 months. It may be possible to consolidate meetings if these events are close to coincident. Because the treatment is known to be benign, we propose that conference calls will be an adequate approach to DSMC meetings.

All study subjects will be monitored for hypervitaminosis D. Operationally, we will define caution by urinary calcium/creatinine ratio ≥ 1.0 mmol/mmol from the samples that are obtained monthly starting 1 month after randomization. Whenever any patient exceeds the caution limit, the study medication will be temporally discontinued and a specific case study will be initiated to examine the contribution of confounding factors (e.g. possibility of volume restriction, diet with excessive vitamin D intake, sunlight exposure, other over the counter pills, etc.). A serum vitamin D level will also be obtained. If a basis for an elevated urinary calcium/creatinine cannot be determined and/or if the serum vitamin D level is >120 ng/ml, the study medicine will be stopped and the woman will be followed in the intention-to-treat paradigm.. The appropriate IRB will be notified, and the NIH and FDA will be notified per their protocols. The DSMB will be convened via fax and teleconference to review any subject who exceeds these predefined hypervitaminosis D limits. After the detailed case review has been performed by the clinical center to ascertain the cause of the hypervitaminosis D in the subject and reviewed by the DSMB to make an informed decision about the study's continuation. If the elevated levels in the subject are attributed to the vitamin D supplement, the subject will be withdrawn from supplementation but will continue to be monitored.

L. Interim Analyses

For the primary aim of asthma and recurrent wheeze, we have developed an interim analysis plan to monitor this composite endpoint over the three years of subject follow-up. This sequential monitoring plan consists of two interim analyses (conducted at year 3 month 12 or when all first year study visit data are available and year 4 month 12/when all first and second year visit data are available), and a final analysis at the end of year 5, when the three-year follow-up has been completed on all subjects. For maximum flexibility in the analysis, we will

Downloaded From: http://jamanetwork.com/pdfaccess.ashx?url=/data/journals/jama/934885/ by a Harvard University User on 03/30/2017

use a Lan-Demets alpha spending function[72] with O'Brien-Fleming bounds. For our group sequential design, we plan to use a Lan-Demets spending function with three equally spaced looks (with respect to the amount of information collected) and O'Brien-Fleming type bounds. The software program LANDEM (http://www.biostat.wisc.edu/landemets/)[73] was used to calculate the stopping boundar, which were $|z|=3.7103$, 2.5114, and 1.993 at the first, second, and third looks, respectively. The hypothesis tests are conducted with two-sided alternatives (i.e. a two-sided p-value), The power reflects the proportion of replicates where the Wald test statistic from the hypothesis test of $\beta_1=0$ is less than the stopping boundary specified for the first, second, or third looks, where β_1 is the coefficient for Vitamin D supplementation status. The replication is considered a 'success' if the stopping boundary is exceeded at any of the analysis times. In the simulations, the incidence of asthma and/or recurrent wheeze at the first and second interim looks is proportionate to the overall incidence expected at the end of the study period (i.e. at the first look, the incidence is one-third of the specified rate; at the second look, it is two-thirds). We do not expect incidence rates of 40-50% to be achieved until the third year of follow-up; therefore, the simulations reflect the approximate amount of information anticipated at each interim analysis. Only the primary endpoint of asthma and/or recurrent wheeze will be monitored sequentially. If the boundaries are crossed at either the first or second year analyses, results will be immediately reported and the study will be stopped.

M. Adverse Events. There are expected Adverse Events that occur with pregnancy, and they include pre-eclampsia, perinatal infections, anemia, prolonged rupture of the membranes and preterm labor, abnormal labor, and meconium staining. For the fetus/infant, Adverse Events include preterm delivery with birthweight <1500g, NICU admissions, hypoglycemia, and fetal abnormalities. If a serious Adverse Event occurs, the IRB and NIH will be notified in a timely manner. The mother's blood work will be reviewed and if there are abnormal levels of vitamin D or of urinary Ca/Cr, the DSMC will be notified. The DSMC will have access to the vitamin D dose to which each subject has been randomized. In the event of intrauterine/fetal death or perinatal death, information regarding the complications of the pregnancy, the events that led up to the fetal or perinatal death, and whether fetal anomalies were present will be recorded. Maternal vitamin D/calcium parameters will be measured within 24 hours after delivery, and the DSMC, IRBs, NIH, and FDA will be notified. It should be reiterated that vitamin D 4,000 IU/day has not been linked with any of these adverse events.

VIII. HUMAN SUBJECTS RESEARCH

Protection of Human Subjects

Risks to the Subjects

Human Subjects Involvement and Characteristics: For this project, we will be conducting a clinical trial of "high dose" vitamin D supplementation vs. "usual dose" prenatal vitamins in pregnant women for the prevention of asthma and allergies in the women's offspring at 3 years of age. We propose to randomize 720 pregnant women during their 1st trimester with 360 women to the "high dose" arm and 360 women to the usual care arm, follow them through their pregnancy and delivery, and follow the women and their offspring until the 3rd birthday of the children in the expectation to evaluate at least 600 three year olds. A brief study description and a copy of the consent form will be given to pregnant women receiving their care at 3 Clinical Centers (Boston, St. Louis, and San Diego) at their first prenatal visit (between 8 and 16 weeks gestation). Women interested in the study will be invited to a separate visit for more detailed explanation and providing informed consent. There are several reasons for this separate visit: 1) allow the pregnant women to review the informed consent, and 2) to assure as much as possible the dedication of the women to participation in the study until the 3rd birthday of their child. Pregnant women will be selected on the basis of an age >18 years but <35 years, ability to speak English or Spanish, intention to participate in the trial for the full 4 years, no pre-

Downloaded From: http://jamanetwork.com/pdfaccess.ashx?url=/data/journals/jama/934885/ by a Harvard University User on 03/30/2017

existing type 1 or type 2 diabetes mellitus, hypertension, and parathyroid or uncontrolled thyroid disease. Written informed consent (in English or Spanish, according to individual preferences) will be obtained from each subject by study coordinators at each Clinical Center prior to voluntary participation. Participating women will be followed during the remainder of their pregnancy, and their newborns will be followed after delivery.

Sources of Research Material: Data from questionnaires, maternal (labor and delivery) and neonatal medical records will be used for research purposes only. Peripheral blood will be obtained from the mothers at a prenatal visit, 14 weeks after enrollment, and at delivery for measurement of vitamin D levels. Cord blood will be obtained from the research subjects to be used by the investigators for measurement of vitamin D levels and storage of white cell pellets for future genetic studies. Peripheral blood will be obtained from the children to be used by the investigators for measurement of vitamin D levels at 1 yr and 3yrs and total and allergen-specific IgE in the child at 3 yrs. These blood samples will be used by the investigators for research purposes only; the results will be kept strictly confidential. With parental permission, results of measurements of serum total and allergen-specific IgE at age 3 years will be sent to the child's primary care physician.

The major risk involved with vitamin D toxicity is hypercalcemia, which can be detected by measurement in serum or by calcium excretion in urine. Based on Dr. Hollis' extensive review of the literature and his experience with his current vitamin D supplementation in pregnancy trial, vitamin D supplementation of 4,400 IU/day throughout pregnancy is safe. Nevertheless, we will be monitoring urine calcium-to-creatinine ratios as a measure to calcium excretion in the urine each time the mothers come for their prenatal visits. This method of monitoring was chosen because it is reliable and does not require phlebotomy.

Another risk of participation in this study is the social-psychological risk resulting from inadvertent disclosure of medical history information. This potential risk is guarded against by storage of completed questionnaires in a locked filing system and by labeling other phenotypic/genotypic data using ID numbers only. In certain instances, the funding agency for this grant may require posting of data for sharing with outside researchers. In these instances, these researchers will be asked to sign a statement of confidentiality before being allowed access to the data. In addition, no personal identifiers will be included in datasets that will be shared with outside researchers.

The only potential risks of blood drawing are hematoma and infection at the skin site, minimal pain of venous puncture, uncommonly fainting reactions, and additionally discomfort and crying from the child.

Adequacy of Protection against Risks

Recruitment of Subjects and Informed Consent: Subjects will be recruited voluntarily from those meeting entry criteria. Written consent will be obtained from participating women before randomization, collection of questionnaire data, extraction of information from maternal (labor and delivery) and neonatal records, and samples of cord blood and peripheral blood. Subjects are under no obligation to participate. All signed consent forms are copied times three, one placed in the patient's research file and a second in a common consent form folder for the study and a copy given to the patient. The consenting process is an ongoing process and participants will be informed of important new developments that relate importantly to the safety of the study. In addition at each follow-up visit study procedures and requirements are reviewed.

Protection against Risks: To monitor for the rare potential for Vitamin D toxicity urine samples will be evaluated for Calcium/Creatinine at each clinical prenatal visit and for Vitamin D levels at the prenatal research visit 14 weeks post-randomization. Should levels of Calcium/Creatinine exceed 1.0 mmol/mmol or Vitamin D levels exceed 200 nmol, a specific case study will be initiated to examine the contribution of confounding factors (e.g. diet, sunlight exposure, other over the counter pills, etc.). The appropriate IRB will be notified, and the NIH and FDA will be notified per their protocols. The DSMC will be convened via fax and teleconference to review

Downloaded From: http://jamanetwork.com/pdfaccess.ashx?url=/data/journals/jama/934885/ by a Harvard University User on 03/30/2017

any subject who exceeds these predefined hypervitaminosis D limits. A detailed case review will be performed by the DSMC to ascertain the cause of the hypervitaminosis D in the subject and to make an informed decision about the study's continuation. If the elevated levels in the subject are attributed to the vitamin D supplement, the subject will be withdrawn from supplementation but will continue to be monitored. As noted above, steps will be taken to ensure that study information remains confidential. As we have mentioned in the background section, there have been no reported adverse effects on the fetus due to vitamin D supplementation. By monitoring the urinary calcium/creatinine ratio in the pregnant mothers and ensuring that the vitamin D levels do not exceed 200 nmol, the risks to the fetus will be minimized. The discomforts associated with blood drawing are minimal. Phlebotomy will be performed by licensed phlebotomists or research assistants that are adequately trained for blood drawing in infants. For maternal samples, we will minimize blood draws by piggybacking the collection at delivery with the blood draw for clinical purposes.

Potential Benefit to Individual Subjects and Society: For the participating mothers, a direct benefit will be close monitoring, and thus better adherence, of prenatal vitamin intake, which has already established benefits for fetal wellbeing. Additionally, even for those women in the placebo group, they will be receiving 400 IU of vitamin D per day in the prenatal vitamin that the study will be supplying for free. This 400 IU dose is twice that of the current AI recommendations of the IOM. The prenatal vitamins will be provided free of charge to all participants in the trial. The benefits of vitamin D on several outcomes related to bone health are well-documented[74]. In addition, there is emerging evidence that vitamin D may aid in combating infections, through its effects on innate immunity[75, 76]. This mechanism may also apply with infections during pregnancy[77]. Thus, mothers in the intervention arm will get the potential benefits of improved bone health and of a decrease in clinically significant infections.

For infants in the trial, a potential benefit is the close monitoring for the development of asthma and allergies. Additionally, we will offer 400 IU/day of vitamin D to all the infants who consume less than 1000 ml of formula per day, according to the recommendations of the American Academy of Pediatrics. This include all children who are breastfed, either partially or exclusively and all formula-fed infants until they consume at least 1000 ml of formula per day. These vitamin D supplements will be provided free of charge to the infants in the trial.

The potential benefit to society is significant if the trial shows a decrease in the prevalence of asthma, wheezing illnesses, and allergies at 3 years of age. Allergic diseases such as asthma are a major public health problem in the U.S. Since the risks involved in the protocol are small and the societal benefits are potentially large, the risk-benefit ratio is strongly on the side of benefit.

Importance of the Knowledge to Be Gained:

As previously stated, the main potential benefit of this research proposal is that it may lead to public health interventions that could lead to a decrease of up to 50% of the asthma prevalence worldwide, and may contribute to our understanding of the pathogenesis of allergic diseases such as asthma. While there are individual risks involved in this project, we will institute mechanisms to minimize these risks. Thus, because of the potentially great societal benefit, the individual risks are reasonable

Downloaded From: http://jamanetwork.com/pdfaccess.ashx?url=/data/journals/jama/934885/ by a Harvard University User on 03/30/2017

References

1. Yunginger JW, Reed CE, O'Connell EJ, Melton LJ, 3rd, O'Fallon WM, Silverstein MD. A community-based study of the epidemiology of asthma. Incidence rates, 1964-1983. Am Rev Respir Dis 1992;146:888-94.

2. King ME, Mannino DM, Holguin F. Risk factors for asthma incidence. A review of recent prospective evidence. Panminerva Med 2004;46:97-110.

3. Mannino DM, Homa DM, Akinbami LJ, Moorman JE, Gwynn C, Redd SC. Surveillance for asthma--United States, 1980-1999. MMWR Surveill Summ 2002;51:1-13.

4. CDC. Asthma prevalence, health care use, and mortality, 2002. . Hyattsville, MD: US Department of Health and Human Services, CDC, National Center for Health Statistics; 2004.

5. Smith DH, Malone DC, Lawson KA, Okamoto LJ, Battista C, Saunders WB. A national estimate of the economic costs of asthma. Am J Respir Crit Care Med 1997;156:787-93.

6. Adams PF, Hendershoot GE, Marano MA. Current Estimates from the National Health Interview Survey, 1996. Hyattsville, MD: National Center for Health Statistics 1999;Vital Health Stat Ser 10.

7. Holick MF. High prevalence of vitamin D inadequacy and implications for health. Mayo Clin Proc 2006;81:353-73.

8. Nesby-O'Dell S, Scanlon KS, Cogswell ME, et al. Hypovitaminosis D prevalence and determinants among African American and white women of reproductive age: third National Health and Nutrition Examination Survey, 1988-1994. Am J Clin Nutr 2002;76:187-92.

9. US Environmental Protection Agency. Report to Congress on indoor air quality, volume II: assessment and control of indoor air pollution, Report No. EPA 400-1-89-001C. Washington, DC: EPA; 1989.

10. Hollis BW, Wagner CL. Assessment of dietary vitamin D requirements during pregnancy and lactation. Am J Clin Nutr 2004;79:717-26.

11. Hollis BW, Wagner CL. Vitamin D deficiency during pregnancy: an ongoing epidemic. Am J Clin Nutr 2006;84:273.

12. Hollis BW, Wagner CL. Nutritional vitamin D status during pregnancy: reasons for concern. Cmaj 2006;174:1287-90.

13. Sanchez PA, Idrisa A, Bobzom DN, et al. Calcium and vitamin D status of pregnant teenagers in Maiduguri, Nigeria. J Natl Med Assoc 1997;89:805-11.

14. Taylor SN, Wagner CL, Hulsey TC, Ebeling M, Hollis BW. Bone mineralization correlates with vitamin D status in the vitamin D insufficient range in newborns. In: ASBMR Scientific Meeting - Contemporary Diagnosis and Treatment of Vitamin D Related Disorders; December 4-5, 2006; Arlington, VA; December 4-5, 2006.

15. Lee JM, Smith JR, Philipp BL, Chen TC, Mathieu J, Holick MF. Vitamin d deficiency in a healthy group of mothers and newborn infants. Clin Pediatr (Phila) 2007;46:42-4.

16. Bassir M, Laborie S, Lapillonne A, Claris O, Chappuis MC, Salle BL. Vitamin D deficiency in Iranian mothers and their neonates: a pilot study. Acta Paediatr 2001;90:577-9.

17. Sachan A, Gupta R, Das V, Agarwal A, Awasthi PK, Bhatia V. High prevalence of vitamin D deficiency among pregnant women and their newborns in northern India. Am J Clin Nutr 2005;81:1060-4.

18. Judkins A, Eagleton C. Vitamin D deficiency in pregnant New Zealand women. N Z Med J 2006;119:U2144.

19. van der Meer IM, Karamali NS, Boeke AJ, et al. High prevalence of vitamin D deficiency in pregnant non-Western women in The Hague, Netherlands. Am J Clin Nutr 2006;84:350-3; quiz 468-9.

Downloaded From: http://jamanetwork.com/pdfaccess.ashx?url=/data/journals/jama/934885/ by a Harvard University User on 03/30/2017

20. Nguyen M, Guillozo H, Garabedian M, Balsan S. Lung as a possible additional target organ for vitamin D during fetal life in the rat. Biol Neonate 1987;52:232-40.
21. Nguyen TM, Guillozo H, Marin L, Tordet C, Koite S, Garabedian M. Evidence for a vitamin D paracrine system regulating maturation of developing rat lung epithelium. Am J Physiol 1996;271:L392-9.
22. Marin L, Dufour ME, Nguyen TM, Tordet C, Garabedian M. Maturational changes induced by 1 alpha,25-dihydroxyvitamin D3 in type II cells from fetal rat lung explants. Am J Physiol 1993;265:L45-52.
23. Marin L, Dufour ME, Tordet C, Nguyen M. 1,25(OH)2D3 stimulates phospholipid biosynthesis and surfactant release in fetal rat lung explants. Biol Neonate 1990;57:257-60.
24. Nguyen M, Trubert CL, Rizk-Rabin M, et al. 1,25-Dihydroxyvitamin D3 and fetal lung maturation: immunogold detection of VDR expression in pneumocytes type II cells and effect on fructose 1,6 bisphosphatase. J Steroid Biochem Mol Biol 2004;89-90:93-7.
25. Glasgow JF, Thomas PS. Rachitic respiratory distress in small preterm infants. Arch Dis Child 1977;52:268-73.
26. Rehan VK, Torday JS, Peleg S, et al. 1Alpha,25-dihydroxy-3-epi-vitamin D3, a natural metabolite of 1alpha,25-dihydroxy vitamin D3: production and biological activity studies in pulmonary alveolar type II cells. Mol Genet Metab 2002;76:46-56.
27. Phokela SS, Peleg S, Moya FR, Alcorn JL. Regulation of human pulmonary surfactant protein gene expression by 1alpha,25-dihydroxyvitamin D3. Am J Physiol Lung Cell Mol Physiol 2005;289:L617-26.
28. Gaultier C, Harf A, Balmain N, Cuisinier-Gleizes P, Mathieu H. Lung mechanics in rachitic rats. Am Rev Respir Dis 1984;130:1108-10.
29. Brun P, Dupret JM, Perret C, Thomasset M, Mathieu H. Vitamin D-dependent calcium-binding proteins (CaBPs) in human fetuses: comparative distribution of 9K CaBP mRNA and 28K CaBP during development. Pediatr Res 1987;21:362-7.
30. Lunghi B, Meacci E, Stio M, et al. 1,25-dihydroxyvitamin D3 inhibits proliferation of IMR-90 human fibroblasts and stimulates pyruvate kinase activity in confluent-phase cells. Mol Cell Endocrinol 1995;115:141-8.
31. Stio M, Celli A, Lunghi B, Raugei G, Modesti A, Treves C. Vitamin D receptor in IMR-90 human fibroblasts and antiproliferative effect of 1,25-dihydroxyvitamin D3. Biochem Mol Biol Int 1997;43:1173-81.
32. Stio M, Lunghi B, Celli A, Nassi P, Treves C. Effect of 1,25-dihydroxyvitamin D3 on proliferation in senescent IMR-90 human fibroblasts. Mech Ageing Dev 1996;91:23-36.
33. Camargo J, C.A., Rifas-Shiman SL, Litonjua AA, et al. Maternal intake of vitamin D during pregnancy and risk of recurrent wheeze in children at age 3 years. Am J Clin Nutr 2007;85:788-95.
34. Devereux G, Litonjua AA, Turner S, et al. Maternal vitamin D intake during pregnancy and early childhood wheezing. Am J Clin Nutr 2007;85:853-9.
35. Lehtonen-Veromaa M, Mottonen T, Irjala K, et al. Vitamin D intake is low and hypovitaminosis D common in healthy 9- to 15-year-old Finnish girls. Eur J Clin Nutr 1999;53:746-51.
36. Vieth R, Cole DE, Hawker GA, Trang HM, Rubin LA. Wintertime vitamin D insufficiency is common in young Canadian women, and their vitamin D intake does not prevent it. Eur J Clin Nutr 2001;55:1091-7.

Downloaded From: http://jamanetwork.com/pdfaccess.ashx?url=/data/journals/jama/934885/ by a Harvard University User on 03/30/2017

37. Datta S, Alfaham M, Davies DP, et al. Vitamin D deficiency in pregnant women from a non-European ethnic minority population--an interventional study. Bjog 2002;109:905-8.
38. Talwar SA, Aloia JF, Pollack S, Yeh JK. Dose response to vitamin D supplementation among postmenopausal African American women. Am J Clin Nutr 2007;86:1657-62.
39. Hollis BW. Circulating 25-hydroxyvitamin D levels indicative of vitamin D sufficiency: implications for establishing a new effective dietary intake recommendation for vitamin D. J Nutr 2005;135:317-22.
40. Brooke OG, Brown IR, Bone CD, et al. Vitamin D supplements in pregnant Asian women: effects on calcium status and fetal growth. Br Med J 1980;280:751-4.
41. Cockburn F, Belton NR, Purvis RJ, et al. Maternal vitamin D intake and mineral metabolism in mothers and their newborn infants. Br Med J 1980;281:11-4.
42. Delvin EE, Salle BL, Glorieux FH, Adeleine P, David LS. Vitamin D supplementation during pregnancy: effect on neonatal calcium homeostasis. J Pediatr 1986;109:328-34.
43. Mallet E, Gugi B, Brunelle P, Henocq A, Basuyau JP, Lemeur H. Vitamin D supplementation in pregnancy: a controlled trial of two methods. Obstet Gynecol 1986;68:300-4.
44. Ala-Houhala M, Koskinen T, Terho A, Koivula T, Visakorpi J. Maternal compared with infant vitamin D supplementation. Arch Dis Child 1986;61:1159-63.
45. Hollis BW, Wagner CL. Vitamin D requirements during lactation: high-dose maternal supplementation as therapy to prevent hypovitaminosis D for both the mother and the nursing infant. Am J Clin Nutr 2004;80:1752S-8S.
46. Wagner CL, Hulsey TC, Fanning D, Ebeling M, Hollis BW. High-dose vitamin D3 supplementation in a cohort of breastfeeding mothers and their infants: a 6-month follow-up pilot study. Breastfeeding Medicine 2006;1:57-68.
47. Vieth R. Vitamin D supplementation, 25-hydroxyvitamin D concentrations, and safety. Am J Clin Nutr 1999;69:842-56.
48. Heaney RP, Davies KM, Chen TC, Holick MF, Barger-Lux MJ. Human serum 25-hydroxycholecalciferol response to extended oral dosing with cholecalciferol. Am J Clin Nutr 2003;77:204-10.
49. Food and Nutrition Board. Institute of Medicine. Dietary reference intakes for calcium, phosphorus, magnesium, vitamin D, and fluoride. Washington, DC: National Academy Press; 1997.
50. Hathcock JN, Shao A, Vieth R, Heaney R. Risk assessment for vitamin D. Am J Clin Nutr 2007;85:6-18.
51. Wagner CL, Greer FR. Prevention of rickets and vitamin d deficiency in infants, children, and adolescents. Pediatrics 2008;122:1142-52.
52. Wang X, Chen C, Wang L, Chen D, Guang W, French J. Conception, early pregnancy loss, and time to clinical pregnancy: a population-based prospective study. Fertil Steril 2003;79:577-84.
53. Hollis BW, Kamerud JQ, Selvaag SR, Lorenz JD, Napoli JL. Determination of vitamin D status by radioimmunoassay with an 125I-labeled tracer. Clin Chem 1993;39:529-33.
54. Hollis BW, Horst RL. The assessment of circulating 25(OH)D and 1,25(OH)(2)D: Where we are and where we are going. J Steroid Biochem Mol Biol 2006.
55. Broadus AE, Horst RL, Lang R, Littledike ET, Rasmussen H. The importance of circulating 1,25-dihydroxyvitamin D in the pathogenesis of hypercalciuria and renal-stone formation in primary hyperparathyroidism. N Engl J Med 1980;302:421-6.

Downloaded From: http://jamanetwork.com/pdfaccess.ashx?url=/data/journals/jama/934885/ by a Harvard University User on 03/30/2017

56. Horst RL, Littledike ET, Riley JL, Napoli JL. Quantitation of vitamin D and its metabolites and their plasma concentrations in five species of animals. Anal Biochem 1981;116:189-203.
57. Reinhardt TA, Horst RL, Orf JW, Hollis BW. A microassay for 1,25-dihydroxyvitamin D not requiring high performance liquid chromatography: application to clinical studies. J Clin Endocrinol Metab 1984;58:91-8.
58. Stewart AF, Horst R, Deftos LJ, Cadman EC, Lang R, Broadus AE. Biochemical evaluation of patients with cancer-associated hypercalcemia: evidence for humoral and nonhumoral groups. N Engl J Med 1980;303:1377-83.
59. Hollis BW. Editorial: The determination of circulating 25-hydroxyvitamin D: no easy task. J Clin Endocrinol Metab 2004;89:3149-51.
60. Hollis BW, Wagner CL. Normal serum vitamin D levels. N Engl J Med 2005;352:515-6.
61. Goff JP, Horst RL. Assessing adequacy of cholecalciferol supplementation in chicks using plasma cholecalciferol metabolite concentrations as an indicator. J Nutr 1995;125:1351-7.
62. Willett WC, Sampson L, Stampfer MJ, et al. Reproducibility and Validity of a Semiquantitative Food Frequency Questionnaire. Am J Epidemiol 1985;122.
63. Fawzi WW, Rifas-Shiman SL, Rich-Edwards JW, Willett WC, Gillman MW. Calibration of a semi-quantitative food frequency questionnaire in early pregnancy. Ann Epidemiol 2004;14:754-62.
64. US Department of Agriculture ARS. USDA National Nutrient Database for Standard Reference, Release 14, SR14; 2001. In: 2001.
65. Han J, Colditz GA, Hunter DJ. Risk factors for skin cancers: a nested case-control study within the Nurses' Health Study. Int J Epidemiol 2006.
66. Johansson SG, Yman L. In vitro assays for immunoglobulin E. Methodology, indications, and interpretation. Clin Rev Allergy 1988;6:93-139.
67. Yman L, Bloomberg F, H. S. Characterization of dog and cat allergens. Direct specific detection of electrophoretically separated allergens by means of IgE antibodies and enzyme labelled anti-IgE. In: A O, ed. Advances in Allergology and Immunology. Oxford: Pergamon Press; 1980:499-504.
68. Riekert KA, Rand CS. Electronic monitoring of medication adherence: When is high-tech best? J Clin Psychol Med Settings 2002;9:25-34.
69. Sorkness CA, Lemanske RF, Jr., Mauger DT, et al. Long-term comparison of 3 controller regimens for mild-moderate persistent childhood asthma: The Pediatric Asthma Controller Trial. J Allergy Clin Immunol 2007;119:64-72.
70. Szefler SJ, Phillips BR, Martinez FD, et al. Characterization of within-subject responses to fluticasone and montelukast in childhood asthma. J Allergy Clin Immunol 2005;115:233-42.
71. Rand C, Bilderback A, Schiller K, Edelman JM, Hustad CM, Zeiger RS, for The MIAMI Study Research Group. Adherence with montelukast or fluticasone in a long-term clinical trial: results from the Mild Asthma Montelukast Versus Inhaled Corticosteroid (MIAMI) study. . J Allergy Clin Immunol (in Press) 2007.
72. Lan KKG, DeMets DL. Discrete sequential boundaries for clinical trials. Biometrika 1983;70:659-63.
73. Reboussin DM, DeMets DL, Kim KM, Lan KK. Computations for group sequential boundaries using the Lan-DeMets spending function method. Control Clin Trials 2000;21:190-207.

Downloaded From: http://jamanetwork.com/pdfaccess.ashx?url=/data/journals/jama/934885/ by a Harvard University User on 03/30/2017

74. Cranney A, Weiler HA, O'Donnell S, Puil L. Summary of evidence-based review on vitamin D efficacy and safety in relation to bone health. Am J Clin Nutr 2008;88:513S-9S.
75. Liu PT, Stenger S, Li H, et al. Toll-like receptor triggering of a vitamin D-mediated human antimicrobial response. Science 2006;311:1770-3.
76. Martineau AR, Wilkinson RJ, Wilkinson KA, et al. A single dose of vitamin D enhances immunity to mycobacteria. Am J Respir Crit Care Med 2007;176:208-13.
77. Liu N, Kaplan AT, Low J, et al. Vitamin D Induces Innate Antibacterial Responses in Human Trophoblasts via an Intracrine Pathway. Biol Reprod 2008.

Downloaded From: http://jamanetwork.com/pdfaccess.ashx?url=/data/journals/jama/934885/ by a Harvard University User on 03/30/2017

Current Protocol

Downloaded From: http://jamanetwork.com/pdfaccess.ashx?url=/data/journals/jama/934885/ by a Harvard University User on 03/30/2017

DETAILED PROTOCOL
"Randomized Trial: Maternal Vitamin D Supplementation to Prevent Childhood Asthma"
Acronym: VDAART (Vitamin D Antenatal Asthma Reduction Trial

I. Background and Significance

Asthma is one of the leading causes of morbidity in children with 90% of all cases diagnosed by age 6[1, 2]. It remains the most common chronic disease of childhood in the world[3, 4], and incurs significant healthcare costs[5, 6]. The burden of the disease in both the developed and the developing world is significant and increasing rapidly with over 300 million people affected worldwide. Thus, preventing the development of this disorder would be of great public health importance.

Vitamin D deficiency has been documented in many populations worldwide[7, 8], and has been reported in healthy children, young adults (especially African Americans), and middle-aged and elderly adults[7, 8]. Vitamin D deficiency has occurred despite fortification of foods in some countries and despite intake of multivitamins containing vitamin D. This suggests that as countries adopt a western lifestyle, there is shift from outdoor activities to more time spent indoors. For example, it is estimated that in the US alone, Americans spend an average of 93% of their time indoors[9]. **Relevant to this study, pregnant and lactating mothers and their neonates are at especially high risk for vitamin D deficiency**[10-13]. Preliminary data from ongoing clinical trials show that many infants whose mothers are not being supplemented with sufficient doses of vitamin D have circulating 25(OH)D levels in the "insufficient" range (defined as levels between 25 - 62.5 nmol/L)[14]. Furthermore, it has recently been documented that 50% of mothers and 65% of their newborn infants from an inner city hospital were vitamin D deficient, with levels <30 nmol/L[15], despite the fact that most mothers were taking their prenatal vitamins. This analysis also showed a strong positive correlation between maternal and newborn plasma levels, providing further evidence that infant vitamin D status is dependent on maternal vitamin D status. These results are similar to other recent reports that show a high prevalence of vitamin D deficiency in pregnant women and their newborns, even in some sun replete areas of the world. The prevalence of deficiency (defined as 25(OH)D levels < 25 nmol/L among pregnant women in these studies were: 80% in Iran[16], 42% in Northern India[17], 61% in New Zealand[18], and 60-84% among non-Western women in the Netherlands[19].

The importance of vitamin D deficiency in pregnancy in relation to asthma development is that it has many recognized effects in on the developing lung and immune system. Nguyen and coworkers, through a series of studies in fetal rat lung, have identified the type II alveolar cells as a target for $1,25(OH)_2D_3$ action. Firstly, they identified specific binding sites (vitamin D receptors) for $1,25(OH)_2D_3$ that are distinguishable at a time corresponding to the start of type II pneumocyte differentiation and onset of surfactant secretion[20]. Next, with the use of monoclonal antibodies, they localized these vitamin D receptors to type II pneumocytes[21].Thirdly, in explanted fetal rat lung tissue, they showed that $1,25(OH)_2D_3$ accelerated the decrease in type II cells glycogen content and increased surfactant synthesis and surfactant secretion[22, 23]. Vitamin D was about half as potent as an equivalent dose of dexamethasone in stimulating surfactant synthesis and secretion. They also showed that a paracrine system for vitamin D is present in rat fetal lung and function during the last 3 days of pregnancy[21], and that some of the effects of vitamin D may be mediated by fructose 1,6 biphosphatase, whose gene contains a vitamin D response element in the promoter region[24]. **These recent observations strongly suggest that vitamin D plays a role in lung maturation late in pregnancy, and support earlier observations such as the association of respiratory distress in vitamin D deficient preterm infants ("rachitic respiratory distress")[25]. In humans, the effect of vitamin D on surfactant production has been confirmed[26], although the mechanisms appear to be more complex than in the rat[27].**

Downloaded From: http://jamanetwork.com/pdfaccess.ashx?url=/data/journals/jama/934885/ by a Harvard University User on 03/30/2017

Aside from effects on type II pneumocytes and surfactant production, vitamin D also appears to have effects on lung growth and development. Gaultier et al[28] studied lung mechanics in 50-day old rats born to mothers deprived of dietary vitamin D and reported significantly decreased lung compliance compared with rats born to mothers whose diet was supplemented with vitamin D, suggesting that disturbances in lung growth occurred in the vitamin D deficient rats. In humans, vitamin D also has been shown to play a role in the developing lung. Early studies used the presence of calbindin, a vitamin D dependent calcium binding protein, as a molecular marker of $1,25(OH)_2D_3$ action in tissues. Among many other tissues, Brun et al[29] reported high levels of calbindin in human fetal lung tissue at 14-32 weeks of gestation, suggesting that vitamin D plays a role in fetal lung development, as early as 14 weeks. Lunghi and colleagues[30] obtained normal human fetal (16 weeks gestation) lung fibroblasts and reported that in the presence of vitamin D, pyruvate kinase activity and lactate production of the cells increased. Other findings included a decrease in cell number and DNA synthesis in the vitamin D exposed cells compared with control cells. Subsequently, they showed that the vitamin D receptor was present in these human fetal fibroblasts[31] and confirmed their original findings in fetal lung fibroblasts in a subsequent study on senescent human lung fibroblasts[32]. **Thus, these animal and human tissue studies show that later in pregnancy, vitamin D stimulates lung maturation and surfactant production and secretion. Additionally, the data shows that at various points in fetal development, as early as 14 weeks of gestation, vitamin D has a regulatory role in fetal lung growth and development.**

We have recently shown in two independent birth cohorts that higher maternal intakes during pregnancy of vitamin D from both foods and supplements were associated with an almost 60% reduction of asthma and recurrent wheezing in 3-5 yr old children[33, 34].

There is also evidence that vitamin D affects immune system development and function. The fetal immune system begins to develop early in gestation[35]. In humans, pre-B and pre-T cells arise by 8 weeks gestation, predominantly in the fetal liver. The thymus begins to develop at 6 weeks gestation, the spleen and lymph nodes later, and the gut-associated lymphoid tissue later still. These organs, however, continue to grow and develop for many years after birth. Vitamin D receptors (VDR)[36, 37] and vitamin D metabolic enzymes[7, 38] have been identified in many other tissues aside from bone and the intestine, suggesting involvement in the metabolism and function of many cell types. Specifically, VDR is expressed in cells of the immune system, such as T[39], activated B cells[40], and dendritic cells[41]. Evidence exists that vitamin D may induce a shift in the balance between Th1 and Th2-type cytokines toward Th2 dominance[42, 43]. Reduced secretion of Th1 cytokines IL-2 and IFN-γ[43-45] and an increase in the Th2 cytokine IL4[46, 47] have been observed in several experiments after treatment with $1,24(OH)_2D$. **These studies, however, did not investigate the developing fetal immune system.** Evans, et al[48] showed that human decidual cells are able to synthesize active 1,25(OH)(2)D(3), particularly in early gestation (first trimester), and this may act in an autocrine/paracrine fashion to regulate both acquired and innate immune responses at the fetal-maternal interface. More directly, Pichler et al.[49, 50] showed that in CD4+ as well as CD8+ human cord blood cells, vitamin D inhibits not only IL-12-generated IFN-γ production, but also suppresses IL-4, and IL-4-induced expression of IL-13, thus, **confirming that in the neonate, vitamin D regulates immune responses and contributes to the development of the immune system**. The differences between the studies on the Th1-Th2 dominance lie in part, in the timing of vitamin D exposure of the cells to vitamin D (i.e. prenatal vs. postnatal). Thus, the response of “naïve” T-cells to vitamin D exposure may differ from that of mature cells when exposed to vitamin D[50]. In children with established asthma, we have recently shown that vitamin D levels were inversely associated with total IgE levels and with eosinophil count[51]. Others have also shown a relationship between vitamin D levels and total serum IgE in adults[52]. In addition, small trials have shown an effect of vitamin D on eczema. In one trial exposure to

Downloaded From: http://jamanetwork.com/pdfaccess.ashx?url=/data/journals/jama/934885/ by a Harvard University User on 03/30/2017

tropical sunlight (presumably increasing vitamin D levels)[53] and in another vitamin D supplementation have been shown to lead to improvement in atopic eczema[54].

Aside from the effects of vitamin D on Th1 and Th2 responses, there is a growing recognition that it promotes the induction of T-reg cells[55-57] . The characteristic features of Treg cells include the expression of potentially inhibitory cytokines (IL10 and TGFβ), and the ability to potently inhibit antigen-specific T cell activation[58, 59]. These effects of vitamin D on the immune system underlie the regulator role of vitamin D and are thought to be orchestrated by an array of intracellular signaling pathways in lymphocytes and antigen-presenting cells of which Il10 and TGFβ are the most important (reviewed in Griffin et al.[60]). These pathways lead to either over- or under-expression of genes. For example, vitamin D has been shown to modify levels or function of NF-κB proteins such as Rel-B and c-Rel.[61-63] Additionally, there is recent evidence that vitamin D regulates many genes that have been associated with asthma[64]. Finally, vitamin D-dependent binding of vitamin D receptor (VDR) complexes directly to promoter regions of activation-induced genes in T lymphocytes has been shown[65].

II. Hypothesis and Specific Aims

Our hypothesis is that supplementing pregnant women with sufficient vitamin D will decrease the incidence of allergies, asthma and wheezing illnesses in children. The initial basis for this hypothesis is our finding in two separate birth cohorts of the protective effect of vitamin D intake on wheezing illnesses in 3-5 yr old children. Our birth cohort studies are consistent with data from studies that have investigated animal fetal models and human fetal lung tissue that show that (1) vitamin D receptors are present in the fetal lung, (2) vitamin D enhances lung maturation in the last stages of pregnancy, (3) vitamin D controls growth and development of the fetal lung, and (4) profile of human cord blood cell response to antigens is affected by vitamin D. Finally, there is a large amount of literature that shows that there is a high prevalence of vitamin D deficiency among women of child-bearing age and in pregnant women, such that infants are being born deficient in vitamin D. Furthermore, in both our birth cohorts, vitamin D intake in the first year of life and in childhood did not modify the association of maternal vitamin D intakes with any of the wheeze outcomes. Additionally, there is experimental data that vitamin D affects the development of the neonatal immune system. Taken together, these data strongly suggest that the right time to intervene is during pregnancy. Therefore, we propose a two-arm, randomized, double-blind, placebo-controlled, multi-center clinical trial to determine whether vitamin D supplementation in the pregnant mother will prevent asthma and allergy at age 3. We will utilize asthma/recurrent wheezing as our primary end point and allergy and lower respiratory infection as secondary end points. We will recruit 870 pregnant women during the first trimester of pregnancy and randomize them to one of two treatment arms of a 5-year clinical trial: the addition of 4000 IU daily of vitamin D to usual pregnancy vitamin D supplementation (daily prenatal vitamins with 400 IU vitamin D; total dose in the intervention arm of 4,400 IU/day) in the treatment group compared with placebo plus usual vitamin D supplementation (daily prenatal vitamins with 400 IU vitamin D) in the control group. Because we expect an 8% rate of miscarriages and stillbirths, and about 18% withdrawals or loss to follow-up, we expect that we will have 660 children (330 in each arm) for analysis of the primary outcome at 3 years of age.

Our primary aims are:

1. To determine whether vitamin D supplementation in the pregnant mother is associated with reduced incidence of asthma in the child during the first 3 years of life. Our primary outcomes will be doctor's diagnosis of asthma and/or recurrent wheeze in the child at age 3 years.

Downloaded From: http://jamanetwork.com/pdfaccess.ashx?url=/data/journals/jama/934885/ by a Harvard University User on 03/30/2017

2. To determine whether a vitamin D dose of 4,400 IU per day vs. the standard 400 IU/day in pregnancy is sufficient to maintain mothers' vitamin D levels in the range of ≥ 75 nmol/L 25(OH)D.

In addition to our primary aims, we have the following secondary aims of the trial:

3. To determine whether vitamin D supplementation in the pregnant mother is associated with reduced secondary outcomes in the neonate of cord blood total IgE, and reduced secondary outcomes in the 3-yr old child of (a) allergic sensitization (total and specific IgE), (b) eosinophil count, (c) doctor's diagnosis of eczema and (d) lower respiratory tract infections.

4. To determine whether vitamin D supplementation in the pregnant mother is associated with improved vitamin D status in their offspring through measurement of 25(OH)D levels in cord blood (at delivery), and children's blood (at 1 and 3 yrs of age).

5. To determine whether sufficient vitamin D supplementation in the pregnant mother is associated with reduced incidence of preterm birth (birth <37 weeks gestation), preeclampsia, gestational hypertension, and/or Hemolytic anemia, Elevated Liver enzymes, Low Platelet count (HELLP syndrome) (PB/PE) compared to a usual care control group in VDAART.

III. Vitamin D supplementation: safety considerations.

The current adequate intake (AI) for pregnant and lactating women is 200 IU/day (although most prenatal vitamins contain 400 IU vitamin D). However, multiple studies have shown that these current recommendations result in a high degree of vitamin D deficiency in different adult populations[66-68]. One recent study supplemented 160 minority women in the UK with 800-1600 IU/day vitamin D throughout their pregnancies[68]. The investigators found a mean increase in circulating 25(OH)D concentrations from 14.5 ± 2.2 nmol/L at the beginning of pregnancy to 28 ± 16 nmol/L at term after vitamin D supplementation. Therefore, mothers who were deficient at the beginning of their pregnancy were still deficient at the end of their pregnancy after supplementation, despite supplementation with a higher than recommended dose of vitamin D. A more recent study has supported these findings. Talwar et al [69] studied 208 postmenopausal African American women over 3 years. Their results showed that a dose of 2,000 IU/day raised 25(OH)D levels to 75 nmol/L in only 60% of the participants. This finding is relevant because pregnant women have greater vitamin D requirements (due to the growing fetus) than postmenopausal women. The results of these studies point out that the AI for vitamin D during pregnancy is grossly inadequate. **Given that a single minimal erythemic dose (MED) exposure (for a Caucasian individual, this is approximated by ~10-12 minutes of peak summer sun exposure) will release ~10,000 – 20,000 IU vitamin D into the circulation within 24 hours[70], the dose of vitamin D supplementation required to maintain adequate vitamin D status in adults is likely to be orders of magnitude greater than the current AI.**

The safety of giving "large" (relative to current AI, but not to normal responses to sun exposure) doses of vitamin D and the potential risk of vitamin D toxicity are valid concerns. The exact amount of vitamin D required to induce toxicity in humans is unknown. The safe Tolerable Upper Intake Level (UL) for vitamin D was set at 2,000 IU/day by the Food and Nutrition Board (FNB)[71] in 1997. However, this recommendation was not based on current evidence, and is viewed by many as being too restrictive. Vieth[72] estimates that the physiologic limit for daily vitamin D intake is 10,000 – 20,000 IU/day, and toxicity has never occurred when physiologic amounts are ingested. The data on safety of vitamin D supplementation in physiologic doses

Downloaded From: http://jamanetwork.com/pdfaccess.ashx?url=/data/journals/jama/934885/ by a Harvard University User on 03/30/2017

and animal models of toxicity have been extensively reviewed by Dr. Hollis, who is a co-investigator on this proposal[10]. In this review, several studies of high-dose vitamin D supplementation (ranging from 400 IU/day to 10,000 IU/day) conducted in healthy adults and pregnant and lactating women were noted, and these studies are summarized in Table 1. It is noteworthy that none of these studies have reported adverse outcomes due to supplementation. Supporting the notion that high-dose supplementation is safe, Hathcock and co-workers[73] reviewed new evidence in addition to the evidence reviewed by Dr. Hollis in 2004, and applying the risk assessment methodology used by the FNB, concluded that the absence of toxicity in trials conducted on healthy adults supported the selection of 10,000 IU/day as the new daily UL for vitamin D. We should note here that we are proposing a dose that is less than half of this. Furthermore, as noted above, Dr. Hollis' group is conducting a Phase 3 trial of vitamin D supplementation of up to 4,400 IU/day during pregnancy (4,000 IU/day + prenatal vitamins) (http://clinicaltrials.gov; RO1 HD 043921). Dr Wagner (Dr. Hollis' co-investigator) has generously supplied us the October, 2008 Data Safety Monitoring Committee (DSMC) report for their trial. **To date 347 women have completed the study and have delivered their babies and no episodes of vitamin D toxicity have occurred in the women, nor have adverse reactions been found in the fetuses or infants. The rates of spontaneous abortions, or other pregnancy related complications such as prematurity, have not increased in the vitamin D supplemented arms. Indeed in these subjects followed for 3123 person months of follow up, a total of 92 "adverse events" have been reported to the trial's DSMC. These events included diabetes (n=18), preeclampsia (n=5), gestational diabetes (n=17), hypertension (n=14), preterm labor (n=29), premature rupture of membranes (n=1), deep vein thrombosis (n=2), maternal infection (n=6), and placental abnormality (n=1). In detailed reviews, none of these pregnancy complications were attributed to vitamin D supplementation. Additionally, no neonatal physical abnormalities were detected on examination (C. Wagner, personal communication).** Therefore, the administration of 4,400 IU/day of vitamin D to pregnant women is safe. Dr. Hollis holds an IND with the FDA for safety of vitamin D supplementation in pregnancy (IND 66,346). He has shown that this dose is appropriate to bring levels in all of the women, regardless of race/ethnicity, to sufficient levels. Furthermore, the 4,400 IU/day dose eliminated all seasonal variation in 25(OH)D levels, which lower doses did not. His trials and data from others show that this dose is safe and not associated with any adverse effects in the mother or in the fetus/infant. This protocol will be covered under Dr. Hollis' IND.

Dr. Hollis' trial has also generated more preliminary data regarding the response to the various doses of vitamin D. Figure 1 shows the response to varying doses of vitamin D in pregnant women from 3 ethnic groups: Caucasian (n=38 in Control group; n=37 in 2,400 IU, and n=39 in 4,400 IU), African American (n=24 in Control; n=35 in 2,400 IU; and n=26 in 4,400 IU), and Hispanic (n=45 in Control; n=48 in 2,000 IU; and n=44 in 4,000 IU). Baseline 25(OH)D levels varied by ethnic group, with African Americans having the lowest levels and Caucasians having the highest baseline levels. The 2400 IU/day dose raised the circulating 25(OH)D levels to a mean of about 95 nmol/L, and only approximately 60% achieved levels of >75 nmol/L. The 4400 IU/day dose most rapidly and consistently increased 25(OH)D levels in the participants from all ethnic groups, with a mean of about 112 nmol/L, and practically all participants achieved levels of at least 75 nmol/L. Additionally, the 4400 IU/day dose eliminated all seasonal variation in circulating 25(OH)D levels. There were no instances of adverse events from the 4000 IU/day dose, either in the mother or the infant, even in those mothers whose beginning 25(OH)D levels were > 75 nmol/L. **Thus, 4400 IU/day of vitamin D was the dose that achieved sufficient supplementation in the pregnant women. The preliminary results and experience from Dr. Hollis' clinical trials have provided valuable information for our current proposal, regarding the dosing and safety of vitamin D supplementation in pregnancy.**

Downloaded From: http://jamanetwork.com/pdfaccess.ashx?url=/data/journals/jama/934885/ by a Harvard University User on 03/30/2017

TABLE 1. Summary of published high-dose vitamin D supplementation studies in healthy adults and pregnant and lactating women*. Adapted from Hollis and Wagner[10]

Reference†	Subject type	No. of subjects	Vitamin D dose	Therapy duration	Initial 25(OH)D	Endpoint 25(OH)D	Actual change in 25(OH)D
			IU/d	mo	nmol/L	nmol/L	nmol/L
**Supplementation in pregnancy**							
Brooke et al, 1980[74]‡	Pregnant Asians	67 Control<br>59 Supplemented	0<br>1,000	3	20<br>20	16<br>168	-3.7<br>+148
Cockburn et al, 1980[75]	Pregnant women	82 Control<br>82 Supplemented	0<br>400	4	32<br>39	32<br>43	0<br>+3.7
Delvin et al, 1986[76]	Pregnant women	13 Supplemented	1,000	3	32±10[5]	65±17.5	+32
Mallet et al, 1986[77]	Pregnant women	29 Supplemented	1,000	3	9.5±5	25±15.7	+15.7
Datta et al, 2002[68]	Pregnant minorities	80 Supplemented	800-1,600	>6	14.5±2.2	28±15.7	+13.5
**Supplementation in Lactating women**							
Ala-Houhala, 1985[78]	Lactating women	16 Supplemented<br>17 Supplemented	1,000<br>2,000	4.5	11<br>32	65<br>90	+40<br>+57
Hollis and Wagner, 2004[79]	Lactating women	9 Supplemented<br>9 Supplemented	2,000<br>4,000	3	69±24<br>81±17	90±17.5<br>111±28.5	+21<br>+30
Wagner et al, 2006[80]	Lactating women and infants	10 Supplemented<br>9 Supplemented<br>10 Supplemented	400<br>6,400<br>300	6	80<br>85	96<br>147	+10.5<br>+62
**Supplementation in non-Pregnant Healthy subjects**							
Vieth et al, 2001[72]	Healthy Males and females	10 M, 23 F<br>10 M, 23 F	1,000<br>4,000	5	41±16<br>47±18	69±17<br>96±15	+28<br>+50
Heaney et al, 2003[81]	Healthy males	67 Supplemented	200<br>1,000<br>5,000<br>10,000	5	70±24<br>72±16<br>69±17<br>65±24	57±18<br>84±16<br>161±37<br>225±62	-12.5<br>+12<br>+92<br>+159

* 25(OH)D, 25-hydroxyvitamin D.

† Sunlight exposure was not discussed in the studies by Brooke et al, Cockburn et al, Delvin et al, Mallet et al, and Datta et al. Minimal sunlight exposure was controlled for in the studies by Ala-Houhala, Vieth et al, Heaney et al, and Hollis and Wagner.

‡ It is likely that the wrong dose was reported. The response observed is one that would be expected after supplementation with 10,000 IU/d for 3 mos.

Downloaded From: http://jamanetwork.com/pdfaccess.ashx?url=/data/journals/jama/934885/ by a Harvard University User on 03/30/2017

Figure 1. Mean 25(OH)D levels in African American, Caucasian, and Hispanic pregnant women in the three supplementation groups.

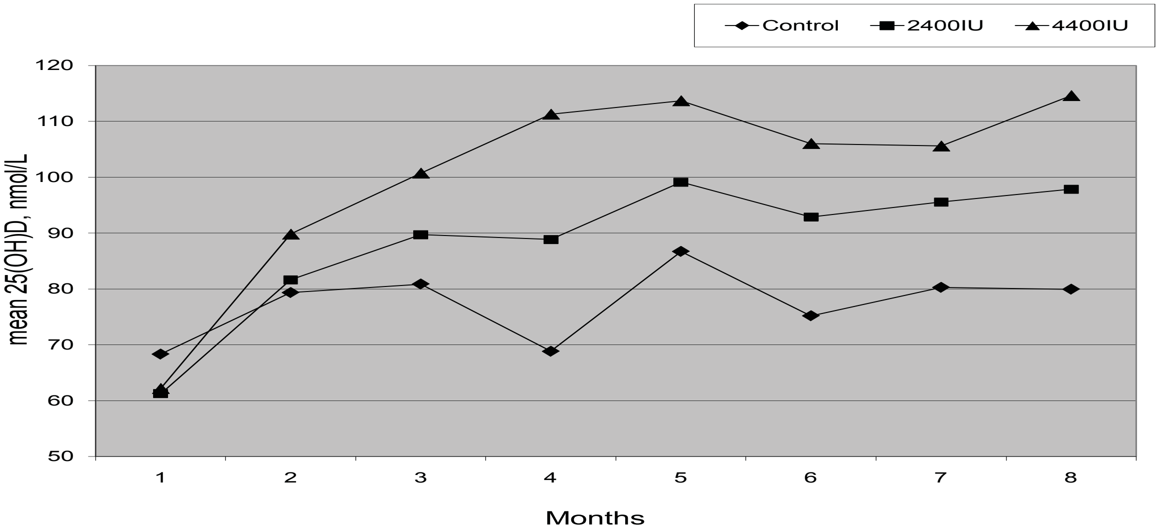

IV. Study Overview and Subject Selection

This is a multicenter, randomized, double-blind, controlled clinical trial that compares high-dose vitamin D_3 supplementation (4,400 IU/day) vs. usual dose vitamin D_3 supplementation (400 IU/day) in pregnancy to prevent asthma in the women's children. The study base will be all pregnant women from each Clinical Center who have a personal history of asthma and/or allergies or a history of asthma and/or allergies in the biologic father of the child (i.e. a "high risk" cohort for asthma and allergies in the child). Eight hundred seventy women (435 in each arm) will be followed through their pregnancy and delivery, and their infants will be followed up to the age of 3 yrs. *A*ll infants who are consuming < 1000 ml of formula per day will be offered vitamin D supplementation (400 IU/day) in accordance with the most recent recommendations from the American Academy of Pediatrics[82]*.* This includes all infants who are breastfed, either partially or exclusively, and all formula-fed infants until they consume at least 1000 ml of formula. The primary outcomes are doctor-diagnosis of asthma and recurrent wheezing at age 3 yrs. Due to dropouts from miscarriages, stillbirths, other pregnancy complications, and loss-to-follow-up, we expect that we will be left with 660 3-yr old children (330 in each arm) for the analyses of the primary outcome (see Specific Aims in Section II above).

Women who come into their Obstetrician's clinics for their first pre-natal visit will be screened on a daily basis by a Study Coordinator and a physician co-investigator will verify eligibility criteria.

Inclusion Criteria (applied to the pregnant women)

- Personal history of asthma, eczema, allergic rhinitis or a history of asthma, eczema, allergic rhinitis in the biological father of the child
- Gestational age between 10 and 18 weeks at the time of randomization
- Maternal age between 18 and 39 years
- Not a current smoker (defined as not having smoked for at least 1 month prior to enrollment) and not a user of other nicotine products (e.g. nicotine patch) for at least 1 month prior to enrollment
- English- or Spanish-speaking

Downloaded From: http://jamanetwork.com/pdfaccess.ashx?url=/data/journals/jama/934885/ by a Harvard University User on 03/30/2017

- Intent to participate for the full 4 years (through pregnancy and then until the 3rd birthday of the child)

Exclusion Criteria

- Not meeting Inclusion Criteria
- Gestational age >18 weeks
- Presence of chronic medical conditions: (i) Hypertension on medications, (ii) Type 1 or type 2 diabetes mellitus, (iii) Parathyroid disease, (iv) Uncontrolled thyroid disease, v) kidney stones, and (vi) Sarcoidosis
- Taking vitamin D supplements containing > 2,000 IU/day of vitamin D3
- Multiple gestation pregnancy (i.e. twins, triplets, etc.)
- Pregnancy achieved by assisted reproduction techniques (e.g. IUI, IVF)
- Current use of illicit drugs (defined as any use in the past 6 months prior to enrollment)
- previously enrolled in this study for a prior pregnancy
- Any major fetal anomalies detected prior to delivery
- Any condition, in the opinion of the Principal Investigator, that would inhibit compliance with the study medications or inhibit long-term participation in the trial
- Patient health questionnaire depression scale >/=15

Both arms of the study will get prenatal multivitamins which contain 400 IU vitamin D. Additional supplementation will occur in a double blind placebo controlled fashion with one arm receiving an additional 4000 IU of vitamin D (total vitamin D 4400 IU daily), and the other arm receiving a matching placebo. We will not exclude subjects who have “normal” levels at the beginning of the trial, since many studies have now documented that a large proportion of pregnant women have insufficient levels. Even women who may have normal levels may have insufficient levels at other times during their pregnancy (i.e. during winter). The 4400 IU dose has been shown to remove seasonal variation in vitamin D levels.

V. Recruitment. The recruitment goal for each site will be 290 subjects. As we anticipate miscarriages and stillbirths (about 8%[83]), and a 17-18% dropout by the end of the 3-year follow-up of the children, this number should give us the target of 220 3-yr old children per Clinical Center or a total of 660 3-yr old children at the end of the trial. The 3 clinical centers have considerable experience and a proven track record in recruiting participants for longitudinal studies of asthma. We expect that each center will be able to fulfill the recruitment goals in 18-24 months.

Screening visit: This screening visit will occur at the subjects' pre-natal visit before or around 18 weeks gestation. The study coordinator at each Clinical Center will obtain the schedules for all prenatal visits for the week, and scheduled visits will be reviewed for potential subjects. During the visit, the Study Coordinator/RA will describe the study, present the potential subject with a written description of the study, and review the eligibility criteria via a screening questionnaire and a study admissions criteria questionnaire. If the subject is eligible, does not meet any exclusion criteria, and expresses interest in the study, the subject will either be enrolled at this visit or an enrollment visit will be scheduled within one month.

Enrollment visit: A separate enrollment/randomization visit will be scheduled within one month of the prenatal visit, but not later than the 18th week of gestation. In some instances, enrollment will occur at the time the subject is screened. At this visit, the Study Coordinator/RA will review the study procedures and consent form. Once subject consent is obtained, the subject will be assigned a Study ID and several questionnaires will be administered, a skin pigmentation test will be performed, and blood will be drawn and stored for 25 (OH) D measurements. After questionnaires have been answered and the subject does not meet any further exclusion

Downloaded From: http://jamanetwork.com/pdfaccess.ashx?url=/data/journals/jama/934885/ by a Harvard University User on 03/30/2017

criteria, the subject will be randomized to one of the treatment arms (see Section VII.C. for details of randomization).

Follow-up: After enrollment, the Study Coordinator will note the subject's scheduled visits with her obstetrician and will make sure that urine samples are collected at each of these scheduled monthly clinical prenatal visits. Also at these monthly visits, a short maternal health questionnaire will be administered, MEMS cap information will be downloaded, and study medication and prenatal vitamins will be refilled. Additionally, the Study Coordinator and RAs will conduct a monthly review of electronic medical records to check for pregnancy complications. At 32-38 weeks gestation, in addition to the monthly routine, a blood draw, skin pigmentation determination, and a number of the questionnaires that were administered at the enrollment visit will be repeated. At delivery, cord blood will be collected and the Study Coordinator and RAs will collect information regarding the type of delivery, birthweight, and other anthropometric measures. After delivery, the research staff will make quarterly calls in the first year to inquire about the health and symptoms of the infant in addition to the type and frequency of feeding of the child and supplement use. A number of the questionnaires administered during pregnancy will also be re-administered to the mother. The mother and child will come in for 3 yearly follow-up visits, during which blood will be drawn, skin pigmentation tests will be performed, and additional questionnaires will be administered, as well as anthropometric measurements of the child. The flow chart for the trial is diagrammed in Figure 2. All questionnaires and measurements to be performed are summarized in Table 2.

Downloaded From: http://jamanetwork.com/pdfaccess.ashx?url=/data/journals/jama/934885/ by a Harvard University User on 03/30/2017

Figure 2. Flow Chart of VDAART Study

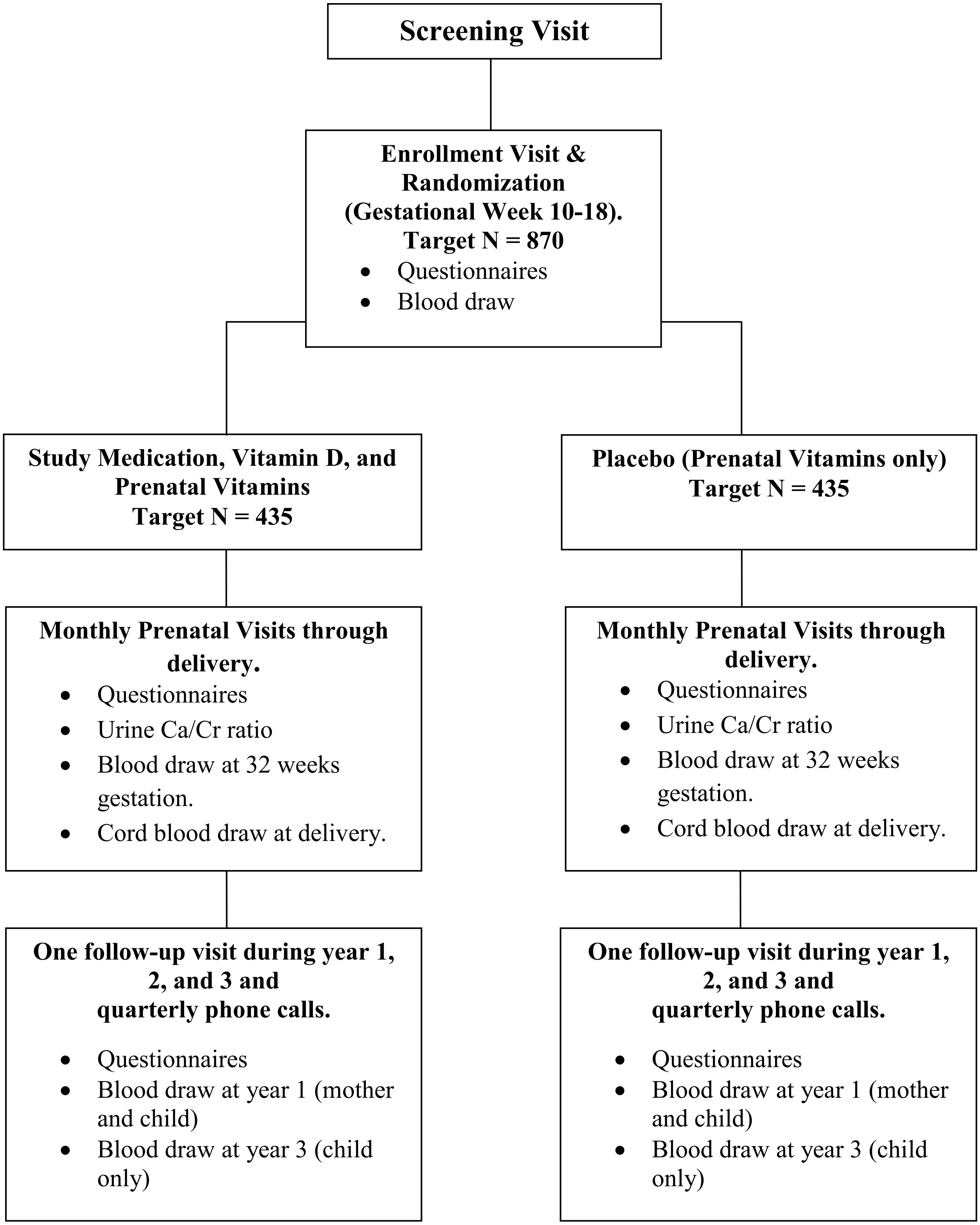

Downloaded From: http://jamanetwork.com/pdfaccess.ashx?url=/data/journals/jama/934885/ by a Harvard University User on 03/30/2017

Table 2. Summary of questionnaires and measurements for the trial.

	Enrollment (10-18 weeks gestation)	Monthly	Third Trimester (32-38 weeks gestation)	Delivery	Quarterly	6 Months after delivery	Year 1	Year 2	Year 3
Study Admission Criteria Questionnaire	x								
Maternal Questionnaire	x								
Food Questionnaire (Maternal)	x		x			x	x		
Food Questionnaire (Child)							x	x	x
Baseline Sun Exposure Questionnaire	x								
Follow-Up Sun Exposure Questionnaire			x				x (mother and child)	x (mother and child)	x (child)
Monthly Maternal Questionnaire		x	x						
Medical Record Review (electronic and/or paper)	x	x	x						
Determination of skin pigmentation	x		x				x (mother and child)		x (child)
Blood draw	x1 (mother)		x1 (mother)	x2 (cord blood)			x1,3 (mother and child)		x4 (child)
Urine for Ca/Cr ratio5		x	x						
Patient Health Questionnaire	x		x				x	x	x
Hardships Questionnaire	x						x	x	x
Labor and Delivery Form				x					
Quarterly Infant Follow-up Questionnaire					x		x	x	x
In-person visit	x		x				x	x	x
Anthropometric Measurements							x (child)	x (child)	x (child)
MEMS information download		x	x	x (after delivery)					

1 Mother: 25(OH)D, blood for DNA extraction and gene expression studies.

2 Child: 25(OH)D, total IgE, and blood for DNA extraction and gene expression studies.

3 Child: total IgE and 25(OH)D

4 Child: total IgE, eosinophil, and 25(OH)D

5 Urinary calcium, creatinine.

Downloaded From: http://jamanetwork.com/pdfaccess.ashx?url=/data/journals/jama/934885/ by a Harvard University User on 03/30/2017

VI. Data Collection

A. Definition of Outcome Variables

A.1. Asthma and recurrent wheeze. Asthma will be defined as a parent's report of physician's diagnosis at any time during the first three years of life, based on questionnaire responses. A diagnosis of asthma in early childhood is difficult, as only a third of children who wheeze before the age of 3 years will develop asthma. However, children who experience more frequent (recurrent) wheeze in early life are more likely to develop asthma[84, 85]. We will define recurrent wheezing as at least one report of wheezing in the third year of life plus any report of wheezing in any of the first two years of life. The primary outcome of the trial will be development of a doctor's diagnosis of asthma and/or recurrent wheeze. We will also consider other wheezing phenotypes. Persistent wheezing will be defined as ≥ 2 reports of wheezing in each of the three years of the child's life. Any wheeze will be defined as any report of wheezing in any of the three years of the child's life.

A.2. Eczema. Similarly to the case for asthma and wheeze, eczema will be defined based on responses to questionnaires. We will use the standardized questions from the International Study for Asthma and Allergy in Children which asks about an itchy rash with a specific distribution. We will also enquire about a physician's diagnosis of eczema.

A.3. Serum Total and Allergen-Specific IgE; Eosinophil count. Total serum IgE will be measured from cord blood samples, and from year 1 and year 3 samples from the children. In addition, allergen-specific IgE and eosinophil counts will be measured from the year 3 samples from the children. Total IgE will be measured in the Channing Laboratory by the UniCAP 250 system (Phadia AB in Portage, MI), with samples measured in duplicate. Serum from each child will also be assayed for IgE to 13 allergens (dust mite [*D. farinae*], cockroach [*Bla g*], cat dander, dog dander, rye grass pollen, ragweed pollen, *Alternaria tenuis*, Aspergillus, wheat, soybean, egg white, milk, and peanut) using the UniCAP system (Phadia AB in Portage, MI). For specific IgE, the enzyme-immunoassay is based on the sandwich technique, utilizing a solid phase (allergen-impregnated discs) for separation. Sensitization to common allergens can be detected by measuring serum IgE specific to those allergens. We will define sensitization to each allergen as a specific IgE level ≥ 0.35 IU/ml. Atopy will then be defined as sensitization to at least one of the 13 allergens included in the study. Total serum IgE is increased in atopic individuals. For the statistical analysis, total serum IgE will be transformed to a log natural scale, in recognition of the distribution of total serum IgE in human populations[86]. Total eosinophil counts will be determined at each clinical center's laboratory.

A.4. Respiratory tract infections. Lower respiratory tract infections (LRIs) will be defined based on parental responses to questionnaires. LRIs will be defined as a parental report of a physician's diagnosis of pneumonia, bronchiolitis, bronchitis, or croup (laryngotracheobronchitis).

B. Screening Questionnaire. This short questionnaire will be administered/obtained at the screening visit at the time of the first pre-natal visit to determine eligibility based on the presence or absence of asthma or allergies in the mother or in the biologic father of the child. If the subject or the biologic father has either asthma or allergies, then the Study Admissions Criteria Form that inquires about the other inclusion and exclusion criteria will be filled out.

C. Baseline Maternal Questionnaire. This questionnaire will inquire about details regarding her asthma and allergy symptoms, demographic information on herself and the biologic father of the child, and smoking in the home. For mothers with asthma, this questionnaire also includes

Downloaded From: http://jamanetwork.com/pdfaccess.ashx?url=/data/journals/jama/934885/ by a Harvard University User on 03/30/2017

questions from the Asthma Control Test (ACT)[87], a validated questionnaire inquiring about control of asthma in the past 4 weeks.

D. **Monthly Maternal Questionnaire**. This questionnaire will be administered to the mother by the Study Coordinator at the monthly scheduled prenatal visits. The mother will be asked to complete this health assessment questionnaire to ascertain:
a. Any change in her health status
b. Use of prescription medications
c. Use of over-the-counter preparations that may influence vitamin D and calcium homeostasis.
d. Use of cigarettes, alcoholic beverages and nutritional supplements
e. Level of asthma control, for those with asthma, using the ACT

E. Monthly Review of Medical Records (paper-based) and Electronic Medical Records (EMR)

Each month, the Study Coordinator or the RA will review the Obstetric medical record of each of the participants for any potential complication of pregnancy. Examples include but are not limited to the following: (a) Preeclampsia, (b) HELLP (Hemolytic anemia, Elevated Liver enzymes, Low Platelet count) syndrome, (c) Gestational diabetes, (d) Hypertension, (e) Preterm delivery, (f) Premature rupture of membranes (PROM), (g) Deep venous thrombosis or a coagulopathy, (h) Maternal infection such as varicella or herpes (1° or 2°), (i) Placental abnormalities such as previa or marginal abruption. For centers where EMRs are not complete, paper medical records will be reviewed and the relevant information extracted.

F. Measurement of 25(OH)D

Blood will be drawn from the mothers at randomization, at the in-person research prenatal visit at 32-38 weeks gestation, and at 1 yr after delivery. Cord blood will also be collected at delivery. Blood from the child will be collected at 1 yr and at 3 yrs. All specimens will be shipped to the DCC, where they will be logged in and processed. Measurement of 25(OH)D will be performed at the Channing Laboratory DCC as previously described[88]. Twenty five (25) microliters of serum or plasma (EDTA or Heparin) is required for the 25-OH-D extraction, however, 100 microliter samples will be aliquoted to allow for duplicate measurements on each sample. A chemiluminescence assay will be employed. The LIAISON® 25 OH Vitamin D assay (DiaSorin, Inc., Stillwater, MN) is a direct competitive chemiluminescence immunoassay (CLIA) for quantitative determination of total 25 OH vitamin D in serum or plasma. During the first incubation, 25 OH Vitamin D is dissociated from its binding protein and binds to the specific antibody on the solid phase. After 10 minutes the tracer, (vitamin D linked to an isoluminol derivative) is added. After a second 10 minute incubation, the unbound material is removed with a wash cycle. Subsequently, the starter reagents are added to initiate a flash chemiluminescent reaction. The light signal is measured by a photomultiplier as relative light units (RLU) and is inversely proportional to the concentration of 25 OH vitamin D present in calibrators, controls, or samples. In order to monitor assay performance each assay will include one quality control sample. The control will be treated as unknown specimen and multiple (5) determinations will be made. Assay variation will be determined and acceptable performance limits will be defined as ≤15 C.V. In addition 20% of the samples will also be re-measured in Dr. Hollis' laboratory in the Medical University of South Carolina.

.

Downloaded From: http://jamanetwork.com/pdfaccess.ashx?url=/data/journals/jama/934885/ by a Harvard University User on 03/30/2017

G. Urinary Ca^{++}/Cr ratios

At each monthly prenatal visit, mothers will provide a urine sample (“clean catch”). Because dehydration can affect urine calcium levels, mothers will be instructed to maintain good hydration throughout their pregnancy. Urine samples will be sent to each Clinical Center’s Clinical Chemistry Laboratory for urinary calcium and creatinine measurement, and the urinary calcium to creatinine ratio will be derived. Dr. Hollis has used this measure to monitor women on vitamin D supplements in his other trials and has shown that this is the earliest sign of impending toxicity.

If an elevated Ca/Cr ratio is found, the woman will be asked to stop the study pills. An elevated Ca/Cr ratio will be defined as ≥ 1.55, when measured on the mmol scale, or ≥0.55, when measured on the mg/mg scale. She will be asked to stop the study medication and to return for a repeat evaluation after being instructed to increase her fluid intake, to ascertain whether this was due to dehydration or true hypercalciuria. If the repeat test is normal she will resume the study medication and continue in the study. If the repeat test remains elevated, she will have further blood tests including serum 25(OH)D levels and calcium level, if these are within normal limits, she will restart her study medication and continue on the study. If her serum 25(OH)D and calcium levels are elevated, she will be withdrawn from study treatment and be further evaluated by her doctor as appropriate. This will be recorded as aserious adverse event. (Please see Section VII. J. for further details).

If there is a subsequent elevated urine Ca:Cr ratio, but it is a lower than the initial elevated urine Ca:Cr ratio, she will be reminded to maintain hydration by drinking water daily, and will continue on her study medication. If the subsequent elevated urine Ca:Cr ratio is greater than the initial elevated ratio, she will be asked to stop her study medication, and reminded to drink non-caffeinated fluid. The woman will return to the clinic center and have a blood draw for serum Ca and 25(OH)D measurement. If these are within normal limits, study medication will resume. If the values of either are out of normal limits, the subject will be withdrawn from study treatment, but continued to be followed under the intention-to-treat paradigm. A serious adverse event will be submitted to the DCC and she will be referred to her physician for follow up treatment.

H. Food Frequency Questionnaire. Maternal dietary intake of vitamin D will be assessed using a standardized, validated brief food frequency questionnaire[89, 90]. This questionnaire has been modified to apply to pregnant women and has been validated against the longer semi-quantitative food frequency questionnaire[89, 90]. Mothers will be asked to complete the FFQ on enrollment. A second questionnaire will be completed at the 32-38-week visit. Additional questionnaires will be completed at 6 months and at 1 year post-partum. Assessment of the responses to these questionnaires will help account for dietary sources of vitamin D in addition to the study intervention. In addition, the FFQ will measure other nutrients and supplements that may have confounding effects, and which we will be able to adjust in the analyses. Filled out questionnaires will be sent to the Harvard Nutrition Questionnaire Services Center at the Harvard School of Public Health for scanning and processing. Individual nutrients will be calculated using the Harvard nutrient composition database, which contains food composition values from the US Department of Agriculture, supplemented by other sources[91].

I. Sun Exposure Questionnaire. We will collect UV exposure history using a modification of the UV exposure questionnaire that is used in the Nurses’ Health Study[92]. Briefly, this questionnaire will inquire about UV exposure information such as average hours per week spent outdoors in summer and winter, sunscreen habits, and average times for sunlamp use or tanning salon attendance.

Downloaded From: http://jamanetwork.com/pdfaccess.ashx?url=/data/journals/jama/934885/ by a Harvard University User on 03/30/2017

J. Measurement of Skin Pigmentation. Mothers will have the degree of pigmentation/melanin measured at study entry, at 32-38 weeks, and at the 1 year visit using the Smart Probe 400 (IMS, Inc., Milford, CT) and a skin tone chart (IMS, Inc.). This is a spectrophotometer device that measures degrees of pigmentation on a continuous scale from 0 to 100, 0 being absolute black and 100 being absolute white. Each mother will have pigmentation measurements recorded from her forehead, exposed forearm, underarm, and abdomen, with two readings from each site averaged and recorded.

K. Stress and Depression Questionnaires.
Patient Health Questionnaire: The Patient Health Questionnaire (PHQ-9) Depression scale is being used in VDAART to assess depressive symptomatology in women. This is a 9-item scale with scores ranging from 0 to 27 with a general question about if these problems have interfered with their lives. A commonly used cut-off score of 15 or higher along with rating that it is "somewhat difficult" or greater in interfering with your life is considered clinically significant. The PHQ-9 has been shown to have reasonable sensitivity, specificity and positive predictive value for clinical depression in community samples. This severity score is the sum of the columns for questions 0-9. Question 9 is important since it asks about thoughts of self harm and any response of 1-3 would be considered clinically significant. If a subject's score is =/>15: 1) Prior to randomization: suicidal ideation or a score of 15 or higher on the depression scale questionnaire has been added as exclusion criteria to the study. The participant will be counseled by the Study Coordinator and the PI will be notified. The participant will then be referred back to her obstetrician for further psychiatric evaluation. 2) Post randomization: suicidal ideation or a score of 15 or more on the depression scale will be recorded in a specific study form and trigger 1) notification to PI and obstetrician and 2) a referral to a psychiatrist or psychosocial specialist. The findings of this report will determine whether the patient can continue in the study.
Hardships Questionnaire: These questionnaires are designed to see whether depression, financial strain, and perceived stress are effect modifiers of the immune system changes from vitamin D supplementation. These would be collected at the enrollment visit, and annually at the year 1, 2, and 3 visits since chronic stresses have been shown in other cohort studies to affect immune system function. Depression may also be effected by Vitamin D supplementation and sunlight, which would be a question that could be asked in this study.

L. Labor and Delivery Information and Questionnaires: At delivery, the research assistant will collect information on type of delivery (caesarian vs. vaginal), birthweight and other anthropometric measurers, need of oxygen, etc. Data from routine hospital newborn screen will be obtained for all infants.

M. Infant Follow-up Questionnaires. Every 3 months, we will administer a questionnaire to the child's caretaker (in most instances, this will be the mother) to obtain information on the neonate's general and respiratory health, with particular attention given to symptoms suggestive of asthma, wheeze, allergies, and atopic eczema. This questionnaire will also inquire about respiratory infections (upper, including ear infections, and lower tract infections, including bronchiolitis), medication use, particularly antibiotics, and smoking by adults in the home. Questions inquiring about breastfeeding, formula feeding, and introduction of solid foods will also be included. At the first year visit and each year thereafter, we will also inquire about the child's diet, using a short food-frequency questionnaire. We will also obtain several anthropometric measurements.

N. DNA Extraction and Samples for Gene Expression Studies: We will extract high molecular weight genomic DNA from the white blood cells from maternal peripheral blood and cord blood with commercially available PureGene Kits (Gentra Systems, Minneapolis, MN). Optical density

Downloaded From: http://jamanetwork.com/pdfaccess.ashx?url=/data/journals/jama/934885/ by a Harvard University User on 03/30/2017

(OD) will be measured to confirm DNA quality and quantity for each sample. This amount is sufficient to perform approximately 16,000 genotype assays using PCR technology (assuming a conservative use of 25 ng/reaction, although we routinely amplify single copy gene sequences from 10 ng of genomic DNA). Every sample undergoes additional quality control before use/release by subjecting it to PCR amplification. After DNA quantification, samples will be adjusted to TE buffer and partitioned into aliquots stored at -80°C. Information about quantity and storage of extracted DNA will be added as variables to each patient's database. DNA samples will then be stored at the Channing Laboratory for future genetic studies.

From the scheduled blood draws from the mothers and cord blood, we will include samples for gene expression studies. For mothers, two 2.5 PAXgene tubes at enrollment, the 32-38 week gestation visit, and at the child 1-yr visit. Two 2.5 cc PAXgene tubes will also be saved from the cord blood draw. These samples will be used to understand the genetics and gene expression of fetal development and asthma in future studies with a special focus on the relationship to vitamin D. While genetics and gene expression are not currently part of the specific aims of this protocol, it is imperative that samples be collected because the time window for appropriate collection is short (i.e. during pregnancy and delivery of the baby).

O. Manufacture and Distribution of Study Medications; the MEMS System

The study medications, including all prenatal vitamins, the vitamin D tablets, and the placebo tablets will be manufactured by Tishcon Corp. Founded in 1976, Tishcon Corp. (www.tishcon.com) is a leading manufacturer and marketer of vitamins, related dietary and herbal supplements, and private label non-prescription (OTC) pharmaceuticals in tablet, hard gelatin capsule, soft gelatin capsule and powder dosage forms. Tishcon is one of 12 soft gelatin capsule manufacturers in the United States. The pills and capsules will be sent in bulk from Tishcon to the DCC. The DCC will contract with the Brigham and Women's Hospital Pharmacy to package and label the pills in bottles. Once packaged and labeled with Study ID numbers, the pill bottles will be shipped to the respective Clinical Centers where a MEMS cap will be utilized on each assigned bottle. Random pills from each lot will be sent to Dr. Hollis' lab where the vitamin D amount will be quantified for quality purposes. Each participant will receive 2 bottles – one bottle will contain the standard prenatal vitamins with 400 IU vitamin D, and will be labeled accordingly, and the other bottle will contain the intervention pill containing 4,000 IU vitamin D or a placebo.

The MEMS System. The MEMS® - Medication Events Monitoring Systems is a pill bottle equipped with special cap that includes an electronic microchip that stores the date and time of each opening of bottle. The MEMS monitors are drug packages with integral electronic micro circuitry designed to compile the dosing histories of ambulatory patients' prescribed medications. Each monitor consists of a conventional medicine bottle fitted with a special closure that records the time and date of each opening and closing of the container through integrated microcircuitry. The MEMS® stores up to 3800 medication events in non-volatile EEPROM memory, allows wireless data transfer, fits standard pharmacy bottles 38mm-400, 42mm-400, and 45mm-400 thread closure, provides 36 months battery life, and is water-resistant and CE marked. Monitors are designed to be used by one patient with one drug. A Reader allows transferring the dose timing data from the MEMS to a MS-Windows based computer. The MEMS monitors have demonstrated reliability and validity and have been widely used with great success in clinical trial research[93], including NHLBI sponsored studies in the Childhood Asthma Research and Education Network[94, 95] and asthma clinical research sponsored by industry[96]. The MEMS® - Medication Events Monitoring Systems is manufactured by Aardex LTD (http://www.aardexgroup.com) with a subsidiary in California for ease of ordering.

Data from the MEMS system will be downloaded at the monthly prenatal visits and after delivery. The data from the pre-natal follow-up visit will be used to provide feedback to the

Downloaded From: http://jamanetwork.com/pdfaccess.ashx?url=/data/journals/jama/934885/ by a Harvard University User on 03/30/2017

participant, reinforcing those subjects with good adherence and providing extra teaching and encouragement for those with suboptimal adherence. The data from both pre-natal follow-up and post-natal downloads can be used to derive a summary adherence measure that can be used as a covariate in our analyses and also for secondary analyses that exclude any women who have taken less than 50% of the prescribed study medication.

P. Statistical considerations

P.1. Overview

This is a randomized, double blinded, multi-center, placebo controlled, clinical trial of prenatal vitamin exposure as a preventive measure to reduce the risk of asthma and recurrent wheeze in childhood. There will be approximately *870* pregnant women enrolled, targeting an evaluable study population of *660* women randomized to Vitamin D supplementation of 4400 IU or 400 IU (standard prenatal supplementation). The treatment arms will be balanced (approximately n=330 in each arm, with some fluctuation possible owing to differential miscarriage or loss to follow-up, both expected to be completely at random). The primary outcome is doctor-diagnosed asthma and recurrent wheeze at 3 years of age. Secondary outcomes include maternal 25(OH)D levels and children's allergy, doctor's diagnosis of eczema, and lower respiratory infection (LRI), at three years of age. The primary interpretive paradigm for analysis of this study is intent to treat.

P.2 Analytic plan

All analyses to be described will occur in a framework of continuous quality assurance and verification. Preliminary statistical analysis will be used to describe the univariate distributions of key measures of interest to detect outliers or data anomalies that need to be addressed by data editing or sequestration of doubtful entries. Additionally, these analyses will examine whether any important risk factors for childhood asthma are imbalanced across treatment arm, as well as assess whether differential loss to follow-up is present across treatment groups. Detailed blinded codebooks will be provided to investigators as data accumulate to facilitate efficient substantive verification of basic distributional summaries and joint distributions of measured factors.

P.2.a. Plan for *Specific Aim 1*

The hypothesis that the incidence of asthma and recurrent wheeze at age three years is reduced in the high-dose Vitamin D supplementation group will be tested using logistic regression, allowing for fixed effects of center. Additional analyses will consider the modification of vitamin D exposure effects by maternal and child demographic/behavioral factors such as maternal age, BMI and weight gain during pregnancy, maternal history of asthma/allergy, and other factors potentially associated with asthma and/or vitamin D exposure. The variables to be considered are summarized in Table 3. For variables that are measured post-natally, recall bias is a potential confounder among parents of infants diagnosed with asthma. However, the prospective nature of the data collection should limit the extent of this and other potential sources of bias. Due to the design of this clinical trial, we expect that the randomization process will distribute these variables equally between the intervention and control groups. However, we will check the distribution of these variables prior to conducting the analyses, and will adjust for variables that may be unevenly distributed across treatment and control groups.

Downloaded From: http://jamanetwork.com/pdfaccess.ashx?url=/data/journals/jama/934885/ by a Harvard University User on 03/30/2017

Table 3. Information on selected potential confounders of the relationship between 25(OH)D levels and asthma/recurrent wheeze in 3-yr old children

Prenatal variables	Socioeconomic status
	Maternal diet
	Maternal sun exposure behavior
	Maternal age and parity
	Maternal history of asthma and allergies
	Maternal pre-pregnancy BMI and weight gain in pregnancy
	Maternal medication use in pregnancy (antibiotics, acetaminophen, NSAIDs, etc.)
	Pregnancy complications (e.g. gestational diabetes, pre-eclampsia)
	Maternal depression
	Maternal stress
Delivery variables	Gestational age
	Mode of delivery (caesarian section, normal vaginal delivery)
	Birthweight, length, head circumference
	Oxygen use
	Need for NICU stay
Postpartum and infancy variables	Breastfeeding or bottle feeding
	Type of formula
	Timing and type of solid food introduction
	Daycare, including timing of and type of daycare (home vs. institution)
	Pets in the home
	Other siblings in the home (gender and number)
	Smoking in the home
	Childhood diet
	Sun exposure behaviors in the child
	Maternal depression
	Maternal stress

Letting M_i be an indicator function for the primary endpoint, asthma, for the ith subject, the model is:

$$\operatorname{logit}\Pr(M_i = 1 \mid x_i, a_i) = \alpha + \beta x_i + \gamma_1 a_{1,i} + \gamma_2 a_{2,i}$$

where x_i denotes an indicator function for level of vitamin D supplementation (0=400 IU, 1=4400 IU) in the ith subject, while $a_{1,i}$ and $a_{2,i}$ are indicator functions for study center. The specific aim for this outcome tests the hypothesis that β=0, asthma and recurrent wheeze incidence do not significantly differ across treatment groups, after adjusting for the potential effects of study center heterogeneity.

To assess modification of vitamin D exposure levels by other demographic and/or behavioral variables, the model is:

$$\operatorname{logit}\Pr(M_i = 1 \mid x_i, a_i) = \alpha + \beta x_i + \gamma_1 a_{1,i} + \gamma_2 a_{2,i} + \sum_k \delta_k z_{ki} + \sum_k \eta_k x_i z_{ki}$$

where z_{ki} represents the k measured covariates for each subject, and the other variables are as described above. This model addresses two hypotheses. First, main effects testing for behavioral/demographic covariates (Table 3), or the hypothesis that δ_k=0, will be used to examine whether maternal age, BMI, or other factors predict asthma and recurrent wheeze risk at age 3. More importantly, inclusion of any significant main effects will adjust for any distributional imbalances of these factors across the Vitamin D supplemented and placebo

Downloaded From: http://jamanetwork.com/pdfaccess.ashx?url=/data/journals/jama/934885/ by a Harvard University User on 03/30/2017

groups. Second, this model tests the hypothesis that η_k=0, or whether any maternal, child, or demographic/behavioral covariates modify the relationship between vitamin D intake and our primary outcome. To obtain a parsimonious model and limit multiple testing, only covariates with significant main effects and strong biologic plausibility for an exposure-dependent impact on asthma/recurrent wheeze risk will be considered for interaction testing with Vitamin D supplementation status. For example, only the prenatal and some delivery variables (e.g., gestational age, birthweight, length) listed in Table 3 will be considered, as the factors modifying the impact of Vitamin D supplementation on asthma and/or recurrent wheeze should occur temporally (i.e. in utero).

P.2.a.1. Interim Analyses for Specific Aim 1

For the primary aim of asthma and/or recurrent wheeze, as well as the other specific aims, we have developed an interim analysis plan to monitor this composite endpoint over the course of the study. The interim analysis plan, for all four specific aims, is discussed in section VII.K.

P.2.b. Plan for Specific Aim 2

For this analysis, our N will be 800, since most of the dropouts will be expected to occur after delivery. Thus, the expected drop in N due to the 8% miscarriage rate was applied. We will test the hypothesis that the dose of 4,400 IU per day is sufficient to get 90% of the women to a sufficient level of 25(OH)D of at least 75 nmol/L, primarily using a logistic regression model, with randomization group (i.e. intervention group vs. usual care) as the independent variable. The dependent variable will be 25(OH)D level at delivery. We will adjust our analyses for other covariates that may affect 25(OH)D level, such as sun exposure (from the SmartProbe and the sun exposure questionnaire), age, and diet (calculated vitamin D intakes from dietary sources).

P.2.c Plan for Specific Aim 3

Incidence of allergy, doctor's diagnosis of eczema, and lower respiratory tract infection (LRI) at age 3 will be individually assessed. Like asthma and recurrent wheeze, two of the three secondary outcomes are dichotomous endpoints, and will be analyzed using logistic regression. For allergy, logistic regression will be used for specific IgE and linear regression will be used for log transformed total serum IgE level. Both a primary analysis of the relationship between vitamin D and the secondary outcome of interest will be conducted, as well as additional analyses including demographic and environmental risk factors for allergy, eczema, and LRI. The primary analysis, as well as additional analyses testing main demographic/behavioral effects and effect modification, will be conducted along the lines of analyses described for Specific Aim 2. The covariates to be considered for main effects and interaction analyses will be identical to those outlined for Specific Aim 2.

P.2.d. Plan for Specific Aim 4

This specific aim investigates the immediate and long-term impact of vitamin D supplementation on circulating 25(OH)D blood levels. The immediate impact of Vitamin D on 25(OH)D will be assessed through maternal plasma and cord blood measurements taken at delivery. The long-term influence will be examined via longitudinal analyses using children's blood measurements obtained at ages 1 and 3.

The hypotheses that 25(OH)D levels in maternal plasma and cord blood will be higher in the 4400 IU group will be tested using standard linear regression. If necessary, 25(OH)D levels will be transformed to meet the distributional requirements for the analysis. The 25(OH)D levels in maternal plasma and cord blood will be treated as separate outcomes, although both testing procedures for each analysis will be similar and are described jointly below. As with specific aims 1 and 2, the primary analysis will examine the relationship between 25(OH)D and vitamin D levels, while the secondary analysis will include other important demographic and

Downloaded From: http://jamanetwork.com/pdfaccess.ashx?url=/data/journals/jama/934885/ by a Harvard University User on 03/30/2017

environmental factors related to 25(OH)D levels, such as climate and dietary vitamin D intake, testing for main effects and effect modification for these factors.

Letting y_i denote the 25(OH)D levels for each subject (as measured in mother's plasma for the first hypothesis, and cord blood for the second hypothesis), the model for the primary analysis is:

$$y_i = \alpha + \beta x_i + \gamma_1 a_{1,i} + \gamma_2 a_{2,i} + \varepsilon_i$$

where the covariates and parameters are as defined in Specific Aim 1. However, Specific Aim 3 tests the hypothesis that β=0, 25(OH)D levels do not significantly differ across the Vitamin D supplemented and control groups. All relevant covariates listed in Table 3 will be considered for the final model. For example, covariates measured post-natally would not be considered for inclusion in the analysis of maternal plasma or cord blood at delivery. Additionally, factors known to be directly associated with OH(25)D levels (i.e. sun exposure behavior, diet, ethnicity), will be included in the final model even if they are not statistically significant predictors of circulating vitamin D levels.

To assess modification of 25(OH)D levels by other demographic and behavioral variables, the model is:

$$y_i = \alpha + \beta x_i + \gamma_1 a_{1,i} + \gamma_2 a_{2,i} + \sum_k \delta_k z_{ki} + \sum_k \eta_k x_i z_{ki} + \varepsilon_i$$

where the parameters are as defined in Specific Aim 1. The k measured covariates for these analyses will be environmental, maternal, and child factors specific to 25(OH)D levels, such as sun exposure behavior, dietary intake of Vitamin D, and ethnicity. Additionally, any relevant covariates from Table 9 demonstrating main effects that have biologically plausible interactions with Vitamin D supplementation will be tested for effect modification. For both models, it is assumed that $\varepsilon_i \sim N(0,\sigma^2)$. The hypothesis that δ_k=0 will be used to examine whether any of these factors predict 25(OH)D levels, and the results will be used to adjust for any distributional imbalances of these factors across Vitamin D supplementation groups. As with the previous specific aims, we will also tests for effect modification via the hypothesis that η_k=0, or whether any maternal, child, or demographic/environmental covariates modify the relationship between vitamin D intake and circulating 25(OH)D levels in mother's plasma or cord blood. Additionally, we will follow the guidelines outlined in Specific Aim 2 to obtain the most parsimonious model.

The hypothesis that 25(OH)D levels in the child's blood will be higher in the 4400 IU group at birth, age 1 and age 3 will be tested using a repeated measures analysis framework such as generalized estimating equations.

P.2.d. Plan for Specific Aim 5

This specific aim investigates whether sufficient vitamin D supplementation in the pregnant mother is associated with reduced incidence of preterm birth (birth <37 weeks gestation) and/or preeclampsia, gestational hypertension, and/or HELLP syndrome compared to a usual care control group in VDAART.

The hypothesis that the incidence of preterm birth/preeclampsia, gestational hypertension and HELLP syndrome (PB/PE) is reduced in the high-dose vitamin D supplementation will be tested using logistic regression, allowing for fixed effect of center. Additional analysis will consider the modification of vitamin D exposure effects by maternal demographic/behavioral factors such as maternal age, BMI and weight gain during pregnancy, and maternal history of asthma/allergy, race, and other factors potentially associated with preterm birth/preeclampsia, gestational hypertension, HELLP or vitamin D exposure.

Letting M_i be an indicator function for the primary endpoint, PB/PE, for the ith subject, the model is:

$$\text{logit}\,\Pr(M_i = 1 \mid x_i, a_i) = \alpha + \beta x_i + \gamma_1 a_{1,i} + \gamma_2 a_{2,i}$$

Downloaded From: http://jamanetwork.com/pdfaccess.ashx?url=/data/journals/jama/934885/ by a Harvard University User on 03/30/2017

where x_i denotes an indicator function for level of vitamin D supplementation (0=400 IU, 1=4,400 IU) in the ith subject, while $a_{1,i}$ and $a_{2,i}$ are indicator functions for study center. The specific aim for this outcome tests the hypothesis that β=0, PB/PE incidence do not significantly differ across treatment groups, after adjusting for the potential effects of study center heterogeneity.

To assess modification of vitamin D exposure levels by other demographic and/or behavioral variables, the model is:

$$\text{logit}\Pr(M_i = 1 \mid x_i, a_i) = \alpha + \beta x_i + \gamma_1 a_{1,i} + \gamma_2 a_{2,i} + \sum_k \delta_k z_{ki} + \sum_k \eta_k x_i z_{ki}$$

where z_{ki} represents the k measured covariates for each subject, and the other variables are as described above. This model addresses two hypotheses. First, main effects testing for behavioral/demographic covariates, or the hypothesis that δ_k=0, will be used to examine whether maternal age, BMI, or other factors predict PB/PE. More importantly, inclusion of any significant main effects will adjust for any distributional imbalances of these factors across the vitamin D supplemented and placebo groups. Second, this model tests the hypothesis that η_k=0, or whether any maternal, child or demographic/behavioral covariates modify the relationship between vitamin D intake and our primary outcomes. To obtain a parsimonious model and limit multiple testing, only covariates with significant main effects and strong biologic plausibility for an exposure-dependent impact on PB/PE risk will be considered for interaction testing with vitamin D supplementation status. **Because of our fixed sample size we have chosen to combine PB and PE as our primary outcome.** Obviously we would like to explore the disease entities separately, and also examine the effect of race independent of vitamin D level. These analyses can be done using interaction terms and stratified analyses recognizing that we have lower power to detect these influences.

P.3 Multiple comparisons.

All pre-specified hypotheses will be tested individually at the 0.05 level of significance, using a conservative two-sided alternative hypothesis (even when the stated hypothesis is one-sided). More specifically, the primary and secondary hypotheses of Vitamin D supplementation and Asthma/Recurrent Wheeze, Allergy/Eczema/LRI, will not be adjusted for multiple comparisons, since they have been defined a priori and are limited in scope. Additionally, an interim analysis plan has been developed for the primary outcome to adjust for the number of times (3) asthma and/or recurrent wheeze will be analyzed over the study period. Since 25(OH)D status is being examined in 5 separate analyses (maternal plasma at 26-28 weeks, maternal plasma at delivery, cord blood at delivery, infant plasma at years 1 and 3), this analysis will be adjusted for 5 multiple comparisons (i.e., using Bonferroni correction). Finally, any findings with respect to interaction terms (i.e., exploring whether environmental and demographic covariates affect the relationship between Vitamin D supplementation and the outcome of interest) for any of the hypotheses will be adjusted (i.e., using FDR methodology) for the number of models tested, and interaction testing will be limited to those defined in sections D.14.2a-c, in order to limit the potential for spurious findings. Any serendipitous findings coming from exploratory analyses will be clearly reported as such, and test statistic p-values will be adjusted by the number of exploratory hypotheses considered (using FDR or another appropriate procedure for multiple comparisons adjustment).

Downloaded From: http://jamanetwork.com/pdfaccess.ashx?url=/data/journals/jama/934885/ by a Harvard University User on 03/30/2017

Q. Power and sample size

Q.1. Power for Specific Aim 1. The trial is designed to detect a *25*% reduction in asthma and recurrent wheeze incidence at 3 years in the vitamin D supplemented (4400 IU) group. Based on the observational results in the Viva Study, we estimate that the incidence of asthma and/or recurrent wheeze in the standard dosing group (400 IU) will be 40-50%. This incidence estimate is based on observations from Project Viva and the URECA Project**. In project Viva, the prevalence of persistent wheeze is 44% and of asthma is 56.2% in children born to families with parental history of asthma (about twice the prevalence in children without family history)**. These estimates are similar to those observed in the URECA Project in children who have turned 2 yrs old. The children in that cohort had a family history (mom or dad) of asthma or allergies. **In the URECA cohort, 99 of 194 (51.0%) children who have reached their 2nd birthday, have experienced "wheezing or asthma by age 2."** These observed rates in 2 cohorts give us confidence that we will see similar rates when we recruit the high-risk (for asthma and allergies in the child) pregnancies for this clinical trial. We anticipate an 8% miscarriage rate[83] and 17-18% LTF among remaining subjects over the study period, resulting in an estimated sample size of 660 subjects (330 per arm) at the end of the study period. Each power estimate is based on 10,000 simulations. The simulations incorporate an interim analysis plan using the Lan-Demets spending function described in section VII.M, although the power may be slightly lower than the estimates presented here. For our group sequential design, we plan to use a Lan-Demets spending function with four approximately equally-spaced looks (with respect to the amount of information collected) and O'Brien-Fleming type bounds. The software program LANDEM (http://www.biostat.wisc.edu/landemets/)[97] was used to calculate the stopping boundaries, which were |z|=4.3329, 2.9631, 2.3590, and 2.0141 at the first, second, third, and final looks, respectively. As noted in N.2.A.1, Lan-DeMets procedure provides a spending function approach which does not require pre-specification of the frequency or timing of interim looks. Therefore, the thresholds will be adjusted if the recruitment targets vary of if the need for more frequent monitoring arises. The hypothesis tests are conducted with two-sided alternatives (i.e. a two-sided p-value), The power reflects the proportion of replicates where the Wald test statistic from the hypothesis test of β_1=0 is less than the stopping boundary specified for the first, second, or third looks, where β_1 is the coefficient for Vitamin D supplementation status from the logistic regression model outlined in section,N.2.a. The replication is considered a 'success' if the stopping boundary is exceeded at any of the analysis times. In the simulations, the incidence of asthma and/or recurrent wheeze at the first and second interim looks is proportionate to the overall incidence expected at the end of the study period (i.e. at the first look, the incidence is one-third of the specified rate; at the second look, it is two-thirds). We do not expect incidence rates of 40-50% to be achieved until the third year of follow-up; therefore, the simulations reflect the approximate amount of information anticipated at each interim analysis.

Finally, in the simulation studies, we also incorporate study heterogeneity into the model by varying the incidence of asthma and/or recurrent wheeze in the control group (400 IU Vitamin D) across study sites. The incidence varies by an absolute difference of 10% (i.e., for a mean incidence of 50%, incidence rates varied from 45-55% across study sites). Thus, the absolute reduction in incidence observed in the treated group (4400 IU Vitamin D) will also vary according to baseline incidence in the control group [i.e., assuming a 30% reduction in incidence, a site with a 50% incidence rate in the control group will have a 35% incidence rate (a 15% decrease) in the treated group, while a site with a 45% incidence in controls will have a 31.5% incidence rate (a 13.5% decrease) among supplemented subjects].

Downloaded From: http://jamanetwork.com/pdfaccess.ashx?url=/data/journals/jama/934885/ by a Harvard University User on 03/30/2017

Table 4. Power to detect a *20-30*% reduction in asthma and recurrent wheeze for *660* patients in a multi-site study*

Mean Incidence of Asthma and/or Recurrent Wheeze in 400 IU group	Percent reduction in Asthma and/or Recurrent Wheeze in 4400 IU group		
	30%	*25%*	*20%*
**0.50**	*0.974*	*0.906*	*0.726*
**0.45**	*0.950*	*0.837*	*0.653*
**0.40**	*0.901*	*0.773*	*0.574*

*18% LTF and 8% miscarriage rate

We anticipate that there will be excellent power *(91%)* to detect a *25*% reduction in asthma, assuming a *50*% incidence of asthma/recurrent wheeze in the 400 IU dosing group. Even if the children in this study have a lower incidence of asthma and recurrent wheeze than previous cohorts (i.e., 40-45%), we will still have good power (77-84%) to detect a 25% reduction in asthma and/or recurrent wheeze. To assess the potential impact of study center heterogeneity and interim analyses on power, power calculations were conducted without adjustment for study-center and with a single analysis time point at the end of the study period (with a two-sided alpha=0.05). The calculations were performed in STPLAN (http://biostatistics.mdanderson.org), using an arcsine approximation for the binomially distributed outcome. Overall, the power estimates were consistently similar to the empirical estimates (shown in Table 5), with at most a 2% difference in absolute power. Therefore, we do not anticipate that multiple study sites or incorporating interim analyses will considerably reduce study power.

Q.2. Power for Specific Aim 2. We expect that there will be 400 women in each arm for this analysis due to the 8% miscarriage rate. We do not expect there to be significant LTF by delivery. Like the analysis plan for Specific Aim 1, we plan to incorporate interim analyses, using a Lan-Demets spending function with O'Brien-Fleming type bounds. For this specific aim, we plan for three looks, which results in stopping boundaries of |z|=3.7103, 2.5114, and 1.993, for the first, second, and final analysis, respectively. As above, the thresholds will be adjusted if the recruitment varies from the expected targets. As shown in Table 5 below, we will have excellent power to detect an increase of 20-60% in the proportion of mothers with 25(OH)D levels > 75 nmol/L between the intervention and the control group. The power calculations did not explicitly account for the interim analyses, however, the power should still be >0.999 even after distributing the alpha level across multiple looks.

Table 5. Power to detect a 20-60% absolute increase in the proportion of mother's with *25(OH)D* > 75 nmol/L for 800 patients in a multi-site study*

Proportion of mothers with 25(OH)D > 75 nmol/L in 400 IU group	*Proportion of mothers with 25(OH)D > 75 nmol/L* in 4400 IU group		
	90%	*80%*	*70%*
**0.50**	*>0.999*	*>0.999*	*>0.999*
**0.40**	*>0.999*	*>0.999*	*>0.999*
**0.30**	*>0.999*	*>0.999*	*>0.999*

* 8% miscarriage rate

Downloaded From: http://jamanetwork.com/pdfaccess.ashx?url=/data/journals/jama/934885/ by a Harvard University User on 03/30/2017

Q.3. Power for Specific Aim 3. The estimated incidence of doctor's diagnosis of allergy (IgE), eczema, and lower respiratory tract infection (LRI) at year 3 in the Viva cohort are 30%, 36%, and 45%, respectively, among the children with a parental history of asthma or allergies. Estimates of eczema and LRI differ from those submitted in the previous grant application, which were based on incidence of these secondary outcomes among all children (including those without a parental history of asthma and allergies). Additionally, information on allergy incidence in Project Viva is now available. We anticipate a 30%-*45*% reduction in each of these outcomes in the high-dose vitamin D supplementation (4400 IU) group, though a larger range is provided in table 6 below. Each power calculation is based on 10,000 simulations, and is identical to the simulations described above, except that interim analyses are not planned for the secondary outcomes, and the studies are powered to detect an effect at the end of the three year follow-up period, with a two-sided alpha level of 0.05. As with the methods described above, these simulations incorporate an effect for study center heterogeneity in baseline incidence of secondary outcomes (i.e., incidence across sites varies by 10%) and 8/18% losses to miscarriage and follow-up/miscarriage, respectively.

This specific aim has an interim analysis plan that is identical to the procedure outlines for specific aim 1. While the power estimates below do not directly incorporate the interim looks, we feel that the power reduction will be modest. With a 30% reduction in incidence, we anticipate that there will be good power (83.4%) to detect a clinically important difference in risk of eczema. Due to the lower incidence of allergy, a 35% reduction is required to have very good (89.0%) power. Additionally, since LRI is more prevalent at age 3, only a 25% reduction is required to have 85.8% power. Even if the incidence of these outcomes is 5% lower than the estimated rates in Viva, there will still be sufficient power to detect an effect of treatment. Overall, there is strong evidence that we will be adequately powered to detect a risk difference in treatment group (4400 IU Vitamin D) for the secondary outcomes, in addition to our primary outcome of interest, asthma and recurrent wheeze.

Table *6*. Power to detect a *25-35%* reduction in *secondary outcomes for 870 subjects in a multi-site study, (effectively 660 patients, assuming an 8% miscarriage rate and 18% loss to follow-up)*

Mean Incidence of secondary outcome in 400 IU group	***Percent reduction in secondary outcome in 4400 IU group***		
	35%	***30%***	***25%***
0.50	*0.998*	*0.982*	*0.913*
0.45	*0.992*	*0.958*	*0.858*
0.40	*0.973*	*0.906*	*0.779*
0.35	*0.936*	*0.845*	*0.691*
0.30	*0.890*	*0.736*	*0.608*
0.25	*0.795*	*0.654*	*0.531*

Q.4. Power for Specific Aim 4. The research to date on the impact of vitamin D supplementation on 25(OH)D is presented in Table 1. As noted in the table, only 4 studies have been conducted on pregnant women, all had fewer than 100 subjects, and the maximum daily dose for these studies was 1,600 IU. The only data available on 25(OH)D status in the supplementation ranges we are proposing has been provided by Dr. Bruce Hollis (unpublished data, see section III). In a study of 347 pregnant women (African Americans. Hispanic, and Caucasians) on either 4000 IU (supplemented) or 400 IU (control) of Vitamin D daily, the maternal plasma 25(OH) D levels were 115 nmol/L in supplemented subjects, and 80 nmol/L in controls after 8 months of supplementation, an overall difference of 35 nmol/L. The estimated standard deviation for 25(OH)D levels in these ranges is 40.

Based on the observed differences/variation in 25(OH)D levels, we used simulation studies to estimate the power to detect a mean difference of 7.5 – 37 nmol/L across the supplemented and control groups in maternal plasma at 26-28 weeks, as well as maternal plasma and cord blood at delivery, using an estimated standard deviation of 40. We anticipate that the difference in 25(OH)D levels in maternal plasma across treatment groups will be less

Downloaded From: http://jamanetwork.com/pdfaccess.ashx?url=/data/journals/jama/934885/ by a Harvard University User on 03/30/2017

pronounced at the 26-28 week measurement period (e.g., 12.5 – 25 nmol/L), and we do not expect to observe a significant difference by supplementation status in circulating vitamin D in infants at 1 and 3 years of age, since no post-natal treatment intervention is planned. The simulations were generated in a similar manner to method described for Specific Aim 2, except that linear regression (rather than logistic) was used to model the relationship between circulating vitamin D and vitamin D supplementation status. Study heterogeneity was incorporated into the model by allowing 25(OH)D levels to vary by 25 nmol/L across study sites. Since this phenotype will be measured five times throughout the study period, the power was defined as the proportion of replicates with a Wald test statistic (i.e., testing the null hypothesis that the coefficient for vitamin D supplementation status is 0) 2-sided p-value was less than 0.01 (0.05/5). Additionally, this specific aim will include 2 interim and a final analysis, which will be identical to the procedure outlines for specific aim 2. While the power estimates below do not directly incorporate the interim looks, we feel that the power reduction will be modest. The results are shown in Table 7.

Table 7. Power to detect a 7.5 – 37 nmol/L difference in 25(OH)D for 870 subjects in a multi-site study, (effectively 660 patients, assuming an 8% miscarriage rate and 18% loss to follow-up).

Mean change in 25(OH)D levels in 4400 IU group (nmol/L)	Power
**7.5**	0.444
**12.5**	0.925
**25**	0.999
**37**	0.999

For a mean difference greater than 7.5 nmol/L, we anticipate having excellent power to detect a difference in 25(OH)D status across treatment groups. This difference of at least this magnitude should be observed in both maternal plasma measurements and cord blood, given the preliminary data (a difference of 35 nmol/L in maternal plasma) provided by Dr. Hollis. To ensure that the results were not affected by incorporating an effect for study center, the simulations were conducted assuming homogenous effects across study sites. The power estimates were within 1.5% of those provided above, and are likely due to variation in estimates via simulation. Therefore, we are confident that we have excellent power to achieve this specific aim.

Q.5. Power for Specific Aim 5.

The trial is 84% powered to detect a *50%* reduction in PB/PE in pregnant mothers in the vitamin D supplemented (4,400 IU/day) group. We estimate that the incidence of preeclampsia and/or spontaneous preterm labor in the standard dosing group (400 IU/day) will be *10%*, resulting in an estimated sample size of 435 subjects per arm at the end of the antenatal period. Our sample size simulations, show that we will have excellent power to detect a 50% reduction in preeclampsia and/or preterm labor in the vitamin D supplemented group which is what we anticipate based on our preliminary data.

Q.6 Missing data considerations

It is expected that missing data will be infrequent, that the treatment is sufficiently benign that any missingness would be unrelated to treatment group, and that the outcome ascertainment process is compatible with standard medical care for children. Thus it is reasonable to predict that missing data would be missing "completely at random" (MCAR) and that inference based on the complete cases would be valid, though only slightly inefficient, if missingness is rare (less than 5%) . Methods are available to assess the compatibility of response patterns with the MCAR condition (Chen and Little, 1999), and these will be used to diagnose the missing data situation. If missingness is not rare, and it is desired to improve the

Downloaded From: http://jamanetwork.com/pdfaccess.ashx?url=/data/journals/jama/934885/ by a Harvard University User on 03/30/2017

efficiency of the analysis, two possible approaches to handling the imputation are described below.

Approach A: Impute the overall (ignoring treatment assignment) incidence of asthma and recurrent wheeze (or the incidence of the secondary outcome of interest) for children that are lost to follow-up, and analyze the resulting imputed dataset as if complete. This is a crude but very simple approach that is often proposed. Approach B: Use subject-specific modeling to determine an imputation distribution for those who drop out. Specifically, model the risk of asthma and recurrent wheeze (or secondary outcome) among completers as a function of baseline covariates, ignoring treatment assignment. Use the covariates z_i observed on dropouts to predict logit[Pr(M_i|z_i,)]. The predicted estimate is then perturbed by repeatedly drawing randomly from a distribution whose variance is the sample variance in the group of completers. Both analyses will be conducted to examine the similarity of results to the complete case analysis. If the results are consonant, for simplicity, the complete case analysis will be reported along with any variations in findings from the described imputation strategies.

VII. Study Implementation and logistics

A. Clinical Center Organizational Structure and Infrastructure

Each Clinical Center has a set organization structure with clearly defined personnel roles, a well-maintained computer infrastructure, and plans to establish numerous mechanisms to promote the flow of information between study center sites and the DCC.

Dr. Strunk and colleagues at the Washington University in St. Louis, MO has been designated as the lead Clinical Center for this grant. Dr. O'Connor at Boston University Medical Center and Dr. Zeiger at Kaiser Permanente and University of California at San Diego are the PIs of their respective Clinical Centers. Dr. Strunk will schedule and lead regular conference calls to discuss the processes of recruiting, adherence, and retention throughout the trial, and will report directly to Dr. Weiss at the DCC.

Because recruitment of participants will take place in Obstetrics Clinics, it will be crucial for each Clinical Center to establish ties and obtain cooperation from their respective Obstetric practices. All three centers have working relationships with the Obstetric practices from prior recruitment efforts. In the St. Louis Center, Dr. George Macones will support the trial by setting into place the system to recruit the pregnant women and obtain the necessary samples of peripheral blood during the prenatal visits and peripheral and cord blood at the time of delivery as well as obtaining urine to be measured for creatinine and calcium as safety for vitamin administration during pregnancy. Dr. Macones will assure that the OB practitioners are familiar with the trial and will support enrollment of their patients into the trial. Dr. Linda Heffner, MD, PhD (Chair, Dept. of OB and Gyn) and Ms. Margaret Shepherd, CNM (OB Department Manager), will fulfill the same roles for the BUMC and Kaiser Permanente San Diego Clinical Centers, respectively.

In each Center, a Study Coordinator will be responsible for recruiting pregnant women into the trial at the time of their first visit to the OB offices and retaining them during the 4 years of their involvement in the trial. Specific activities of this Study Coordinator will include but not be limited to:

- Working with the clerical staff of the OB department to assure that a brochure describing the vitamin D trial is included in the materials sent to the pregnant women giving information about the first OB visit.
- Working with the staff at the OB offices to determine when eligible women will have their first OB visit and being in the clinic to meet the women to give them information about the study, determine their level of interest, perform appropriate screening, give the informed consent and arrange a time for discussion of the trial and the consent and answer any questions.

Downloaded From: http://jamanetwork.com/pdfaccess.ashx?url=/data/journals/jama/934885/ by a Harvard University User on 03/30/2017

- Make an appointment in the Study Clinic for enrollment and initiation of the study medication. The trial will also provide standardized prenatal vitamins, which will be initiated at this visit.
- Remain in contact with the participants via scheduled phone calls and as needed for problem solving about taking study medication and the prenatal vitamin.
- Work with the staff in labor and delivery to establish a system for collection of peripheral blood from the mother to be used for assay of vitamin D and the cord blood for assay of vitamin D and for use in extraction of DNA to be used in the genetic studies.
- Visit the now new mother and her baby after delivery to deliver a small gift and further develop the relationship that will assure long-term follow-up of the child with the quarterly phone calls and annual visits.

Each Center will also have a second Study Coordinator or Study Nurse who will be a partner of the other research patient coordinator in performing all the duties listed to assure recruitment and retention of the women and their offspring.

B. DCC Organizational Structure and Infrastructure

The DCC has a set organization structure with clearly defined personnel roles, a well-maintained computer infrastructure, and plans to establish numerous mechanisms to promote the flow of information between study center sites and the DCC.

The DCC will be chaired by Dr. Scott Weiss and Dr. Augusto Litonjua, and the statistical and informatics component will be managed by Dr. Vincent Carey. The DCC informatics operations will be executed by Dr. Carey's staff, which has an outstanding track record running multicenter trials funded by AHRQ and NHLBI since the mid 1990s. Nancy Laranjo will be the Senior Data Manager for the trial, along with, Deputy Data Manager, John Ziniti, Senior Programmer, and Christopher Garcia, Administrator. In consultation with Dr. Carey, Ms. Laranjo will specify the general communications and data archive workflows for the support and programming staff members. As the design of forms and general data flow processes evolves, prototypes and "dry run" exercises will be carried out with the clinical sites to verify the soundness and feasibility of the operations plan. The detailed task sets will be noted in the DCC Manual of Operations, which will be available through the internal study web site. The DCC will have full staff meetings attended by one of the PIs every month, and basic staff meetings every week. In addition, the chairs of the DCC will have a conference call with the PIs of the Clinical Centers once a month to obtain updates on recruitment and progress of the trial, and discuss any issues that pertain to the conduct of the trial.

C. Randomization and Treatment Assignment

Participants who consent and are deemed eligible to participate will be randomized by the Data Coordinating Center (DCC) to either of the 2 arms of the trial and will be sequentially assigned a six-digit Study Identification Number (Study ID). The first digit of the Study ID will identify the site (St. Louis, San Diego, or Boston) and the next four digits will identify the participant; and the last digit will be a check digit that is calculated from a formula to ensure the accuracy of data entry. Only those who indicate their intention to follow-up until the child is 3 years old will be randomized. Randomization will be performed prior to enrollment of any study participants by the DCC using a system that automates the random assignment of treatment groups to Study ID numbers. The randomization scheme will employ permuted blocks, and one blocked allocation list per stratum (study site and racial/ethnic group) will be generated. That is, separate permuted block randomization lists will be maintained for each racial/ethnic group – White/Non-Hispanics, White/Hispanic, Black (including Hispanic Black), and Other – within each study site. This will ensure that within each site, there is approximate balance across arms over the course of enrollment, and that within ethnic groups within site, there is also approximately

Downloaded From: http://jamanetwork.com/pdfaccess.ashx?url=/data/journals/jama/934885/ by a Harvard University User on 03/30/2017

equal representation on both arms. A woman who is deemed eligible will receive the next available Study ID number for her given study center site and ethnic group. The Study ID will be linked to the women's Screening ID. It should be noted that women who are deemed ineligible will not receive Study IDs (i.e. randomization to a treatment). This will help ensure balance in treatment and control groups by not 'wasting' randomization assignments on ineligible subjects.

C.1. Race and Ethnicity. Race and ethnicity will be collected according to NIH categories. Participants will be asked to first categorize their ethnicity as either Hispanic or not Hispanic. They will then be asked to categorize their race as either White, Black or African-American, Asian, Native Hawaiian or other Pacific Islander, American Indian/Alaskan Native, or Other, If the participant categorizes herself as Black or African-American, she will be considered Black for purposes of randomization regardless of any other ethnic or racial category she has picked. If the participant categorizes herself as non-Hispanic and White, she will be classified as White/non-Hispanic. If she picks Hispanic but not Black, she will be classified as White/Hispanic. Any other racial or ethnic combinations will be classified as Other.

D. Trial Website

Each Center's participants will have access to the trial website. The DCC will establish a website with both public and private (SSL regulated, LDAP database of all study investigators or support staff needing website access will be established with private passwords) web portals at the beginning of the recruitment period. The public pages will provide information on the scope and background of study to assist in recruitment. The private pages will allow for general posting of any protocols changes or announcements, current versions of forms, minutes of phone calls, manuscripts in development, allow investigators to monitor recruitment process, and provide other general information. Patient-level treatment information, medical history, or endpoint information will not be linked to the website. The website will be maintained throughout the trial and closeout period, and aggregate study findings will be posted at the end of the trial.

E. Data management and tracking

Clinical data from screening (first prenatal visit), each subsequent pre-natal visit, delivery day, quarterly follow-up questionnaires, and clinic assessment visits at 1, 2, and 3 years are entered on forms at each study center at the time of the patient visit/contact. The forms will be reviewed by the center coordinator, and uploaded to the internal study website.

Calcium/creatinine ratios will be conducted by the study center laboratory on the day of the patient visit. The calcium/creatinine ratios will be reviewed by the laboratory head at each center. Alert values will be established and if reached will lead to immediate contact to the Study Coordinator and contact with the participant will be attempted within 24 hours of identification. Blood draws for 25(OH)D levels will be sent from the study center laboratory to the DCC on a weekly basis, where they will be logged in and aliquots created for 25(OH)D assays.

Upon receipt on collected forms, all data forms (lab work and patient questionnaires, clinical assessments, etc.) will be downloaded from the internal website and double entered at the DCC with range, logic, and missing data checks built into the data entry software application. The data entry software will be configured to be schema-driven, so that an abstract schematic design is at the base, and the concrete (XML) schema that specifies form content and logic is automatically transformed to a functional data entry application. We currently use Altova XMLSpy and Microsoft InfoPath technologies to implement the schema-driven design. Forms with missing (illegible entries will be considered missing for the purposes of data entry) and/or inaccurate (i.e. out-of-range) entries will not be accepted into the system database, but will be cycled back to the site for correction.

Downloaded From: http://jamanetwork.com/pdfaccess.ashx?url=/data/journals/jama/934885/ by a Harvard University User on 03/30/2017

For clinical data, the data monitor will send weekly reports of missing or inappropriate entries to the study project coordinators for clarification and resolution. Additionally, cross-form edit checks will be performed on a monthly basis, to detect unresolved problems. Data inconsistencies occurring across forms will be resolved with the assistance of study center staff. Standardized edit reports that summarize problems in the database provide an additional method of assuring data quality. To ensure accuracy of patient identification numbers (in particular, minimizing transposition error), digit checks will be utilized. Digit checks will identify whether there are inconsistencies in patient identification numbers across collection time points. Once data have been verified, data will be archived on a monthly basis as a SAS dataset. Data will be stored in coded form (i.e. numeric coding for ethnicity or gender) using standard codebooks currently implemented

For each patient, a data collection tracking query will be developed, which will contain expected time points for each data form/lab work, based upon patient's initial enrollment date. Forms more than one week late (from enrollment to delivery) or one month late (after delivery to year 3) will be flagged as missing, and a query will be generated for each site that contains a list of patient IDs and their outstanding forms/lab data. The data manager at the DCC will follow-up with site coordinators to track down any missing forms. For patients at-risk of dropping out (i.e. missed appointment or non-response to questionnaire request), the study coordinator or research assistant will make repeated attempts to contact the patient. A subject will be dropped only with explicit withdrawal request by the pregnant woman (initially) and later, the parent of the infant/child. Even with missed appointments or calls, every effort will be made to complete the final in-person visit when the child is 3 yrs old.

F. Data Security and Confidentiality

All computer files are stored in password-controlled accounts on DCC computers. There will be an SSL regulated private web portal established for the study at the DCC, LDAP database of all study investigators or support staff will be established with private passwords allocated to all who have a need to examine the study web site

Each participant will be assigned a unique study identification number. Once these data are linked (after a subject successfully enters randomization phase from screening), the study identification number will be the primary identification number. The data coordinating center and trial statisticians will have access to only the unique identification number and therefore cannot link names to data. Files of study data will include the screening and study identification number but not participant names. Any hardcopy of data forms obtained by the coordinating unit (for example, as part of spot-checking for quality control) will be maintained in locked file cabinets. If a participant withdraws from participation at any point in the trial, and requests withdrawal of any information from the database, the clinical center staff will forward the study identification number to the DCC and all files pertaining to that identification number will be removed and destroyed.

At the clinical centers, data are collected on forms with fields for study identification number but without a field for the participant's name. Still, separate lists of participant names, identification numbers, and contact information are used during the study to maintain contact with participants and eventually to provide feedback on study and personal results. These lists will only be available to Center Co-PIs and staff with direct patient contact. All hardcopies of forms are stored in locked file cabinets. All computer files are password protected. Participant results will only be released to the participant unless he/she provides written approval to release data (e.g. laboratory results to personal provider). Data will only be presented and published in aggregate, i.e. no identifying characteristics of participants will be published or presented.

G. QC protocol

Downloaded From: http://jamanetwork.com/pdfaccess.ashx?url=/data/journals/jama/934885/ by a Harvard University User on 03/30/2017

To ensure uniform data collection and reporting procedures, training and certification of study center research assistants (RAs) will occur at the DCC. Additionally, there will be a weekly conference call between the DCC deputy data manager and the site coordinators at each center to globally address any screening or data collection issues and maintain study-wide consistency in procedures. Monthly conference calls will be conducted between the DCC PI and all site investigators to check progress and data integrity. Patient recruitment and follow-up, as well as data collection and archiving procedures, will be monitored by the DCC with bi-annual site visits. 20% of the patient questionnaires and lab forms will be randomly sampled and queried against stored values in the DCC database and archives.

To verify the accuracy of 25(OH)D measurements obtained by the Channing Laboratory, 20% of the maternal blood samples, as well as cord blood samples at delivery, will also be sent to Dr. Hollis' lab. Any discrepant lab values will be checked to determine the source of the variation. If appropriate, a replicate assay will be conducted. Samples will also be obtained from each lot of the vitamin D supplement (4000 IU vitamin D) and prenatal vitamins (400 IU vitamin D) and sent to the lab of Dr. Hollis for analysis to ensure the level of Vitamin D compound in each is with the range specified in trial protocol.

The DCC will provide monthly reports on the timeliness, quality and completeness of the data to the Steering Committee, organized by type of visit, e.g. screening visit 1, randomization day, 6-month visit, etc, and by specific data form. The Steering Committee will remain informed of any potential screening or data collection issues (with patient forms or lab work) and be able to recommend protocol changes or study center intervention as necessary to resolve any outstanding concerns.

H. Blinding

Until the end of the trial, all investigators, clinical staff and participants are masked to trial outcome data, with the exception of the trial statisticians, and the data manager. Due to the nature of the intervention, laboratory technicians and staff obtaining calcium/creatinine ratios may be able to infer treatment group assignment. However, there are other more common causes of elevated calcium/creatinine ratios (i.e. dehydration). Therefore, we do not anticipate any issues with unintentional unblinding of study investigators or clinical staff. There may be a rare case where emergency unblinding may be necessary (e.g. overdose of study pills or persistent elevation of urinary Ca/Cr ratio with hypercalcemia and hypervitaminosis D). In this case, emergency unblinding will occur at the discretion of the DCC and appropriate actions will be taken, including immediate reporting to the DSMB. In cases where non-emergent unblinding may be necessary, The DCC PIs will seek input from the DSMB prior to unblinding.

For quality control purposes, 20% of the blood samples will also be sent to Dr. Hollis' lab for 25 (OH) D measurements. However, we will adhere to established procedures to maintain separation between laboratory staff and clinical staff that have patient contact. For quality assurance, Dr. Hollis will receive a report of measured 25(OH)D levels that are stripped of all identifying patient information (identifier and clinical information).

To ensure rapid intervention in the unexpected event of out-of-range ($\geq$1.55 mmol/mmol) calcium/creatinine ratios, urinary calcium and creatinine measurement will be conducted at each Clinical Center's Clinical Chemistry Laboratory. Again, we will adhere to established procedures to maintain separation between laboratory staff and clinical staff that have patient contact or are responsible for measuring primary or secondary endpoint outcomes. In the event of an out-of-range calcium/creatinine ratio, the patient will be contacted for follow-up, with the procedure detailed in Section I below.

Given the benign treatment intervention, the manufacturing of prenatal vitamins, placebo and Vitamin D supplement by the same drug company (Tishcon Corp), the distribution of study vitamins by the DCC, off-site assays of 25(OH)D levels, protocols to maintain separation

Downloaded From: http://jamanetwork.com/pdfaccess.ashx?url=/data/journals/jama/934885/ by a Harvard University User on 03/30/2017

between information available to laboratory staff, trial statisticians, the DCC, and site clinical staff and investigators, we anticipate that we will successfully maintain blinding of all investigators, clinical staff and participants to both treatment intervention and trial outcome until the end of the trial.

I. Data Safety and Monitoring of Each Subject

An external Data Safety and Monitoring Board (DSMB), composed of vitamin D metabolism experts, early life asthma experts, obstetricians, an ethicist, and a statistician has been appointed by the NHLBI. The board will be convened prior to study initiation to review the protocol and to examine and comment upon "shell" reports that will be used to illustrate data accumulation as the study proceeds.

All study subjects will be monitored for hypervitaminosis D, the only known toxicity of which is hypercalcemia. Operationally, we will define caution by urinary calcium/creatinine ratio ≥ 1.55 mmol/mmol from the samples that are obtained monthly starting 1 month after randomization. Whenever any patient exceeds the caution limit, the study medication will be temporarily discontinued and a specific case study will be initiated. The first elevated urine Ca:Cr ratio will trigger the repeat measure of the urine calcium/creatinine ratio after hydration (drinking at least 8 oz of water prior to coming in for the visit). If the repeat urinary calcium/creatinine after hydration remains elevated, the patient will have serum calcium and vitamin D levels checked at the Clinical Center's Clinical Chemistry Laboratory. If the serum calcium and vitamin D levels are within normal limits, the subject will resume study medication. Predefined cutoff of a calcium of ≥10.2 mg/dl or vitamin D level of ≥ 300nmol/L are criteria for removal from the study treatment. The subject will still be followed in the intention-to-treat paradigm. Further evaluation (including the possibility of taking additional calcium or vitamin D supplements as well as workup for other disorders such as hyperparathyroidism) will be pursued further depending on the serum calcium and vitamin D levels as appropriate. The appropriate IRB will be notified, and the NIH and FDA will be notified per their protocols. The DSMB chair will be notified, as with any severe adverse event. After the detailed case review has been performed by the clinical center to ascertain the cause of the hypervitaminosis D in the subject and reviewed by the DSMB to make an informed decision about the study's continuation. If the elevated levels in the subject are attributed to the vitamin D supplement, the subject will be withdrawn from supplementation but will continue to be monitored.

Subjects who have a subsequent elevated urine Ca:Cr that is a lower ratio than the initial measurement will remain on the study medication and no further action will be required. If the ratio of any subsequent spot urine is greater than the initial ratio, the woman will be asked to stop study medication, and be reminded to properly hydrate. She will be asked to return to the clinical center where blood will be drawn to determine serum Ca and 25(OH)D levels, no repeat urine is required at this time. If the levels of both are within the normal range, the subject will resume study medication and continue to be monitored per protocol each month (with any other elevated ratio again being compared to the initial elevated ratio). If either of the serum values are outside of the normal range, the procedure outlined above will be followed.

J. Adverse Events and Reporting to the DSMB

- Serious Adverse Events (SAEs) will include the following:
  - Symptomatic hypercalcemia and/or elevated 25(OH)D level (as defined above)
  - Pre-eclampsia and Eclampsia
  - Any hospitalization of mother (including severe pregnancy complications such as HELLP [Hemolytic anemia, Elevated Liver enzymes, Low Platelet count] syndrome, deep vein thrombosis or coagulopathy), except for
    - Delivery after 32 weeks

Downloaded From: http://jamanetwork.com/pdfaccess.ashx?url=/data/journals/jama/934885/ by a Harvard University User on 03/30/2017

        - Pre-term labor not resulting in delivery
    - Any newly diagnosed serious maternal illness
    - Maternal death from any cause
    - Pre-term delivery <32 weeks gestation
    - Discovery of major fetal anomalies or diagnosed major congenital anomalies (note: an elective abortion in the absence of diagnosed congenital anomalies will be considered a study withdrawal, not an SAE)
    - Still birth/Intrauterine fetal death
    - Neonatal demise
    - Any neonatal intensive care admission
- Adverse Events (AEs) will include (but not limited to):
    - Pre term delivery between 32 and 37 weeks
    - Gestational diabetes
    - Gestational hypertension
    - Perinatal infections
    - Anemia
    - Prolonged rupture of the membranes
    - Placental abnormalities such as previa or marginal abruption
    - Meconium staining
    - Abnormal labor
    - Low birth weight (<1500g)
    - Asymptomatic hypercalcemia (≥10.2mg/dl)
    - Hypercalciuria
        - Urine calcium/creatinine ratio ≥1 after repeat measure. If ratio normalizes after hydration this is NOT an adverse event

The occurrence of adverse events (AE) and serious adverse events (SAE) will be monitored by each Clinical Center through the monthly maternal health questionnaire and the monthly OB medical record review. Additionally, hypercalciuria will be monitored with the monthly urinary calcium excretion. Monitoring of these urine samples are detailed above. If a SAE is discovered, the Clinical Center staff, under the direction of the Clinical Center PI, will fill out the SAE form and determine whether this event was likely to have been related to the study medication. The form will then be uploaded to the secure VDAART website and an email will be automatically generated to alert the DCC co-PIs and staff. The DCC staff will then review the form for any errors, missing or incomplete information, or any inconsistencies. The form will then be uploaded within to the secure DSMB page on the website after approval by one of the DCC medical staff members (Dr. Litonjua or Dr. Weiss). The DCC is expected to complete this review and approval, and upload of the SAE form to the DSMB page of the secure website within 72 hours of being notified by the Clinical Center. Once the SAE form has been uploaded, an email from Dr. Litonjua and Dr. Weiss will be generated notifying the DSMB Chair and the NHLBI staff. The DSMB Chair will then review the form and involve other DSMB members as he deems appropriate. The DSMB Chair will then communicate with the DCC Staff by email regarding the outcome of the review, including instructions for the DCC to report the SAE to the FDA and the Clinical Center IRBs, if found to be likely related to the study medication. Otherwise, the DCC will prepare annual reports of all SAEs and AEs to the FDA and the IRB at each center.

AEs will also be monitored via the above mechanism. Once an AE is identified, the appropriate form will be completed. These forms will be uploaded to the secure VDAART website as soon as they are completed. The DCC staff will review these forms and prepare quarterly reports to the DSMB. These quarterly reports will be uploaded to the DSMB page of the secure website and an email will be generated notifying all DSMB members and NHLBI staff. All adverse events

Downloaded From: http://jamanetwork.com/pdfaccess.ashx?url=/data/journals/jama/934885/ by a Harvard University User on 03/30/2017

will be reviewed by the entire DSMB at quarterly intervals, by e-mail, in between the regularly scheduled semi-annual meetings. SAEs and AEs will be reported by masked treatment groups. In addition, all SAEs and AEs will be reported by ethnicity and gender (for events among the infants), and with information about the expected rate of occurrence of these events in a general population of pregnant women (or infants). The Director of NHLBI, with advice from the DCC and the DSMB, will have the sole power to stop the study in the event of an unanticipated statistically and clinically significant difference between arms in rates of serious adverse events, whether or not these are attributable to the study treatment. The definition of statistical significance for study stopping will be adjusted alpha = 0.01.

K. Interim Analyses

For each specific aim, we have developed an interim analysis plan to monitor the endpoints over the course of the study. For specific aims 1 and 3, which monitor primary (asthma and recurrent wheeze) and secondary (e.g., eczema, LRI) outcomes in the infants, this sequential monitoring plan consists of three interim analyses (conducted at year 3 month 5, year 4 month 5, and year 5 month 5 and a final analysis at the end of the study, when the three-year follow-up has been completed on all subjects. For specific aims 2 and 4, which involve outcomes monitored at delivery (i.e., circulating 25(OH)D levels in maternal and cord blood), there will be two interim looks and a final analysis (year 2 month 5, year 3 month 5, and year 4 month 5, respectively).

For maximum flexibility in the analysis, we will use a Lan-Demets alpha spending function[98] with O'Brien-Fleming bounds. The software program LANDEM (http://www.biostat.wisc.edu/landemets/)[97] was used to calculate the crossing boundaries, which, for the first and third specific aims are $|z|=4.3329$, 2.9631, 2.3590, and 2.0141, at the first, second, third and final analyses, respectively. For the second and fourth specific aim, with 3 looks in total, the crossing boundaries are $|z|=3.7103$, 2.5114, and 1.993 at the first, second looks and final analysis, respectively. While the looks in the interim analyses have been predetermined, the Lan-DeMets procedure provides a spending function approach which does not require pre-specification of the frequency or timing of interim looks. This can be advantageous if the timing of data collection varies of if the need for more frequent monitoring arises. Conservative O'Brien-Fleming bounds are used to preserve most of the type-I error for the final analysis. For all specific aims, the hypothesis tests are conducted with two-sided alternatives (i.e. a two-sided p-value). If the boundaries are crossed for efficacy endpoints at any interim analyses, for any of the aims, the study will continue. If the boundaries are crossed in Specific Aims 2-4, the primary specific aim, whether Vitamin D supplementation in pregnancy is association with a reduction in asthma and/or recurrent wheeze risk, still needs to be assessed, thus the study should continue to monitor this endpoint. If the boundaries are crossed during an interim analysis of the primary aim, the study will also be continued, as the target intervention period (i.e., pregnancy) will have passed for all recruited subjects, and will feel that it is most important to examine the impact of this intervention on the children at 3 years of age. If the study is stopped early due to the success of the intervention, important clinical and outcome information may be lost. Therefore, we feel that it is important to continue the study even if interim analysis boundaries are crossed. The only situation when consideration will be given to prematurely stopping the trial will be for safety considerations. It is inevitable that adverse health events will be observed in all contexts noted above even if both treatments have no biological effects whatsoever. If the rate of adverse events on the active arm substantially and persistently exceeds the rate on the control arm, even in the absence of biological theory that connects the adverse events to vitamin D exposures, and no evidence of therapeutic effect or benefit of the active treatment is apparent in interim analyses, then premature termination of the study may be considered on safety grounds. The DSMB will be asked to articulate

Downloaded From: http://jamanetwork.com/pdfaccess.ashx?url=/data/journals/jama/934885/ by a Harvard University User on 03/30/2017

reasonable definitions of `substantial and persistent excess' of adverse event rate if this situation emerges.

An IOM committee has been named to undertake a study to assess current relevant data and update as appropriate the Daily Reference Intakes (DRIs) for vitamin D and calcium. It is likely that the report from this committee (expected to be released in May, 2010 – 10 months into the recruitment phase; http://iom.edu/?ID=61170) will recommend a higher DRI for vitamin D (currently 200 IU/day for women of child-bearing age). This is one other concern regarding a potential cause for early study termination. If the IOM recommends Vitamin D supplementation of up to 2,000 IU/day during the course of the study, and if this increase in recommended supplementation occurs early in the course of the study (i.e., while the study is still in the antenatal monitoring phase), the likely target supplementation recommendation (≤2,000 IU/day) is well below the supplementation level indicated for the treatment group in this study. Even if the 2,000 IU recommendation results in additional supplementation occurring in the placebo arm, there should still be a measurable difference in outcomes, with regard to maternal vitamin D levels. Dr. Hollis' previous trial had shown a statistically significant difference in 25(OH)D levels between the 2,400 IU/day and the 4,400 IU/day doses (Figure 1 in the VDAART Protocol). 25(OH)D levels will be available in the mothers to assess the impact of the IOM recommendation on the study outcomes. Consideration of increasing the dose in the intervention arm will be considered, after a meeting with the VDAART Trial Steering Committee, the NHLBI staff, and the DSMB. If an IOM recommended increase in supplementation occurs after the antenatal supplementation period, this will have little impact on any study outcome, as the crucial window of Vitamin D supplementation for the prevention of asthma occurs in the antenatal period. In the unlikely event of an IOM recommended increase in supplementation >2,000 IU/day, a meeting between the VDAART Steering Committee, the NHLBI Staff, and the DSMB members will be convened to discuss potential alternative plans, including early termination of the trial.

L. Study Timeline

**Year 1**	Months 0-4	Planning and preparation<br>- Training of RAs<br>- Preparation of Study medications<br>- Printing of questionnaires
	Month 6	Screening and recruitment begins<br>Randomization and intervention
	Month 12	First group of mothers deliver
**Year 2**	Month 3	First group of quarterly calls for follow-up of infants begins
	Month 6	Data clean up, interim analysis of SA2 and SA4
	Month 11	Last group of mothers screened, recruited, and randomized
	Month 12	First group of in-person visits for 1-yr old infants
**Year 3**	Month 6	Last group of mothers deliver<br>Data clean up, interim analysis of all specific aims
	Months 6-12	Quarterly follow-up calls and yearly in-person visits continue
**Year 4**	Month 1-5	Quarterly follow-up calls and yearly in-person visits continue . Data clean up, interim analysis of all SA1/SA3, final analysis of SA2/SA4
	Months 6-12	Quarterly follow-up calls and yearly in-person visits continue
**Year 5**	Months 1-5	Quarterly follow-up calls and yearly in-person visits continue
	Month 5	First group of in-person visits for 3-yr old children

Downloaded From: http://jamanetwork.com/pdfaccess.ashx?url=/data/journals/jama/934885/ by a Harvard University User on 03/30/2017

	Months 5-12	Data clean up, interim analysis of all SA1/SA3<br>Quarterly follow-up calls and yearly in-person visits continue
**No Cost Extension**	Month 4<br>Months 5-10	Finish in-person visits for last group of 3-yr old children<br>Finish data clean-up and statistical analyses<br>Manuscript preparation and presentation of findings

VIII. HUMAN SUBJECTS RESEARCH

Protection of Human Subjects

Risks to the Subjects

Human Subjects Involvement and Characteristics: For this project, we will be conducting a clinical trial of “high dose” vitamin D supplementation vs. “usual dose” prenatal vitamins in pregnant women for the prevention of asthma and allergies in the women’s offspring at 3 years of age. We propose to randomize 870 pregnant women during their 1st trimester with 435 women to the “high dose” arm and 435 women to the usual care arm, follow them through their pregnancy and delivery, and follow the women and their offspring until the 3rd birthday of the children, with the expectation that we will evaluate at least 660 three year olds. A brief study description and a copy of the consent form will be given to pregnant women receiving their care at 3 Clinical Centers (Boston, St. Louis, and San Diego) at their first prenatal visit. Women interested in the study will be invited to a separate visit for more detailed explanation and providing informed consent. There are several reasons for this separate visit: 1) allow the pregnant women to review the informed consent, and 2) to assure as much as possible the dedication of the women to participation in the study until the 3rd birthday of their child. Pregnant women will be selected on the basis of an age >18 years but <40 years, ability to speak English or Spanish, intention to participate in the trial for the full 4 years, no pre-existing type 1 or type 2 diabetes mellitus, hypertension, parathyroid or uncontrolled thyroid disease, kidney stones, and sarcoidosis. Written informed consent (in English or Spanish, according to individual preferences) will be obtained from each subject by study coordinators at each Clinical Center prior to voluntary participation. Participating women will be followed during the remainder of their pregnancy, and their newborns will be followed after delivery.

Sources of Research Material: Data from questionnaires, maternal (labor and delivery) and neonatal medical records will be used for research purposes only. Peripheral blood will be obtained from the mothers at a prenatal visit, at 32-38 weeks gestation, and at 1 yr after delivery for measurement of vitamin D levels. Cord blood will be obtained from the research subjects to be used by the investigators for measurement of vitamin D levels and storage of white cell pellets for future genetic studies. Peripheral blood will be obtained from the children to be used by the investigators for measurement of vitamin D levels at 1 yr and 3yrs and total and allergen-specific IgE in the child at 3 yrs. These blood samples will be used by the investigators for research purposes only; the results will be kept strictly confidential. With parental permission, results of measurements of serum total and allergen-specific IgE at age 3 years will be sent to the child’s primary care physician.

The major risk involved with vitamin D toxicity is hypercalcemia, which can be detected by measurement in serum or by calcium excretion in urine. Based on Dr. Hollis’ extensive review of the literature and his experience with his current vitamin D supplementation in pregnancy trial, vitamin D supplementation of 4,400 IU/day throughout pregnancy is safe. Nevertheless, we will be monitoring urine calcium-to-creatinine ratios as a measure to calcium excretion in the urine each time the mothers come for their prenatal visits. This method of monitoring was chosen because it is reliable and does not require phlebotomy.

Downloaded From: http://jamanetwork.com/pdfaccess.ashx?url=/data/journals/jama/934885/ by a Harvard University User on 03/30/2017

Another risk of participation in this study is the social-psychological risk resulting from inadvertent disclosure of medical history information. This potential risk is guarded against by storage of completed questionnaires in a locked filing system and by labeling other phenotypic/genotypic data using ID numbers only. In certain instances, the funding agency for this grant may require posting of data for sharing with outside researchers. In these instances, these researchers will be asked to sign a statement of confidentiality before being allowed access to the data. In addition, no personal identifiers will be included in datasets that will be shared with outside researchers.

The only potential risks of blood drawing are hematoma and infection at the skin site, minimal pain of venous puncture, uncommonly fainting reactions, and additionally discomfort and crying from the child.

Adequacy of Protection against Risks

Recruitment of Subjects and Informed Consent: Subjects will be recruited voluntarily from those meeting entry criteria. Written consent will be obtained from participating women before randomization, collection of questionnaire data, extraction of information from maternal (labor and delivery) and neonatal records, and samples of cord blood and peripheral blood. Subjects are under no obligation to participate. All signed consent forms are copied times three, one placed in the patient's research file and a second in a common consent form folder for the study and a copy given to the patient. The consenting process is an ongoing process and participants will be informed of important new developments that relate importantly to the safety of the study. In addition at each follow-up visit study procedures and requirements are reviewed.

Protection against Risks: To monitor for the rare potential for Vitamin D toxicity urine samples will be evaluated for Calcium/Creatinine at each clinical prenatal visit and for vitamin D levels at the blood samples collected during pregnancy. Should levels of Calcium/Creatinine exceed 1.55 mmol/mmol or Vitamin D levels exceed 300 nmol/L, a specific case study will be initiated to examine the contribution of confounding factors (e.g. diet, sunlight exposure, other over the counter pills, etc.). The appropriate IRB will be notified, and the NIH and FDA will be notified per their protocols. If the elevated levels in the subject are attributed to the vitamin D supplement, the subject will be withdrawn from supplementation but will continue to be monitored. As noted above, steps will be taken to ensure that study information remains confidential. As we have mentioned in the background section, there have been no reported adverse effects on the fetus due to vitamin D supplementation. By monitoring the urinary calcium/creatinine ratio in the pregnant mothers and ensuring that the vitamin D levels do not exceed 300 nmol/L, the risks to the fetus will be minimized. The discomforts associated with blood drawing are minimal. Phlebotomy will be performed by licensed phlebotomists or research assistants that are adequately trained for blood drawing in infants. For maternal samples, we will minimize blood draws by piggybacking the collection at delivery with the blood draw for clinical purposes.

Potential Benefit to Individual Subjects and Society: For the participating mothers, a direct benefit will be close monitoring, and thus better adherence, of prenatal vitamin intake, which has already established benefits for fetal wellbeing. Additionally, even for those women in the placebo group, they will be receiving 400 IU of vitamin D per day in the prenatal vitamin that the study will be supplying at no charge. This 400 IU dose is twice that of the current AI recommendations of the IOM. The prenatal vitamins will be provided free of charge to all participants in the trial. The benefits of vitamin D on several outcomes related to bone health are well-documented[99].

For infants in the trial, a potential benefit is the close monitoring for the development of asthma and allergies. Additionally, we will offer 400 IU/day of vitamin D to all the infants who consume less than 1000 ml of formula per day, according to the recommendations of the American Academy of Pediatrics. This includes all children who are breastfed, either partially or exclusively and all formula-fed infants until they consume at least 1000 ml of formula per day. These vitamin D supplements will be provided free of charge to the infants in the trial.

Downloaded From: http://jamanetwork.com/pdfaccess.ashx?url=/data/journals/jama/934885/ by a Harvard University User on 03/30/2017

The potential benefit to society is significant if the trial shows a decrease in the prevalence of asthma, wheezing illnesses, and allergies at 3 years of age. Allergic diseases such as asthma are a major public health problem in the U.S. Since the risks involved in the protocol are small and the societal benefits are potentially large, the risk-benefit ratio is strongly on the side of benefit.

Importance of the Knowledge to Be Gained:

As previously stated, the main potential benefit of this research proposal is that it may lead to public health interventions that could lead to a decrease of up to 50% of the asthma prevalence worldwide, and may contribute to our understanding of the pathogenesis of allergic diseases such as asthma. While there are individual risks involved in this project, we will institute mechanisms to minimize these risks. Thus, because of the potentially great societal benefit, the individual risks are reasonable.

Downloaded From: http://jamanetwork.com/pdfaccess.ashx?url=/data/journals/jama/934885/ by a Harvard University User on 03/30/2017

References

1. Yunginger JW, Reed CE, O'Connell EJ, Melton LJ, 3rd, O'Fallon WM, Silverstein MD. A community-based study of the epidemiology of asthma. Incidence rates, 1964-1983. Am Rev Respir Dis 1992;146:888-94.
2. King ME, Mannino DM, Holguin F. Risk factors for asthma incidence. A review of recent prospective evidence. Panminerva Med 2004;46:97-110.
3. Mannino DM, Homa DM, Akinbami LJ, Moorman JE, Gwynn C, Redd SC. Surveillance for asthma--United States, 1980-1999. MMWR Surveill Summ 2002;51:1-13.
4. CDC. Asthma prevalence, health care use, and mortality, 2002. . Hyattsville, MD: US Department of Health and Human Services, CDC, National Center for Health Statistics; 2004.
5. Smith DH, Malone DC, Lawson KA, Okamoto LJ, Battista C, Saunders WB. A national estimate of the economic costs of asthma. Am J Respir Crit Care Med 1997;156:787-93.
6. Adams PF, Hendershoot GE, Marano MA. Current Estimates from the National Health Interview Survey, 1996. Hyattsville, MD: National Center for Health Statistics 1999;Vital Health Stat Ser 10.
7. Holick MF. High prevalence of vitamin D inadequacy and implications for health. Mayo Clin Proc 2006;81:353-73.
8. Nesby-O'Dell S, Scanlon KS, Cogswell ME, et al. Hypovitaminosis D prevalence and determinants among African American and white women of reproductive age: third National Health and Nutrition Examination Survey, 1988-1994. Am J Clin Nutr 2002;76:187-92.
9. US Environmental Protection Agency. Report to Congress on indoor air quality, volume II: assessment and control of indoor air pollution, Report No. EPA 400-1-89-001C. Washington, DC: EPA; 1989.
10. Hollis BW, Wagner CL. Assessment of dietary vitamin D requirements during pregnancy and lactation. Am J Clin Nutr 2004;79:717-26.
11. Hollis BW, Wagner CL. Vitamin D deficiency during pregnancy: an ongoing epidemic. Am J Clin Nutr 2006;84:273.
12. Hollis BW, Wagner CL. Nutritional vitamin D status during pregnancy: reasons for concern. Cmaj 2006;174:1287-90.
13. Sanchez PA, Idrisa A, Bobzom DN, et al. Calcium and vitamin D status of pregnant teenagers in Maiduguri, Nigeria. J Natl Med Assoc 1997;89:805-11.
14. Taylor SN, Wagner CL, Hulsey TC, Ebeling M, Hollis BW. Bone mineralization correlates with vitamin D status in the vitamin D insufficient range in newborns. In: ASBMR Scientific Meeting - Contemporary Diagnosis and Treatment of Vitamin D Related Disorders; December 4-5, 2006; Arlington, VA; December 4-5, 2006.
15. Lee JM, Smith JR, Philipp BL, Chen TC, Mathieu J, Holick MF. Vitamin d deficiency in a healthy group of mothers and newborn infants. Clin Pediatr (Phila) 2007;46:42-4.
16. Bassir M, Laborie S, Lapillonne A, Claris O, Chappuis MC, Salle BL. Vitamin D deficiency in Iranian mothers and their neonates: a pilot study. Acta Paediatr 2001;90:577-9.
17. Sachan A, Gupta R, Das V, Agarwal A, Awasthi PK, Bhatia V. High prevalence of vitamin D deficiency among pregnant women and their newborns in northern India. Am J Clin Nutr 2005;81:1060-4.
18. Judkins A, Eagleton C. Vitamin D deficiency in pregnant New Zealand women. N Z Med J 2006;119:U2144.
19. van der Meer IM, Karamali NS, Boeke AJ, et al. High prevalence of vitamin D deficiency in pregnant non-Western women in The Hague, Netherlands. Am J Clin Nutr 2006;84:350-3; quiz 468-9.
20. Nguyen M, Guillozo H, Garabedian M, Balsan S. Lung as a possible additional target organ for vitamin D during fetal life in the rat. Biol Neonate 1987;52:232-40.

Downloaded From: http://jamanetwork.com/pdfaccess.ashx?url=/data/journals/jama/934885/ by a Harvard University User on 03/30/2017

21. Nguyen TM, Guillozo H, Marin L, Tordet C, Koite S, Garabedian M. Evidence for a vitamin D paracrine system regulating maturation of developing rat lung epithelium. Am J Physiol 1996;271:L392-9.
22. Marin L, Dufour ME, Nguyen TM, Tordet C, Garabedian M. Maturational changes induced by 1 alpha,25-dihydroxyvitamin D3 in type II cells from fetal rat lung explants. Am J Physiol 1993;265:L45-52.
23. Marin L, Dufour ME, Tordet C, Nguyen M. 1,25(OH)2D3 stimulates phospholipid biosynthesis and surfactant release in fetal rat lung explants. Biol Neonate 1990;57:257-60.
24. Nguyen M, Trubert CL, Rizk-Rabin M, et al. 1,25-Dihydroxyvitamin D3 and fetal lung maturation: immunogold detection of VDR expression in pneumocytes type II cells and effect on fructose 1,6 bisphosphatase. J Steroid Biochem Mol Biol 2004;89-90:93-7.
25. Glasgow JF, Thomas PS. Rachitic respiratory distress in small preterm infants. Arch Dis Child 1977;52:268-73.
26. Rehan VK, Torday JS, Peleg S, et al. 1Alpha,25-dihydroxy-3-epi-vitamin D3, a natural metabolite of 1alpha,25-dihydroxy vitamin D3: production and biological activity studies in pulmonary alveolar type II cells. Mol Genet Metab 2002;76:46-56.
27. Phokela SS, Peleg S, Moya FR, Alcorn JL. Regulation of human pulmonary surfactant protein gene expression by 1alpha,25-dihydroxyvitamin D3. Am J Physiol Lung Cell Mol Physiol 2005;289:L617-26.
28. Gaultier C, Harf A, Balmain N, Cuisinier-Gleizes P, Mathieu H. Lung mechanics in rachitic rats. Am Rev Respir Dis 1984;130:1108-10.
29. Brun P, Dupret JM, Perret C, Thomasset M, Mathieu H. Vitamin D-dependent calcium-binding proteins (CaBPs) in human fetuses: comparative distribution of 9K CaBP mRNA and 28K CaBP during development. Pediatr Res 1987;21:362-7.
30. Lunghi B, Meacci E, Stio M, et al. 1,25-dihydroxyvitamin D3 inhibits proliferation of IMR-90 human fibroblasts and stimulates pyruvate kinase activity in confluent-phase cells. Mol Cell Endocrinol 1995;115:141-8.
31. Stio M, Celli A, Lunghi B, Raugei G, Modesti A, Treves C. Vitamin D receptor in IMR-90 human fibroblasts and antiproliferative effect of 1,25-dihydroxyvitamin D3. Biochem Mol Biol Int 1997;43:1173-81.
32. Stio M, Lunghi B, Celli A, Nassi P, Treves C. Effect of 1,25-dihydroxyvitamin D3 on proliferation in senescent IMR-90 human fibroblasts. Mech Ageing Dev 1996;91:23-36.
33. Camargo J, C.A., Rifas-Shiman SL, Litonjua AA, et al. Maternal intake of vitamin D during pregnancy and risk of recurrent wheeze in children at age 3 years. Am J Clin Nutr 2007;85:788-95.
34. Devereux G, Litonjua AA, Turner S, et al. Maternal vitamin D intake during pregnancy and early childhood wheezing. Am J Clin Nutr 2007;85:853-9.
35. Whitelaw A, Parkin J. Development of immunity. Br Med Bull 1988;44:1037-51.
36. Dickson I. New approaches to vitamin D. Nature 1987;325:18.
37. Minghetti PP, Norman AW. 1,25(OH)2-vitamin D3 receptors: gene regulation and genetic circuitry. Faseb J 1988;2:3043-53.
38. Akeno N, Saikatsu S, Kawane T, Horiuchi N. Mouse vitamin D-24-hydroxylase: molecular cloning, tissue distribution, and transcriptional regulation by 1alpha,25-dihydroxyvitamin D3. Endocrinology 1997;138:2233-40.
39. Mahon BD, Wittke A, Weaver V, Cantorna MT. The targets of vitamin D depend on the differentiation and activation status of CD4 positive T cells. J Cell Biochem 2003;89:922-32.
40. Heine G, Anton K, Henz BM, Worm M. 1alpha,25-dihydroxyvitamin D3 inhibits anti-CD40 plus IL-4-mediated IgE production in vitro. Eur J Immunol 2002;32:3395-404.
41. Adorini L, Penna G, Giarratana N, et al. Dendritic cells as key targets for immunomodulation by Vitamin D receptor ligands. J Steroid Biochem Mol Biol 2004;89-90:437-41.

Downloaded From: http://jamanetwork.com/pdfaccess.ashx?url=/data/journals/jama/934885/ by a Harvard University User on 03/30/2017

42. Cantorna MT, Zhu Y, Froicu M, Wittke A. Vitamin D status, 1,25-dihydroxyvitamin D3, and the immune system. Am J Clin Nutr 2004;80:1717S-20S.
43. Matheu V, Back O, Mondoc E, Issazadeh-Navikas S. Dual effects of vitamin D-induced alteration of TH1/TH2 cytokine expression: enhancing IgE production and decreasing airway eosinophilia in murine allergic airway disease. J Allergy Clin Immunol 2003;112:585-92.
44. Iho S, Kura F, Sugiyama H, Takahashi T, Hoshino T. The role of monocytes in the suppression of PHA-induced proliferation and IL 2 production of human mononuclear cells by 1,25-dihydroxyvitamin D3. Immunol Lett 1985;11:331-6.
45. Reichel H, Koeffler HP, Tobler A, Norman AW. 1 alpha,25-Dihydroxyvitamin D3 inhibits gamma-interferon synthesis by normal human peripheral blood lymphocytes. Proc Natl Acad Sci U S A 1987;84:3385-9.
46. Boonstra A, Barrat FJ, Crain C, Heath VL, Savelkoul HF, O'Garra A. 1alpha,25-Dihydroxyvitamin d3 has a direct effect on naive CD4(+) T cells to enhance the development of Th2 cells. J Immunol 2001;167:4974-80.
47. Cantorna MT, Woodward WD, Hayes CE, DeLuca HF. 1,25-dihydroxyvitamin D3 is a positive regulator for the two anti-encephalitogenic cytokines TGF-beta 1 and IL-4. J Immunol 1998;160:5314-9.
48. Evans KN, Nguyen L, Chan J, et al. Effects of 25-hydroxyvitamin D3 and 1,25-dihydroxyvitamin D3 on cytokine production by human decidual cells. Biol Reprod 2006;75:816-22.
49. Pichler J, Gerstmayr M, Szepfalusi Z, Urbanek R, Peterlik M, Willheim M. 1 alpha,25(OH)2D3 inhibits not only Th1 but also Th2 differentiation in human cord blood T cells. Pediatr Res 2002;52:12-8.
50. Annesi-Maesano I. Perinatal events, vitamin D, and the development of allergy. Pediatr Res 2002;52:3-5.
51. Brehm JM, Celedon JC, Soto-Quiros ME, et al. Serum Vitamin D Levels and Markers of Severity of Childhood Asthma in Costa Rica. Am J Respir Crit Care Med 2009.
52. Hypponen E, Berry DJ, Wjst M, Power C. Serum 25-hydroxyvitamin D and IgE - a significant but nonlinear relationship. Allergy 2009;64:613-20.
53. Byremo G, Rod G, Carlsen KH. Effect of climatic change in children with atopic eczema. Allergy 2006;61:1403-10.
54. Sidbury R, Sullivan AF, Thadhani RI, Camargo CA, Jr. Randomized controlled trial of vitamin D supplementation for winter-related atopic dermatitis in Boston: a pilot study. Br J Dermatol 2008;159:245-7.
55. Gregori S, Casorati M, Amuchastegui S, Smiroldo S, Davalli AM, Adorini L. Regulatory T cells induced by 1 alpha,25-dihydroxyvitamin D3 and mycophenolate mofetil treatment mediate transplantation tolerance. J Immunol 2001;167:1945-53.
56. Gregori S, Giarratana N, Smiroldo S, Uskokovic M, Adorini L. A 1alpha,25-dihydroxyvitamin D(3) analog enhances regulatory T-cells and arrests autoimmune diabetes in NOD mice. Diabetes 2002;51:1367-74.
57. Meehan MA, Kerman RH, Lemire JM. 1,25-Dihydroxyvitamin D3 enhances the generation of nonspecific suppressor cells while inhibiting the induction of cytotoxic cells in a human MLR. Cell Immunol 1992;140:400-9.
58. Schwartz RH. Natural regulatory T cells and self-tolerance. Nat Immunol 2005;6:327-30.
59. Chatenoud L, Salomon B, Bluestone JA. Suppressor T cells--they're back and critical for regulation of autoimmunity! Immunol Rev 2001;182:149-63.
60. Griffin MD, Xing N, Kumar R. Vitamin D and its analogs as regulators of immune activation and antigen presentation. Annu Rev Nutr 2003;23.
61. D'Ambrosio D, Cippitelli M, Cocciolo MG, et al. Inhibition of IL-12 production by 1,25-dihydroxyvitamin D3. Involvement of NF-kappaB downregulation in transcriptional repression of the p40 gene. J Clin Invest 1998;101:252-62.

Downloaded From: http://jamanetwork.com/pdfaccess.ashx?url=/data/journals/jama/934885/ by a Harvard University User on 03/30/2017

62. Xing N, Maldonado ML, Bachman LA, McKean DJ, Kumar R, Griffin MD. Distinctive dendritic cell modulation by vitamin D(3) and glucocorticoid pathways. Biochem Biophys Res Commun 2002;297:645-52.
63. Yu XP, Bellido T, Manolagas SC. Down-regulation of NF-kappa B protein levels in activated human lymphocytes by 1,25-dihydroxyvitamin D3. Proc Natl Acad Sci U S A 1995;92:10990-4. Erratum in: Proc Natl Acad Sci U S A 1996 Jan 9;93(1):524. .
64. Bosse Y, Maghni K, Hudson TJ. 1{alpha},25-DIHYDROXY-VITAMIN D3 STIMULATION OF BRONCHIAL SMOOTH MUSCLE CELLS INDUCES AUTOCRINE, CONTRACTILITY AND REMODELLING PROCESSES. Physiol Genomics 2007 (In Press).
65. Cippitelli M, Santoni A. Vitamin D3: a transcriptional modulator of the interferon-gamma gene. Eur J Immunol 1998;28:3017-30.
66. Lehtonen-Veromaa M, Mottonen T, Irjala K, et al. Vitamin D intake is low and hypovitaminosis D common in healthy 9- to 15-year-old Finnish girls. Eur J Clin Nutr 1999;53:746-51.
67. Vieth R, Cole DE, Hawker GA, Trang HM, Rubin LA. Wintertime vitamin D insufficiency is common in young Canadian women, and their vitamin D intake does not prevent it. Eur J Clin Nutr 2001;55:1091-7.
68. Datta S, Alfaham M, Davies DP, et al. Vitamin D deficiency in pregnant women from a non-European ethnic minority population--an interventional study. Bjog 2002;109:905-8.
69. Talwar SA, Aloia JF, Pollack S, Yeh JK. Dose response to vitamin D supplementation among postmenopausal African American women. Am J Clin Nutr 2007;86:1657-62.
70. Hollis BW. Circulating 25-hydroxyvitamin D levels indicative of vitamin D sufficiency: implications for establishing a new effective dietary intake recommendation for vitamin D. J Nutr 2005;135:317-22.
71. Food and Nutrition Board. Institute of Medicine. Dietary reference intakes for calcium, phosphorus, magnesium, vitamin D, and fluoride. Washington, DC: National Academy Press; 1997.
72. Vieth R. Vitamin D supplementation, 25-hydroxyvitamin D concentrations, and safety. Am J Clin Nutr 1999;69:842-56.
73. Hathcock JN, Shao A, Vieth R, Heaney R. Risk assessment for vitamin D. Am J Clin Nutr 2007;85:6-18.
74. Brooke OG, Brown IR, Bone CD, et al. Vitamin D supplements in pregnant Asian women: effects on calcium status and fetal growth. Br Med J 1980;280:751-4.
75. Cockburn F, Belton NR, Purvis RJ, et al. Maternal vitamin D intake and mineral metabolism in mothers and their newborn infants. Br Med J 1980;281:11-4.
76. Delvin EE, Salle BL, Glorieux FH, Adeleine P, David LS. Vitamin D supplementation during pregnancy: effect on neonatal calcium homeostasis. J Pediatr 1986;109:328-34.
77. Mallet E, Gugi B, Brunelle P, Henocq A, Basuyau JP, Lemeur H. Vitamin D supplementation in pregnancy: a controlled trial of two methods. Obstet Gynecol 1986;68:300-4.
78. Ala-Houhala M, Koskinen T, Terho A, Koivula T, Visakorpi J. Maternal compared with infant vitamin D supplementation. Arch Dis Child 1986;61:1159-63.
79. Hollis BW, Wagner CL. Vitamin D requirements during lactation: high-dose maternal supplementation as therapy to prevent hypovitaminosis D for both the mother and the nursing infant. Am J Clin Nutr 2004;80:1752S-8S.
80. Wagner CL, Hulsey TC, Fanning D, Ebeling M, Hollis BW. High-dose vitamin D3 supplementation in a cohort of breastfeeding mothers and their infants: a 6-month follow-up pilot study. Breastfeeding Medicine 2006;1:57-68.
81. Heaney RP, Davies KM, Chen TC, Holick MF, Barger-Lux MJ. Human serum 25-hydroxycholecalciferol response to extended oral dosing with cholecalciferol. Am J Clin Nutr 2003;77:204-10.

Downloaded From: http://jamanetwork.com/pdfaccess.ashx?url=/data/journals/jama/934885/ by a Harvard University User on 03/30/2017

82. Wagner CL, Greer FR. Prevention of rickets and vitamin d deficiency in infants, children, and adolescents. Pediatrics 2008;122:1142-52.
83. Wang X, Chen C, Wang L, Chen D, Guang W, French J. Conception, early pregnancy loss, and time to clinical pregnancy: a population-based prospective study. Fertil Steril 2003;79:577-84.
84. Castro-Rodriguez JA, Holberg CJ, Wright AL, Martinez FD. A clinical index to define risk of asthma in young children with recurrent wheezing. Am J Respir Crit Care Med 2000;162:1403-6.
85. Ly NP, Gold DR, Weiss ST, Celedon JC. Recurrent wheeze in early childhood and asthma among children at risk for atopy. Pediatrics 2006;117:e1132-8.
86. Barbee RA, Halonen M, Lebowitz M, Burrows B. Distribution of IgE in a community population sample: correlations with age, sex, and allergen skin test reactivity. J All Clin Immun 1981;68:106-11.
87. Schatz M, Mosen DM, Kosinski M, et al. Validity of the Asthma Control Test completed at home. Am J Manag Care 2007;13:661-7.
88. Hollis BW, Kamerud JQ, Selvaag SR, Lorenz JD, Napoli JL. Determination of vitamin D status by radioimmunoassay with an 125I-labeled tracer. Clin Chem 1993;39:529-33.
89. Rifas-Shiman SL, Willett WC, Lobb R, Kotch J, Dart C, Gillman MW. PrimeScreen, a brief dietary screening tool: reproducibility and comparability with both a longer food frequency questionnaire and biomarkers. Public Health Nutr 2001;4:249-54.
90. Willett WC, Sampson L, Stampfer MJ, et al. Reproducibility and Validity of a Semiquantitative Food Frequency Questionnaire. Am J Epidemiol 1985;122.
91. US Department of Agriculture ARS. USDA National Nutrient Database for Standard Reference, Release 14, SR14; 2001. In: 2001.
92. Han J, Colditz GA, Hunter DJ. Risk factors for skin cancers: a nested case-control study within the Nurses' Health Study. Int J Epidemiol 2006.
93. Riekert KA, Rand CS. Electronic monitoring of medication adherence: When is high-tech best? J Clin Psychol Med Settings 2002;9:25-34.
94. Sorkness CA, Lemanske RF, Jr., Mauger DT, et al. Long-term comparison of 3 controller regimens for mild-moderate persistent childhood asthma: The Pediatric Asthma Controller Trial. J Allergy Clin Immunol 2007;119:64-72.
95. Szefler SJ, Phillips BR, Martinez FD, et al. Characterization of within-subject responses to fluticasone and montelukast in childhood asthma. J Allergy Clin Immunol 2005;115:233-42.
96. Rand C, Bilderback A, Schiller K, Edelman JM, Hustad CM, Zeiger RS, for The MIAMI Study Research Group. Adherence with montelukast or fluticasone in a long-term clinical trial: results from the Mild Asthma Montelukast Versus Inhaled Corticosteroid (MIAMI) study. . J Allergy Clin Immunol (in Press) 2007.
97. Reboussin DM, DeMets DL, Kim KM, Lan KK. Computations for group sequential boundaries using the Lan-DeMets spending function method. Control Clin Trials 2000;21:190-207.
98. Lan KKG, DeMets DL. Discrete sequential boundaries for clinical trials. Biometrika 1983;70:659-63.
99. Cranney A, Weiler HA, O'Donnell S, Puil L. Summary of evidence-based review on vitamin D efficacy and safety in relation to bone health. Am J Clin Nutr 2008;88:513S-9S.

Downloaded From: http://jamanetwork.com/pdfaccess.ashx?url=/data/journals/jama/934885/ by a Harvard University User on 03/30/2017

Summary of Protocol Changes

Downloaded From: http://jamanetwork.com/pdfaccess.ashx?url=/data/journals/jama/934885/ by a Harvard University User on 03/30/2017

Summary of Changes to VDAART Protocol

**Table of changes to VDAART Protocol**			
Change	Page/Section	Reason for change	Date
1. Change units of 25(OH)D from ng/ml to nmol/L	throughout document	Recommended change by DSMB	8.6.09
2. Added two paragraphs reviewing vitamin D effects on immune system	bottom of page 2 to top of page 3	Recommended change by DSMB	8.6.09
3. Switched order of Aims 1 and 2	pages 2-3	Steering Committee decision; to conform with scientific aims in NIH grant	8.6.09
4. Changed dose of vitamin D3 for intervention arm from 4,000 IU/day to 4,400 IU/day	throughout document	Recommended change by DSMB; to account for the 400 IU contained in prenatal vitamins	8.6.09
5. Defined current smoker	page 7	Recommended change by DSMB	8.6.09
6. Added to exclusion criteria:			8.6.09
a. kidney stones and sarcoidosis	page 8	Steering Committee decision; these conditions increase risk for hypercalcemia and stone formation	8.6.09
b. Patient health questionnaire depression scale > 15	page 8	Steering Committee decision; attempt to avoid enrolling patients with severe depression.	8.6.09
c. Illicit drug use	page 8	Requested by DSMB; also increases risk of noncompliance with study procedures	8.6.09
d. not previously enrolled in this trial for a previous pregnency	page 8	Recommended change by DSMB	8.6.09
e. discovery of any fetal abnormalities	page 8	Recommended change by DSMB	8.6.09
f. PI discretion	page 8	Steering Committee decision; in the event of any other condition that may prohibit compliance with trial medications and procedures	8.6.09
7. Rewrite Section V (Recruitment)	page 8-9	Steering Committee decision; clarifications and added details to screening, recruitment and follow-up procedures	8.6.09
8. Addition of protocol schemata (Figure 2)	page 10	DSMB request to provide summary of protocol process	8.6.09
9. Table 2:	page 11		8.6.09

Downloaded From: http://jamanetwork.com/pdfaccess.ashx?url=/data/journals/jama/934885/ by a Harvard University User on 03/30/2017

a. remove 6-8 week post-enrollment visit		Steering Committee decisions to streamline visits and decrease participant burden	8.6.09
b. remove maternal blood at delivery		Steering Committee decisions to streamline visits and decrease participant burden	8.6.09
c. 28-32 week visit changed to 32-38 week third trimester visit		Steering Committee decisions to streamline visits and decrease participant burden	8.6.09
d. change monthly post natal calls for the first 6 months to quarterly for the duration of the trial		Steering Committee decisions to streamline visits and decrease participant burden	8.6.09
10. Added definition of Asthma, wheeze; added eosinphil count; moved IgE measurement to this section	page 12, Section VI. Data Collection	Recommended change by DSMB	8.6.09
11. Addition of croup, laryngotracheobronchitis to lower respiratory illnesses	page 12, Section VI.A4.	Recommended change by DSMB: more complete listing of lower respiratory illnesses	8.6.09
12. Added description of Screening Questionnaire	page 12, Section VI.B.	Steering Committee decision; not previously described	8.6.09
13. Added description of Baseline Maternal Questionnaire	page 12, Section VI.C.	Steering Committee decision; not previously described	8.6.09
14. Monthly Maternal Questionnaire replaces Section VI.B.	page 13, Section VI.D.	Steering Committee decision; Clarification of flow of data and questionnaire	8.6.09
15. Remove sentence beginning with "EMR systems are in place…"	page 13	Steering Committee decision; Clarification of protocol and EMR systems. Paper-based in some centers.	8.6.09
16. Change method for measurement of 25(OH)D; will not use Heartland Assays, Inc.; this will now be measured at the DCC (Channing Lab)	page 13	Recommended by DSMB; potential conflict of interest with Heartland Assays, Inc.	8.6.09
17. Added procedure to follow after discovery of elevated urinary Ca/Cr ratio	pages 13-14, Section VI.G.	Recommended by DSMB; clarification of procedures	8.6.09
18. Changed timing of adminstration of FFQ; second FFQ will now be adminstered at the 32-38 week visit. Added FFQ to 6 month call and 1 year visit	page 14, Section VI.H.	Reflect change in timing of prenatal visit; post natal questionnaires added on recommendation by DSMB	8.6.09
19. Added phrase "…2 readings from each site averaged…"	page 14, Section VI.J.	Clarification to skin pigmentation procedure	8.6.09
20. Added descriptions of stress and depression questionnaires	pages 14-15, Section VI.K.	Steering Committee decision; not previously described	8.6.09

Downloaded From: http://jamanetwork.com/pdfaccess.ashx?url=/data/journals/jama/934885/ by a Harvard University User on 03/30/2017

21. Addition of process for handling depression should it be identified after randomization: The participant will be counseled by the Study Coordinator and the PI will be notified. The participant will then be referred back to her obstetrician for further psychiatric evaluation.	pages 15, Section VI.K.	Steering Committee decision and DSMB recommendation; strategy to handle significant depression should it arise.	8.6.09
22. Changed timing of infant questionnaires from monthly for the first 6 months to quarterly for the duration of the trial	Section VI.M., page 15	Steering Committee decision to streamline data flow and decrease participant burden	8.6.09
23. Switched plans for Specific Aims 1 and 2 to reflect switch in order for these aims	pages 17-18	Steering Committee decision	8.6.09
24. Added details for randomization and block assignment	page 26, Section VII.C.	Recommended change by DSMB	8.6.09
25. Added details about unblinding, interim analysis, and stopping rules	Section VII. H., page 29	Recommended change by DSMB	8.6.09
26. Added details on safety and monitoring of urine Ca/Cr ratio	Section VII. I., pages 29-30	Recommended change by DSMB	8.6.09
27. Changed list of serious adverse events and adverse events	Section VII.J., page 30	Recommended change by DSMB	8.6.09
28. Added details of process of reporting AEs	page 31	Recommended change by DSMB	8.6.09
29. Added details of interim analyses and stopping rules	Section VII. K., page 31	Recommended change by DSMB	8.6.09
30. Added study timeline to include interim analyses schedule	Section VII.L., page 33	Recommended change by DSMB	8.6.09
31. Corrected exclusion criteria for depression questionnaire from >15 to > or = to 15	Section IV, page 7	Clarification of the inclusion criteria	1.8.2010
32. Eliminated Follow-Up Sun Exposure Questionnaire and Determination of Skin Pigmentation for the mother in year 3.	Section V, Table 2, page 11	Corrected the table to reflect the text of the protocol	1.8.2010
33. Corrected procedure for distributing MEMS caps (done locally, not by IDS at the DCC)	Section VI, O., page 16	Clarification of MEMS distribution procedure	1.8.2010
34. Removed wording about each site posting information unique to their location on the website	SectionVII,D., page 27	Corrected the functionality of the VDAART website	1.8.2010
35. Changed age inclusion criteria from 18 -35 to 18-39	Section IV, page 7 and Section VII, page 34	Steering Committee decision, DSMB approval. See proposal: Expanding the Age Requirement for details.	2.8.2010

Downloaded From: http://jamanetwork.com/pdfaccess.ashx?url=/data/journals/jama/934885/ by a Harvard University User on 03/30/2017

36 . Changed urine Ca: Cr ratio to >/= .55mg/mg (or 1.55 mmol/mmol)	Section VI, G., page 14 and SectionVII H., I., page 29, and page 35	Steering Committee decision, DSMB approval. See proposal: Changing the Urine Ca:Cr Ratio Limit	2.8.2010
37. Changed procedure for elevated urine Ca:Cr ratio	Section VI, G., page 14 and Section VII, I., page 30	Steering Committee decision, DSMB approval. See proposal: Changing the Urine Ca:Cr Ratio Limit	2.8.2010
38. Removed Melissa McEnery and changed administrator to Christopher Garcia	VII, B., page 26	Corrected to reflect current personnel	2.17.2010
39. Outlined the procedure for a subsequent elevated urine Ca:Cr ratio	Section VI, G., page 14 and Section VII, I., page 30-31	Steering Committee decision	2.17.2010
40. Added 5th aim: preeclampsia and preterm birth	Section II page 4; Section VI ,P., pages 21-22 ; Section VI, Q., page 25	DCC requested changed, approved by DSMB	3.4.2011
41. Added language about taking anthropometric measurements at office visits	Section V, Page 9 and Section V, Table 2, page 11; Section VI, M., page 15	DCC requested changed, approved by DSMB	3.4.2011
42. Updated the allergens to be assayed at years 1 and 3	Secction VI, A., page 12	DCC initiated	1.23.2012
43. Added the collection of a 2.5 ml pax gene RNA tube for the year 3 visit.	Addendum blood consent added	Steering Committee decision. Needed for gene exptession studies.	2.1.2013

Downloaded From: http://jamanetwork.com/pdfaccess.ashx?url=/data/journals/jama/934885/ by a Harvard University User on 03/30/2017

Addendum to Analysis Plan

Downloaded From: http://jamanetwork.com/pdfaccess.ashx?url=/data/journals/jama/934885/ by a Harvard University User on 03/30/2017

VDAART Analysis Plan (Addendum to Protocol)

All analyses of primary and secondary outcomes stated in the VDAART Protocol will be performed using the intent-to-treat (ITT) paradigm, unless specifically stated.

I. Definition of outcomes.

A. Asthma and recurrent wheezing. The primary outcome of the trial will be development of a doctor's diagnosis of asthma and/or recurrent wheeze. While asthma is acknowledged to have its roots in early life, making a definite diagnosis is difficult until the child is older, and wheezing illnesses may be precursors of an asthma diagnosis. Thus, we will have a composite definition for the primary outcome.

- Asthma will be defined as a parent's report of physician's diagnosis at any time during the first three years of life, based on questionnaire responses. For time-to-event analyses, the time of incident asthma will be determined by the time of first positive questionnaire response.
- Recurrent wheezing will be defined as at least one report of wheezing in the third year of life plus any report of wheezing in any of the first two years of life.

Recognizing that wheezing symptoms may be modified by medication use, variable age of onset, and other variables, the following will be included in the composite outcome:

- children who had at least one wheezing episode (during the 1st 2 years of life), plus an asthma controller medication (defined as steroid inhalers or nebulizers OR steroid pills or liquids OR leukotriene modifiers) in the third year
- children who did not report any wheezing in the first 2 years but who report 2 separate episodes of wheezing in the third year OR are on controller medication (defined as above) on at least 2 separate reports in the third year OR at least 1 report of wheezing and at least 1 separate report of controller medication use in the third year

For recurrent wheezing (and the other wheezing phenotypes), the time of incident recurrent wheezing will be determined by the time of first report of wheezing, for time-to-event analyses. In the event that the child meets the definition based solely on the use of controller medication, the first report of use of this controller medication will be used to determine the time of onset of wheezing for time-to-event analyses.

For these outcomes, because the questions were asked every 3 months, and recognizing that missing questionnaires occurred, event times will be treated as interval-censored.

B. Eczema. Eczema will be defined as a positive response to parent's report of physician's diagnosis at any time during the first three years of life based on the questionnaire responses.

C. Lower respiratory tract infections (LRI). Lower respiratory tract infections (LRIs) will be defined based on parental responses to questionnaires. LRIs will be defined as a parental report of a physician's diagnosis of pneumonia, bronchiolitis, bronchitis, or croup (laryngotracheobronchitis).

Downloaded From: http://jamanetwork.com/pdfaccess.ashx?url=/data/journals/jama/934885/ by a Harvard University User on 03/30/2017

LRIs may occur multiple times over a three-year period in a child. Information on repeated episodes of LRI will be employed for rate estimation and comparison.

D. Total and specific IgE. Total and specific serum IgE will be measured from cord blood samples, and from year 3 samples from the children. Total IgE will be analyzed as a continuous outcome. Sensitization will be defined as any specific IgE ≥ 0.35 IU.

II. Secondary Analyses.

A. Analyses based on levels. Secondary analyses using baseline and follow-up maternal 25OHD levels will be performed. We will investigate the effect of baseline and follow-up maternal levels separately on the development of primary and secondary outcomes in the trial. Additionally, we will categorize mothers into categories based on joint baseline and follow-up levels. Mothers who have baseline levels and follow-up levels ≥ 40 mg/dl will be categorized in the HI-HI group; those who have baseline levels and follow-up levels < 40 mg/dl will be categorized in the LO-LO group; mothers who do not meet these definitions will be categorized either in the HI-LO or LO-HI groups based on their baseline and follow-up levels.

Downloaded From: http://jamanetwork.com/pdfaccess.ashx?url=/data/journals/jama/934885/ by a Harvard University User on 03/30/2017

Summary of changes to Analysis Plan

Downloaded From: http://jamanetwork.com/pdfaccess.ashx?url=/data/journals/jama/934885/ by a Harvard University User on 03/30/2017

Summary of Changes to VDAART Analysis Plan

Change	Date
1. Amendment to recurrent wheeze phenotype and other phenotypes for analysis<br>2. Amendment to analysis plan incorporating time-to-event analyses, using interval censoring	Approved by Steering Committee on January 29, 2015<br>Uploaded to VDAART Website and ClinicalTrials.gov website on February 4, 2015; released on ClinicalTrials.gov website on June 5, 2015
3. Manuscript incorporating changes to analysis plan	Approved by DSMB on April 16, 2015

Downloaded From: http://jamanetwork.com/pdfaccess.ashx?url=/data/journals/jama/934885/ by a Harvard University User on 03/30/2017
