# Supplementary material for: Prenatal vitamin D supplementation reduces risk of asthma/recurrent wheeze in early childhood: A combined analysis of two randomized controlled trials
Source: PLoS One. 2017 Oct 27;12(10):e0186657. doi: 10.1371/journal.pone.0186657 (PMC5659607; doi:10.1371/journal.pone.0186657)
Supplement: S6 Text — (DOCX) [file pone.0186657.s008.docx]

**Effect of prenatal supplementation with vitamin D on asthma or recurrent wheezing in offspring by age 3: the VDAART Randomized Clinical Trial**

Augusto A. Litonjua, MD, MPH^1,2,^*

Vincent J. Carey, PhD^1,2^

Nancy Laranjo, BA^1^

Benjamin J. Harshfield, BA^1^

Thomas F. McElrath, MD, PhD^2,3^

George O’Connor, MD, MS^4^

Megan Sandel MD, MPH^5^

Ronald E. Iverson, Jr., MD, MPH^6^

Aviva Lee-Paritz, MD^6^

Robert C. Strunk MD, PhD^7^

Leonard B. Bacharier, MD^7^

George A. Macones, MD, MSCE^8^

Robert S. Zeiger, MD, PhD^9^

Michael Schatz, MD, MS^9^

Bruce W. Hollis, PhD^10^

Eve Hornsby, PhD^11^

Catherine Hawrylowicz, PhD^11^

Ann Chen Wu, MD, MPH^2,12^

Scott T. Weiss, MD, MS^1,2^

Affiliations:

^1^Channing Division of Network Medicine, Department of Medicine, Brigham and Women’s Hospital, Boston, MA

^2^Harvard Medical School, Boston, MA

^3^Department of Obstetrics and Gynecology, Brigham & Women's Hospital, Boston, MA

^4^Pulmonary Center, Department of Medicine, Boston University School of Medicine, Boston, MA

^5^Department of Pediatrics, Boston Medical Center, Boston, MA

^6^Department of Obstetrics and Gynecology, Boston Medical Center, Boston, MA

^7^Division of Pediatric Allergy, Immunology and Pulmonary Medicine, Department of Pediatrics, Washington University School of Medicine and St. Louis Children’s Hospital, St. Louis, MO

^8^Department of Obstetrics and Gynecology, Washington University School of Medicine, St. Louis, MO

^9^Kaiser Permanente Southern California, San Diego, CA

^10^Department of Pediatrics, Medical University of South Carolina, Charleston, SC

^11^King’s College London School of Medicine, Asthma, Allergy and Respiratory Science, 5th Floor Tower Wing, Guy’s Hospital Campus, London SE1 9RT, UK

^12^Department of Population Medicine, Harvard Pilgrim Health Care Institute and Children’s Hospital Boston, Boston, MA

*To whom correspondence should be addressed:

Augusto A. Litonjua, MD, MPH

Channing Division of Network Medicine

Department of Medicine,

Brigham and Women’s Hospital

181 Longwood Avenue, Boston, MA 02115

Telephone: 617-525-0997

Fax: 617-525-0958

Email: augusto.litonjua@channing.harvard.edu

Key words: Vitamin D; asthma; allergy; randomized controlled trial; Developmental Origins; prenatal, asthma prevention

Abbreviations:

VDAART – Vitamin D Antenatal Asthma Reduction Trial

25(OH)D – 25Hydroxyvitamin D

IgE – Immunoglobulin E

LRI – lower respiratory tract illnesses

IU – International Units

nmol/L – nanomole per liter

ng/ml – nanogram per milliliter

LC-MS/MS – Liquid Chromatography tandem Mass Spectrometry

DSMB – Data and Safety Monitoring Board

NIH – National Institutes of Health

MEMS® - Medication Event Monitoring System

HR – Hazard Ratio

RR – Rate Ratio

OR – Odds Ratio

ITT – Intention-to-treat

PUFAs – polyunsaturated fatty acids

**Text Word Count (excluding Abstract, Legends, Tables, and References) = 3,434**

**Date of Revision: December 11, 2015**

**ABSTRACT (word count=347)**

**Importance:** Asthma and wheezing begin early in life, and prenatal vitamin D deficiency has been variably associated with these disorders in offspring.

**Objective:** To determine whether prenatal vitamin D supplementation can prevent asthma or recurrent wheeze in early childhood.

**Design, Setting, and Participants:** The Vitamin D Antenatal Asthma Reduction Trial (VDAART) was a randomized, double-blind, placebo-controlled trial that began enrollment in October 2009 and completed follow-up in January 2015 in 3 centers across the United States. 881 pregnant women between the ages of 18 and 39 years at high risk for having children with asthma were randomized at 10-18 weeks gestation. Five participants were deemed ineligible shortly after randomization and were discontinued.

**Intervention:** Women were randomized to either daily 4,000 IU vitamin D plus a prenatal vitamin containing 400 IU vitamin D (n=440) or a placebo plus a prenatal vitamin containing 400 IU vitamin D (n=436).

**Main outcomes and Measures:** Co-primary outcomes of (1) parental report of physician-diagnosed asthma or recurrent wheezing through 3 years of age and (2) 3^rd^ trimester maternal 25-hydroxyvitamin D levels.

**Results:** Eight-hundred and ten infants were born in the study, and 806 were included in the analyses for the 3-year outcomes. Two hundred eighteen children developed asthma/recurrent wheeze – 98 (24.3%, 95% CI, 19-29) in the 4,400 IU/day group vs. 120 (30.4%, 95% CI, 26-73) in the 400 IU/day group (hazard ratio = 0.8, 95% CI = 0.6-1.0, p=.051). Of the women receiving 4,400 IU/day, 74.9% had 25-hydroxyvitamin D levels ≥ 75 nmol/L by the 3^rd^ trimester of pregnancy compared with 34.0% in the 400 IU/day group (difference = 40.9%, 95% CI, 34.2-47.5, p<0.001).

**Conclusions and Relevance:**

In pregnant women at risk for having a child with asthma, supplementation with 4400 IU/d of vitamin D compared with 400 IU/d significantly increased vitamin D levels in the women. The incidence of asthma and recurrent wheezing in their children at age 3 was lower by 6.1%, but this did not meet statistical significance; however, the study may have been underpowered.  Longer follow-up of the children is ongoing to determine whether the difference is clinically important.

**Trial Registration:** clinicaltrials.gov Identifier: NCT00920621**INTRODUCTION**

Wheezing illnesses begin as early as the first few weeks of life, suggesting that there may be prenatal determinants involved with the genesis of these illnesses. While wheezing and other asthma-like symptoms are common in the preschool ages, it remains difficult to predict which of these preschoolers will have asthma that persists through mid-childhood and onward. Nevertheless, preschool children with wheezing have lung function deficits that persist through later life.^1,2^ Thus, prenatal interventions that prevent early recurrent wheezing may be important to reduce the short-term morbidity and to prevent the potential long-term sequelae of reduced lung growth.

Vitamin D deficiency occurs worldwide. Vitamin D status is defined by the circulating level of 25-hydroxyvitamin D (25(OH)D), from either plasma or serum. Pregnant and lactating mothers and their neonates are at especially high risk for vitamin D deficiency, and in a nationally representative sample of pregnant US women, 69% had 25(OH)D levels in the insufficient range (i.e. ≤ 75 nmol/L).^3^

Vitamin D deficiency in pregnancy may be important in early asthma and wheezing, as vitamin D has effects on the developing lung and immune system during the fetal and early post-natal periods.^4-8^ Observational studies that have investigated the association of vitamin D in pregnancy on the development of wheeze, asthma, and allergies in early life have produced mixed results. We therefore designed a clinical trial of vitamin D supplementation in pregnancy to address the question of whether supplementation with vitamin D during pregnancy can prevent the development of asthma-related phenotypes in very young children – the Vitamin D Antenatal Asthma Reduction Trial (VDAART).

**METHODS**

*Participants*

Pregnant women were recruited from 3 clinical sites across the United States – Boston Medical Center, Boston, MA; Washington University at St. Louis, St. Louis, MO; and Kaiser Permanente Southern California Region, San Diego, CA. The Data Coordinating Center was based in the Channing Division of Network Medicine, Brigham and Women’s Hospital, Boston, MA. Eligible participants were women between the ages of 18 and 39 years, who presented between the estimated gestational ages of 10 and 18 weeks; who had a history of asthma, eczema, or allergic rhinitis, or whose partner (biologic father of the child) had a history of asthma, eczema, or allergic rhinitis; who was a non-smoker; and who was English- or Spanish- speaking, with intent to participate for 4 years (up to the 3^rd^ birthday of the child). The VDAART study protocol was approved by the institutional review boards at each participating institution and at the Brigham and Women’s Hospital. All women provided written informed consent.

*Study Design*

Details of the study design are available in the protocol (Litonjua et al^9^ and Supplement 1) We conducted a randomized, double-blind, placebo-controlled study of vitamin D (daily 4,000 IU vitamin D plus a multivitamin with 400 IU vitamin D) vs. placebo (daily placebo pill plus a multivitamin with 400 IU vitamin D). Participants were screened for eligibility between October 2009 and July 2011. Follow up of the last child was completed in January 2015. Prenatal interval visits, Labor and Delivery room data collection, and postnatal visits and data collected in these visits are detailed in the eMethods (Supplement 2). Study staff met with pregnant women monthly to administer a brief health questionnaire, assess medication use, and monitor for complications (via the questionnaire and medical record review). Adherence was assessed with electronic medication container caps. After delivery, children were monitored by phone every 3 months and in-person yearly for 3 years, where infants’ health, respiratory symptoms, and medications were assessed. Data collection is summarized in eTable 1, and the number of scheduled and completed phone calls and visits are summarized in eTable 2 (Supplement 2).

Randomization was performed using a system that automates the random assignment of treatment groups to study identification numbers. The randomization scheme employed stratified permuted blocks with randomly varied block sizes of 4 and 6, and one block allocation list per stratum (study site and racial/ethnic group).^9^

The study had 2 primary outcomes. The first was parental report of physician diagnosis of asthma or occurrence of recurrent wheeze in the child's first 3 years of life ascertained from questionnaires administered every 3 months. Parental report of physician's diagnosis of asthma was taken directly from the infant/child questionnaires. Recurrent wheeze was defined by the occurrence of at least 1 of the following 5 conditions: 1) parental report of wheeze after child's second birthday preceded by at least 1 report of wheeze prior to second birthday; 2) report of child's use of asthma controller medication (defined as report of use of steroid inhalers/nebulizers, leukotriene modifiers, or steroid pills or liquids) after the second birthday, preceded by a report of wheeze before the second birthday; 3) 2 or more distinct parental reports of wheeze after the second birthday; 4) at least 1 parental report of wheeze and use of asthma controller medications at distinct visits, both subsequent to the second birthday; 5) 2 distinct reports of use of asthma controller medications after the second birthday. The second, co-primary outcome was achieved maternal 25(OH)D level of ≥ 75 nmol/L at the third trimester sampling. This cutoff was the usual, accepted minimum level of sufficiency based on evidence of associations with outcomes^10^ at the start of the trial. However, the Institute of Medicine released their statement in 2011,^11^ setting the level of ≥ 50 nmol/L as sufficient for bone health. Thus, both cutoffs were analyzed.

Pre-specified secondary outcomes were assessed at 3 years of age, including child 25(OH)D levels, parental report of physician’s diagnosis of eczema with rash in typical distribution, total IgE and allergen sensitization (specific IgE to a panel of aeroallergens and food allergens), and lower respiratory tract infections (LRIs) (defined as parental report of physician-diagnosed bronchitis, bronchiolitis, croup, or pneumonia). Questions about the presence of LRIs were asked every 3 months, allowing for recurrent events. Counts of reported LRIs were assembled from all available follow-up data on each child. For details on the questions from which the outcomes were derived and the measurement of total and specific IgE, please see the eMethods (Supplement 2).

Blood was drawn from mothers at enrollment, 32-38 weeks gestation, and 1 and 3 years after delivery. Cord blood was obtained and children had blood drawn at ages 1 and 3 years. Circulating 25(OH)D from maternal and child plasma samples was determined using the DiaSorin Liaison® chemiluminescence immunoassay,^12^ whereas for cord blood samples liquid chromatography-tandem mass spectrometry^13^ was used (eMethods, Supplement 2). Levels of 25(OH)D are reported in nmol/L (1 nmol/L = 0.4 ng/ml).

Race and ethnicity information was collected because they are determinants of circulating 25(OH)D levels. Participants were asked to first categorize themselves as either Hispanic or non-Hispanic, then to categorize their race into prespecified categories. The child’s ethnicity and race was determined by responses to the questions regarding maternal or paternal ethnicity and race. In the analyses, ethnic and racial groups were collapsed into 4 groups: African American, white Hispanic, non-white Hispanic, and Other. Further details are in the eMethods (Supplement 2).

*Safety Monitoring*

Monitoring for adverse events occurred on a monthly basis during the active intervention phase. The occurrence of prespecified severe adverse events and adverse events (symptomatic hypercalcemia, eclampsia, preeclampsia, HELLP syndrome, death) were identified through maternal questionnaires and review of the obstetrical medical record. In addition, potential hypercalcemia was monitored through monthly checks of urinary calcium-to-creatinine ratios (eMethods, Supplement 2). Neonatal adverse events (preterm delivery, congenital anomalies, fetal or neonatal death) were identified through the labor and delivery medical record review. Attribution of adverse events to vitamin D treatment was determined by independent review by the Data and Safety Monitoring Board (DSMB).

*Statistical Analysis*

The trial was designed to detect a 25% reduction^14^ in the incidence of asthma and recurrent wheeze in the first 3 years of life in the supplemented group. Using data from two birth cohorts,^15,16^ the incidence of asthma and recurrent wheeze in the control group was estimated at 40-50%, when either the mother or the father had asthma or allergies. The recruitment target was 870 pregnant women. Assuming an 8% miscarriage rate and 17-18% loss to follow-up, the targeted sample size at age 3 was 660 children. With a 3-year incidence of 45% in the control group, the power to detect a 25% reduction in the supplemented group was 83%.

In order to make full use of all available observation time and to obtain a fully nonparametric intent-to-treat analysis, the original protocol-based plan to base treatment effect inference on logistic regression analysis was replaced by a plan to use interval-censored event-time analysis. This change in plan was reviewed and approved, prior to unbinding and analysis, by the VDAART steering committee, and the overall final analysis was reviewed and approved by the VDAART DSMB. Tests of treatment effect for the first occurrence of the primary outcome employed the earlier of time to asthma diagnosis or earliest wheeze report or medication event indicative of recurrent wheeze.  Distributions of outcome-free time were compared between groups using nonparametric maximum likelihood estimation to create Kaplan-Meier curves, and optimal nonparametric testing with interval censored response times.^17,18^ Estimates of proportions of children experiencing the primary outcome at selected times are derived from these curves (K-M estimates). The nonparametric procedure with interval-censored response times does not allow incorporation of covariate effects. The Weibull family of parametric models for interval-censored event times^19^ was used to conduct adjusted testing and estimation of treatment and covariate effects. Additional models incorporating effects of clinical center and maternal educational level were conducted post hoc using logistic regression based on the subset of participants providing complete follow-up times, and interval censored Weibull regression employing all available observation time.

Outcome events were analyzed using the interval formed from the last negative visit and first positive visit, or departure from, or completion of study. Individuals who had no reports of asthma or recurrent wheeze up to time of departure from study were censored at that time.

Reports of LRIs were counted, represented as an integer-valued outcome variable and analyzed using a negative binomial generalized linear model to accommodate extra-Poisson variation.^20^ The effect of treatment on the LRI rate per month was estimated using the log of person-months on study as offset. Mean total IgE at age 3 years was compared between groups using a common-variance t test with log-transformed measures. Analyses of maternal vitamin D and secondary outcomes assume missingness occurred completely at random. Statistical analyses were conducted using SAS version 9.3 (SAS Institute, Inc.) and R version 3.1 (R Foundation for Statistical Computing; packages interval, survival, ggplot2). All tests were 2-sided and the significance level was pre-specified at p<0.05, even for the co-primary outcomes, as these have distinct interpretations.**RESULTS**

***Characteristics of the trial population***

Eight hundred eighty-one women were randomized (Figure 1), but 5 women were found to be ineligible, resulting in 876 women – 440 to the 4,400 IU/day dose group and 436 to the 400 IU/day vitamin D group. The baseline characteristics of the pregnant women were comparable between the two treatment groups (Table 1). For the analyses on the childhood outcomes, data on 806 mothers and their children were available – 405 in the 4,400 IU/day group vs 401 in the 400 IU/day group (35 participants in the 4,400 IU dose group and 35 participants in the 400 IU dose group did not contribute data on the infants and children due to fetal deaths or early loss-to-follow-up). Fifty-eight of 806 children did not provide a full three years of observation time, and thus do not provide information on the protocol-defined outcome. No significant differences were observed in comparisons of characteristics of newborns in the two treatment groups (Table 2).

***Effect of treatment on maternal, cord blood, and child 25(OH)D levels***

Maternal levels of 25(OH)D in the 3^rd^ trimester were higher in the 4400 IU/day dose (mean 97.8 nmol/L) vs. the 400 IU/day dose (mean 66.9 nmol/L, difference = 31.0, 95% CI 26.3-35.6, p<0.001; eFigure 1a). The proportion of women who achieved 25(OH)D levels ≥ 75 nmol/L in the 4,400 IU dose group (74.9%) was greater than in the 400 IU dose group (34.0%, difference = 40.9%, 95% CI, 34.2-47.5; p< 0.001)(Table 3). Similarly, the proportion of women who achieved 25(OH)D levels of 50 nmol/L was greater in the 4,400 IU dose group (88.6%) than in the 400 IU dose group (71.6%, difference = 17%, 95% CI, 11.3-22.7, p-value <0.001).For cord blood 25(OH)D levels, samples from the 4,400 IU/day group had statistically significantly greater levels than those from the 400 IU/day group (Table 2). No treatment group related differences in 25(OH)D levels were observed in the mothers 1-year post-partum (eFigure 1a) or in the children at 1 year and 3 years (eFigure 1b).

***Effect of treatment on asthma/recurrent wheeze by age 3 years***

Two hundred and eighteen (27%) of the eligible 806 children developed asthma/recurrent wheezing according to the composite definition by their 3^rd^ year visit with many children fulfilling multiple criteria and with broad overlap between these criteria (eTable3). There were 58 children for whom the outcome could not be resolved (29 in the 4,400 IU/day dose and 29 in the 400 IU/day dose) because they were missing interim or final visit data that would have categorized the outcome; however, all available observation time on all 806 children was used in the interval-censored analysis. In the 4,400 IU/day group, 98 children (K-M estimate 24.3%, 95% CI, 18.7-28.5) developed asthma/recurrent wheeze by age 3 years, whereas in the 400 IU/day group, 120 children (K-M estimate 30.4%, 95% CI,25.7-73.1) developed asthma or recurrent wheeze by age 3 years (hazard ratio 0.8, 95% CI, 0.6-1.0; non-parametric p= 0.051) (Figure 2 and Table 3). K-M estimates of the incidence of asthma/recurrent wheeze at 1 year were 16.0% (95% CI, 11.6-19.5) in the 4,400 IU/day group vs. 24.9% (95% CI, 20.2-31.7) in the 400 IU/day group. At 2 years of age, the K-M estimates were 22.0% (95% CI, 16.7-26.5) in the 4,400 IU/day group vs. 29.4% (95% CI, 25.2-36.1) in the 400 IU/day group.

We performed sensitivity analyses to complement the main ITT analysis (eResults and eTable4), using a Weibull model for interval-censored event times and a logistic regression analysis using only individuals who provided the full 3 year outcomes (N=748). When adjustments for site and maternal education were made, inferences and estimates of treatment effect were consistent with the primary ITT analysis for both modeling approaches. For the site-adjusted logistic regression, the odds ratio was 0.74 (95% CI, 0.54-1.02; P=.063) and for the site and education adjusted model, the odds ratio was 0.72 (95% CI, 0.52-1.00; P=.049).

***Secondary outcomes***

The results of the analyses on the secondary outcomes are presented in Table 3. There were no significant differences in the development of eczema, LRI, and total IgE levels. However, children in the 4,400 IU/day group had fewer positive specific IgE tests (10.7%, 95% CI, 9.4-12.1) than children in the 400 IU/day group (12.4%, 95% CI, 11.4-13.5, p=0.017).

***Safety***

There were no significant differences in the rates of severe adverse events between the 2 treatment groups (eResults and eTable5), and no adverse event was attributed to vitamin D treatment. No events of hypercalcemia in the mothers occurred with vitamin D treatment.

**DISCUSSION**

This randomized, double-blinded multicenter clinical trial studied effects of prenatal treatment with vitamin D on 806 infants followed from birth to 3 years. The study demonstrated that supplementation with 4,400 IU/day of vitamin D significantly raised 25(OH)D levels in pregnant women. We estimated that the 3-year incidence of asthma or recurrent wheeze in the infants was 24.3% with 4400 IU/day and 30.4% for a 400 IU/day supplement. This absolute reduction of 6.1% was not statistically significant (p=0.051). Both the 3-year incidence of asthma or recurrent wheeze observed for recipients of 400 IU/day and the reduction associated with the 4400 IU/day treatment were less than we hypothesized when we designed the study, and thus study power may have been lower than anticipated. In addition, most of the secondary outcomes were not statistically significantly different between groups, and these analyses should be considered exploratory given the null primary outcome and the absence of adjustment for multiple comparisons. Therefore, whether supplementation of pregnant women with vitamin D will reduce asthma and recurrent wheeze in their offspring at age 3 remains unclear. Larger studies and longer follow-up of the children in this study will be needed to answer the question. If additional studies identify a significant effect, given the high prevalence of low vitamin D levels in pregnant women, the effect of this inexpensive intervention on child health could be substantial.

At present, there is no consensus on the definition of a minimal clinically important difference for prenatal interventions against offspring asthma and wheezing. A multi-center trial conducted in 6154 primary care clinic participants that used a multi-intervention approach (increased intake of n-3 PUFAs and oily fish, reduced parental smoking, and reduced indoor dampness) showed a lower asthma incidence by 2 years of age in the intervention group (71/1374, 5.2%) vs. the control group (337/4780, 7.1%) with a difference of 1.9% (OR = 0.72, 95% CI, 0.55-0.93).^21^ Another multi-intervention trial (avoidance of house dust mite, pet allergen, and environmental tobacco smoke; encouragement of breast-feeding; and delayed introduction of solid foods) in 545 high-risk infants on the basis of an immediate family history found a decreased asthma incidence in 2-year old children in the intervention (40/246, 16.3%) vs the control (53/230, 23.0%) groups, for a 6.7% difference (adjusted OR = 0.60, 95% CI, 0.37-0.95).The effect sizes for these diverse interventions are of comparable magnitude to our estimate.

While the diagnosis of asthma in early life is difficult, the primary symptom associated with asthma – wheezing – is common in this period in life, with attendant high resource utilization.^22^ It is recognized that several wheezing phenotypes exist in early childhood (≤3 years old), and a subset of these children (about 40%) will have symptoms that persist through age 6 years^23^ and are ultimately diagnosed as asthma. While several asthma predictive indices have been tested, these are specific but not very sensitive predictors of asthma that persists through age 6.^24,25^Wheezing in early life, with or without a subsequent asthma diagnosis, may have long-term consequences, such as deficits in lung function in adolescence,^1^ impairments in aspects of health-related quality of life,^26^ and more rapid lung function declines in transient wheezers despite attaining normal lung function in early adulthood.^27^ Therefore, we chose the outcome of a parental report of asthma or recurrent wheeze by 3 years of age, as we hypothesized that prenatal interventions are likely to affect this early phenotype.

A number of limitations in VDAART are worthy of mention. The VDAART population was selected because the offspring of these mothers were at high risk of asthma and allergies; thus, caution should be exercised before generalizing these results to non-high risk populations. Timing of vitamin D supplementation during pregnancy may not have been ideal. On average, supplementation was initiated at about the 14^th^ week of gestation, but lung development begins in the 4^th^ week of gestation.^28^ Finally, fewer women than expected achieved the target threshold level of 75 nmol/L 25(OH)D by the third trimester, suggesting that the dose of 4,400 IU/day may not be adequate for all pregnant women.

Because lung development continues throughout childhood,^28^ it is possible that vitamin D continues to exert its influence on the respiratory and immune systems in the post-natal period. In this trial, we saw a significant difference in 25(OH)D levels in cord blood between the 2 groups, but this difference disappeared by 1 year. Consistent with these findings, the differences in asthma or recurrent wheeze between the 2 groups in the first year were greater than in the second and third years, suggesting an early effect that was not sustained. Thus, adequate vitamin D supplementation after birth to maintain 25(OH)D levels in the sufficient range, in addition to prenatal supplementation, may be needed to more fully prevent wheezing illnesses, and perhaps asthma, in early life.

All of these factors - the timing of the intervention, the dose of vitamin D administered, adherence with treatment, and inadequate post-natal vitamin D supplementation - may have limited the effect of the intervention on asthma or recurrent wheeze by 3 years. Future studies will need to address these issues.

**Conclusions**

In pregnant women at risk for having a child with asthma, supplementation with 4400 IU/d of vitamin D compared with 400 IU/d significantly increased vitamin D levels in the women. The incidence of asthma and recurrent wheezing in their children at age 3 was lower by 6.1%, but this did not meet statistical significance; however, the study may have been underpowered. Longer follow-up of the children is ongoing to determine whether the difference is clinically important.

**Author contributions:**Vincent J. Carey, Ph.D., , and Benjamin J. Harshfield, B.A. had full access to all of the data in the study and take responsibility for the integrity of the data and the accuracy of the data analysis.

**Funding:** VDAART was supported by [U01HL091528](http://www.sciencedirect.com.ezp-prod1.hul.harvard.edu/science/article/pii/S1551714414000263#gts0005) from the National Heart, Lung, and Blood Institute. Additional support was provided by U54TR001012 from the National Centers for  Advancing Translational Sciences (NCATS) for participant visits at Boston Medical Center.

**Role of the Sponsor:** NHLBI monitored the conduct of the trial and selected the membership of the Data and Safety Monitoring Board (DSMB). All communication between the investigators and the DSMB coursed through the staff of the NHLBI. All manuscripts, including this current one, during the course of the trial are presented to the DSMB for approval prior to submission for peer review. NHLBI had no role in the design and the conduct of the study, in the collection, analysis, and interpretation of the data; or in the preparation, review, or approval of the manuscript, other than what pertained to the DSMB.

**Data and Safety Monitoring Board:** Lynn M. Taussig, MD (Chair), Mitchell P. Dombrowski, MD, Carol L. Freund, PhD, Frank R. Greer, MD, Martin Hewison, PhD, Dennis R. Ownby, MD, Anthony Scialli, MD, Gerald Teague, MD, John N. Van Den Anker, MD, PhD, O. Dale Williams, PhD, Susan R. Hintz, MD, MS, and Jean Lowe, PhD.

**Data Coordinating Center:** Augusto A. Litonjua, Scott T. Weiss, Vincent J. Carey, Nancy Laranjo, Benjamin J. Harshfield, Sharon O’Toole, Stacey Brown, Amali Chung, SujataDatta, Caroline Holcomb, Sarah Kraft, Bruce Hollis

**Clinical Centers:** *Washington University at St. Louis:* Robert C. Strunk (Lead Clinical Center PI), Leonard B. Bacharier (co-investigator), Michael Nelson, Monica Anderson, Danae Larson, Yvonne Burrage, Jennifer Byers, Megan Isaac-Schmid; *Boston University Medical Center:* George T. O’Connor (Clinical Center PI), Megan Sandel (co-investigator), Katherine Muse, Jessica Long, Erin Collins, Amanda Barbeau, Benvy Caldwell, Tawil Contreras, Corey Costanzo, Ingrid Gonzalez, Mahsan Mohammadi, Ashley Oliver, Penny Price, Chloe Sakow, Danuzia Silva, Aneesa Thannicka, Lena Wang; *Kaiser Permanente Southern California:* Robert S. Zeiger (Clinical Center PI), Michael X. Schatz (co-investigator), Shawn Menafee , Diana Donofero, Kathleen Harden, Terrie Long, Travis Macaraeg, Maria Marcial, Elsa Rodriguez, Elizabeth Sanchez.

**Acknowledgments:** We wish to thank the participants of VDAART for their participation and contribution to the trial;

References

1. Morgan WJ, Stern DA, Sherrill DL, et al. Outcome of asthma and wheezing in the first 6 years of life: follow-up through adolescence. *Am J Respir Crit Care Med.* 2005;172(10):1253-1258.

2. Strachan D, Gerritsen J. Long-term outcome of early childhood wheezing: population data. *The European respiratory journal. Supplement.* 1996;21:42s-47s.

3. Ginde AA, Sullivan AF, Mansbach JM, Camargo CA, Jr. Vitamin D insufficiency in pregnant and nonpregnant women of childbearing age in the United States. *American journal of obstetrics and gynecology.* 2010;202(5):436 e431-438.

4. Zosky GR, Berry LJ, Elliot JG, James AL, Gorman S, Hart PH. Vitamin D deficiency causes deficits in lung function and alters lung structure. *Am J Respir Crit Care Med.* 2011;183(10):1336-1343.

5. Ferreira GB, Gysemans CA, Demengeot J, et al. 1,25-Dihydroxyvitamin D3 promotes tolerogenic dendritic cells with functional migratory properties in NOD mice. *Journal of immunology.* 2014;192(9):4210-4220.

6. Xystrakis E, Kusumakar S, Boswell S, et al. Reversing the defective induction of IL-10-secreting regulatory T cells in glucocorticoid-resistant asthma patients. *The Journal of clinical investigation.* 2006;116(1):146-155.

7. Penna G, Amuchastegui S, Giarratana N, et al. 1,25-Dihydroxyvitamin D3 selectively modulates tolerogenic properties in myeloid but not plasmacytoid dendritic cells. *Journal of immunology.* 2007;178(1):145-153.

8. Yurt M, Liu J, Sakurai R, et al. Vitamin D supplementation blocks pulmonary structural and functional changes in a rat model of perinatal vitamin D deficiency. *American journal of physiology. Lung cellular and molecular physiology.* 2014;307(11):L859-867.

9. Litonjua AA, Lange NE, Carey VJ, et al. The Vitamin D Antenatal Asthma Reduction Trial (VDAART): rationale, design, and methods of a randomized, controlled trial of vitamin D supplementation in pregnancy for the primary prevention of asthma and allergies in children. *Contemp Clin Trials.* 2014;38(1):37-50.

10. Dawson-Hughes B, Heaney RP, Holick MF, Lips P, Meunier PJ, Vieth R. Estimates of optimal vitamin D status. *Osteoporos Int.* 2005;16(7):713-716.

11. Ross AC, Manson JE, Abrams SA, et al. The 2011 report on dietary reference intakes for calcium and vitamin D from the Institute of Medicine: what clinicians need to know. *The Journal of clinical endocrinology and metabolism.* 2011;96(1):53-58.

12. Ersfeld DL, Rao DS, Body JJ, et al. Analytical and clinical validation of the 25 OH vitamin D assay for the LIAISON automated analyzer. *Clin Biochem.* 2004;37(10):867-874.

13. Grebe SK, Singh RJ. LC-MS/MS in the Clinical Laboratory - Where to From Here? *The Clinical biochemist. Reviews / Australian Association of Clinical Biochemists.* 2011;32(1):5-31.

14. Litonjua AA, Weiss ST. Is vitamin D deficiency to blame for the asthma epidemic? *J Allergy Clin Immunol.* 2007;120(5):1031-1035.

15. Gern JE, Visness CM, Gergen PJ, et al. The Urban Environment and Childhood Asthma (URECA) birth cohort study: design, methods, and study population. *BMC Pulm Med.* 2009;9:17.

16. Oken E, Baccarelli AA, Gold DR, et al. Cohort profile: project viva. *Int J Epidemiol.* 2015;44(1):37-48.

17. Sun J. A non-parametric test for interval-censored failure time data with application to AIDS studies. *Statistics in medicine.* 1996;15(13):1387-1395.

18. Fay MP, Shaw PA. Exact and Asymptotic Weighted Logrank Tests for Interval Censored Data: The interval R package. *J Stat Softw.* 2010;36(2):1-33.

19. Miller RG, Jr. *Survival Analysis.* Vol 661118031067, 9781118031063. 2 ed: John Wiley & Sons; 2011.

20. Venables WN, Ripley BD. *Modern Applied Statistics with S-PLUS.* 3rd ed. New York, NY: Springer; 1999.

21. Dotterud CK, Storro O, Simpson MR, Johnsen R, Oien T. The impact of pre- and postnatal exposures on allergy related diseases in childhood: a controlled multicentre intervention study in primary health care. *BMC Public Health.* 2013;13:123.

22. Stevens CA, Turner D, Kuehni CE, Couriel JM, Silverman M. The economic impact of preschool asthma and wheeze. *The European respiratory journal.* 2003;21(6):1000-1006.

23. Martinez FD, Wright AL, Taussig LM, Holberg CJ, Halonen M, Morgan WJ. Asthma and wheezing in the first six years of life. The Group Health Medical Associates. *N Engl J Med.* 1995;332(3):133-138.

24. Bacharier LB, Guilbert TW. Diagnosis and management of early asthma in preschool-aged children. *J Allergy Clin Immunol.* 2012;130(2):287-296; quiz 297-288.

25. Ducharme FM, Tse SM, Chauhan B. Diagnosis, management, and prognosis of preschool wheeze. *Lancet.* 2014;383(9928):1593-1604.

26. Braig S, Brandt S, Wabitsch M, et al. Age-specific influence of wheezing phenotypes on pre-adolescent and adolescent health-related quality of life. *Pediatr Allergy Immunol.* 2014;25(8):781-787.

27. Edwards CA, Osman LM, Godden DJ, Douglas JG. Wheezy bronchitis in childhood: a distinct clinical entity with lifelong significance? *Chest.* 2003;124(1):18-24.

28. Schittny J, Burri P. Development and growth of the lung. In: Fishman A, Elias J, Fishman J, Grippi M, Senior R, Pack A, eds. *Fishman's Pulmonary Diseases and Disorders.* Vol 1. New York: McGraw-Hill Professional; 2008:91-114.

**Figure Legends:**

**Figure 1** – **Participant Flow**

**Figure 2 – Asthma or Recurrent Wheeze-Free Proportion by Treatment.** Kaplan-Meier survival estimates..Shown are asthma or recurrent wheeze-free proportions in the children from mothers in the two treatment groups. 95% CI estimates are displayed at 1 year, 2 years, and 3 years of age intervals for each curve. Estimates were obtained from nonparametric maximum likelihood estimation, with optimal nonparametric testing with interval censored response times. These interval censored response times lead to gaps in the curves, which we have denoted with diagonal lines. The hazard ratio for the time to first event of asthma or recurrent wheeze was 0.8, 95% CI, 0.6-1.0, p-value = 0.051.

**Table 1. Baseline Characteristics of VDAART mothers (n=876)**

|  | | | | | | | | | | | | | | | | | | |  | 4400 IU  (n=440) | 400 IU  (n=436) |
| --- | --- | --- | --- | --- | --- | --- | --- | --- | --- | --- | --- | --- | --- | --- | --- | --- | --- | --- | --- | --- | --- |
| Age in yrs, mean (sd) | | | | | | | | | | | | |  | | | | | | | 27.5 (5.5) | 27.3 (5.6) |
| Gestation age in weeks, mean (sd) | | | | | | | | | | | | | | | | | |  | | 14.1 (2.8) | 14.2 (2.7) |
| Vitamin D (25 OHD) nmol/l, mean (sd) | | | | | | | | | | | | | | | |  | | | | 58.1 (25.3)  n=437 | 56.2 (25.2)  n=433 |
| Mother | | | | | | | | | | | | | | | |  | | | |  |  |
|  | | | | | | | | | | | | | | | asthma, n (%) | | | | | 191 (43) | 167 (38) |
|  | | | | | | | | | | allergic rhinitis, n (%) | | | | | | | | | | 275 (63) | 283 (65) |
|  | | | | | | | | | | | | | | eczema, n (%) | | | | | | 139 (32) | 139 (32) |
| Father | | | | | | | | | | | | | | | |  | | | |  |  |
|  | | | | | | | | | | | | | | asthma, n (%) | | | | | | 108 (25) | 94 (22) |
|  | | | | | | | | | | allergic rhinitis, n (%) | | | | | | | | | | 173 (39) | 192 (44) |
|  | | | | | | | | | | | | | | eczema, n (%) | | | | | | 81 (18) | 63 (14) |
| Mother’s Race and ethnicity | | | | | | | |  | | | | | | | | | | | |  |  |
|  | | | | | | | | African American, n (%) | | | | | | | | | | | | 190 (43) | 190 (44) |
|  | | | | | | white Hispanic, n (%) | | | | | | | | | | | | | | 59 (13) | 61 (14) |
|  | | | | | white non-Hispanic, n (%) | | | | | | | | | | | | | | | 114 (26) | 116 (27) |
|  | | | | | | | | | | | | | | | | | Other, n (%) | | | 77 (18) | 69 (16) |
| Education completed | | | | | | | | |  | | | | | | | | | | |  |  |
|  | | | | | | less than high school, n (%) | | | | | | | | | | | | | | 66 (15) | 42 (10) |
|  | | | high school, technical school, n (%) | | | | | | | | | | | | | | | | | 123 (28) | 142 (33) |
|  | | | | | | | | | | | some college, n (%) | | | | | | | | | 108 (25) | 105 (24) |
|  | college graduate or graduate school, n (%) | | | | | | | | | | | | | | | | | | | 143 (33) | 147 (34) |
| Marital status | |  | | | | | | | | | | | | | | | | | |  |  |
|  | | | | | | | | | | | | | | | married, n (%) | | | | | 191 (43) | 203 (47) |
|  | | | | | | | Divorced or separated, n (%) | | | | | | | | | | | | | 13 (3) | 10 (2) |
|  | | | | | | | | | | | | not married, n (%) | | | | | | | | 236 (54) | 223 (51) |
| Household income | |  | | | | | | | | | | | | | | | | | |  |  |
|  | | | | | | | | | | | | | | < 30,000 n (%) | | | | | | 132 (30) | 128 (29) |
|  | | | | | | | | | | | | 30 - 49,999, n (%) | | | | | | | | 62 (14) | 58 (13) |
|  | | | | | | | | | | | | 50 - 74,999, n (%) | | | | | | | | 50 (11) | 51 (12) |
|  | | | | | | | | | | | | 75 - 99,999, n (%) | | | | | | | | 42 (10) | 39 (9) |
|  | | | | | | | | | | | | 100 - 149K, n (%) | | | | | | | | 35 (8) | 36 (8) |
|  | | | | | | | | | | | | >150,000 n (%) | | | | | | | | 18 (4) | 14 (3) |
|  | | | | Refused or unknown, n (%) | | | | | | | | | | | | | | | | 101 (23) | 110 (25) |

**Table 2. Pregnancy and infant characteristics of mothers whose children contribute delivery and/or follow-up data (n=806)***

| **Maternal Characteristics** | | | | | | | | |  | | 4400 IU (n=405) | | 400 IU (n=401) | | Mean difference between groups (95% CI) |
| --- | --- | --- | --- | --- | --- | --- | --- | --- | --- | --- | --- | --- | --- | --- | --- |
| **25(OH)D levels (nmol/L):** | | | | | | | | |  | |  | |  | |  |
| Baseline | | Mean (95% CI) | | | | | | | | | 58.2 (55.7 , 60.8) n=402 | | 56.4 (53.9 , 58.9) n=399 | | 1.8 (-1.7 , 5.4) |
| 3rd Trimester | | | | | Mean (95% CI) | | | | | | 98.1 (94.3 , 102.0) n=383 | | 66.9 (64.2 , 69.6) n=387 | | 31.2 (26.6 , 35.9) |
| Cord Blood (measured by  LC-MS/MS) | | | | | | Mean (95% CI) | | | | | 70.0 (66.5 , 73.5) n=303 | | 48.0 (45.3 , 50.7) n=317 | | 22.0 (17.6 , 26.4) |
| Time on study, days (Enrollment to Delivery) | | | | Mean (95% CI) | | | | | | | 173.5 (171.2 ,175.8) | | 174.4 (172.0 , 176.8) | | -0.9 (-4.3 , 2.4) |
| Medication adherence (MEMS) percent | | | | Mean (95% CI) | | | | | | | 70 (67.3 , 72.7) n=402 | | 71.3 (68.6, 74.0) n=400 | | -1.3 (-5.2 , 2.5) |
|  |  | | | | | | | | | | n | % | n | % | Rate difference (95% CI)^a^ |
| Mode of delivery | | | | | | |  | | | |  |  |  |  |  |
| C-section |  | | | | | | | | | | 123 | 31 | 116 | 29 | 2 (-5, 8) |
| Vaginal |  | | | | | | | | | | 279 | 69 | 285 | 71 | -2 (-8, 5) |
| Delivery < 37 weeks | | |  | | | | | | | | 40 | 10 | 31 | 8 | 2 (-2, 6) |
| **Birth and child's first year of life characteristics** | | | | | | | | | | | 4400 IU (n=405) | | 400 IU (n=401) | | Mean difference between groups (95% CI) |
| Birth weight (g) | | | | | | Mean (95% CI) | | | | | 3267.9 (3214.9 , 3321.1) n=402 | | 3282.5 (3224.9 , 3340.1) n=401 | | -14.5 (-92.8 , 63.7) |
| Birth length (cm) | | | | | | Mean (95% CI) | | | | | 50.6 (50.3 , 50.9) n=401 | | 50.7 (50.3 , 51.0) n=399 | | -0.1 (-0.5 , 0.4) |
| Head circumference (cm) | | | | | | Mean (95% CI) | | | | | 34 (33.8 , 34.2) n=398 | | 34 (33.8 , 34.2) n=398 | | 0 (-0.3 , 0.3) |
|  |  | | | | | | | | | | n | % | n | % | Rate difference (95% CI)^a^ |
| Child's gender  Male  Female | | |  | | | | | | | | 200  205 | 49  51 | 220  181 | 55  45 | -5 (-13, 2)  5 (-2, 13) |
| Child's Race and Ethnicity  African American  White Hispanic  White, non-Hispanic  Other | | | | | | | |  | | | 199  53  82  71 | 49  13  20  18 | 191  51  79  80 | 48  13  20  20 | 2(-6, 9)  0 (-5, 5)  1 (-5, 6)  -2 (-8, 3) |
| Reported breastfeeding in the first year | | | | | | | | | |  | 213 | 55 | 206 | 53 | 2 (-6, 9) |
| Reported formula use in the first year | | | | | | | | | |  | 338 | 87 | 330 | 85 | 2 (-3, 7) |
| Reported use of vitamin D drops (400 IU/drop per day) in the first year | | | | | | | | | |  | 179 | 46 | 169 | 43 | 2 (-5, 10) |
| Reported use of multivitamin drops in the first year | | | | | | | | | |  | 74 | 19 | 87 | 22 | -3 (-9, 3) |

*Numbers and percentages reflect some missing data.

^a^ Differences may not sum up due to rounding.

Table 3. Treatment comparisons for primary and secondary outcomes* in VDAART

| **Co-primary Endpoints** | |  | **4400 IU/day** | **400 IU/day** | **Difference(95% CI)** | | **2-sided p (ITT)** | | |
| --- | --- | --- | --- | --- | --- | --- | --- | --- | --- |
| Asthma or recurrent wheeze in first 3 years of life | | |  |  |  |  | |  |  |
|  | # positive/N (%) |  | 98/405 (24.3) | 120/401 (30.4) | -6.1% (-30%-18%) | | 0.051 | | |
|  | HR (2-sided 95% CI) | | 0.8 (0.6-1.0) | 1.0 (---) |  | |  | |  |
| Maternal 25(OH)D≥ 75 nmol/L | | |  |  |  | |  | | |
|  | # positive/N (%) |  | 289/386 (74.9) | 133/391 (34.0) | 40.9% (34.2-47.5) | | <0.001 | | |
|  |  | |  |  |  | |  | | |
| **Secondary Endpoints** | |  |  |  |  | |  | | |
| Eczema with rash in first 3 years of life | |  |  |  |  | |  | | |
|  | # positive/N (%) |  | 83/405 (21%) | 89/401(23%) | -2% (-17%-13%) | | 0.562 | | |
|  | HR (2-sided 95% CI) | | 0.9(0.7-1.2) | 1.0 (---) |  | |  | | |
| Lower respiratory infections in first three years of life | | |  |  |  | |  | | |
|  | % (# reporting any/N) | | 31.9% (129/405) | 34.2% (137/401) | -2.3% (-9.1%-4.4%) | | 0.07 | | |
|  | # unique events reported | | 222 | 276 |  | |  | | |
|  | Negative Binomial rate ratio (2-sided 95% CI) | | 0.8 (0.6-1.0) | 1.0 (---) |  | |  | | |
| Log Total IgE concentration at age 3 | |  |  |  |  | |  | | |
|  | Geometric mean (95% CI) |  | 29.3(24.2-35.4) | 37.3 (30.7- 45.4) | -8 (-17.2-1.2) | | 0.08 | | |
|  | N |  | 276 | 262 |  | |  | | |
| Any allergic sensitization by age 3 | | |  |  |  | |  | | |
|  | # reporting/N (%) | | 146/279 (52.3) | 147/264 (55.7) | -3.4% (-12.0-5.4) | | 0.49 | | |
|  | (2-sided 95% CI) | | (46.3-58.3) | (49.5-61.8) |  | |  | | |
| Total number of positive specific IgE tests | |  |  |  |  | |  | | |
|  | % positive (# of events/# tests) | | 10.7% (414/3881) | 12.4% (458/3700) | -1.7% (-3.4%-0.0%) | | 0.017 | | |
|  | Mean (sd). # of specific tests per person | | 13.9 (0.48) | 13.9 (0.86) |  | |  | | |

*Asthma or recurrent wheeze, and eczema with rash were analyzed as events with interval-censored times of occurrence; estimated proportions are based on the method of Kaplan and Meier.  Hazard ratios for these outcomes were estimated using a Weibull model, and p-values were derived from the nonparametric procedure of Sun. Maternal achievement of 25(OH)D levels of ≥75 nmol and report of any allergic sensitizations by age 3 were analyzed as Bernoulli outcomes. Lower respiratory infection was analyzed as a count of recurring events governed by the negative binomial distribution, to accommodate extra-Poisson variation in these counts (binomial generalized linear model).  The total number of positive specific IgE per child was recorded as a binary outcome per test, summed to a binomial outcome per visit.  Log total IgE was analyzed as a Gaussian variable, transformed to geometric mean (t-test).
