# Supplementary material for: Prenatal vitamin D supplementation reduces risk of asthma/recurrent wheeze in early childhood: A combined analysis of two randomized controlled trials
Source: PLoS One. 2017 Oct 27;12(10):e0186657. doi: 10.1371/journal.pone.0186657 (PMC5659607; doi:10.1371/journal.pone.0186657)
Supplement: S7 Text — (DOCX) [file pone.0186657.s009.docx]

**Title**:

**Effect of Vitamin D_3_ Supplementation during Pregnancy on Risk of Persistent Wheeze in the Offspring**

*A Randomized Clinical Trial*

**Authors:**

Bo L Chawes, MD, PhD^1^*; Klaus Bønnelykke, MD, PhD ^1^*; Jakob Stokholm, MD, PhD ^1+2^; Nadja H Vissing, MD^1^; Elín Bjarnadóttir, MD^1+2^; Ann-Marie M Schoos, MD, PhD^1^; Helene M Wolsk, MD^1^; Tine Marie Pedersen, MD^1+2^; Rebecca K Vinding, MD^1+2^; Sunna Thorsteinsdóttir, MD^1^; Lambang Arianto, MD^1^; Henrik W Hallas, MD^1^; Lene Heickendorff, MD, DMSc^3^; Susanne Brix, MSc, PhD^4^; Morten A Rasmussen, MSc, PhD^1^; Hans Bisgaard, MD, DMSc ^1^.

*These authors contributed equally to the manuscript.

**Affiliations:**

1) Copenhagen Prospective Studies on Asthma in Childhood, Herlev and Gentofte Hospital, University of Copenhagen, Copenhagen; Denmark.

2) Department of Pediatrics, Naestved Hospital, Naestved; Denmark.

3) Department of Clinical Biochemistry, Aarhus University Hospital, Aarhus; Denmark.

4) Center for Biological Sequence Analysis, Department of Systems Biology, Technical University of Denmark, Lyngby; Denmark

**Correspondence:**

Professor Hans Bisgaard, MD, DMSc
Copenhagen Prospective Studies on Asthma in Childhood, Herlev and Gentofte Hospital, University of Copenhagen

Ledreborg Alle 34

DK-2820 Gentofte; Denmark

Tel: (+45) 39777360

Fax: (+45) 39777129
E-mail: bisgaard@copsac.com

Website: www.copsac.com

**Article type:** Original article

**Word count:**  2,9643,074

**Tables:** 2 + 3 online

**Figures:** 4 + 1 online

**Supplements:** 2

**Short title:** Vitamin D_3_ supplementation during pregnancy and childhood wheezing

**Date of revision:** December 9^th^ 2015

**Abbreviations:** COPSAC = Copenhagen Prospective Study on Asthma in Childhood; LCPUFA = long chain polyunsaturated fatty acids; RCT = randomized clinical trial.

**ABSTRACT** (370 words)

**Importance:** Observational studies have suggested that increased dietary vitamin D intake during pregnancy may protect against wheezing in preschool children, but the preventive effect of vitamin D supplementation to pregnant women is unknown.

**Objective:** To determine whether supplementation of vitamin D_3_ during the third trimester of pregnancy reduces the risk of persistent wheeze in the offspring.

**Design, Setting and Participants:** A double-blind, single-center, randomized clinical trial conducted within the Copenhagen Prospective Study on Asthma in Childhood 2010 (COPSAC_2010_) cohort. Enrollment began March 2009 aiming for 708 participants, but due to delayed ethical approval only 623 women were recruited at 24 weeks of pregnancy. Clinical follow-up of the children (N=581) was completed when the youngest child turned 3 years old in March 2014.

**Intervention:** Vitamin D_3_ (2400IU/day) (N=315) or matching placebo tablets (N=308) from pregnancy week 24 to 1 week postpartum. All women received 400IU/day of vitamin D_3_ as part of usual pregnancy care.

**Main Outcome Measure:** Age at onset of persistent wheeze during the first 3 years of life diagnosed adherent to an algorithm based on 9 scheduled and additional unscheduled clinic visits and a day-to-day symptom diary. Secondary outcomes were number of wheezy episodes, asthma, neonatal airway immunology, systemic inflammation, respiratory infections, allergic sensitization and eczema.

**Results:** During the first 3 years of life, persistent wheeze was diagnosed in 47 (16%) of children in the vitamin D_3_ group and 57 (20%) of controls. Vitamin D_3_ supplementation was not associated with the risk of persistent wheeze: hazard ratio, 0.76; 95% CI, 0.52-1.12, *P*=.16, but the number of wheezy episodes was reduced: mean 5.9 vs. 7.2 episodes; incidence risk ratio, 0.83; 95% CI, 0.71-0.97, *P*=.02, and the airway immune profile was up-regulated (principle component analysis, *P*=.04). There was no effect on additional end-points.

In the vitamin D_3_ vs. control group, intrauterine death was observed in 1 (0%) vs. 3 (1%) and congenital malformations in 17 (5%) vs. 23 (8%).

**Conclusions and Relevance:** The use of 2800IU/day of vitamin D_3_ during pregnancy, compared with 400IU/day, did not result in a statistically significant reduced risk of persistent wheeze in the offspring through age 3. However, interpretation of the study is limited by a wide confidence interval that includes a clinically important protective effect.

**Trial Registration:** ClinicalTrials.gov identifier: NCT00856947.

**Keywords, MeSH:** asthma, intervention, pregnancy, RCT, vitamin D

**INTRODUCTION**

Asthma often begins in early childhood and is the most common chronic childhood disorder^1^. The incidence has increased during the last half-century in westernized societies, presumably related to a changing lifestyle or environment inducing immune deregulation in early life and subsequent chronic inflammation^2^. In parallel, vitamin D deficiency has also become a common health problem in westernized societies, possibly caused by a more sedentary indoor lifestyle and decreased intake of vitamin D containing foods^3^. Vitamin D possesses a range of immune regulatory properties, and it has been speculated that vitamin D deficiency during pregnancy may affect fetal immune programming and contribute to asthma pathogenesis^4,5^.

This hypothesis is supported by a recent observational study in the Copenhagen Prospective Studies on Asthma in Childhood 2000 (COPSAC_2000_) high-risk birth cohort showing an association between low cord blood vitamin D levels and an increased risk of childhood wheezing^6^. Results of other studies have been ambiguous, as some showed an association between increased maternal dietary vitamin D intake^7,8^ or increased cord blood vitamin D levels^6,9,10^ and reduced risk of wheezy disorders, whereas others reported no such association^11,12^. No randomized clinical trial (RCT) of vitamin D supplementation to pregnant women has investigated whether such relationship is causal and thus modifiable.

Therefore, we performed a double-blind RCT of vitamin D_3_ supplementation during pregnancy in the 2010 population-based COPSAC_2010_ mother-child cohort to assess the risk of persistent wheeze during the first 3 years of life^13^.

**METHODS**

The trial protocol and statistical plan are available in **Supplement 1**^14^.

The COPSAC_2010_ study was approved by the Local Ethics Committee with a separate approval for the vitamin D_3_ RCT during pregnancy, the Danish Data Protection Agency, and the Danish Health and Medicines Authority. Written and oral informed consent was obtained at enrollment of participants.

*Study Design*

This double-blind, placebo-controlled study recruited pregnant women in Denmark by a monthly surveillance of reimbursement to general practitioners for the first pregnancy visit. The identified women were invited to contact the COPSAC clinic by telephone, and they were given detailed verbal information and screened for eligibility. Thereafter, detailed information was sent and the first visit to the clinic was planned within pregnancy weeks 22–26. Exclusion criteria were gestational age above week 26; any endocrine, cardiovascular, or nephrological disorders; or vitamin D intake >600 IU/day^13^.

The offspring were recruited to the COPSAC_2010_ birth cohort and followed by the study pediatricians with scheduled visits at 1 week, 1, 3, 6, 12, 18, 24, 30, and 36 months, and with acute visits for any respiratory or skin-related symptoms. The symptom burden between visits was captured with daily diary cards monitoring: (1) significant troublesome lung symptoms including components of cough, wheeze, and dyspnea; (2) skin symptoms; and (3) respiratory infections. The study pediatricians acted as general practitioners for the cohort and were solely responsible for diagnosis and treatment of asthma, allergy, and eczema adhering to predefined algorithms and blinded to the intervention^13^.

*Study Intervention*

Women were randomized 1:1 to a daily dose of 2400IU vitamin D_3_ (cholecalciferol) supplementation or matching placebo tablets (Camette, Denmark A/S) from pregnancy week 24 to 1 week postpartum. In addition, all women were instructed to continue supplementation of 400IU vitamin D_3_ during pregnancy as recommended by the Danish National Board of Health; thus, the study is a dose-comparison of 2800 IU/d vs 400IU/d of vitamin D_3_. Women were randomized using a computer-generated list of random numbers, supplied by an external investigator, who had no further involvement in the RCT. The intervention code was unblinded when the youngest child turned 3 years old or in case of a medical emergency.

The mother’s serum vitamin D level was measured^15,16^ at time of randomization corresponding to pregnancy week 24 and at the first visit after birth, i.e. 1 week postpartum (**Supplement 2**), when the women stopped the supplement. This allowed assessment of adherence to the treatment plan, which was further complemented by counting returned tablets.

*Primary End-point*

Persistent wheeze was diagnosed according to a previously validated quantitative algorithm^17,18^ requiring all of the following: (1) recurrent wheeze (verified diary recordings of ≥5 episodes of troublesome lung symptoms lasting ≥3 days within 6 months); (2) typical symptoms of asthma, e.g. exercise induced symptoms, prolonged nocturnal cough, persistent cough outside common cold; (3) need for intermittent bronchodilator; and (4) response to a 3-month trial of inhaled corticosteroids and relapse upon cessation^17^. Risk of persistent wheeze analyzed by age-at-onset analysis (Cox regression) from birth to age 3 years was the primary end-point of the RCT.

*Secondary End-points*

Asthma was diagnosed in children fulfilling the persistent wheeze criteria at age 3 years.

Wheezy episodes included the number of episodes of troublesome lung symptoms lasting ≥3 consecutive days in the first 3 years of life.

Upper respiratory infections included episodes of common cold, acute tonsillitis, and acute otitis media until age 3 years^13^.

Lower respiratory infections included pneumonia and bronchiolitis. Pneumonia was diagnosed in children with significant cough, tachypnea, fever, and abnormal lung stethoscopy, whereas bronchiolitis was defined as cough, tachypnea, chest retractions, auscultative widespread crepitation and/or rhonchi in a child below 1 year^19–21^.

Airway immunology was assessed at age 1 month by measuring unstimulated levels of 20 cytokines and chemokines (IL-12p70, IP-10, IFN-γ, TNF-α, MIP-1β, MCP-1, MCP-4, IL-4, IL-5, IL-13, eotaxin-1, eotaxin-3, TARC, MDC, IL-17, IL-1β, IL-8, TGF-β1, IL-10, and IL-2) in airway mucosal lining fluid sampled by a nasosorptive technique as previously detailed^22–24^ (**Supplement 2**).

Systemic low-grade inflammation was determined by measuring serum levels of high-sensitivity C-reactive protein (hs-CRP), interleukin-6 (IL-6), tumor necrosis factor-α (TNF-α) and CXCL8 (IL-8) at age 6 months^25^.

Allergic sensitization was diagnosed at 6 and 18 months as any skin prick test ≥2 mm (ALK-Abelló, Hørsholm, DK) or specific IgE≥0.35 kU_A_/L against raw milk, pasteurized egg, dog or cat (ImmunoCAP; Thermo Fischer Scientific, Allerød, DK)^13^.

Eczema at age 0-3yrs was diagnosed according to the criteria of Hanifin&Rajka^26^ including typical morphology and localization of skin lesions^27,28^.

*Safety*

Parents were routinely interviewed about the mother’s medical history during pregnancy and the children’s health at all scheduled and unscheduled visits to the research unit. All diagnoses were registered online in the dedicated COPSAC database.

*Study Power*

The prespecified sample size calculation found that 708 participants (354 in each group) would be required to obtain 80% power to detect a difference between the treatment groups (alpha=0.05, two-tailed) based on a 12% expected frequency of persistent wheeze in the control group (estimated from the 16.5% observed in the COPSAC_2000_ high-risk cohort) and an effect of 0.5 in the vitamin D_3_ group.

*Statistical Analysis*

The effect of vitamin D_3_ supplementation on the primary endpoint, age at onset of persistent wheeze, as well as lower respiratory infections and eczema was analyzed by Cox proportional hazards regression, where p-values correspond to Wald tests. The children were retained in the model from birth until age of diagnosis, drop out, or age at their last clinic visit before the RCT was unblinded.

The effect of vitamin D_3_ supplementation on the cross-sectional end-points asthma and allergic sensitization was analyzed by logistic regression, whereas the effect on number of wheezy episodes and upper respiratory infections was analyzed by a generalized estimating equation (GEE) Poisson regression model taking account of repeated subject measurements.

The effect on airway immunology in the vitamin D_3_ vs. control group was analyzed and visualized by a principal component analysis^29^ (PCA) capturing the overall immunological trends in the data and their relation to the intervention analyzed by Wilcoxon rank sum test. Initially, the mediator levels were log-transformed. Prior to the PCA the variables were scaled to unit variance.

The primary analysis of age at onset of persistent wheeze is an intention to treat analysis, which is presented crude and adjusted for sex, birth season, maternal vitamin D level at randomization, and participation in a concomitant factorial designed, double-blind, RCT of 2.4g/day n-3 long chain polyunsaturated fatty acids (LCPUFA) during pregnancy (ClinicalTrials.gov: NCT00798226). A significance level of 0.05 in 2-sided test was used in all types of analyses, which were conducted using SAS version 9.3 for Windows (SAS Institute Inc., Cary, NC, US) and MATLAB R2014a (Natick, MA, US). No imputation was performed for missing data.

Additional methodological details are outlined in **Supplement 2** and the COPSAC_2010_ design paper^13^.

**RESULTS**

Of 1,876 pregnant Danish women screened for eligibility to the COPSAC_2010_ mother-child cohort, 1,138 were not included or declined to participate. We randomized 623 of the 738 eligible women from March 4^th^, 2009 to November 17^th^, 2010 as the ethical approval of the vitamin D trial was delayed during enrollment of the first 115 eligible women into the COPSAC_2010_ cohort. In addition, 43 women were withdrawn before childbirth. We unblinded 8 randomizations during pregnancy (including 4 intrauterine deaths) and 3 children were excluded due to chronic disorders, leaving 581 children for the primary analysis (**Figure 1**). The clinical follow-up rate of the children after unblinding the RCT on March 28^th^, 2014 was 94% at age 0-3yrs.

At randomization, 52% of the women had sufficient vitamin D levels (>75nmol/L), 34% had insufficient levels (50-75nmol/L) and 14% had deficient levels (<50nmol/L). Baseline characteristics of the participating mother-child pairs are outlined in **Table 1** showing no clinically important differences in maternal serum vitamin D levels at randomization (mean (SD) level: 76.5nmol/L (25.5) vs. 76.4nmol/L (25.3)) or season of birth (e.g. winter birth: 36% vs. 36%).

Adherence to the intervention, defined as mothers taking >80% of the prescribed tablets, was 74%. The intervention resulted in a significant increase in maternal serum vitamin D level in the treatment group (mean (SD) at randomization vs. postpartum: 76.5nmol/L (25.5) vs. 106.2nmol/L (35.7)) compared with the control group (76.4nmol/L (25.3) vs. 73.1nmol/L (31.7)): mean difference 33.0nmol/L [95% CI, 27.1-39.0], p<0.0001 (**eFigure 1**). Correspondingly, the percentage of women with sufficient levels of vitamin D (>75nmol/L) after the intervention was 81% in the treatment group compared to 44% in the control group: mean difference 37% [95% CI, 30-45], p<0.0001.

*Vitamin D_3_ Supplementation and Risk of Persistent Wheeze*

During the first 3 years of life, persistent wheeze was diagnosed in 104 (18%) of the children, with 47 (16%) affected children in the vitamin D_3_ group vs. 57 (20%) in the control group. The intention to treat analysis of age at onset of persistent wheeze did not show a significant effect on risk of persistent wheeze from vitamin D_3_ supplementation during pregnancy: HR=0.76 [95% CI, 0.52-1.12], p=0.16 (**Figure 2**).

Sex, season of birth, maternal vitamin D_3_ level at randomization and the n-3 LCPUFA RCT did not interact with the supplementation effect (p>0.17 for all interaction analyses). Adjusting the primary analysis for these variables did not modify the results: HR=0.75 [0.51-1.10], p=0.14 (**Table 2**).

A post hoc analysis of the effect of post-intervention levels of vitamin D_3_ showed a reduced risk of persistent wheeze per 10nmol/L increase in maternal serum vitamin D level after intervention: HR=0.94 [0.89-0.99], p=0.03 (**Figure 3**). This is consistent with the effect estimate for persistent wheeze of 0.76 (approximately 20% reduced risk) associated with the intervention, which on average resulted in a 33nmol/L higher level of vitamin D after the intervention. Adjusting the analysis for sex, season of birth, maternal smoking during pregnancy, and vitamin D_3_ level at randomization did not modify the result: HR=0.93 [0.88-0.99], p=0.02.

*Vitamin D_3_ Supplementation and Secondary End-points (****Table 2****)*

Wheezy episodes: Vitamin D_3_ supplementation during pregnancy resulted in significantly fewer wheezy episodes during the first 3 years of life in the intervention group vs. the control group: mean 5.9 vs. 7.2 episodes, IRR=0.83 [0.71-0.97], p=0.02.

Asthma at age 3yrs was diagnosed in 69 (13%) of the children, with 32 (12%) in the vitamin D_3_ group and 47 (14%) in the control group: OR=0.82 [0.50-1.36], p=0.45.

Airway immunology (**eTable 1**): The purely data driven PCA showed a uniform up-regulated mediator pattern in principle component 1 (PC1), which explained 54% of the variation in the data and significantly separated children in the intervention groups (p=0.04) (**Figures 4A-B**).

Systemic low-grade inflammation: Children in the vitamin D_3_ vs. control group did not show a significant difference in levels of hs-CRP at age 6 months: median (interquartile range), 1.10mg/L (0.56-4.23) vs. 1.45mg/L (0.51-4.90), p=0.09. There were also no significant differences in levels of IL-6, TNF-α or CXCL8 between children in the intervention vs. control group **(eTable 2**).

Respiratory infections: Vitamin D_3_ supplementation did not modify the number of upper respiratory infections (5.2/yr vs. 5.3/yr, IRR=0.99 [0.90-1.09], p=0.84). Lower respiratory infections at age 0-3yrs were diagnosed in 94 (32%) of the children in the vitamin D_3_ group vs. 95 (33%) in the control group (HR=0.96 [0.72-1.27], p=0.76).

Allergic sensitization: The risk of allergic sensitization was not significantly affected by the vitamin D_3_ supplement assessed by either skin prick test (OR=1.24 [0.66-2.31], p=0.51) or specific-IgE (OR=1.55 [0.89-2.73], p=0.13).

Eczema development was unaffected by the intervention: vitamin D_3_ vs. placebo, 68 (23%) vs. 72 (25%); HR=0.90 [0.65-1.26], p=0.55.

*Safety*

In the vitamin D_3_ vs. control group, intrauterine death was observed in 1 (0%) vs. 3 (1%), any congenital malformations in 17 (5%) vs. 23 (8%), and child hospitalization after birth in 32 (11%) vs. 28 (10%) cases (**eTable 3**).

**DISCUSSION**

Maternal supplementation with 2800IU/day vs. 400IU/day of vitamin D_3_ during the third trimester of pregnancy did not result in a reduced risk of persistent wheeze through age 3 years in the offspring. However, a clinically important protective effect cannot be excluded as the lower limit of the confidence interval was 0.52. The possibility of a protective effect is further supported by secondary end-point analyses showing a significant reduction in number of wheezy episodes and an up-regulated neonatal airway immune profile. However, the development of upper and lower respiratory infections, allergic sensitization and eczema were unaffected by the vitamin D_3_ supplementation.

The main limitation of the study is a reduced statistical power to detect an effect on the primary end-point of persistent wheeze. In addition, the vitamin D_3_ supplementation dose may have been too low^30,31^ as suggested by the significant decreased risk of persistent wheeze per increase in maternal serum vitamin D level at cessation of the trial. The normal level of vitamin D in a mother during pregnancy for optimal immune and lung development is unknown and might be as high as 100-150nmol/L. Also, we may have begun supplementation too late. Initiating vitamin D_3_ supplementation at earlier pregnancy stages may be beneficial, as recent data in humans suggest that vitamin D affects fetal lung development as early as the start of the second trimester.^32^ However, our data suggesting no effect of early (pre-intervention) levels of vitamin D argue against this hypothesis. Finally, the study did not include postnatal supplementation of the children, which could have induced an additive effect if the effects of maternal supplementation declined postnatally.

The primary strength of the study is the single-center design with standardized diagnoses performed solely by the experienced COPSAC research pediatricians. Other multi-center studies have used various clinicians with different training and experience, which may introduce diagnostic heterogeneity. The longitudinal clinical assessments at both scheduled and acute visits to the research center accompanied by daily diary recordings of respiratory symptoms to capture disease burden between visits provided a highly specific primary end-point with strong data on age at onset, which is a major advantage compared with studies using retrospective or cross-sectional unspecific community-based diagnoses^7–9^. This approach is important as diagnosis of wheezy disorders in young children is heterogeneous outside research settings due to lack of objective tests and non-standardized diagnostic procedures^2^. Our close clinical surveillance resulted in a high follow-up rate of the children during the 3 year double-blinded period with >94% completing the 3-year-visit.

Vitamin D levels are associated with lifestyle factors such as diet, sun exposure, physical activity, and tobacco smoke exposure, which confer a risk of residual confounding in observational studies^33^. Our placebo controlled study design with unbiased randomization mitigates such confounding and allowed us to examine the isolated effect of vitamin D_3_ supplementation. In addition, the mothers had good adherence to the intervention with 74% taking >80% of the intervention tablets with no differences between intervention groups.

Vitamin D deficiency may lead to wheezy disorders by interfering with fetal lung cell maturation during pregnancy and subsequent lung function development^34^. An alternative mechanism founded on alterations of the airway microbiome by induction of the antimicrobial cathelicidin in bronchial epithelial cells has also been proposed^35^. It is a common belief that vitamin D possesses a range of immune regulatory properties, which are important for immune constitution in early life^5^. Our finding of a significantly up-regulated airway immune profile at age 1 month in the vitamin D_3_ supplemented group supports this hypothesis as the interrelationship between the relative up-regulations of Th1, Th2, Th17 and Treg mediators may lead to an increased wheeze propensity.

Vitamin D immune-modulatory mechanisms have been suggested to increase the frequency of respiratory infections,^12^ leading to virus-induced wheezing^36^. Such a pathway is not supported by our results showing no effect of vitamin D_3_ supplementation on either upper or lower respiratory tract infections.

Effective preventive strategies to alleviate the large burden of childhood wheezing and related disorders represent a major unmet clinical need. This RCT of vitamin D_3_ supplementation during pregnancy did not show a statistically significant effect on the primary outcome of persistent wheeze, although a clinically important protective effect cannot be excluded. Therefore, further studies with a larger sample size, higher dose, and potentially earlier intervention during pregnancy and postnatally should be performed to establish the potential benefits of vitamin D_3_ supplementation to pregnant women to reduce occurrence of wheezy disorders in the offspring.

*Conclusion*

The use of 2800IU/day of vitamin D_3_ during pregnancy, compared with 400IU/day, did not result in a statistically significant reduced risk of persistent wheeze in the offspring through age 3 years. However, interpretation of the study is limited by a wide confidence interval that includes a clinically important protective effect.

**ACKNOWLEDGEMENTS**

We gratefully express our gratitude to the children and families of the COPSAC_2010_ cohort study for all their support and commitment. We acknowledge and appreciate the efforts of the COPSAC research team.

The COPSAC biobank is publicly available at the Danish National Biobank ([www.biobankdenmark.dk](http://www.biobankdenmark.dk)) and data will become available in the Danish Data Archive (www.sa.dk).

The guarantor of the study is HB who is responsible for the integrity of the work as a whole, from conception and design to conduct of the study and acquisition of data, analysis and interpretation of data and writing of the manuscript. HB had full access to all the data in the study and takes responsibility for the integrity of the data and the accuracy of the data analysis. BC was responsible for data analysis and wrote the first draft of the manuscript. KB contributed to design of the study, interpretation of data and writing of the manuscript. JS and MAR contributed to the data analysis. SB was responsible for the analyzing levels of airway immune mediators and LH for analyzing maternal Vitamin D levels. All co-authors have contributed substantially to the analyses and/or interpretation of the data and have provided important intellectual input and approval of the final version of the manuscript. No honorarium, grant, or other form of payment was given to anyone to produce the manuscript.

The lead author affirms that the manuscript is an honest, accurate, and transparent account of the study being reported; that no important aspects of the study have been omitted; and that any discrepancies from the study as planned have been explained.

COPSAC is funded by private and public research funds all listed on [www.copsac.com](http://www.copsac.com/). The Lundbeck Foundation; Danish State Budget; Danish Council for Strategic Research; Danish Council for Independent Research and The Capital Region Research Foundation have provided core support for COPSAC. No pharmaceutical company was involved in the study. The funding organizations did not have any role in design and conduct of the study; collection, management, analysis, and interpretation of the data; preparation, review, or approval of the manuscript; and decision to submit the manuscript for publication.

All authors have completed the Unified Competing Interest form at [www.icmje.org/coi_disclosure.pdf](http://www.icmje.org/coi_disclosure.pdf) (available on request from the corresponding author). HB has received funds for research and for members of research staff from the above funds and has been paid as consultant for CHIESI. The remaining authors declare that (1) no authors have support from any medical company for the submitted work; (2) no authors have any relationship with companies that might have an interest in the submitted work in the previous 3 years; (3) their spouses, partners, or children have no financial relationships that may be relevant to the submitted work; and (4) no authors have any non-financial interests that may be relevant to the submitted work.

**REFERENCES**

1. Bisgaard H, Szefler S. Prevalence of asthma-like symptoms in young children. *Pediatr Pulmonol*. 2007;42(8):723-728. doi:10.1002/ppul.20644.

2. Eder W, Ege MJ, von Mutius E. The Asthma Epidemic. *N Engl J Med*. 2006;355:2226-2235. doi:10.1056/NEJMra054308.

3. Holick MF. Vitamin D deficiency. *N Engl J Med*. 2007;357(3):266-281. doi:10.1056/NEJMra070553.

4. Litonjua AA, Weiss ST. Is vitamin D deficiency to blame for the asthma epidemic? *Journal of Allergy and Clinical Immunology*. 2007;120(5):1031-1035. doi:10.1016/j.jaci.2007.08.028.

5. Litonjua AA. Childhood asthma may be a consequence of vitamin D deficiency. *Curr Opin Allergy Clin Immunol*. 2009;9(3):202-207. doi:10.1097/ACI.0b013e32832b36cd.

6. Chawes BL, Bønnelykke K, Jensen PF, Schoos A-MM, Heickendorff L, Bisgaard H. Cord blood 25(OH)-vitamin D deficiency and childhood asthma, allergy and eczema: the COPSAC2000 birth cohort study. *PLoS ONE*. 2014;9(6):e99856. doi:10.1371/journal.pone.0099856.

7. Camargo CA, Rifas-Shiman SL, Litonjua AA, et al. Maternal intake of vitamin D during pregnancy and risk of recurrent wheeze in children at 3 y of age. *Am J Clin Nutr*. 2007;85(3):788-795.

8. Devereux G, Litonjua AA, Turner SW, et al. Maternal vitamin D intake during pregnancy and early childhood wheezing. *Am J Clin Nutr*. 2007;85(3):853-859.

9. Baïz N, Dargent-Molina P, Wark JD, Souberbielle J-C, Annesi-Maesano I, EDEN Mother-Child Cohort Study Group. Cord serum 25-hydroxyvitamin D and risk of early childhood transient wheezing and atopic dermatitis. *J Allergy Clin Immunol*. 2014;133(1):147-153. doi:10.1016/j.jaci.2013.05.017.

10. Camargo CA, Ingham T, Wickens K, et al. Cord-Blood 25-Hydroxyvitamin D Levels and Risk of Respiratory Infection, Wheezing, and Asthma. *Pediatrics*. 2011;127(1):e180-e187. doi:10.1542/peds.2010-0442.

11. Jones AP, Palmer D, Zhang G, Prescott SL. Cord blood 25-hydroxyvitamin D3 and allergic disease during infancy. *Pediatrics*. 2012;130(5):e1128-e1135. doi:10.1542/peds.2012-1172.

12. Morales E, Romieu I, Guerra S, et al. Maternal vitamin D status in pregnancy and risk of lower respiratory tract infections, wheezing, and asthma in offspring. *Epidemiology*. 2012;23(1):64-71. doi:10.1097/EDE.0b013e31823a44d3.

13. Bisgaard H, Vissing NH, Carson CG, et al. Deep phenotyping of the unselected COPSAC2010 birth cohort study. *Clin Exp Allergy*. 2013;43(12):1384-1394. doi:10.1111/cea.12213.

14. Schulz KF, Altman DG, Moher D, for the CONSORT Group. CONSORT 2010 Statement: updated guidelines for reporting parallel group randomised trials. *BMJ*. 2010;340(mar23 1):c332-c332. doi:10.1136/bmj.c332.

15. Højskov CS, Heickendorff L, Møller HJ. High-throughput liquid-liquid extraction and LCMSMS assay for determination of circulating 25(OH) vitamin D3 and D2 in the routine clinical laboratory. *Clin Chim Acta*. 2010;411(1-2):114-116. doi:10.1016/j.cca.2009.10.010.

16. Maunsell Z, Wright DJ, Rainbow SJ. Routine Isotope-Dilution Liquid Chromatography-Tandem Mass Spectrometry Assay for Simultaneous Measurement of the 25-Hydroxy Metabolites of Vitamins D2 and D3. *Clinical Chemistry*. 2005;51(9):1683-1690. doi:10.1373/clinchem.2005.052936.

17. Bisgaard H, Hermansen MN, Loland L, Halkjaer LB, Buchvald F. Intermittent inhaled corticosteroids in infants with episodic wheezing. *N Engl J Med*. 2006;354(19):1998-2005. doi:10.1056/NEJMoa054692.

18. Bisgaard H, Bønnelykke K, Sleiman PMA, et al. Chromosome 17q21 gene variants are associated with asthma and exacerbations but not atopy in early childhood. *Am J Respir Crit Care Med*. 2009;179(3):179-185. doi:10.1164/rccm.200809-1436OC.

19. Bisgaard H, Hermansen MN, Bønnelykke K, et al. Association of bacteria and viruses with wheezy episodes in young children: prospective birth cohort study. *BMJ*. 2010;341:c4978.

20. Vissing NH, Chawes BLK, Bisgaard H. Increased risk of pneumonia and bronchiolitis after bacterial colonization of the airways as neonates. *Am J Respir Crit Care Med*. 2013;188(10):1246-1252. doi:10.1164/rccm.201302-0215OC.

21. Chawes BLK, Poorisrisak P, Johnston SL, Bisgaard H. Neonatal bronchial hyperresponsiveness precedes acute severe viral bronchiolitis in infants. *J Allergy Clin Immunol*. 2012;130(2):354-361.e3. doi:10.1016/j.jaci.2012.04.045.

22. Chawes BLK, Edwards MJ, Shamji B, et al. A novel method for assessing unchallenged levels of mediators in nasal epithelial lining fluid. *J Allergy Clin Immunol*. 2010;125(6):1387-1389.e3. doi:10.1016/j.jaci.2010.01.039.

23. Følsgaard NV, Chawes BL, Rasmussen MA, et al. Neonatal Cytokine Profile in the Airway Mucosal Lining Fluid Is Skewed by Maternal Atopy. *Am J Respir Crit Care Med*. 2012;185(3):275-280. doi:10.1164/rccm.201108-1471OC.

24. Følsgaard NV, Schjørring S, Chawes BL, et al. Pathogenic bacteria colonizing the airways in asymptomatic neonates stimulates topical inflammatory mediator release. *Am J Respir Crit Care Med*. 2013;187(6):589-595. doi:10.1164/rccm.201207-1297OC.

25. Chawes BLK, Stokholm J, Bønnelykke K, Brix S, Bisgaard H. Neonates with reduced neonatal lung function have systemic low-grade inflammation. *J Allergy Clin Immunol*. 2015;135(6):1450-1456.e1. doi:10.1016/j.jaci.2014.11.020.

26. Hanifin J, Rajka G. Diagnostic features of atopic dermatitis. *Acta Derm Venereol*. 1980;92:44-47.

27. Bisgaard H, Halkjaer LB, Hinge R, et al. Risk analysis of early childhood eczema. *J Allergy Clin Immunol*. 2009;123(6):1355-1360.e5. doi:10.1016/j.jaci.2009.03.046.

28. Halkjaer LB, Loland L, Buchvald FF, et al. Development of Atopic Dermatitis During the First 3 Years of Life: The Copenhagen Prospective Study on Asthma in Childhood Cohort Study in High-Risk Children. *Arch Dermatol*. 2006;142(5):561-566. doi:<p>10.1001/archderm.142.5.561</p>.

29. Rasmussen MA, Colding-Jørgensen M, Hansen LT, Bro R. Multivariate evaluation of pharmacological responses in early clinical trials – a study of rIL-21 in the treatment of patients with metastatic melanoma. *British Journal of Clinical Pharmacology*. 2010;69(4):379-390. doi:10.1111/j.1365-2125.2009.03600.x.

30. Hollis BW, Johnson D, Hulsey TC, Ebeling M, Wagner CL. Vitamin D supplementation during pregnancy: double-blind, randomized clinical trial of safety and effectiveness. *J Bone Miner Res*. 2011;26(10):2341-2357. doi:10.1002/jbmr.463.

31. Litonjua AA, Lange NE, Carey VJ, et al. The Vitamin D Antenatal Asthma Reduction Trial (VDAART): rationale, design, and methods of a randomized, controlled trial of vitamin D supplementation in pregnancy for the primary prevention of asthma and allergies in children. *Contemp Clin Trials*. 2014;38(1):37-50. doi:10.1016/j.cct.2014.02.006.

32. Kho AT, Sharma S, Qiu W, et al. Vitamin D related genes in lung development and asthma pathogenesis. *BMC Med Genomics*. 2013;6:47. doi:10.1186/1755-8794-6-47.

33. Theodoratou E, Tzoulaki I, Zgaga L, Ioannidis JPA. Vitamin D and multiple health outcomes: umbrella review of systematic reviews and meta-analyses of observational studies and randomised trials. *BMJ*. 2014;348:g2035.

34. Zosky GR, Berry LJ, Elliot JG, James AL, Gorman S, Hart PH. Vitamin D deficiency causes deficits in lung function and alters lung structure. *Am J Respir Crit Care Med*. 2011;183(10):1336-1343. doi:10.1164/rccm.201010-1596OC.

35. Yim S, Dhawan P, Ragunath C, Christakos S, Diamond G. Induction of cathelicidin in normal and CF bronchial epithelial cells by 1,25-dihydroxyvitamin D(3). *J Cyst Fibros*. 2007;6(6):403-410. doi:10.1016/j.jcf.2007.03.003.

36. Hollams EM. Vitamin D and atopy and asthma phenotypes in children. *Curr Opin Allergy Clin Immunol*. 2012;12(3):228-234. doi:10.1097/ACI.0b013e3283534a32.

**FIGURE LEGENDS**

**Figure 1: CONSORT study flow diagram.**

*Exclusion criterions were gestational age above week 26, any endocrine, cardiovascular, or nephrological disorders or vitamin D intake >600 IU/day.

**Figure 2: Effect of vitamin D_3_ supplementation on risk of persistent wheeze.**

Kaplan-Meier curve showing age at onset of persistent wheeze during the 3 years of double-blinded clinical follow-up of children born to mothers receiving Vitamin D_3_ vs. control during the third pregnancy trimester. The hazard ratio (HR) describes the unadjusted risk of persistent wheeze.

**Figure 3: Effect of maternal serum vitamin D_3_ level on risk of persistent wheeze.**

Kaplan-Meier curve showing age at onset of persistent wheeze during the 3 years of double-blind clinical follow-up stratified by tertiles of maternal post-intervention serum vitamin D_3_ levels measured 1 week postpartum. The hazard ratio describes risk of persistent wheeze per 10nmol/L increase in maternal serum vitamin D level after intervention.

1^st^ tertile: mean 50.00 nmol/L (range 15.7-72.0); 2^nd^ tertile: mean 87.86 nmol/L (range 72.1-103.0); 3^rd^ tertile: mean 131.60 nmol/L (range 104.0-258.0).

**Figure 4: Effect of vitamin D_3_ supplementation on the neonatal airway immune profile.**

The figures visualize the results of a principal component analysis (PCA), which extracts patterns of correlation between immune mediators shown in a loading plot (A) and the multivariate distribution of the children in relation to these patterns shown in a score plot (B).

**A:** PCA loading plot visualizing the correlation structure between 20 immune mediators, which are plotted as different markers colored according to immune function: light blue triangle = T helper lymphocyte type 1 (Th1) response (CCL2, CCL4, CXCL10, TNFα, IFNγ, IL-12p70), red diamond = Th2 response (CCL11, CCL13, CCL17, CCL22, CCL26, IL-4, IL-5, IL-13), brown square = Th17 response (CXCL8, IL-17α, IL-1β), dark blue circle = T regulatory response (TGF1β, IL-10), green triangle = lymphocyte expansion (IL-2).

0/0 is the base for interpreting how well a particular mediator is explained in the graph. Increasing length of the arrows from 0/0 corresponds to an increased proportion of the mediator being explained by the model.

Principal component 1 (PC1) reflects the overall immune activity regardless of individual mediators, accounting for 54% of the variation in data. PC2 reflects a pattern related to pertubation of CCL17, CCL2, CCL11, CCL13 and TGF1β versus IL-12p70, IL-10, IL-13, IL-5, IFNγ, IL1β, TNFα, IL4, IL2, IL-17α, accounting for additionally 8% of the variation. Thus, children with loading values above 0 on PC2 have an immune profile skewed towards CCL17, CCL2, CCL11, CCL13 and TGF1β, whereas children with loading values below 0 have an immune profile skewed towards IL-12p70, IL-10, IL-13, IL-5, IFNγ, IL-1β, TNFα, IL-4, IL-2 and IL-17α.

**B:** PCA score plot showing the similarity between participating children with respect to immune profile, where each circle corresponds to a child, and two points closely positioned have similar profile. The ellipses reflect the 2D distribution of children in the vitamin D (blue circles) versus control (green circles) group, where 0/0 is the multivariate average of the distribution. PC1 reflects a significant discrepancy between the groups (p=0.04), pointing at generally higher airway immune activity in children from mothers supplemented with vitamin D_3_ during pregnancy.

**TABLES**

**Table 1:** **Baseline characteristics.**

Baseline characteristics of the COPSAC_2010_ mother-child pairs, who participated in the vitamin D_3_ RCT.

|  | **All** | **Randomization** | |
| --- | --- | --- | --- |
|  |  | **Vitamin D_3_** | **Control** |
|  | **581** | **295 (51%)** | **286 (49%)** |
| **Child** |  |  |  |
| Gender, Male No. (%) | 298 (51) | 155 (53) | 143 (50) |
| Age at unblinding, Years (SD) | 4.1 (0.5) | 4.1 (0.5) | 4.1 (0.5) |
| White No. (%) | 555 (96) | 283 (96) | 272 (95) |
| Season of Birth |  |  |  |
| Winter No. (%) | 209 (36) | 106 (36) | 103 (36) |
| Spring No. (%) | 106 (18) | 53 (18) | 53 (18) |
| Summer No. (%) | 120 (21) | 63 (21) | 57 (20) |
| Fall No. (%) | 146 (25) | 73 (25) | 73 (26) |
| **Pregnancy** |  |  |  |
| Smoking No. (%) | 46 (8) | 20 (7) | 26 (9) |
| Alcohol ≥ 1 unit/week No. (%) | 89 (15) | 46 (16) | 43 (15) |
| Cat or Dog in the home during Pregnancy No. (%) | 202 (35) | 92 (31) | 110 (38) |
| Antibiotic usage during Pregnancy No. (%) | 205 (35) | 103 (35) | 102 (37) |
| Participation in the n-3 LCPUFA RCT No. (%) | 581 (100) | 295 (51) | 286 (49) |
| Pre-intervention serum vitamin D level, mean (SD), nmol/L | 76.4 (25.4) | 76.5 (25.5) | 76.4 (25.3) |
| **Birth** |  |  |  |
| Term Birth >37 weeks No. (%) | 561 (97) | 284 (96) | 277 (97) |
| Primi-parity No. (%) | 263 (45) | 122 (41) | 141 (49) |
| Intra-partum Antibiotics No. (%) | 183 (32) | 97 (33) | 86 (30) |
| Antibiotics to the Child No. (%) | 14 (2) | 5 (2) | 9 (3) |
| APGAR score 5 min. <10 No. (%) | 26 (5) | 14 (5) | 12 (4) |
| Child hospitalized after Birth No. (%) | 56 (10) | 29 (10) | 27 (9) |
| Caesarean Section No. (%) | 128 (22) | 69 (23) | 59 (20) |
| Emergency Section No. (%) | 71 (12) | 39 (13) | 32 (11) |
| Elective Section No. (%) | 57 (10) | 30 (10) | 27 (9) |
| **Socioeconomics** |  |  |  |
| Maternal age at Birth, mean (SD), years | 32.3 (4.3) | 32.5 (4.4) | 32.0 (4.3) |
| Maternal Asthma No. (%)^*^ | 152 (26) | 79 (27) | 73 (26) |
| Maternal Educational Level^**^ |  |  |  |
| Low No. (%) | 45 (8) | 20 (7) | 25 (9) |
| Medium No. (%) | 375 (65) | 186 (63) | 189 (66) |
| High No. (%) | 160 (27) | 88 (30) | 72 (25) |
| Household Annual Income^***^ |  |  |  |
| Low No. (%) | 54 (9) | 27 (9) | 27 (9) |
| Medium No. (%) | 304 (53) | 151 (51) | 153 (54) |
| High No. (%) | 222 (38) | 116 (40) | 106 (37) |

** History of doctor diagnosed asthma.*

*** Low (elementary school or college graduate), Medium (tradesman or medium length), High (university candidate).*

**** Low (below 50.000 Euro), Medium (50.000 – 110.000 Euro), High (above 110.000 Euro);*

*1 Euro = 1.07 USD.*

**Table 2: Primary and secondary end-point analyses.**

Effect of vitamin D_3_ supplementation during pregnancy on risk of persistent wheeze and related end-points in the offspring.

|  | **Vitamin D_3_** | | **Control** | | **Difference [95% CI]** | **Estimate [95% CI]** | **P-value** |
| --- | --- | --- | --- | --- | --- | --- | --- |
|  | Total, No. | Cases,  No. (%) | Total, No. | Cases, No. (%) |  |  |  |
| **Primary End-Point** | | | | | | | |
| Persistent wheeze, 0-3yrs | 295 | 47 (16%) | 286 | 57 (20%) | 0.04 [-0.02-0.10] | HR=0.76 [0.52-1.12] | .16 |
| Persistent wheeze, 0-3yrs, adjusted^*^ | 292 | 47 (16%) | 285 | 57 (20%) | 0.04 [-0.02-0.10] | aHR=0.75 [0.51-1.10] | .14 |
| **Secondary End-Points** | | | | | | | |
| Wheezy episodes, 0-3yrs^**^ | 287 | 5.9 (5.2-6.6) | 278 | 7.2 (6.4-8.1) | 1.3 [0.2-2.4] | IRR=0.83 [0.71-0.97] | .02 |
| Asthma, 3yrs | 278 | 32 (12%) | 271 | 47 (14%) | 0.02 [-0.03-0.08] | OR=0.82 [0.50-1.36] | .45 |
| Upper respiratory infections, 0-3yrs^***^ | 295 | 5.2 (4.8-5.5) | 284 | 5.3 (4.9-5.6) | 0.1 [-0.6-0.4] | IRR=0.99 [0.90-1.09] | .84 |
| Lower respiratory infections, 0-3yrs | 292 | 94 (32%) | 284 | 95 (33%) | 0.01 [-0.01-0.06] | HR=0.96 [0.72-1.27] | .76 |
| Eczema, 0-3yrs | 295 | 68 (23%) | 286 | 72 (25%) | 0.02 [-0.05-0.09] | HR=0.90 [0.65-1.26] | .55 |
| Allergic sensitization, 0-3yrs^****^ | | | | | | | |
| Skin prick test | 294 | 24 (8%) | 283 | 19 (7%) | -0.01 [-0.06-0.03] | OR=1.24 [0.66-2.31] | .51 |
| Specific IgE | 289 | 34 (12%) | 278 | 22 (8%) | -0.04 [-0.09-0.02] | OR=1.55 [0.89-2.73] | .13 |

** Adjusted for gender, birth season, n-3 LCPUFA RCT, and maternal vitamin D at time of randomization.*

*** Reported as mean no. episodes (95% CI) at age 0-3yrs.*

**** Reported as mean no. episodes per year (95% CI) at age 0-3yrs.*

***** Allergic sensitization at 6 and/or 18 months positive against dog, cat, milk or egg.*
