# Supplementary material for: Prenatal vitamin D supplementation reduces risk of asthma/recurrent wheeze in early childhood: A combined analysis of two randomized controlled trials
Source: PLoS One. 2017 Oct 27;12(10):e0186657. doi: 10.1371/journal.pone.0186657 (PMC5659607; doi:10.1371/journal.pone.0186657)
Supplement: S8 Text — (DOCX) [file pone.0186657.s010.docx]

Supplement to:

Effect of High-dose Vitamin D3 Supplementation during Pregnancy on Risk of Persistent Wheeze in the Offspring

*A Randomized Controlled Trial*

Chawes BL, Bønnelykke K, Stokholm J, Vissing NH, Bjarnadóttir E, Schoos A-MM, Wolsk HM, Pedersen TM, Vinding RK, Thorsteinsdóttir S, Arianto L, Hallas HW, Heickendorff L, Brix S, Rasmussen MA, Bisgaard H.

This supplement contains the following items:

1. Original protocol and protocol changes.

2. Original statistical analysis plan and changes to the analysis plan

# 1. Original protocol and protocol changes

## Original Protocol

***The following is an English translation of the original protocol in Danish.***

Local Ethics Committee: H-B-2009-014; Approved: 23-02-2009

Danish Health and Medicines Authority: 2612-3959; Approved: 23-02-2009

ClinicalTrials.gov: NCT00856947

EudraCT: 2008-007871-26

**Aim**

To investigate whether supplementation with high-dose vitamin D during third trimester of pregnancy has a favorable effect on the development of asthma and related disorders in the offspring.

**Hypothesis**

High-dose vitamin D_3_ supplementation during third trimester of pregnancy will reduce the risk of developing asthma in the offspring.

**Background**

Asthma, eczema and allergy are the most common chronic diseases among children and over the past 40 years, the incidence of these diseases has increased in industrialized countries through yet unknown factors in the environment.

Decreased levels of maternal vitamin D in pregnancy and thereby reduced fetal vitamin D levels in utero are among the early environmental exposures suspected to have an influence on the increased incidence of asthma in children.[1] Based on epidemiological studies, a high intake of vitamin D during pregnancy has been associated with protective effects on asthmatic symptoms in young children.[2,3] Preliminary results of a newer study indicates twice the risk of asthmatic symptoms in preschool children with low vitamin D levels at birth compared to children with a high level of vitamin D levels at birth.[4]

The results are consistent with several other studies, which suggest that the population in westernized countries have a reduced supply and level of vitamin D leading to an increased risk of various diseases. E.g., vitamin D levels in the fetus has been associated with the development of schizophrenia, diabetes mellitus and bone development.[5–7] Furthermore, high levels of vitamin D in adults appears to protect against a number of diseases, including bone diseases and cancer. [8–10]

The reason for these reduced levels of vitamin D may be found in the lifestyle of modern society. The majority of our vitamin D supply derives from sun exposure, and because of increasing awareness of harmful effects of sun exposure in relation to skin cancer, our supply of vitamin D has been markedly reduced. This is a recent development, which has led to the hypothesis that the current levels of vitamin D is too low according to the level for which we are genetically programmed.
Vitamin D level is however associated with and highly influenced by other factors as well. Therefore, it is necessary to conduct controlled, blinded studies on the effect of vitamin D supplementation to provide sufficient basis for future recommendations.

**Method and trial procedure**

The women are recruited from the COPSAC_2010_ cohort; Local Ethics Committee (H-B-2008-093), Danish Data Protection Agency (2008-41-2599).

The study is a double-blinded, placebo-controlled, randomized parallel group design. 800 pregnant women will be randomized in a 1:1 ratio to intake of either high dose vitamin D supplementation or placebo according to one of the following regimes:

1. Placebo (+ guidance in recommended supplement of vitamin D (400units daily)) or
2. High dose vitamin D supplement (2400units daily) (+ guidance in recommended supplement of vitamin D (400units daily))

The regimes are administered orally as 2 tablets daily.

Blinding and randomization are carried out by the Capital Region Pharmacy and stratified according to treatment group in the fish oil intervention study (ClinicalTrials.gov: NCT00798226). This allows for equal numbers receiving high dose vitamin D supplementation in both the fish oil active group and the fish oil placebo group.

The intervention is initiated at the beginning of the third trimester (pregnancy week 24) and continued until 1^st^ visit to the COPSAC clinic after birth at week 1-2 postpartum. At the clinical visit in pregnancy week 24, the women will be provided with the intervention treatment and interviewed about current daily vitamin D intake and history of diseases likely to influence vitamin D levels. At pregnancy week 36 adherence to the regime will be assessed by interview at the COPSAC clinic. Furthermore, the women will be instructed to return the remaining tablets at the end of the intervention for evaluation of their compliance.

At pregnancy week 24 and 1^st^ visit after birth a blood sample will be drawn from the mother in order to measure 25-OH-vitamin D, total calcium, parathyroid-hormone (PTH) and alkaline phosphatase.

**Inclusion criteria**

The study population consists of healthy pregnant women and their children participating in the COPSAC_2010_ cohort. Vitamin D supplements are administered during the third pregnancy trimester. The women will be included in the study independent of residence, age, race and social status during week 22-26 of pregnancy.

**Exclusion criteria**

Pregnant women are excluded from the trial, if they carry a disease leading to an increased risk of potential side effects from high-dose vitamin D supplementation: Endocrinologic disease in the form of calcium metabolic disorders, parathyoroidea disease, thyroid disorders or type 1 diabetes; Tuberculosis; Sarcoidosis or illness requiring chronic treatment with diuretics or heart medications, including calcium channel blockers or if they have a current intake of vitamin D supplements over the recommended dose.

**Risks and disadvantages:**

Known potential adverse effects of vitamin D intoxication is hypercalcemia and accompanying symptoms such as loss of appetite, nausea, vomiting, weight loss, headache, lethargy, fatigue, confusion and renal impairment. These side effects are not found by the administration of vitamin D in physiological doses. Vitamin D intoxication occurs only by the intake of very high doses of Vitamin D (4 times higher doses than administered in our study). In order to avoid administering vitamin D supplements to women with a high initial level, women with an intake above the recommended dose in the previous 6 months are excluded. Expected disadvantages related to blood sample procedures and are temporary in nature without the risk of permanent injury.

**Ethical aspects**

Oral vitamin D supplement has been shown to be safe and non-toxic in many randomized trials, including studies involving pregnant women. The risk of adverse effects in the pregnant woman or the fetus is suspected to be minimal. Based on the previous studies, it is expected that a large proportion of the participating women will have a daily low Vitamin D level, and therby vitamin D supplementation to these women will be a health benefit. The control group receive recommended dose of vitamin D, and ethical problems in relation to sufficient treatment of the control group is thereby not a problem.

We believe that the study as outlined above is ethically acceptable and randomized trials of vitamin D supplements are necessary for future recommendations of vitamin D intake.

**Reference List**

1 Litonjua AA, Weiss ST. Is vitamin D deficiency to blame for the asthma epidemic? *J Allergy Clin Immunol* 2007;**120**:1031–5. doi:10.1016/j.jaci.2007.08.028

2 Devereux G, Litonjua AA, Turner SW, *et al.* Maternal vitamin D intake during pregnancy and early childhood wheezing. *Am J Clin Nutr* 2007;**85**:853–9.

3 Camargo CA, Rifas-Shiman SL, Litonjua AA, *et al.* Maternal intake of vitamin D during pregnancy and risk of recurrent wheeze in children at 3 y of age. *Am J Clin Nutr* 2007;**85**:788–95.

4 Camargo CA, Ingham T, Wickens K, *et al.* Cord-Blood 25-Hydroxyvitamin D Levels and Risk of Respiratory Infection, Wheezing, and Asthma. *Pediatrics* 2011;**127**:e180–7. doi:10.1542/peds.2010-0442

5 McGrath J, Selten J-P, Chant D. Long-term trends in sunshine duration and its association with schizophrenia birth rates and age at first registration — data from Australia and the Netherlands. *Schizophr Res* 2002;**54**:199–212. doi:10.1016/S0920-9964(01)00259-6

6 Hyppönen E, Läärä E, Reunanen A, *et al.* Intake of vitamin D and risk of type 1 diabetes: a birth-cohort study. *The Lancet* 2001;**358**:1500–3. doi:10.1016/S0140-6736(01)06580-1

7 Javaid M, Crozier S, Harvey N, *et al.* Maternal vitamin D status during pregnancy and childhood bone mass at age 9 years: a longitudinal study. *The Lancet* 2006;**367**:36–43. doi:10.1016/S0140-6736(06)67922-1

8 Bischoff-Ferrari HA, Giovannucci E, Willett WC, *et al.* Estimation of optimal serum concentrations of 25-hydroxyvitamin D for multiple health outcomes. *Am J Clin Nutr* 2006;**84**:18–28.

9 Holick MF. Vitamin D deficiency. *N Engl J Med* 2007;**357**:266–81. doi:10.1056/NEJMra070553

10 Adams JS, Clemens TL, Parrish JA, *et al.* Vitamin-D Synthesis and Metabolism after Ultraviolet Irradiation of Normal and Vitamin-D-Deficient Subjects. *N Engl J Med* 1982;**306**:722–5. doi:10.1056/NEJM198203253061206

## Changes to the protocol

Changes to the original protocol are indicated in <https://clinicaltrials.gov/ct2/show/NCT00856947>

Briefly, these encompass introduction of novel assessments, including neurological development, growth, systemic immune status and airway mucosal immune status.

# 2. Original statistical analysis plan and changes to the analysis plan

## Original statistical analysis plan

***Outcome definition***

***Primary outcome***

***Persistent wheeze***

***Description:*** Age at onset of persistent wheeze diagnosed according to a predefined algorithm of recurrent troublesome lung symptoms, response to treatment and relapse after withdrawal of treatment

***Secondary outcomes***

***Asthma exacerbations***

***Description:*** Age at onset of severe asthma exacerbations diagnosed by predefined criteria of acute severe asthma requiring oral/high dose inhaled steroids or acute hospital contact

***Eczema***

***Description:*** Age at onset of eczema diagnosed prospectively by research doctors according to predefined algorithm based upon Hanifin and Rajka criteria

***Allergic sensitization***

***Description:*** Allergic sensitization at 6 and/or 18 months of age assessed by skin prick test and specific IgE in blood

***Infections***

***Description:***

Main analysis: Number of lower respiratory tract infections registered in daily diaries

Secondary analyses: Acute otitis media, number of upper respiratory tract infections, number of other infections, total number of infections

***Statistical analyses:***

The effect of high-dose Vitamin D_3_ supplementation on age at onset of persistent wheeze, lower respiratory infections, and eczema is analyzed by Cox proportional hazards regression, where p-values correspond to Wald tests. The children are retained in the model from birth until age of diagnosis, drop out, or age at their last clinic visit before the RCT was unblinded.

The effect of Vitamin D_3_ supplementation on the cross-sectional end-points asthma and allergic sensitization is analyzed by logistic regression, whereas the effect on number of wheezy episodes and upper respiratory infections is analyzed by a generalized estimating equation (GEE) Poisson regression model.

The effect on airway immunology is analyzed by calculating geometric mean ratios of each mediator in the high-dose Vitamin D_3_ vs. control group and by a principal component analysis (PCA) capturing the overall immunological trends in the data and their relation to the intervention analyzed by Wilcoxon rank sum test. Initially, the mediator levels were log-transformed. Prior to the PCA the variables ware scaled to unit variance.

The primary analysis of persistent wheeze is presented crude and adjusted for sex, birth season, maternal Vitamin D level at randomization, and the n-3 LCPUFA RCT.

A significance level of 0.05 is used in all types of analyses.

## Changes to the statistical analysis plan

***Power calculation***

A power calculation was performed based upon the available number of 587 children participating in the Vitamin D trial. The Vitamin D_3_ RCT had a 65% power to detect a difference between the treatment groups (alpha=0.05, two-tailed) based on the 587 included children, an effect of 0.5 in the Vitamin D_3_ supplementation group, and a 12% expected frequency of persistent wheeze in the control group.

***Additional secondary endpoints:***

The novel assessments introduced in the cohort resulted in additional secondary end-points:

***Airway mucosal immune status***

***Description:*** Immune status measured in airway mucosal lining fluid at 4 weeks and 2 years of age (combined assessments by prinicipal component analyses for each age point)

***Systemic immune status***

***Description:***

Main analysis:

- Immune status at 18 months measured in stimulated whole blood as cytokine release (combined assessments by principal component analyses)

Secondary analysis:

- Composition of immune cell subsets in whole blood at birth and at 18 months of age

***Neurological development 0-3 years***

***Description:***

Main analysis:

- Cognitive development assessed at 2½ years using the cognitive part of Bayley Scales of Infant and Toddler Development, third edition

Secondary analyses:

- Milestone development monitored prospectively by the parents using a registration form based on The Denver Development Index and WHO milestones registration (combined assessment by principal component analysis)
- Language development assessed at 1 and 2 years of age with the Danish version of The MacArthur Bates Communicative Developmental Inventory (CDI)
- The child´s general development (language, fine and gross motor, social and problem solving) at 3 years of age assessed with Ages and stages Questioner, third edition (ASQ-3)

***Growth***

***Description:***

Main analysis:

- Body composition (fat mass and bone mineral density) assessed by DEXA scan at 3 years of age

Secondary analysis:

- Development of BMI from birth to 3 years assesses longitudinally in the research clinic
